# Developing SHP2-based combination therapy for KRAS-amplified cancer

**DOI:** 10.1172/jci.insight.152714

**Published:** 2023-02-08

**Authors:** Tianxia Li, Osamu Kikuchi, Jin Zhou, Yichen Wang, Babita Pokharel, Klavdija Bastl, Prafulla Gokhale, Aine Knott, Yanxi Zhang, John G. Doench, Zandra V. Ho, Daniel V.T. Catenacci, Adam J. Bass

**Affiliations:** 1 Department of Medical Oncology, Dana-Farber Cancer Institute, Boston, Massachusetts, USA.; 2 Herbert Irving Comprehensive Cancer Center, Columbia University Medical Center, New York, New York, USA.; 3 Experimental Therapeutics Core and Belfer Center for Applied Cancer Science, Dana-Farber Cancer Institute, Boston, Massachusetts, USA.; 4 Cancer Program, The Broad Institute of MIT and Harvard, Cambridge, Massachusetts, USA.; 5 Department of Medicine, University of Chicago Medical Center, Chicago, Illinois, USA.; 6 Department of Medicine, Harvard Medical School, Boston, Massachusetts, USA.; 7 Department of Medicine, Brigham and Women’s Hospital, Boston, Massachusetts, USA.

**Keywords:** Oncology, Drug therapy, Oncogenes, Signal transduction

## Abstract

Gastroesophageal adenocarcinomas (GEAs) harbor recurrent amplification of *KRAS*, leading to marked overexpression of WT KRAS protein. We previously demonstrated that SHP2 phosphatase, which acts to promote KRAS and downstream MAPK pathway activation, is a target in these tumors when combined with MEK inhibition. We hypothesized that SHP2 inhibitors may serve as a foundation for developing novel combination inhibitor strategies for therapy of *KRAS*-amplified GEA, including with targets outside the MAPK pathway. Here, we explore potential targets to effectively augment the efficacy of SHP2 inhibition, starting with genome-wide CRISPR screens in *KRAS*-amplified GEA cell lines with and without SHP2 inhibition. We identify candidate targets within the MAPK pathway and among upstream RTKs that may enhance SHP2 efficacy in *KRAS*-amplified GEA. Additional in vitro and in vivo experiments demonstrated the potent cytotoxicity of pan-ERBB kinase inhibitions in vitro and in vivo. Furthermore, beyond targets within the MAPK pathway, we demonstrate that inhibition of CDK4/6 combines potently with SHP2 inhibition in *KRAS*-amplified GEA, with greater efficacy of this combination in *KRAS*-amplified, compared with *KRAS*-mutant, tumors. These results suggest therapeutic combinations for clinical study in *KRAS*-amplified GEAs.

## Introduction

Gastroesophageal adenocarcinomas (GEAs) were responsible for more than 1 million new cases of cancer in 2020 and an estimated 769,000 deaths, making them, collectively, the fifth most frequently diagnosed cancer and the third leading cause of death due to cancer (1). Metastatic GEAs have dismal outcomes, with median overall survival of less than 12 months (2). Despite vigorous effort to improve systemic treatment, chemotherapy against GEA still relies heavily on cytotoxic agents, more recently complemented with immune checkpoint inhibitors (3), making improved targeted therapy a clear need.

Recent studies from The Cancer Genome Atlas and other groups reported molecular classification of gastric cancer using different modalities, such as somatic copy-number analysis, whole-exome sequencing, and mRNA-Seq (4–6). These results showed that a molecular subtype with chromosomal instability (CIN) is the most common subtype of GEA and is characterized by extensive aneuploidy and lack of somatic hypermutation. Within CIN-subtype GEA, the oncogene *KRAS*, which is renowned for activation by missense mutation in many cancers, is recurrently subject to focal high-level amplification without mutation. This *KRAS* amplification is frequently found in CIN-type gastric cancer (GC; 14.3%) and esophageal adenocarcinoma (10.4%).

In our prior publication (7), we pursued functional study of *KRAS*-amplified (*KRAS*-amp) GEA and found that these tumors, relative to *KRAS*-mutant (*KRAS*-mut) GEA models, are relatively insensitive to MEK inhibition. Deeper study revealed that MEK inhibition led to adaptive resistance resulting from marked activation of KRAS and the resulting induction of the PI3K-AKT pathway. Because we found the induction of KRAS activation in the setting of marked overexpression of WT KRAS mediated this adaptive resistance, we evaluated the potential to target physiologic mediators of KRAS activation to enhance efficacy of MEK therapy. SHP2, encoded by the *PTPN11* gene, is a tyrosine phosphatase that acts downstream of RTK and directly promotes physiologic activation of KRAS. SHP2 promotes nucleotide exchange in KRAS proteins, leading to transition from the inactive GDP-bound form to the active GTP-bound form in concert with guanine nucleotide exchange factors such as SOS1 (7, 8). Subsequently, SHP2 activity, by promoting KRAS-GTP, leads to activation of the RAS/MAPK and PI3K pathways (9). In our prior study (7), we found that targeting either SOS1 or SHP2 could abrogate adaptive activation of KRAS after MAPK pathway inhibition, thus potentiating the effects of MAPK therapy. In that study, we used a novel allosteric SHP2 inhibitor (SHP2i), SHP099, in combination with the MEK inhibitor (MEKi) trametinib and demonstrated combinatorial efficacy in *KRAS*-amp GEA cell lines in vivo and in vitro. Furthermore, efficacy of SHP2 inhibition with MAPK therapy has been reported in cancers with *KRAS* mutation (10–13). SHP2 inhibition is also being evaluated for its ability to block adaptive resistance to other targeted therapies, including inhibitors of tyrosine kinase inhibitors (14).

Given the centrality of MAPK signaling to KRAS-driven cancer, specifically the *KRAS*-mut cancers that have been the focus of nearly all preclinical and clinical studies, considerable effort has been made to develop combination therapies in which a secondary agent is added to a backbone of MAPK inhibition, most typically with pharmacologic inhibitors of MEK1/2. Although these combinations often show striking efficacy in preclinical models, the translation of these combinations has been challenging, in part due to on-target toxicity from inhibitors of the MAPK pathway (15). Consistent with these efforts in KRAS-driven cancer, we evaluated SHP2 therapy in our previous study as an adjunct to MEK therapy in *KRAS*-amp GEA. However, beyond its potential role in combination with MAPK inhibition, we hypothesize that SHP2 inhibition may serve as a foundation to develop additional therapeutic combinations for *KRAS*-amp tumors. Indeed, our prior studies (7) with *KRAS*-amp models demonstrated that in the *KRAS*-amp state, SHP2 monotherapy could substantially block MAPK signaling, as measured by inhibition of p-ERK — results not seen to the same degree with SHP2 therapy in *KRAS*^G12D^ mutant models. These results are consistent with our model whereby the overexpressed KRAS protein present in *KRAS*-amp cancers is still susceptible to the physiologic control mechanisms. As such, blockade of targets such as SHP2 may have particular efficacy with an oncogenic overexpression of WT KRAS protein relative to a tumor with a mutant KRAS protein whose cycling between active and inactive forms is dysregulated. Therefore, we aimed in this study to perform an unbiased query of the potential to develop combination therapies for *KRAS*-amp GEA integrating SHP2 inhibition with secondary targets outside the MAPK pathway.

## Results

### Genome-wide CRISPR screen identifies targets that potentiate SHP2 inhibition.

As a systematic way to define candidate targets to enhance the efficacy of SHP099 in *KRAS*-amp GEA, we started with a genome-wide loss-of-function CRISPR screen to define genes whose loss enhances the antiproliferative effects of SHP099. We conducted a screen using a genome-wide sgRNA library in the presence or absence of SHP2i SHP099 in 2 *KRAS*-amp GC cell lines (KE-39, HUG1-N). Prior to the screen, we determined the concentration of SHP099 to be used in the screening, aiming to find a dose with evidence of target engagement and with sufficient proliferation in *KRAS*-amp models to enable the discovery of secondary targets to enhance activity (Supplemental Figure 1, A–C; supplemental material available online with this article; https://doi.org/10.1172/jci.insight.152714DS1). Because SHP2 promotes RAS activation (9), we used KRAS protein activity, determined by querying GTP-bound forms of KRAS and p-ERK as biomarkers, to assess the efficacy of SHP2 inhibition.

First, we found that treatment with 5 μM SHP099 effectively reduced the GTP-bound active form of KRAS induced by 25 nM trametinib. Without trametinib, 5 μM SHP099 could block p-ERK without leading to a compensatory increase in KRAS GTP, consistent with partial activity against KRAS (Supplemental Figure 1, A and B). Although this level of drug could attenuate growth, the *KRAS*-amp cells could still proliferate, thus allowing us to query targets that may augment SHP2 efficacy (Supplemental Figure 1C).

**Figure 1:**
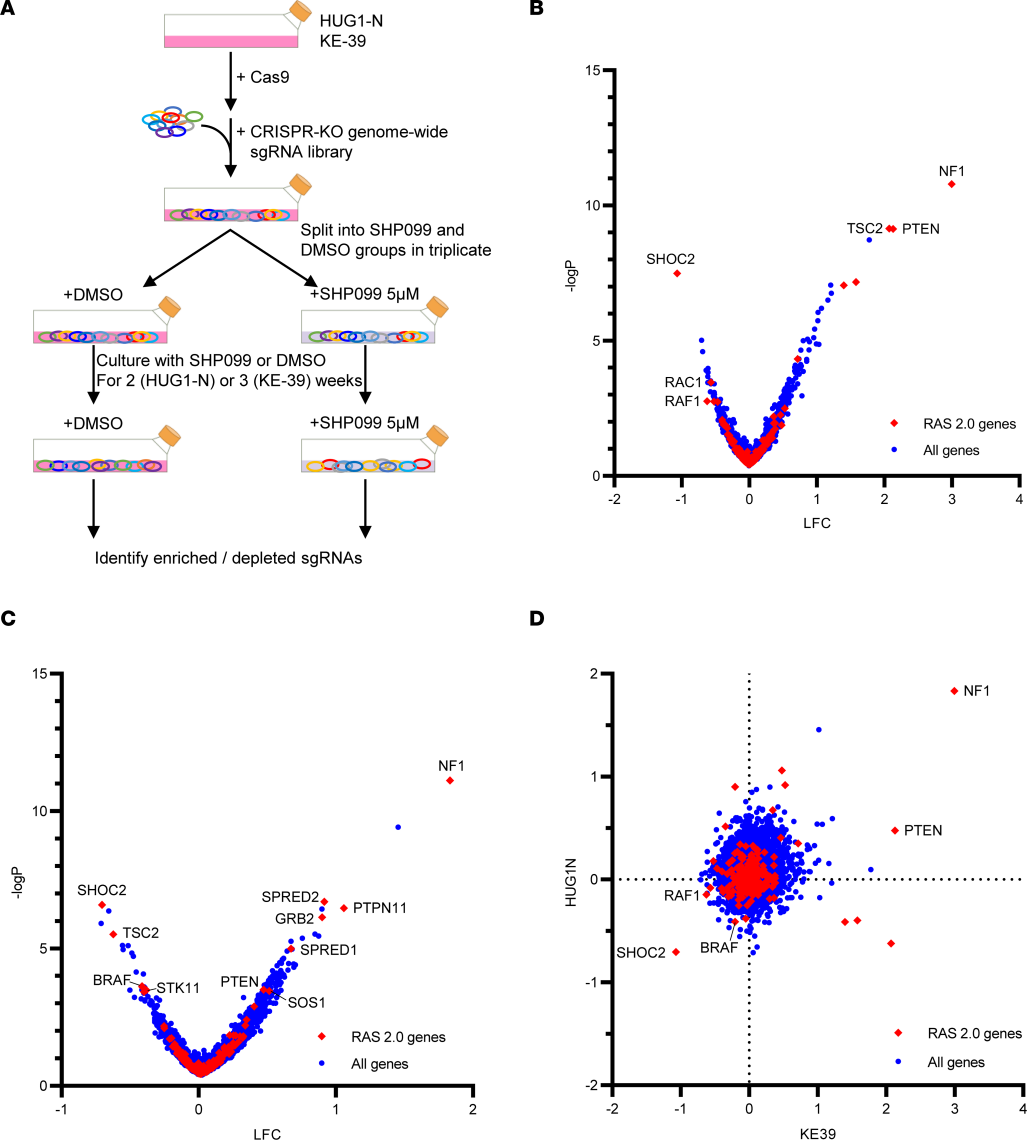
Genome-wide screen identified candidate targets that enhance sensitivity to an SHP2i. (**A**) Experimental scheme of genome-wide CRISPR anchor screening. KE-39 and HUG1-N *KRAS*-amp GEA cells were transduced with a genome-wide Brunello CRISPR-KO library and exposed to either 5 μM SHP099 or vehicle control to identify mediators of SHP099 sensitivity. Surviving cells from each virus infection were isolated, and the sgRNA sequences were amplified by PCR and sequenced. (**B** and **C**) Volcano plots of (**B**) HUG1-N and (**C**) KE-39 showing genes conferring resistance and sensitivity to SHP099. The LFC and –log_10_ (FDR) were calculated using the mean of the 4 sgRNAs per gene. *P* values were calculated using hypergeometric distribution. Red dots show RAS-related genes (RAS pathway 2.0). (**D**) Comparison of HUG1-N and KE-39 LFC data with a subset of RAS-related genes (RAS pathway 2.0 in red) and all other genes (blue). The list of top genes is provided in Supplemental Table 1.

Cas9-expressing KE-39 and HUG1-N cells were transduced with the Brunello genome-scale CRISPR-KO sgRNA library (16), which contains 4 sgRNAs per gene. We infected cells with an MOI of 0.3–0.5 prior to antibiotic selection to remove uninfected cells. We then maintained a cell population with coverage of 500 cells/sgRNA per condition. We cultured cells with 5 μM SHP099 or DMSO control for 2 weeks (HUG1-N) or 3 weeks (KE-39) in triplicate, with the difference in timing reflecting the rates of growth of these cells in vitro (Figure 1A). At the completion of the screen for each replicate, we obtained 40 million cells (representing an average 500-fold coverage of the library) for genomic DNA extraction, which were then submitted for deconvolution by next-generation sequencing.

To identify genes whose depletion modified the response to SHP2i, we averaged the measured abundance of sgRNAs targeting each gene to calculate gene-level scores, and we calculated LFC between SHP099-treated and control groups to determine the preferential dependence of each gene in the presence of SHP099 (Figure 1, B–D). The most highly enriched sgRNAs (i.e., genes whose silencing induced resistance to SHP2 inhibition) were directed at *NF1*, which is a RAS-specific GTPase-activating protein whose loss leads to aberrant RAS activation (17). The observation that *NF1* depletion rescues SHP2 inhibition provided reassurance about the quality of our CRISPR screen. In contrast to *NF1*, among the most highly depleted sgRNAs (i.e., sgRNAs in which gene silencing enhances the antiproliferative effects of SHP099) were those directed at SHOC2, a protein that dephosphorylates a critical inhibitory site on RAF kinases (namely, ARAF, BRAF, and CRAF) (18), where loss of the gene would inhibit RAS-mediated MAPK activation. This result highlights the importance of additional reduction of MAPK activity to complement SHP2 inhibition in *KRAS*-amp GEA.

Before deeper analysis of the potential “hits” from this screen, we decided to perform a secondary screen in a larger number of cell lines. Therefore, we generated a larger gene set of candidates that may augment SHP2 efficacy, based upon our genome-wide screen. Using the average log_2_ fold change (LFC) in guide detection in both KE-39 and HUG-1N data (Figure 1, B and C), we identified 244 genes in which genetic targeting contributes to enhance sensitivity to SHP099 in 1 or both of the 2 cell lines in our genome-wide screen (Supplemental Table 1).

### Secondary screen confirms KO of upstream and downstream genes has additive cytotoxicity to SHP2 inhibition.

**Figure 2:**
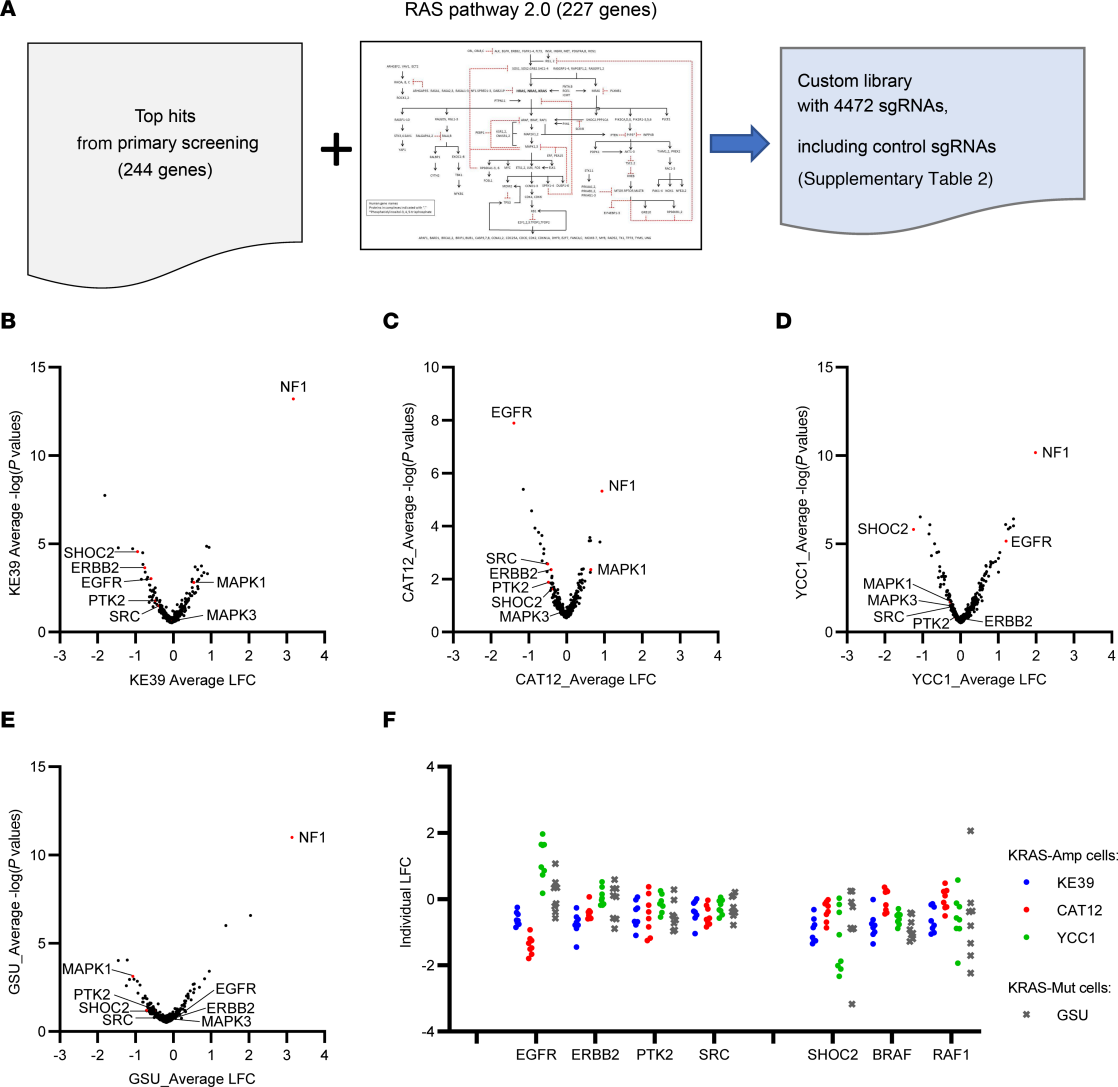
Secondary screen showed both genes upstream and downstream of KRAS are potential targets to enhance SHP2i efficacy. (**A**) Experimental scheme of secondary screening with a focused custom library. (**B**–**E**) Volcano plots of *KRAS*-amp cell lines and a *KRAS*-mut cell line, GSU, showing different RTK and MAPK genes conferring resistance and sensitivity to SHP099. (**B**–**D**) KE-39 (**B**), CAT12 (**C**), and YCC1 (**D**), which have *KRAS* amplification. (**E**) GSU with *KRAS*^G12D^ mutation served as a control. (**F**) LFCs of individual sgRNA for key genes of RTK and MAPK pathway within the indicated cell lines.

We then prepared for our secondary screen in an additional set of *KRAS*-amp cell lines with the goal of also performing the screen using a *KRAS*-mut GC cell line as a comparator. For this screen, we designed a custom, focused, secondary screening, CRISPR/Cas9 sgRNA library. Because we found that many hits were from the RTK/RAS/MAPK pathway and because of our interest in systematically interrogating targets in this pathway, we decided to include 227 RTK/RAS/MAPK genes of RAS pathway 2.0 curated by the National Cancer Institute, as well as the top 244 synthetic lethal candidate genes from the primary screen that were nominated according to their differential sensitivity scores by the STARS algorithm (19) (Supplemental Table 2). Using an all-in-one vector system encoding both Cas9 and sgRNAs, we generated a library of 4,472 sgRNAs (in this case, using 8 sgRNAs per gene; Supplemental Table 3) with additional guides added to the library as positive and negative controls. In addition to those targeting genes of interest (Figure 2A), we screened 3 *KRAS*-amp cancer cell lines, including 2 cancer cell lines (KE-39 and YCC-1) and 1 patient-derived cell line (CAT12) (Figure 2, B–D). We also included a *KRAS*-mut cell line as a comparison (GSU, with *KRAS*^G12D^) (Figure 2E).

After completion of the secondary screen, we first evaluated the KE-39 cell line because it was tested in both the genome-wide and focused screens. The comparison demonstrated reproducibility of the 2 screens when we focused upon the shared RAS-related gene set (Supplemental Figure 2A). Indeed, we found *BRAF*, *CRAF* (*RAF1*), and *SHOC2* among the top depleted genes (denoting sensitization to SHP2i), and *NF1* and *PTEN* were among the top enriched hits (denoting resistance). We then evaluated the composite group of *KRAS*-amp models from our secondary screen. Again, *NF1* was the top enriched gene when silenced. On average, the top 10 depleted genes were *SHOC2*, *RIC1*, *GAREM1*, *CRKL*, *RAPGEF1*, *RUNX1*, *BRAF*, *TCF7L2*, *CHD2*, and *VPS16* in the *KRAS*-amp cells. These included genes related to the MAPK pathway but also others, including *RIC1*, *GAREM1*, *CRKL*, *RUNX1*, *TCF7L2*, *CHD2*, and *VPS16* (Supplemental Table 4).

Given our focus on finding targets that could be readily translated into therapeutics, we focused our analysis on those of most ready translational relevance. SgRNAs of multiple MAPK pathway genes (namely, *SHOC2*, *BRAF*, and *RAF1*) encoding several RTKs, such as *EGFR*, *ERBB2*, *SRC*, and *PTK2* (encoding focal adhesion kinase [FAK]), were relatively depleted in the SHP099-treated cells when compared with vehicle control. Although none of these genes except *SHOC2* are universal among the *KRAS*-amp different cell lines, these results clearly indicate that targets related to cellular signaling both upstream and downstream of KRAS are potential modes of enhancing SHP2 therapy (Figure 2F; Supplemental Figure 2, B and C; and Supplemental Table 5).

We also compared our results from the *KRAS*-amp lines with that from the same secondary library evaluated in the GSU (*KRAS*^G12D^) (Figure 2E and Supplemental Figure 2D). As in the *KRAS*-amp state, genes directly related to MAPK signaling scored highly in the GSU model (e.g., *BRAF, RAF1*). By contrast, silencing of distinct RTKs showed minimal effects on GSU when added to SHP2 inhibition. Although these screening results broadly indicated that direct MAPK blockade may be the optimal target to enhance SHP2 efficacy, they did suggest additional targets for evaluation and suggested that upstream RTK inhibition may better complement SHP2i in *KRAS*-amp, compared with *KRAS*-mut, GEA.

### SHP2 inhibition moderately sensitizes KRAS-amp GC cells to ERBB and SRC inhibitors, not FAK inhibitor.

Next, we examined combination strategies with SHP099 in *KRAS*-amp GEA, starting with those candidate targets upstream of RAS. We specifically chose the tyrosine kinase inhibitors lapatinib, bosutinib, and defactinib to inhibit SHP2 and either ERBB families, SRC families, or FAK, respectively, given that these kinases scored in our screen. We used 10 nM MEK1/2 inhibitor trametinib as a positive control, given the established capacity of this agent to potentiate SHP2 inhibition. Because both *ERBB2* and *EGFR* scored separately in our screen, but we noted variability of the effects of ERBBs inhibition, we chose to evaluate lapatinib, which targets both receptors as either a homodimer or heterodimer. We exposed KE-39 to 1 μM lapatinib (a pan-ERBB inhibitor), bosutinib (an inhibitor of SRC-family kinases including Src, Lyn, and Hck), and defactinib (an FAK inhibitor) with and without 5 μM SHP099.

**Figure 3:**
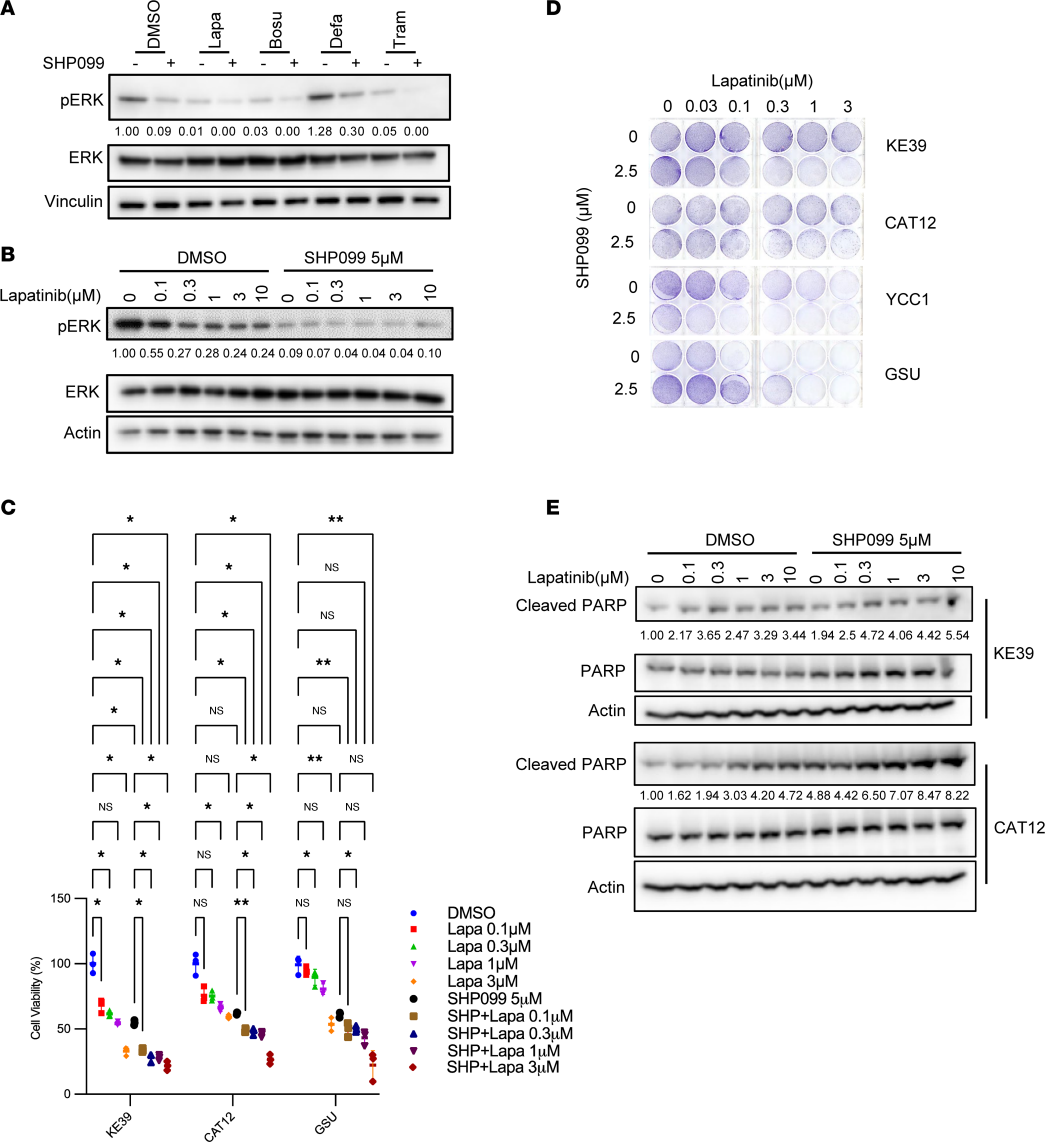
SHP2 inhibition sensitizes *KRAS*-amp GC cells to an ERBB inhibitor. (**A**) Representative immunoblots of p-ERK as a marker of MAPK pathway activity in KE-39 cells after 24-hour treatment with lapatinib (Lapa; pan-ERBBi, 1 μM), bosutinib (Bosu; SRCi, 1 μM), defactinib (Defa; FAKi, 1 μM), or trametinib (Tram; MEKi, 10 nM) with or without SHP099 (SHP2i, 5 μM). DMSO was used as a vehicle control. Vinculin was used as a loading control. (**B**) Representative immunoblots of p-ERK in KE-39 cells after 24-hour treatment with lapatinib at indicated doses with or without 5 μM SHP099. DMSO was used as a vehicle control; actin was used as a loading control. (**C**) Cell viability (*n* = 3 independent experiments) in GC lines (KE-39, CAT12, and GSU) after 72-hour treatment with lapatinib at indicated doses with or without 5 μM SHP099. Cell viabilities were normalized to the DMSO control group and are expressed as a percentage of maximum proliferation. One-way ANOVA (*P* < 0.0001 for KE-39, *P* < 0.0001 for CAT12, *P* < 0.0001 for GSU), with post hoc Tukey test. *P < 0.05; **P < 0.01. (**D**) Clonogenic assay of 3 *KRAS*-amp GC lines (KE-39, CAT12, and YCC1) and 1 mutant GC line (GSU) treated with lapatinib at indicated doses with or without 2.5 μM SHP099 after 10 days. Images are representative of 3 independent experiments. (**E**) Representative immunoblots of cleaved PARP and PARP in KE-39 and CAT12 cells after 72-hour treatment with lapatinib at indicated doses with or without 5 μM SHP099.

With SHP099, p-ERK was more substantially suppressed when combined with lapatinib or bosutinib, indicating the possibility of a combination strategy using these inhibitors with SHP2i (Figure 3A). In time-course experiments up to 24 hours, lapatinib showed the most sustainable effect on suppressing p-ERK among 3 upstream tyrosine kinase inhibitors, whereas bosutinib showed the best effect on suppressing p-AKT (Supplemental Figure 3A). By contrast, we did not see enhanced biochemical effects with defactinib with a clinically achievable concentration up to 1 μM (20).

Next, to determine the cytotoxicity of these combination treatments, we performed a cell viability assay with these inhibitors in a collection of KRAS-altered cell lines (Supplemental Figure 3B) with fixed concentration, showing that lapatinib and bosutinib in combination with SHP099 had complementary effects in *KRAS*-amp cell lines but that, again, the addition of defactinib to SHP099 had weaker effects. Similarly, defactinib did not show either suppression of MAPK pathway (Supplemental Figure 3C) or inhibitory effect of cell viability (Supplemental Figure 3D).

**Figure 4:**
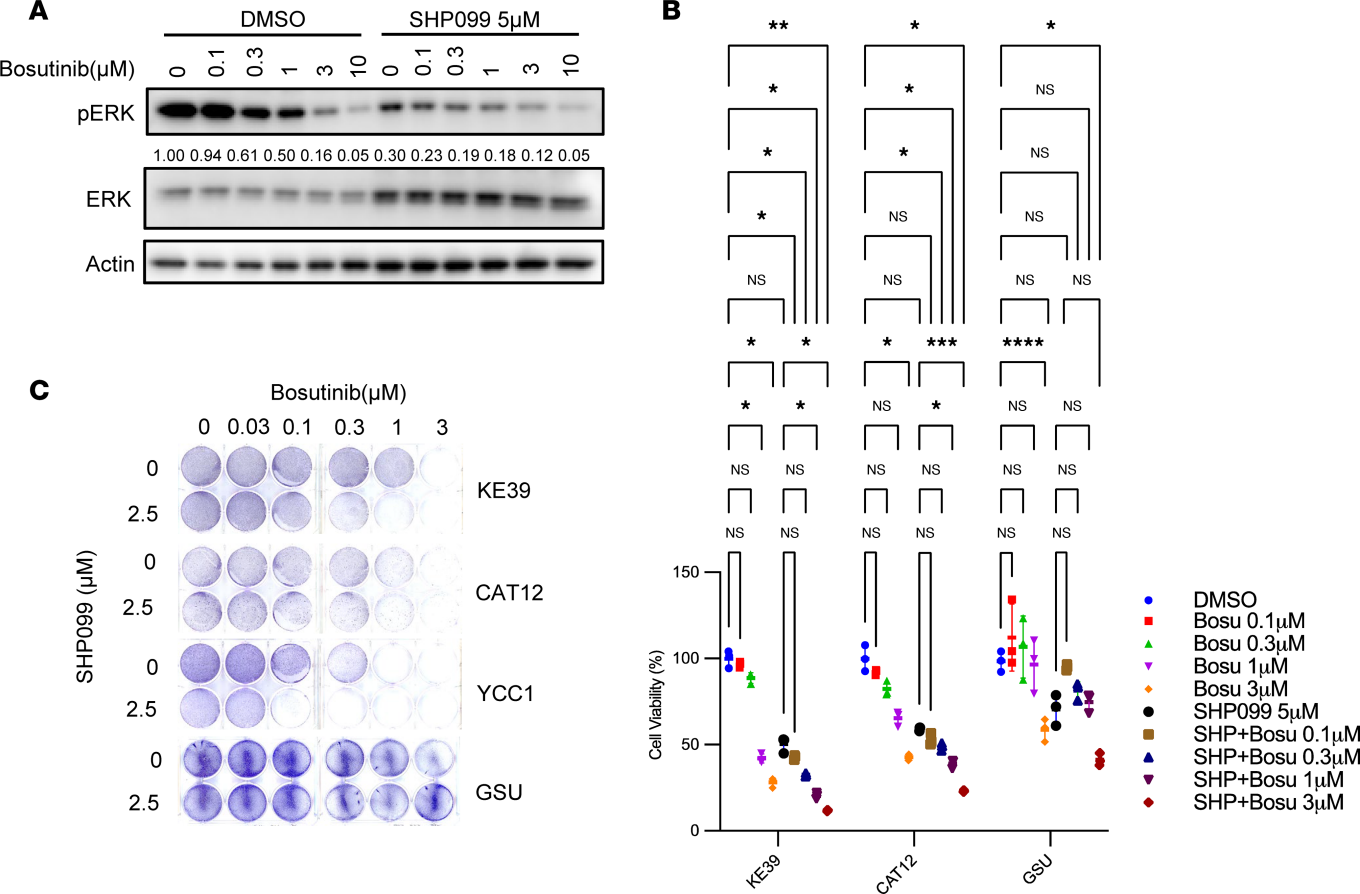
SHP2 inhibition sensitizes *KRAS*-amp GC cells to an SRC inhibitor. (**A**) Representative immunoblots of p-ERK in KE-39 cells after 24-hour treatment with bosutinib at indicated doses with or without 5 μM SHP099. DMSO was used as a vehicle control; actin was used as a loading control. (**B**) Cell viability (*n* = 3 independent experiments) in GC lines (KE-39, CAT12, and GSU) after 72-hour treatment with bosutinib (Bosu) at indicated doses with or without 5 μM SHP099 (SHP). Cell viabilities were normalized to the DMSO control group and are expressed as a percentage of maximum proliferation. One-way ANOVA (*P* < 0.0001 for KE-39, *P* < 0.0001 for CAT12, and *P* < 0.0001 for GSU), with post hoc Tukey test. *P < 0.05, **P < 0.01, ***P < 0.001, ****P < 0.0001. (**C**) Clonogenic assay of 3 *KRAS*-amp GC lines (KE-39, CAT12, and YCC1) and 1 *KRAS*-mut GC line (GSU) treated with bosutinib at indicated doses with or without 2.5 μM SHP099 after 10 days. Images are representative of 3 independent experiments.

Next, we performed additional study of the combination of SHP099 with lapatinib and bosutinib (Figure 3, B–E, and Figure 4). We first evaluated the combination of SHP2 with lapatinib and examined this combination with immunoblotting (Figure 3B) and CellTiter-Glo cell viability assays (Figure 3C). Lapatinib suppressed p-ERK well at the concentration of 0.3 to 1 μM, and the addition of SHP099 further suppressed p-ERK (Figure 3B). The cell viability assay showed the combination of lapatinib and SHP099 had stronger effects than lapatinib alone in *KRAS*-amp cell lines. Furthermore, we performed clonogenic assays to determine the cytotoxicity in longer-term experiments (10 days) and found that the combination of SHP099 with lapatinib markedly decreased growth relative to lapatinib or SHP099 alone in multiple *KRAS*-amp cell lines (Figure 3D and Supplemental Figure 4A). In contrast, we did not see the same collaborative effect in the *KRAS*-mut GSU cells (Figure 3D). Interestingly, YCC-1, which had positive LFC scores in *EGFR* and *ERBB2*, showed sensitivity to the SHP099 and lapatinib combination, probably due to the strong baseline dependency of YCC1 on EGFR (Supplemental Table 6) and the difference of individual gene KO and chemical co-inhibition of EGFR and ERBB2, which occurs with a dual kinase inhibitor.

We next used evaluated bosutinib, a dual SRC/ABL kinase inhibitor that is FDA approved for chronic myelogenous leukemia treatment (21). Bosutinib moderately suppressed p-ERK at concentrations of 100 to 300 nM (Figure 4A), and 5 μM SHP099 modestly sensitized *KRAS*-amp cells to bosutinib (Figure 4B). We further examined this combination with a clonogenic assay to determine the cytotoxicity in longer-term experiments (10 days) and found that the combination of SHP099 and various doses of bosutinib had stronger effects than bosutinib alone in *KRAS*-amp cells but not in *KRAS*-mut GSU cells (Figure 4C and Supplemental Figure 4B).

We compared the lapatinib and bosutinib results, putting greater emphasis on the dose levels where we saw efficacy relative to clinically achievable doses as determined by prior clinical studies. Although both compounds showed efficacy against *KRAS*-amp cells, lapatinib showed efficacy at a dose of 1 μM, which is below levels achieved in prior clinical studies (22). By contrast, although efficacy of bosutinib was also noted at an in vitro dose of 1 μM, the dose was higher than that used in patients in prior clinical studies (23). Additionally, we investigated whether lapatinib induced apoptosis, by checking cleavage of PARP. As shown in Figure 3E, lapatinib plus SHP099 induced PARP cleavage in both CAT12 and KE-39 cells.

**Figure 5:**
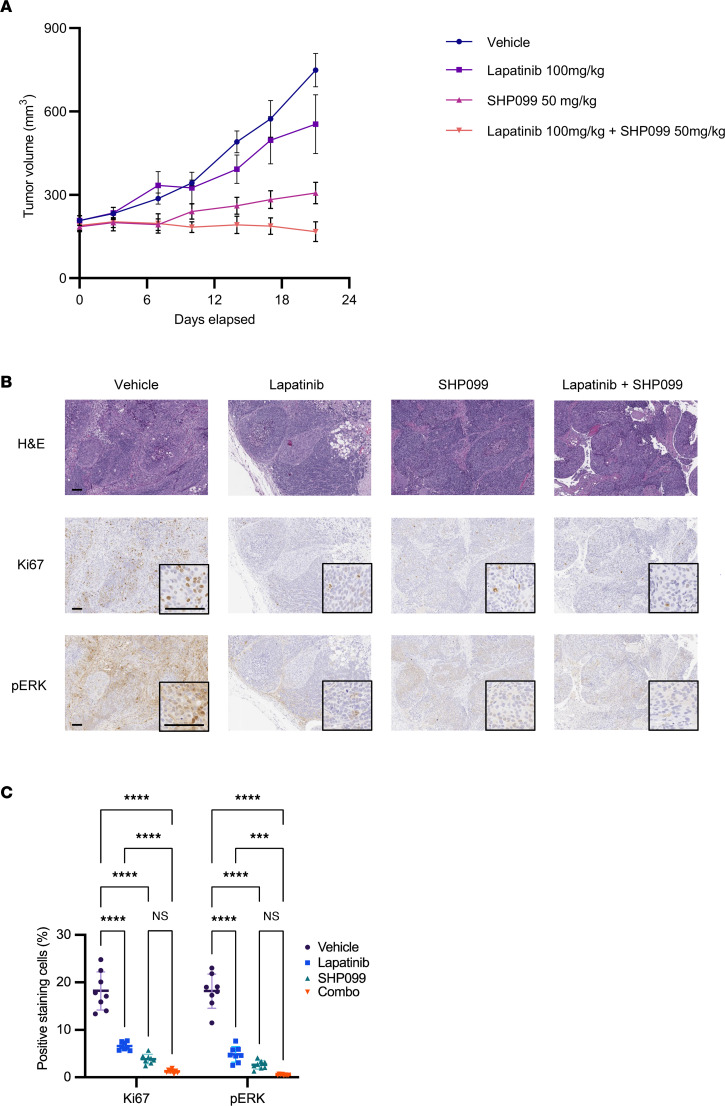
Antitumor effects of SHP099 and lapatinib combination on *KRAS*-amp cell-derived xenograft tumors. (**A**) Tumor growth of CAT12-derived xenografts. We injected 3 × 10^6^ CAT12 cells into flanks of NSG mice. Once tumors were approximately 150 mm^3^, mice were treated with vehicle, SHP099 (50 mg/kg), lapatinib (100 mg/kg), or both drugs in combination for approximately 3 weeks. Error bars represent mean ± SEM (*n* = 9 per group). Two-way ANOVA with day 21 tumor volume (*P* for interaction = 0.679; *P* < 0.0001 for SHP099; *P* < 0.018 for lapatinib). (**B** and **C**) Representative immunohistochemistry of CAT-12 tumors for indicated protein, Error bars represent mean ± SD (*n* = 8 per group). Two-way ANOVA (*P* for interaction < 0.0001 in Ki67 and *P* for interaction < 0.0001 in p-ERK) with post hoc Tukey test. ***P < 0.001, ****P < 0.0001. Scale bars: 100 μm. Combo, combination.

On the basis of these data, we chose lapatinib, a noncovalent pan-ERBB inhibitor approved by FDA as a treatment for breast cancer with HER2 expression (24, 25), as an agent to combine with SHP099 for in vivo testing. We performed studies to evaluate SHP099 and lapatinib alone and in combination in the CAT12 patient-derived cell line, selecting this model both because of its demonstrated in vivo growth and the impact of both *EGFR* and *ERBB2* genes in the genetic screens with SHP099. We chose a lapatinib dose of 100 mg/kg on the basis of a previous report of preclinical and clinical pharmacokinetic studies of lapatinib (26). The tumor growth after 3 weeks of treatment in the groups receiving lapatinib–SHP099 combination therapy was significantly suppressed in comparison with tumor growth in the control or monotherapy groups (Figure 5A). Statistically, this combination therapy showed an additive effect without interaction in 2-way ANOVA analysis (*P* for interaction = 0.6786; *P* < 0.0001 for SHP099; *P* = 0.0175 for lapatinib) (Figure 5A). Furthermore, the combination treatment with lapatinib and SHP099 resulted in a marked suppression of Ki67 and p-ERK in the xenografted tumors (Figures 5, B and C, and Supplemental Figure 5A).

No apparent ill effects were observed in these experiments. No significant weight loss was observed (Supplemental Figure 5B). These studies demonstrated the feasibility of development of MAPK inhibitor–sparing combinations of RTK and SHP2is in *KRAS*-amp GEA, with inhibitors of the ERBB family emerging as lead candidates for combination.

### CDK4/6 inhibition sensitizes KRAS-amp GEA models to SHP2 inhibition.

Next, we examined combination strategies with SHP2 and targets downstream of KRAS. The CRISPR screening data identified SHOC2 as well as CRAF and BRAF as candidate targets. However, we recognized the limitations of the CRISPR data in this area, given the presence of many paralogs (e.g., MEK1 and MEK2), wherein genetic silencing of individual genes may not phenocopy the effects of inhibitors, which can block pairs of similar targets. Therefore, we decided to more broadly evaluate targets downstream of KRAS.

**Figure 6:**
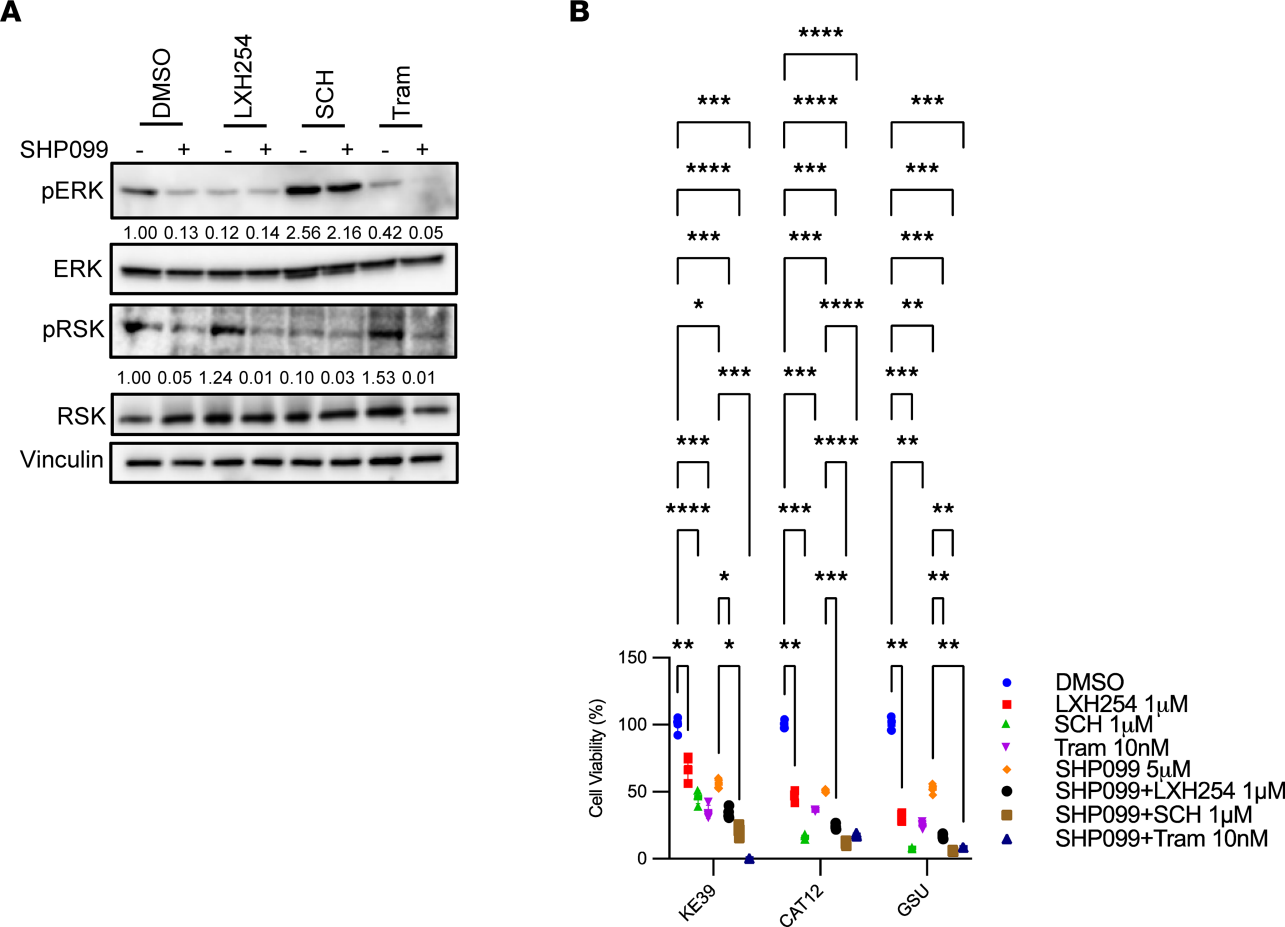
SHP2 inhibition sensitizes both *KRAS*-amp and *KRAS*-mut GC cells to MAPK inhibitors. (**A**) Representative immunoblots of p-ERK and p-RSK as markers of MAPK pathway activity in KE-39 cells after 24-hour treatment with LXH254 (RAFi, 1 μM), SCH772984 (SCH; ERKi, 1 μM), or trametinib (Tram; MEKi, 10 nM), with or without SHP099 (SHP2i, 5 μM). DMSO was used as a vehicle control; vinculin was used as a loading control. (**B**) Cell viability (*n* = 3 independent experiments) in GC lines (KE-39, CAT12, and GSU) after 72-hour treatment of indicated inhibitors used in **A**. Cell viabilities were normalized to the DMSO control group and are expressed as a percentage of maximum proliferation. One-way ANOVA (*P* < 0.0001 for KE-39, *P* < 0.0001 for CAT12, and *P* < 0.0001 for GSU), with post hoc Tukey test. *P < 0.05, **P < 0.01, ***P < 0.001, ****P < 0.0001.

Although the stated goal of this study was to identify additional targets for combination with SHP2 inhibition outside of targets in the MAPK pathway, we first validated diverse MAPK targets. We validated that inhibitors blocking multiple distinct nodes within the MAPK pathway could potentiate antiproliferative effects of SHP099 in *KRAS*-amp GEA. Specifically, we evaluated not only trametinib (a MEK1/2 inhibitor) but also LXH254 (a BRAF/CRAF inhibitor) and SCH772984 (an ERK inhibitor). Each agent could, as monotherapy, suppress downstream of MAPK pathway with the use of p-RSK as a complement to p-ERK with the evaluation of SCH772984 effects, because suppression of p-ERK did not last for 24 hours (Figure 6A). Each agent induced compensatory p-AKT induction, which could be blocked by addition of SHP2, at multiple time points (Supplemental Figure 3A), and we saw additive effects of each agent when combined with SHP2 in *KRAS*-amp GEA (Supplemental Figure 6). Next, we performed a cell viability assay to determine cytotoxicity (Figure 6B) with a fixed concentration (1 μM) and found that LXH254 and SCH772984 in combination with SHP099 had complementary effects in *KRAS*-amp cell lines, but the combination was not as efficacious as trametinib at 10 nM.

**Figure 7:**
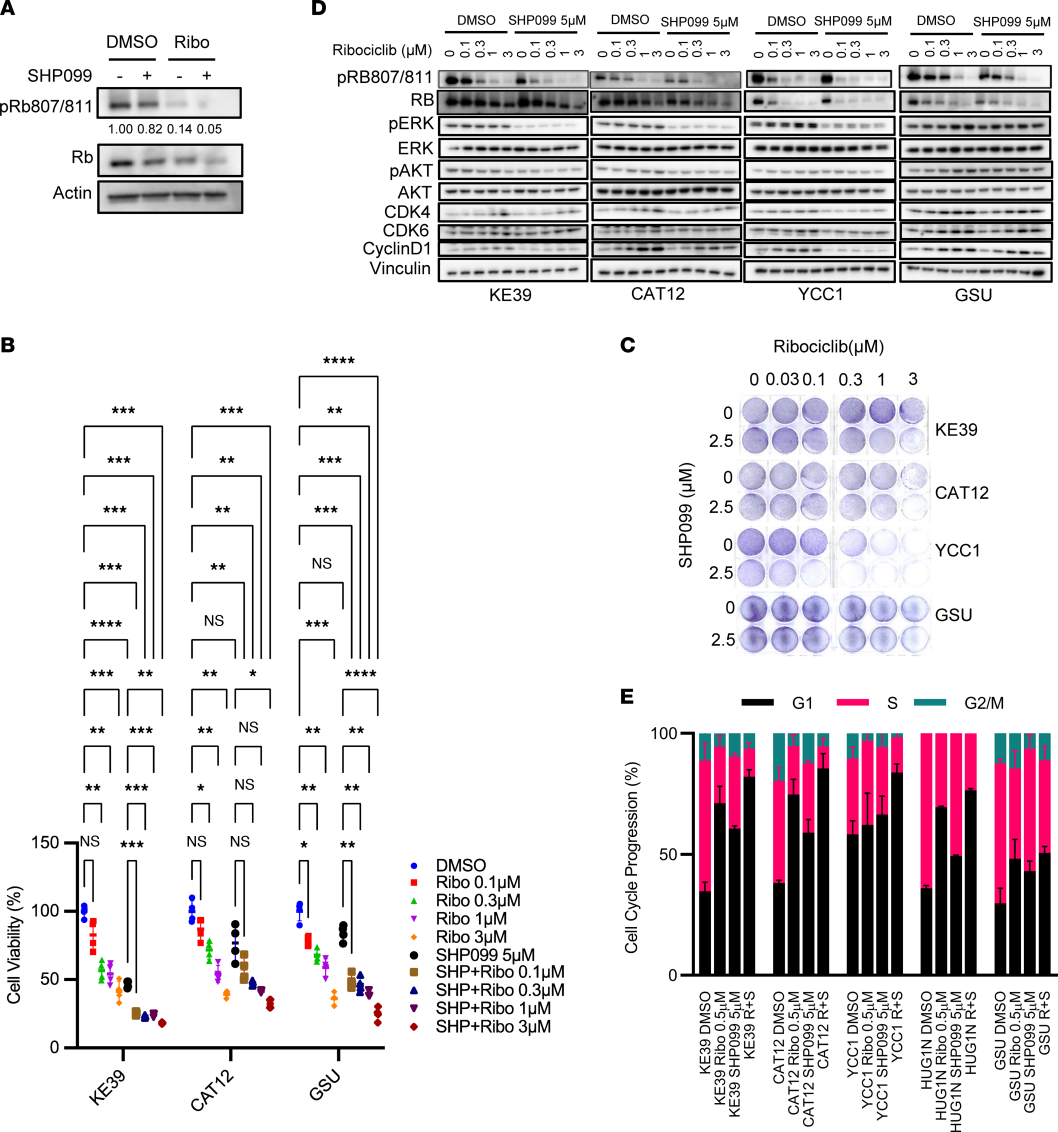
SHP2 inhibition sensitizes *KRAS*-amp GC cells to a CDK4/6 inhibitor. (**A**) Representative immunoblots of p-Rb in KE-39 cells after 24-hour treatment with 1 μM ribociclib with or without 5 μM SHP099. DMSO was used as a vehicle control; actin was used as a loading control. (**B**) Cell viability (*n* = 3 independent experiments) in GC lines (KE-39, CAT12, and GSU) after 72-hour treatment with ribociclib (Ribo) at indicated doses with or without 5 μM SHP099 (SHP). Cell viabilities were normalized to the DMSO control group and are expressed as a percentage of maximum proliferation. One-way ANOVA (*P* < 0.0001 for KE-39, *P* < 0.0001 for CAT12, and *P* < 0.0001 for GSU), with post hoc Tukey test. *P < 0.05, **P < 0.01, ***P < 0.001, ****P < 0.0001. (**C**) Clonogenic assay of 3 *KRAS*-amp GC lines (KE-39, CAT12, and YCC1) and 1 mutant GC line (GSU) treated with ribociclib at indicated doses with or without 2.5 μM SHP099 after 10 days. Images are representative of 3 independent experiments. (**D**) Representative immunoblots of p-Rb, Rb, p-ERK1/2, ERK1/2, p-AKT, AKT, CDK4, CDK6, and cyclinD1 in 3 KRAS-amp GC lines (KE-39, CAT12, and YCC1) and 1 mutant GC line (GSU) after 24-hour treatment with ribociclib at indicated doses with or without 5 μM SHP099. DMSO was used as a vehicle control; vinculin was used as a loading control. (**E**) Four *KRAS*-amp GC lines (KE-39, CAT12, YCC1, and HUG1-N) and 1 mutant GC line (GSU), treated as above, were stained with propidium iodide, then flow cytometry was performed. Error bars represent mean ± SEM. R, ribociclib; S, SHP099.

Given the strong results from our functional genomic testing pointing to the importance of blocking MAPK signaling in concert with SHP2, we next sought additional targets functionally related to mitogenic signaling. We decided to focus on inhibitors of the cell cycle pathway, specifically CDK4/6 inhibitors. Activity of CDK4/6 is directly downstream of the MAPK pathway. Furthermore, multiple FDA-approved inhibitors of CDK4/6 are already used in clinical practice, and the good clinical tolerability profiles of these drugs make them prime candidates for use in combination therapy, as exemplified by their current use in combination with hormonal therapy in estrogen receptor–positive breast cancer. Therefore, we performed studies with the CDK4/6 inhibitor ribociclib, using dephosphorylation of Rb protein as a marker of ribociclib efficacy, confirming ribociclib, at 1 μM in vitro, could effectively dephosphorylate Rb in *KRAS*-amp GEA cell lines (Figure 7A).

Next, we evaluated the effect of co-inhibition of CDK4/6 and SHP2. We chose not to evaluate the combination effect with SHP099 and ribociclib with the Cell Titer-Glo assay, as used with combination of SHP2 and RTK inhibitors, because inhibition of CDK4 has been reported to increase the ATP level per cell (27). We instead used the Realtime-Glo MT assay, which measures reducing potential per cell. We found that the combination of ribociclib and SHP099 had potent cytotoxicity against all *KRAS*-amp cells relative to single-agent ribociclib, which had various effects on these cell lines (Figure 7B). Furthermore, using a 10-day clonogenic assay, we observed longer-term cytotoxicity of the SHP099–ribociclib combination in *KRAS*-amp KE-39, CAT12, and YCC1 cells but more modest effects of the combination in the GSU *KRAS*-mut cells (Figure 7C and Supplemental Figure 7B). In addition, biochemical data demonstrated the combination of SHP2 and ribociclib enhanced dephosphorylation of pRB, which inhibits cell cycles (Figure 7D and Supplemental Figure 7A). Cell cycle analysis also demonstrated cooperative enhancement of G1 arrest in *KRAS*-amp GEA, effects not similarly seen in the *KRAS*-mut GSU GEA model (Figure 7E).

Notably, in contrast to inhibition of nodes within the MAPK pathway, treatment of *KRAS*-amp GEA models with CDK4/6 inhibition did not induce compensatory p-AKT induction in *KRAS*-amp cells (Figure 7D and Supplemental Figure 7A), consistent with their activity to inhibit cell proliferative signaling below the level of the MAPK pathway, thus not inducing the same feedback reactivation as with the MAPK-directed agents. Furthermore, the combination of SHP099 and ribociclib did not consistently induce cleaved PARP, indicating the mechanism of action of this combination is independent of apoptosis (Supplemental Figure 7C).

**Figure 8:**
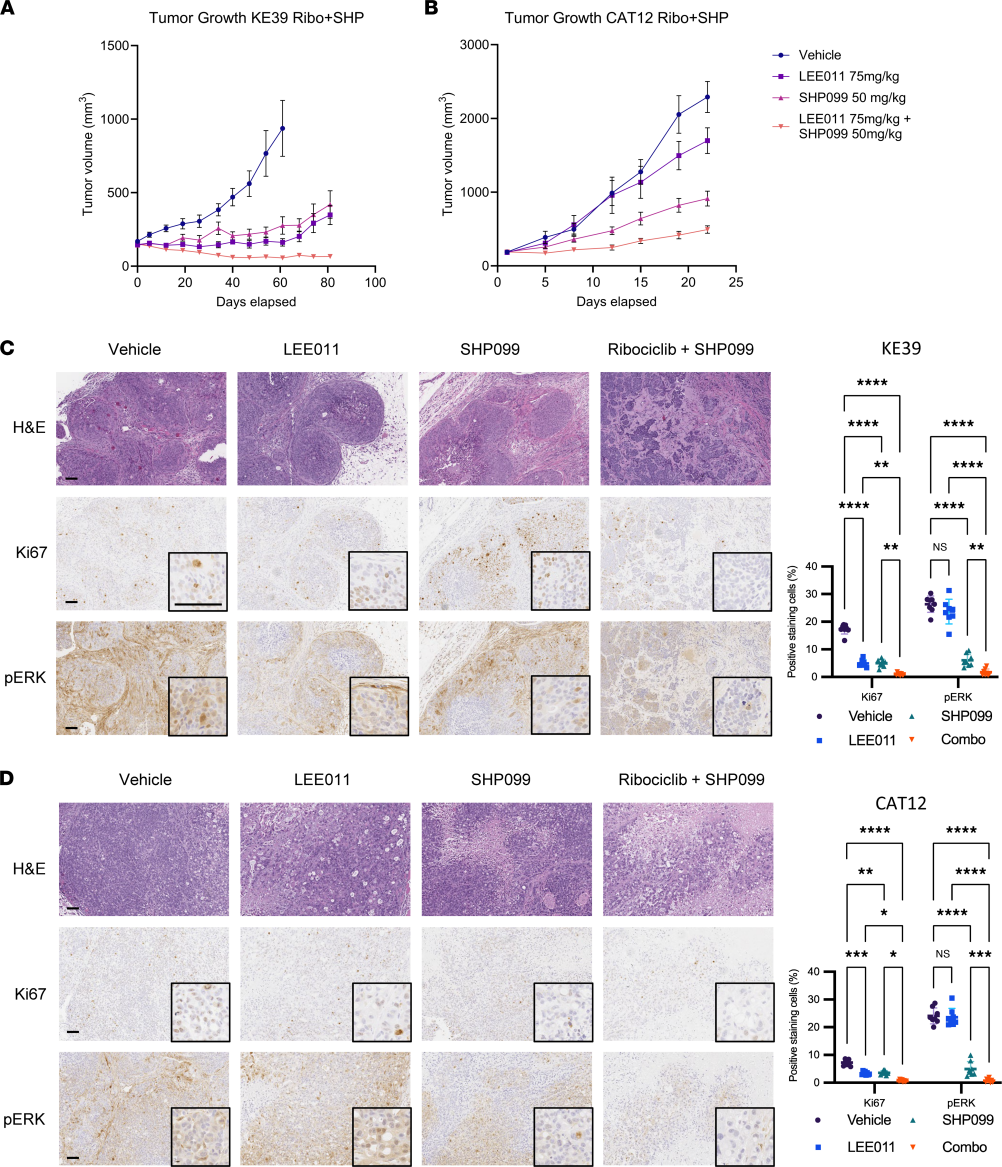
Antitumor effects of SHP099 (SHP) and ribociclib (Ribo) combination on *KRAS*-amp cell-derived xenograft tumors. (**A**) Tumor growth of KE-39–derived xenografts. We injected 5 × 10^6^ KE-39 cells into flanks of NSG mice. Once tumors were approximately 150 mm^3^, mice were treated with vehicle, SHP099 (50 mg/kg), LEE011 (75 mg/kg), or both drugs in combination for approximately 12 weeks. Error bars represent mean ± SEM (*n* ≥ 8 per group). Two-way ANOVA with day 61 tumor volume (*P* for interaction < 0.01, *P* < 0.001 for SHP099, and *P* < 0.0001 for LEE011). (**B**) Tumor growth of CAT12-derived xenografts. We injected 3 × 10^6^ CAT12 cells into flanks of NSG mice. Once tumors were approximately 150 mm^3^, mice were treated with vehicle, SHP099 (50 mg/kg), LEE011 (75 mg/kg), or both drugs in combination for approximately 3 weeks. Error bars represent mean ± SEM (*n* ≥ 8 per group). Two-way ANOVA with day 22 tumor volume (*P* for interaction = 0.57, *P* < 0.0001 for SHP099, and *P* < 0.01 for LEE011). (**C** and **D**) Representative immunohistochemistry of KE-39 (**C**) and CAT12 (**D**) tumors for indicated protein. Error bars represent mean ± SD (*n* = 8 per group). Statistical comparisons between each group were made using 2-way ANOVA for positive stained cells. *P < 0.05, **P < 0.01, ***P < 0.001, ****P < 0.0001.

Given these promising in vitro data with this combination, we also tested the combination treatment with SHP099 and ribociclib in vivo in a xenograft model of KE-39 and CAT12. The ribociclib dose was determined in previous preclinical and phase I trials to be 75 mg/kg (28, 29). With KE-39, the ribociclib–SHP099 combination resulted in continuous regression of tumor size, whereas single-compound treatment resulted in slow but progressive tumor growth (Figure 8A). With CAT12, the ribociclib–SHP099 combination resulted in better suppression of tumor growth than did either ribociclib or SHP099 alone or in the vehicle control arms (Figure 8B). Although SHP2 inhibition had single-agent activity, this combination therapy showed either an additive effect (*P* for interaction = 0.5702) (Figure 8B) or modest synergistic effect (*P* for interaction < 0.01; percentage of total variation, 10.44) (Figure 8A), depending on cell lines used for the xenografts. Furthermore, histological analysis confirmed combination therapy reduced Ki67 staining as well as p-ERK and pRb expression in both KE-39 and CAT12 xenografts (Figure 8, C and D).

No apparent ill effects were observed in these experiments. No significant weight loss was observed (Supplemental Figure 8, A and B). Overall, these data suggest the combination of CDK4/6 and SHP2is is a promising treatment strategy against *KRAS*-amp GEA.

## Discussion

Amplification of *KRAS*, without activating missense mutations, has been described in a variety of cancers but most commonly within the CIN-type GEAs. The oncogene *KRAS* has been heavily studied as a frequently mutated gene in various cancers, including colorectal, pancreatic, and lung cancers. By contrast, there has been minimal study of the effects of marked overexpression of the WT KRAS protein that occurs with genomic amplification. Our group previously reported (7) that *KRAS* amplification in GEA leads to a cell with a highly adaptive state, with marked expression of WT KRAS protein that retains physiologic capacity to toggle between GTP (active) and GDP (inactive) status. Beyond the substantial overexpression of KRAS, this plasticity is distinct from that of typical *KRAS*-mut cells such as *KRAS*^G12D^, which leads to defective GAP-mediated GTP hydrolysis. In our prior study (7), we demonstrated that with enzymatic inhibition of the MAPK pathway leading to reduced negative feedback, *KRAS*-amp cells can rapidly mobilize robust activation of KRAS-GTP, thus promoting cell survival and proliferation — a barrier to the development of MAPK pathway-targeted therapies against *KRAS*-amp GEA.

We originally demonstrated that pharmacologic SHP2 inhibition or genetic inactivation of SOS1, which both act in physiologic RAS activation, could inhibit adaptive WT KRAS after reduction of a negative feedback loop after MEK inhibition. Beyond its ability to attenuate adaptation, we also found that monotherapy with the SHP2i SHP099 could attenuate basal MAPK activity, as measured by p-ERK, and showed some antiproliferative effects both in vitro and in vivo in *KRAS*-amp GEA models. On the basis of these results and the fundamental baseline inferred biology of tumors with *KRAS* amplification, we explored broader combinatorial possibilities for *KRAS*-amp GEA using SHP2is as a backbone for such therapies.

We carried out a comprehensive genome-wide screen with the CRISPR/Cas9 library to systematically identify secondary targets that complement SHP2 inhibition across *KRAS*-amp GEA models. Our results highlight the importance of targeting RTK/RAS/MAPK signaling in *KRAS*-amp cells even with SHP2 inhibition. We then separately investigated targets both upstream and downstream of KRAS. First, we demonstrated efficacy of SHP2 inhibition in combination with the pan-ERBB inhibitor lapatinib. After comparing multiple combinations of RTK inhibitors with SHP099, our data suggest that the multitargeted ERBB agents such as lapatinib are promising agents to be combined SHP2is for subsequent clinical trials.

Our results show some parallels to efforts to establish combinations for *BRAF*-mutated colorectal cancer tumors, which are less sensitive to BRAF inhibitor monotherapy (30, 31) than are melanomas. The combined therapy with EGFR, BRAF, and MEK inhibition has shown efficacy in *BRAF*-mutated colorectal cancer, demonstrating the clinical value of using ERBB inhibition to enhance targeting of an MAPK oncogene (32). However, unlike the case of *BRAF*-mutated colorectal cancer, our combination of lapatinib and SHP099 integrated 2 different targets both upstream of KRAS and did not require MEK- or RAF-directed agents. Of note, ongoing studies are investigating the potential to combine SHP2 inhibition with EGFR signaling, although these are being done in an attempt to enhance efficacy of EGFR therapies in tumors with oncogenic EGFR mutations. Specifically, a phase Ib/II trial investigating the SHP2i RMC-4630 in combination with an EGFR inhibitor is currently underway in non–small cell lung cancer (ClinicalTrials.gov NCT03989115).

Beyond our analysis of the targeting of signaling nodes upstream of KRAS, our finding that SHOC2 is the strongest node that augments SHP2 inhibition indicates the clear importance of targeting MAPK activation downstream of RAS in these tumors, similar to the strength of SHOC2 as a target to complement MEK inhibition in *KRAS*-mut cancers (33). Indeed, our results demonstrated the clear potential efficacy in not just targeting MEK, as in our prior report (7), but also RAF or ERK as an adjunct to SHP2 inhibition. Additional work will be needed to carefully contrast the relative value of targeting these distinct nodes in concert with SHP2 inhibition in *KRAS*-amp GEA. As with development of any combination with MAPK agents in RAS-driven cancers, the potential value indicated by laboratory studies must be balanced, ultimately, against the question of the clinical tolerability of distinct combinations. Indeed, each of the FDA-approved MEKis selumetinib (34), trametinib (35), binimetinib (36), and cobimetinib (37) are associated with side effects, including gastrointestinal and skin toxicity.

Although these historic challenges with development of MAPK-based combinations for treatment of RAS-mutant cancers do not negate the importance of investigating SHP2–MAPK combinations in the clinic, they did motivate the focus of this study to query for additional potential targets that may effectively combine with SHP2 in the *KRAS*-amp state. Given this goal and the strong data supporting inhibition of multiple nodes within the MAPK in concert with SHP2 inhibition, we chose to evaluate targets immediately downstream of the MAPK pathway, specifically the activity of cyclin D, a key downstream effector of MAPK signaling (38). Notably, the CDK4/6 inhibitors palbociclib, ribociclib, and abemaciclib, which inhibit kinases that partner with cyclin D, are already FDA approved for use in metastatic breast cancer and possess favorable tolerability profiles. Our evaluation of CDK4/6 inhibition in combination with SHP2 inhibition demonstrated potent antitumor effects of SHP2–CDK4/6 dual inhibition. A recent study reported the efficacy of CDK4/6–SHP2 co-inhibition in *KRAS*-mut cancers, which is consistent with our *KRAS*-amp GEA results (39). Together, these results suggest that the combination of SHP2 inhibition and inhibition of downstream MAPK pathways, such as cell cycle regulators, is a promising strategy for treatment of KRAS-driven cancers regardless of KRAS genotype. CDK4/6 inhibitors have been also evaluated in concert with RAF (40) and MEK (41) inhibitors of *BRAF* and *RAS* mutant tumors, respectively*,* demonstrating preclinical efficacy.

There is already a host of trials evaluating SHP2is in distinct combinations for cancer, including a newer, more potent derivative of SHP099, TNO155 (42), and RMC-4630. Trials are being conducted for TNO155 with a ribociclib combination in *KRAS*-WT non–small cell lung cancer and *KRAS*-mut colorectal cancer (ClinicalTrials.gov NCT04000529). Furthermore, a phase I/II trial of adagrasib (MRTX849) in combination with TNO155 in patients with advanced solid tumors with *KRAS*^G12C^ mutation is ongoing (KRYSTAL-2; ClinicalTrials.gov NCT04330664). Besides TNO155, another SHP2i, RMC-4630, is in clinical trials with cobimetinib (an MEKi) in relapsed or refractory solid tumors, and with osimertinib in EGFR^+^, locally advanced, or metastatic non–small cell lung cancer (ClinicalTrials.gov NCT03989115). Given the ability of SHP2 inhibition to inhibit signaling into the MAPK pathway, which can both attenuate basal signaling into the MAPK pathway and abrogate cellular adaptations in response to targeted inhibitors, SHP2 has emerged as a highly promising target for combinatorial therapies in tumors with RAS and/or RTK driving alterations.

There are several limitations to this study. First, we could not clarify whether the effects of lapatinib are mediated by inhibition of EGFR kinase activity, HER2 kinase activity, or both. However, genomics of GEAs shows that both ERBB2 and EGFR are subject to amplification in GEA. Moreover, ErbB receptors, including EGFR and HER2, form both homo- and heterodimers, providing a high degree of downstream signaling diversity (43) and the potential for a pan-ErbB inhibitor to have broader potential efficacy and be simpler to deploy in the clinic in combination with SHP2 agents. Second, we have not compared SHP2–CDK4/6 co-inhibition and SHP2–pan-ERBB co-inhibition directly. Ideally, both strategies would be advanced in the clinic to evaluate efficacy and tolerability as well as the potential biomarkers that may guide optimal strategies for given patients.

This study highlights that tumors with amplification of WT KRAS represent an especially promising area for development of SHP2 inhibition. Furthermore, we demonstrated the efficacy of SHP2–CDK4/6 co-inhibition and SHP2–pan-ERBB co-inhibition as highly promising candidate strategies against *KRAS*-amp GEA tumors, highlighting the potential to develop effective SHP2-based combinations in these tumors without the need to include inhibitors of the MAPK pathway. This could directly lead to additional trials of effective yet clinically tolerable combinations for this deadly group of cancers.

## Methods

### Cell lines.

KE-39, HUG1-N, YCC1, and GSU cell lines were provided by the Dana-Farber Cancer Institute Belfer Institute, which had obtained them directly from commercial sources and authenticated them using standard short tandem repeat analysis. CAT12, a patient-derived xenograft, was generated at the University of Chicago, after obtaining written patient consent from a 72-year-old male patient with a poorly differentiated cT3N3M1 esophagogastric adenocarcinoma. KE-39, CAT12, HUG1-N, and GSU cells were grown in RPMI 1640 medium supplemented with 10% FBS. YCC1 cells were grown in DMEM and supplemented with 10% FBS. All cell lines were supplemented with 1 mM penicillin/streptomycin and 2 mM l-glutamine, and maintained at 37°C in a 5% CO_2_ incubator. All cells were routinely tested for *Mycoplasma* and found to be free of contamination.

### Small molecular inhibitors.

SHP099, LXH254, and ribociclib (LEE011) were provided by the Novartis Institutes of Biomedical Research. Lapatinib (catalog L-4804) was purchased from LC Laboratories. Bosutinib, defactinib, SCH772984, and trametinib were purchased from Selleck Chemicals. All inhibitors were dissolved in DMSO before use.

### Genome-wide CRISPR screen and data analysis.

Pooled lentiviruses for the genome-wide sgRNA library (Brunello CRISPR-KO pooled library containing 77,441 sgRNA targeting ~19,000 genes; ref. 19) was supplied by the Broad Institute Genomic Perturbation Platform after preparation from HEK293T cells in T175 flasks, as previously described (44). Briefly, the viral titer and volume for infection in each cell line were predetermined with pilot experiments prior to screening to ensure an approximately 30% infection rate in the screening experiment to minimize the potential for multiple constructs transduced into the same cell. Optimized surface area for cell growth was predetermined in pilot experiments to avoid reaching overconfluence. Puromycin concentration was predetermined with pilot experiments before screening. For the screening experiment, 150 million HUG1-N and KE-39 cells were infected with a lentiviral library in the human genome to ensure each sgRNA was represented by at least 500 cells, on average. For the infection, cells were mixed with the calculated lentivirus volume, supplemented with 8 μg/mL polybrene, and centrifuged at 936*g* for 2 hours in 12-well plates with 3 million cells per well. Fresh medium was added at a 1:1 ratio after centrifugation, and cells were transferred to flasks the next day. Infected cells were selected with 2 μg/mL puromycin from days 4 to 8, expanded for another 4 days to reach the desired cell number, and split into parallel cultures with DMSO or 5 μM SHP099 treatment. At least 40 million cells were used for each treatment condition to keep the minimum presentation number for each sgRNA at greater than 500 cells. Cells were exposed to drug treatment for 2 weeks (HUG1-N) or 3 weeks (KE-39), and genomic DNA was collected using pH 7.9 Phenol/Chloroform/IAA (25:24:1; catalog AM9730; Ambion Inc.) according to the manufacturer’s protocols and quantified using a NanoDrop 2000 spectrophotometer (Thermo Fisher Scientific). PCR and sequencing were performed as described previously (19) on a HiSeq2000 sequencer (Illumina). For analysis, the read counts were normalized to reads per million and then log_2_ transformed. The LFC of each sgRNA of the SHP099-treated group was determined relative to that of the DMSO-treated group. Hypergeometric distribution was used to determine statistical significance (https://portals.broadinstitute.org/gpp/public/analysis-tools/crispr-gene-scoring). *P* values per gene were calculated as the average –log_10_(*P* value) of individual sgRNAs. The average LFC per gene also was calculated to assess the magnitude of effect.

### Secondary, focused CRISPR library synthesis.

A custom library was designed against 509 genes from RAS pathway 2.0 and top hits in the primary screen (Supplemental Table 2), including 15 common essential genes (45). Top-scoring genes were determined on the basis of the genome-wide screens and the LFC data of HUG1-N and KE-39 cell lines. The top 100 genes from each cell line and the top 100 genes from the average LFC data of 2 data sets of HUG1-N and KE-39 cell lines were used as a gene set of interest, and RAS pathway 2.0 genes (*n* = 227) and an additional 46 genes, given druggability and biologic interest, were integrated into the library, which comprised 509 genes in total (Supplemental Table 2). Eight sgRNAs were designed against each of these genes, and 400 intergenic controls (10%) were added. In total, 4,472 sgRNAs (Supplemental Table 3) were included in the library and cloned in pXPR_023 vector.

### Immunoblot reagents.

Immunoblot analysis was performed as previously described (7). Primary Abs against p-ERK1/2 T202/Y204 (catalog 4370), total ERK1/2 (catalog 4695), p-AKT S473 (catalog 4060), total AKT (catalog 9272), p-Rb S807/811 (catalog 8516), total Rb (catalog 9309), p-p90RSK T359/S363 (catalog 9344), RSK1 (catalog 8408), p-HER2/ErbB2 Y1221/1222 (catalog 2243), HER2/ErbB2 (catalog 2165), p-EGFR Y1068 (catalog 3777), cyclin D1 (catalog 2922), CDK4 (catalog 12790), CDK6 (catalog 13331), vinculin (catalog 18799), and β-actin (catalog 12620) were purchased from Cell Signaling Technology. Pierce HRP-conjugated secondary Abs (anti-rabbit and anti-mouse) were purchased from Thermo Fisher Scientific. Amersham ECL Prime chemiluminescent detection reagent (GE Healthcare Life Sciences) was used to visualize protein expression.

### Cell viability assay.

We plated 2,000 cells in 96-well plates in triplicate and exposed them to DMSO or indicated inhibitors for 72 hours. Cell viability was quantified using the CellTiter-Glo Luminescent Cell Viability assay (catalog G7573; Promega) or RealTime-Glo MT Cell Viability Assay (catalog G9712; Promega) according to manufacturer’s instructions.

### Colony formation assay.

We plated 1 × 10^5^ to 3 × 10^5^ cells in 6-well plates. Cells were exposed to DMSO or inhibitors with medium, and drugs were replaced every 3 days. After 7 to 10 days, cells were fixed in 1% paraformaldehyde for 15 minutes at room temperature, then were washed with PBS and stained with 0.1% crystal violet solution for 15 minutes at room temperature. After staining, plates were air dried and scanned to image.

### Cell cycle assay.

A cell cycle assay was performed as previously described (46). Briefly, 1 × 10^5^ cells were plated in a 6-well plate. After overnight incubation, cells were exposed to DMSO or indicated inhibitors for 24 hours and then fixed with 70% ethanol at 4°C for 30 minutes. Fixed cells were washed with PBS containing 1% FBS and then were stained with PI/RNase Staining Solution (catalog 4087; Cell Signaling Technology) for 30 minutes at room temperature. DNA content was measured by LSR II flow cytometer and analyzed by using ModFIT LT software.

### G-LISA assay.

The RAS G-LISA assay was performed using the RAS G-LISA activation kit (catalog BK131; Cytoskeleton) according to the manufacturer’s instructions. Protein (20 μg) from whole-cell lysates was added in triplicate in a 96-well plate, and activated RAS was bound to a RAS–GTP binding protein linked to each well. Bound, active RAS was detected with an RAS-specific Ab and quantified by measuring the relative absorbance at 490 nm with a Tecan Infinite 200Pro plate reader.

### Mouse xenograft studies.

KE-39 and CAT12 cells were prepared in a 1:1 ratio of Matrigel to medium and 3 × 10^6^ CAT12 and 5 × 10^6^ KE-39 cells were prepared in a 1:1 ratio of Matrigel to medium (Matrigel; Corning) and injected subcutaneously into the flanks of NOD.Cg-Prkdc^scid^ IL2rg^tm1Wjl^/SzJ female mice (6–8 weeks old) purchased from Jackson Laboratory. Treatment was initiated when tumors reached 100–150 mm^3^. Electronic calipers were used for tumor measurements twice a week and tumor volumes were calculated using the following formula: volume = length × width^2^ × 0.5. Mice were sacrificed when tumors reached the endpoint. Dissected tumors were snap-frozen and stored at –80°C or were fixed in 10% buffered formalin for histopathologic process.

### IHC and Abs.

Fixed xenograft tumors were embedded in paraffin (FFPE). Unstained sections were stained with the following Abs purchased from Cell Signaling Technology: p-ERK1/2 T202/Y204 (catalog 4370) and p-Rb S807/811 (catalog 8516). The staining kit for Ki67 (catalog VP-K451; Vector Laboratories) was used per manufacturer’s instructions. Representative images were taken using a DM1000 LED light microscope camera (Leica Camera).

### Statistics.

Statistical analyses were performed using Prism, version 9.1 (GraphPad Software). Data are presented as the mean ± SD unless otherwise noted. For in vitro experiments, data were analyzed by the 2-tailed Student’s *t* test between 2 groups or 1-way ANOVA among 3 or more groups, unless otherwise indicated. For xenograft experiments, statistical comparisons were performed using 2-way ANOVA followed by Tukey’s multiple comparison, unless otherwise indicated. All animals were randomized to treatment group before drug administration. Animals were excluded from analysis if they were sacrificed because of health reasons unrelated to the tumor volume endpoint. *P* values are denoted in figures by asterisks (*P < 0.05, **P < 0.01, ***P < 0.001, ****P < 0.0001).

### Study approval.

All animal experiments were conducted in accordance with IACUC-approved animal protocols (no. 11-009) at Dana-Farber Cancer Institute in compliance with NIH guidelines.

## Author contributions

TL, OK and AJB designed experiments and wrote the manuscript. TL, OK, JZ, YW, KB, BP, and YZ performed experiments. TL, OK, PG, AK, JGD, and ZVH analyzed data. DVTC provided imperative advice in designing experiments and provided critical insight. DVTC provided the CAT12 patient-derived cells. AJB conducted scientific direction. The authorship order between co–first authors was determined by flipping a coin. All authors approved the manuscript.

## Supplementary Material

Supplemental data

Supplemental table 1

Supplemental table 2

Supplemental table 3

Supplemental table 4

Supplemental table 5

## Version 1 02/08/2023

Electronic publication
