# Supplementary material for: Developing SHP2-based combination therapy for KRAS-amplified cancer
Source: JCI Insight. 2023 Feb 8;8(3):e152714. doi: 10.1172/jci.insight.152714 (PMC9977440; doi:10.1172/jci.insight.152714)
Supplement: Supplemental data [file jciinsight-8-152714-s170.pdf]

Suppl Fig1

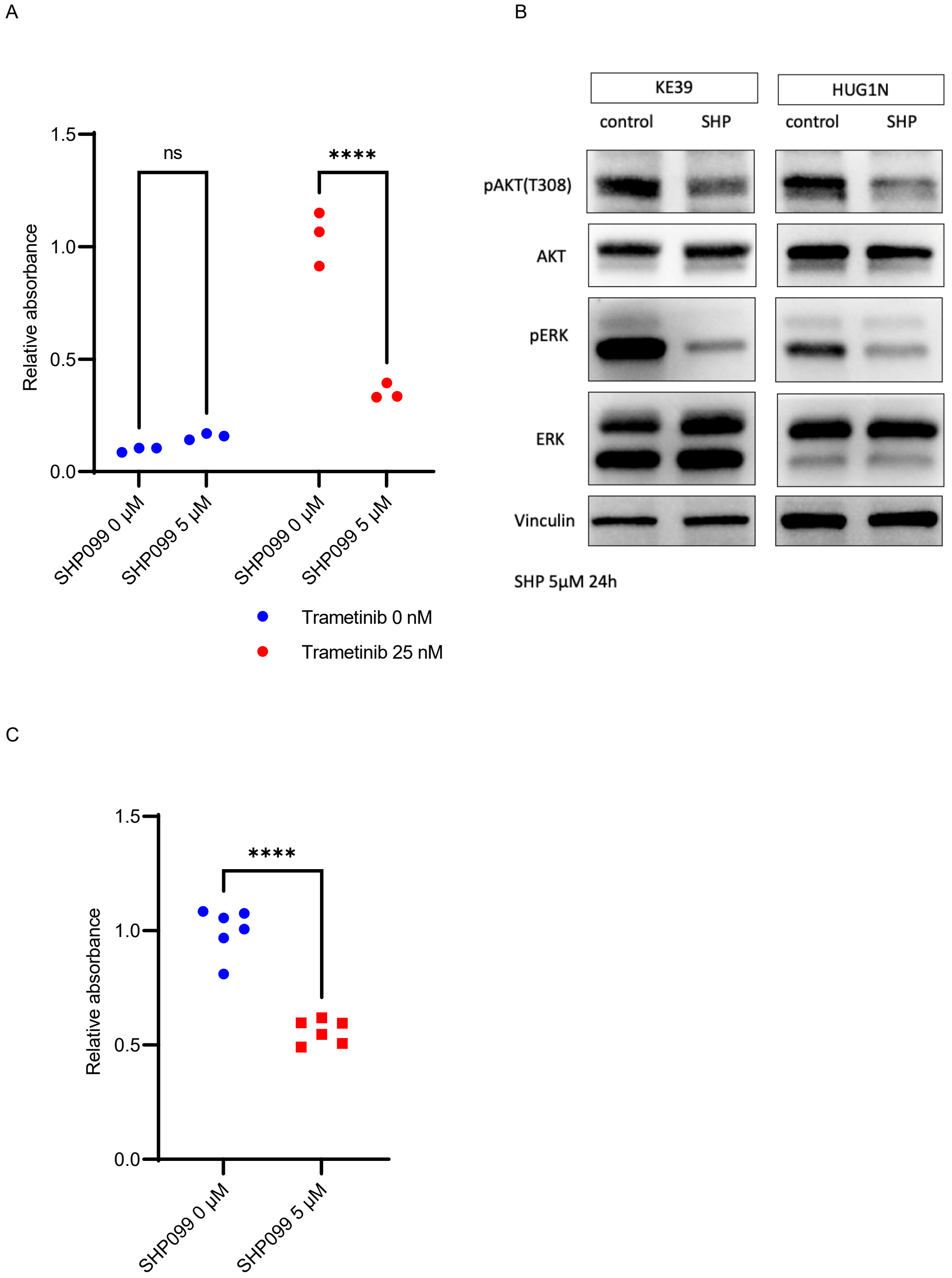

Suppl. Figure S1. Determining concentration of SHP099 for genome-wide CRISPR screening.
(A) Measurement of suppression of KRAS activity determined by GTP-bound form of KRAS by G-LISA assay in KE-39 cells after 24h treatment of SHP099 and or trametinib with indicated concentration (****P<0.0001, one way ANOVA with post-hoc Tukey multiple comparison test). (B) Immunoblots of phospho-ERK and phospho-AKT as a marker of MAPK pathway and PI3K-AKT pathway activities respectively in KE-39 cells after 24h treatment of SHP099 (5 μM). DMSO is used as a vehicle control. Vinculin was used as a loading control. (C) Cell-titer Glo cell viability assay (n=6) in KE39 after 72 h treatment of SHP099 5 μM or DMSO control. (****P<0.0001, t-test)

Suppl Fig2

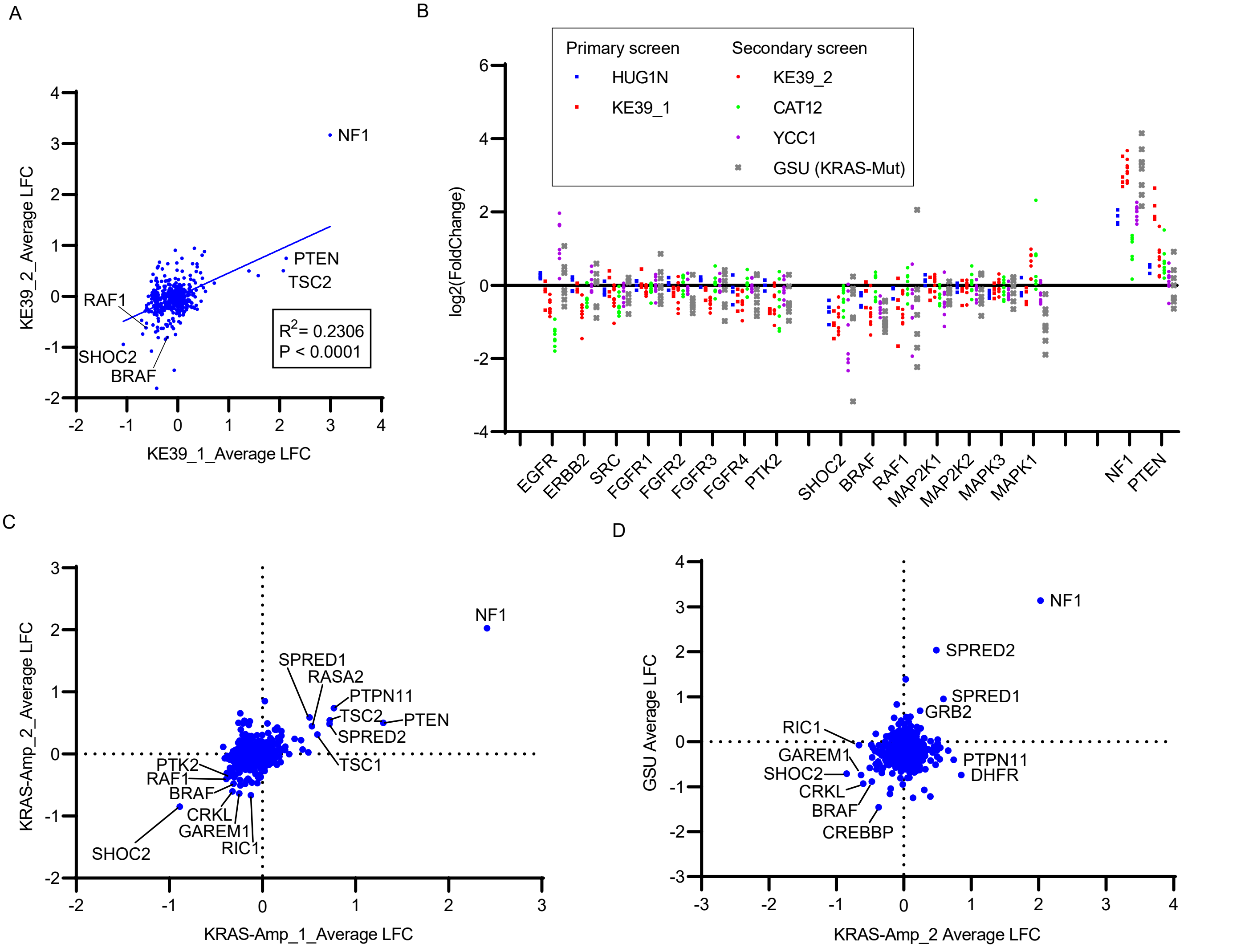

Suppl. Figure S2. Supplementary data from primary and secondary CRISPR screening.
(A) Comparison of primary and secondary screening using KE-39 dataset. (B) Log fold changes of individual sgRNA for key genes of] RTK and MAPK pathway within the indicated cell lines. Data from primary genome-wide screen (4 sgRNAs per gene) are shown in squares, and data from secondary custom screen are shown in circles. (C) X-Y plot of the average log fold changes from primary (X-axis) and secondary (Y-axis) screen. (D) Comparison of log fold change (LFC) of KRAS-amplified cell lines (X-axis) and KRAS-mutant GSU cell (Y-axis).

Suppl Fig3

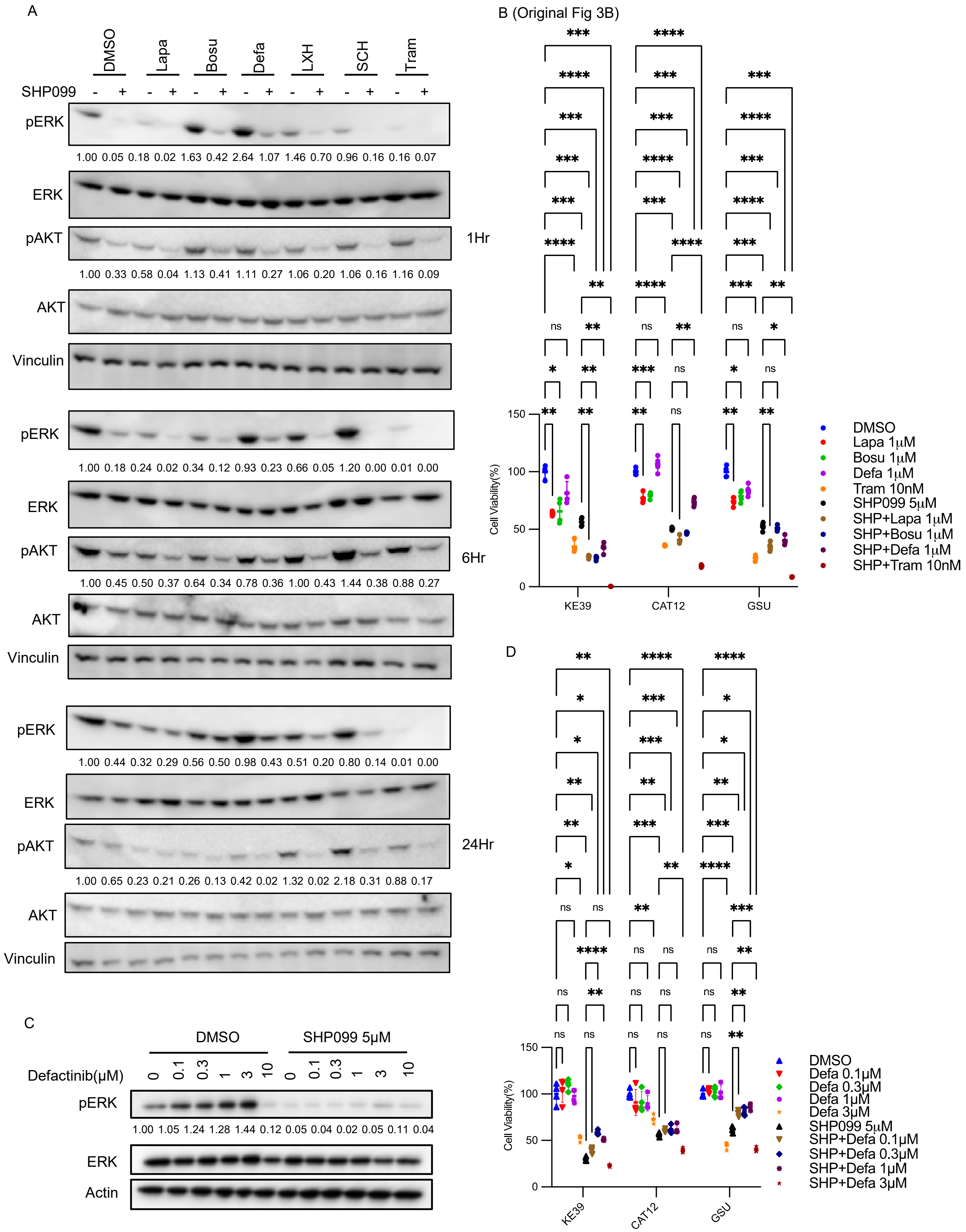

Suppl. Figure S3. **(A)** Representative Immunoblots ofphospho-ERK, ERK, phosphor-AKT and AKT as markers of MAPK pathway activity in KE-39 cells after 1h, 6h and 24h treatment of Lapatinib, Bosutinib, Defactinib, LXH254, SCH772984 and Trametinib at indicated doses with or without SHP099(5μM). DMSO is used as a vehicle control. Vinculin was used as a loading control. (B) Cell viability (n = 3 independent experiments) in GC lines (KE39, CAT12 and GSU) after 72 h treatment of indicated inhibitors used in (A). Cell viabilities are shown as mean ± s.e.m. normalized to the DMSO control group and are expressed as a percentage of maximum proliferation. (C) Representative Immunoblots of pERK as a marker of MAPK pathway activity in KE-39 cells after 24h treatment of Defactinib (FAKi, 1 μM) with or without SHP099 (SHP2i, 5 μM). DMSO is used as a vehicle control. Actin was used as a loading control. (D) Cell viability (n = 3 independent experiments) in GC lines (KE39, CAT12 and GSU) after 72 h treatment of indicated inhibitors used in (C).

Suppl Fig4

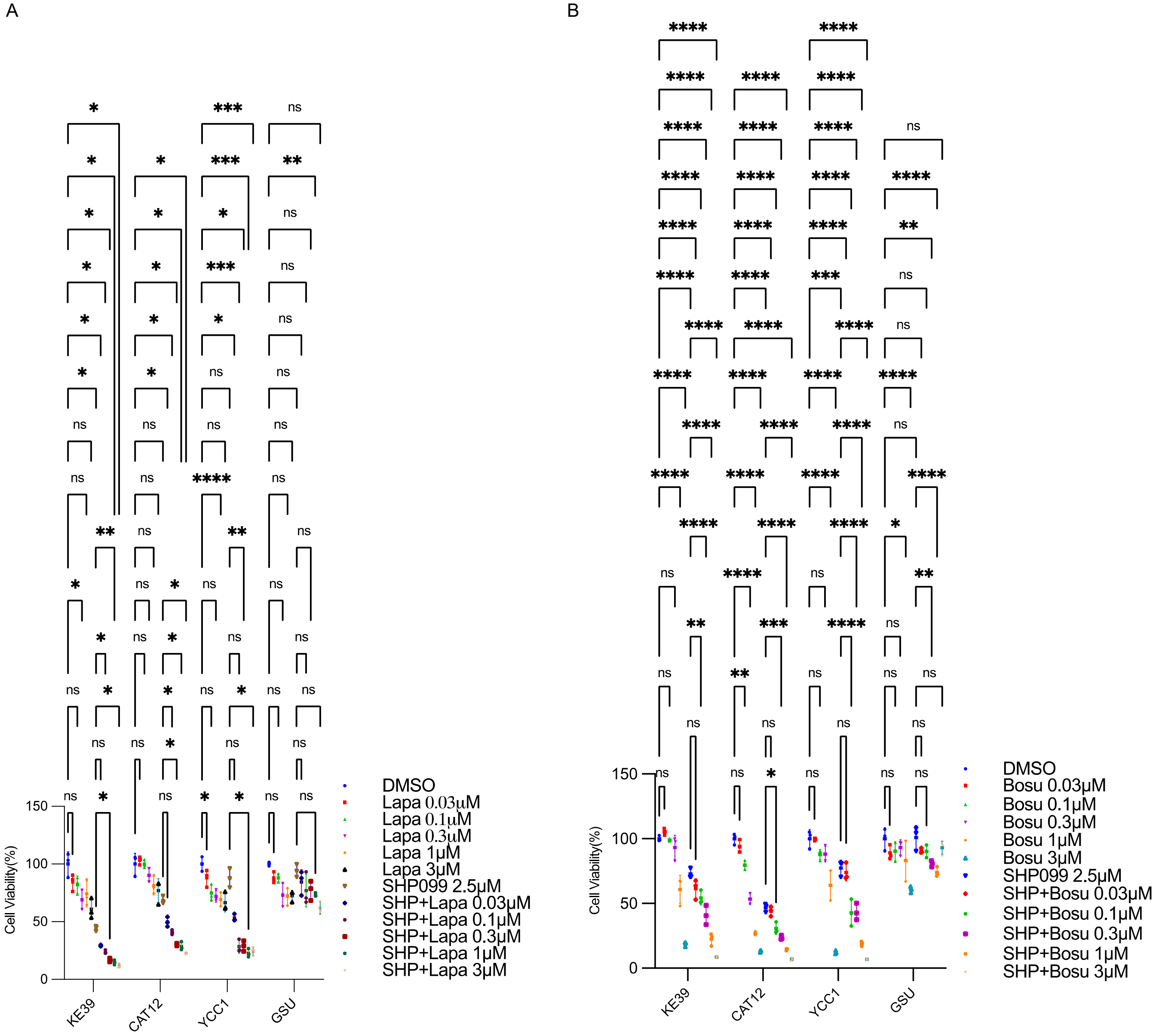

Suppl. Figure S4. (A) Quantification data for Fig3D crystal violet. (B) Quantification data for Fig4C crystal violet.

Suppl Fig S5

A

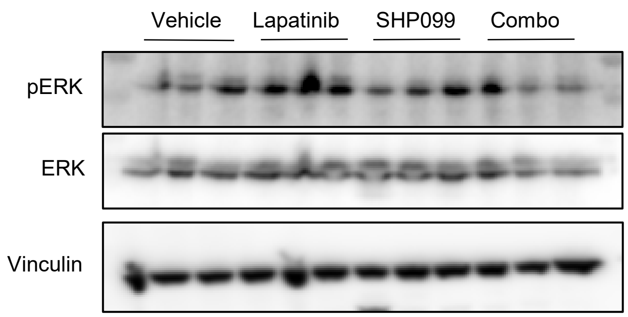

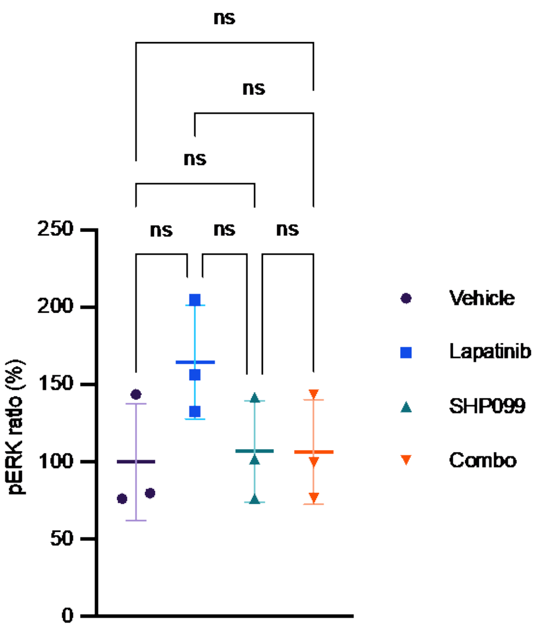

B

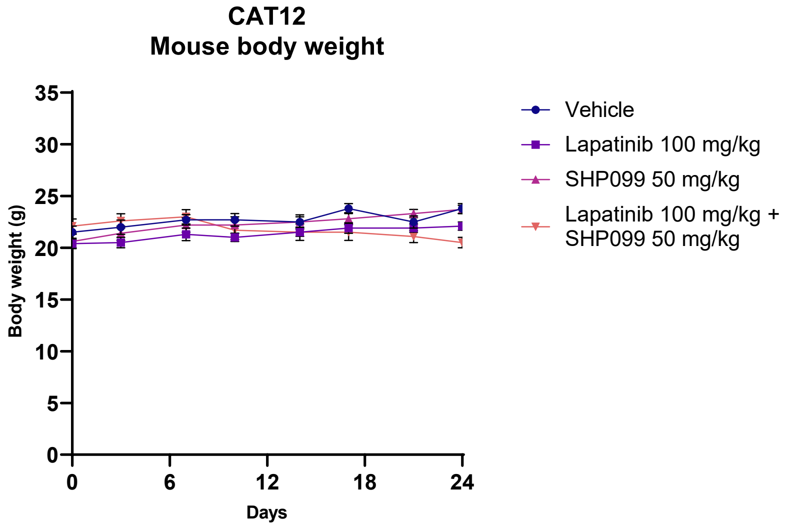

Suppl. Figure S5. (A) Representative Immunoblots of phospho-ERK of CAT12 tumors from indicated groups (n=3 per group). (B) Body weight changes of the nude mice with CAT12-derived xenografts in Figure 5A.

Suppl Fig6

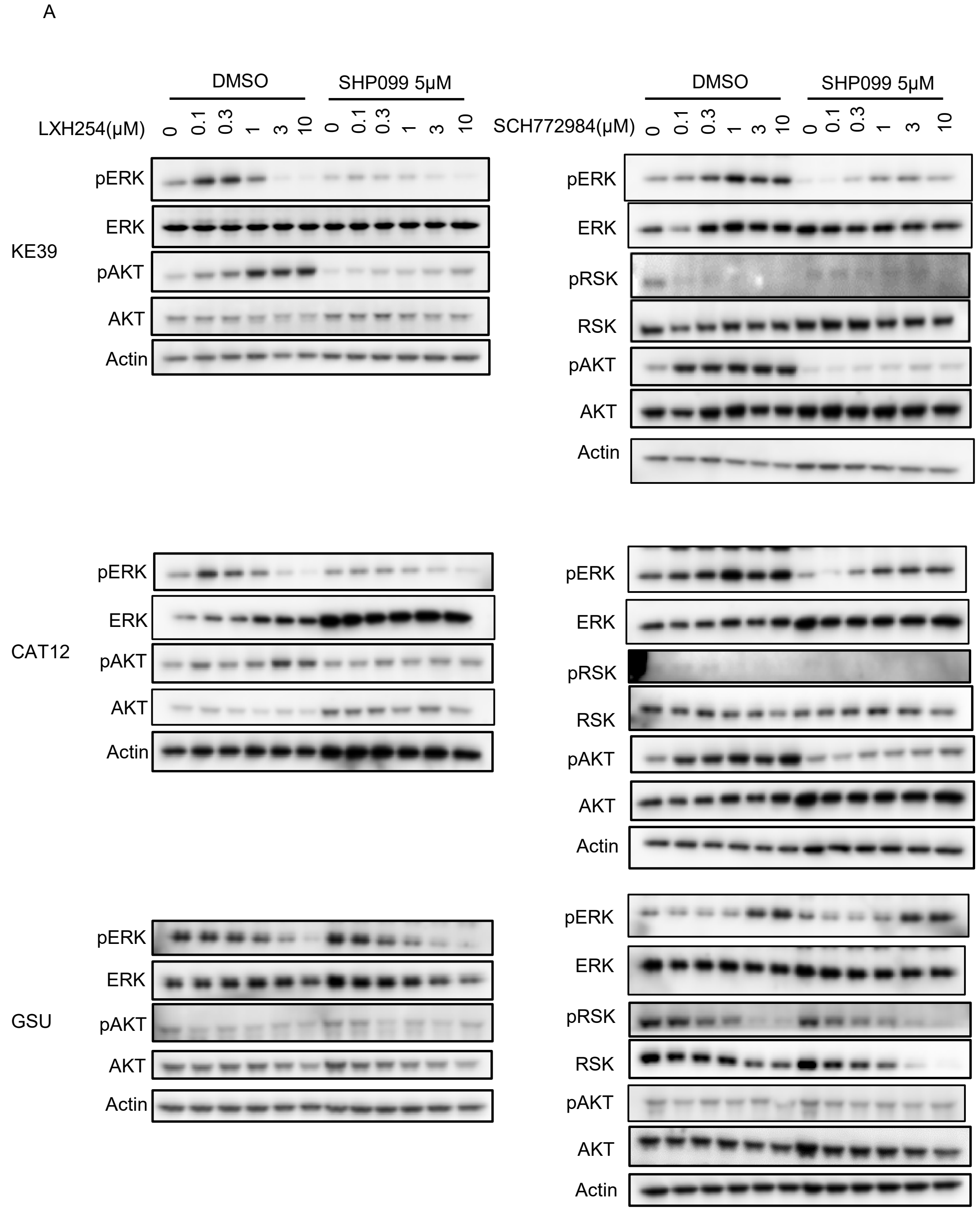

Suppl Figure S6. (A) Representative Immunoblots of phospho-ERK, ERK, phospho-RSK RSK, phosphor-AKT and AKT as markers of MAPK pathway activity in KE-39, CAT12 and GSU cells after 24h treatment of LXH254 (RAFi) and SCH772984 (ERKi) at indicated doses with or without SHP099 (SHP2i, 5 μM). DMSO is used as a vehicle control. βactin was used as a loading control.

Suppl Fig7

A

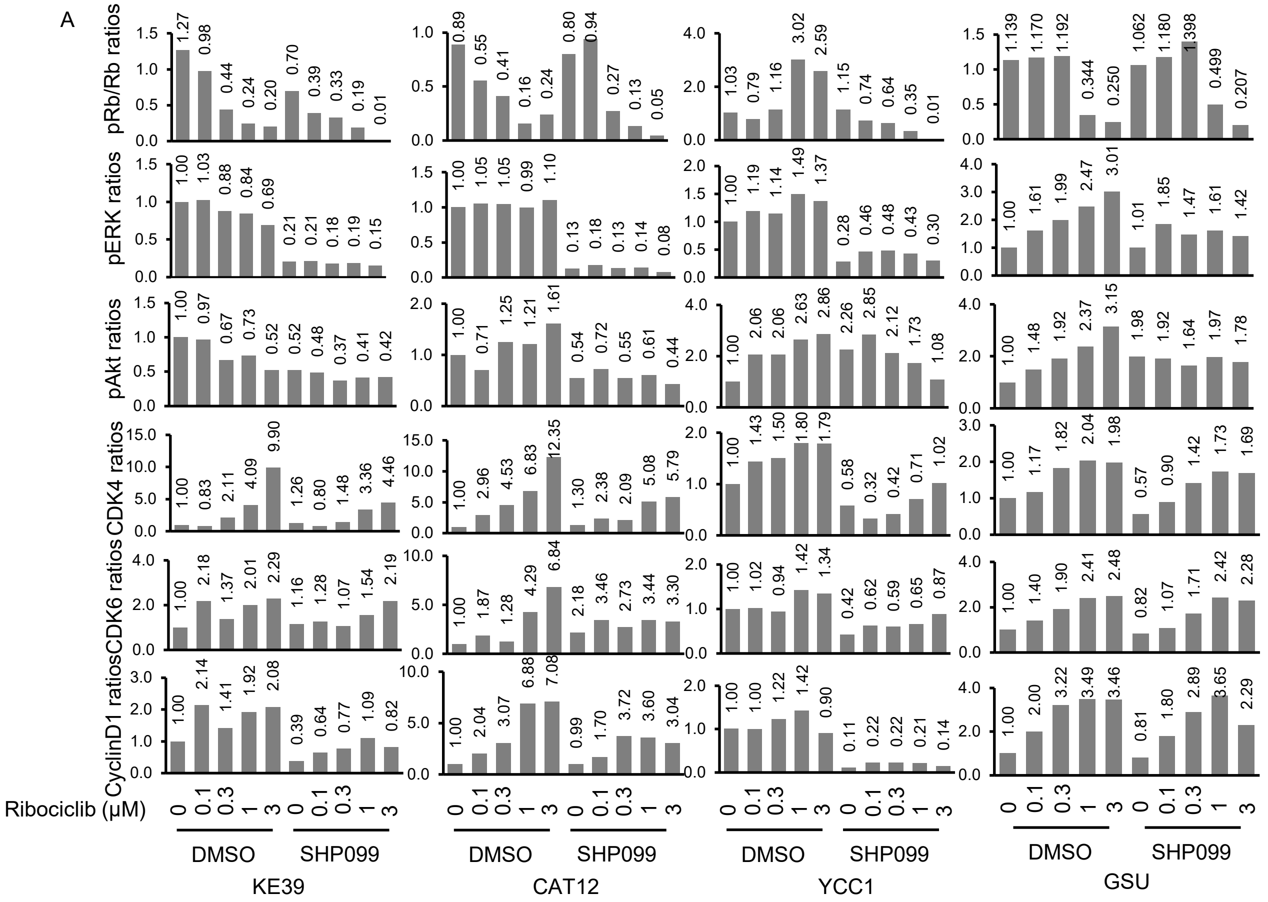

B

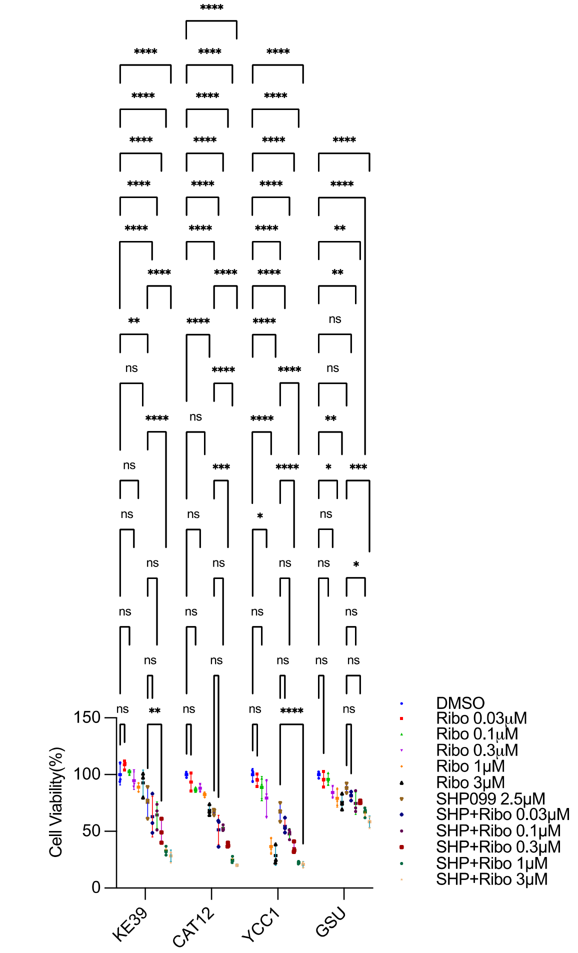

C

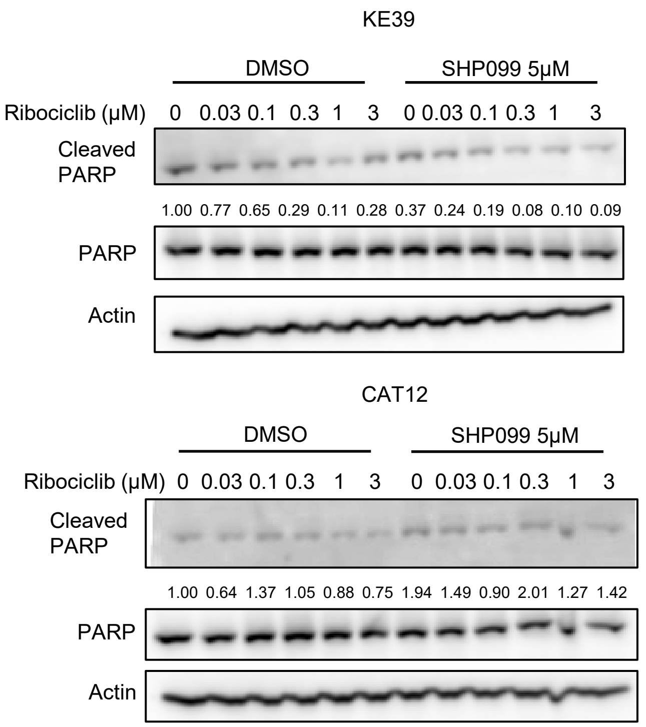

Suppl Figure S7. (A) Quantification data for Fig7D. (B) Quantification data for Fig7C.(C) Representative Immunoblots of cleaved PARP and PARP in KE-39 and CAT12 cells after 72h treatment of Ribociclib at indicated doses with or without SHP099 5µM. actin was used as a loading control.

Suppl Fig S8

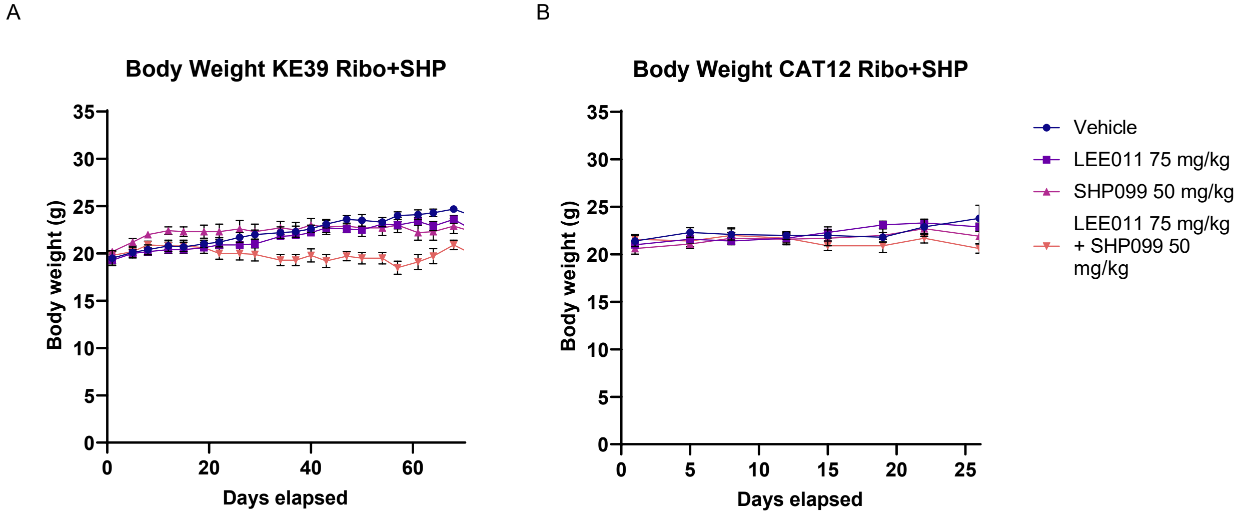

Suppl. Figure S8. (A) Body weight changes of the mice with KE39-dervied xenografts in Figure 7A. (B) Body weight changes of the mice with CAT12-dervied xenografts in Figure 7B.
