# Supplementary material for: Developing SHP2-based combination therapy for KRAS-amplified cancer
Source: JCI Insight. 2023 Feb 8;8(3):e152714. doi: 10.1172/jci.insight.152714 (PMC9977440; doi:10.1172/jci.insight.152714)
Supplement: Supplemental table 1 [file jciinsight-8-152714-s171.pdf]

Gene Symbol	KE39.Average LFC	HUG1N.Average LFC	Average LFC
SHOC2	-1.070867237	-0.705793476	-0.888330356
RANGAP1	-0.446875504	-0.39234488	-0.419610192
KDM3B	-0.575137122	-0.231555166	-0.403346144
RAF1	-0.626574435	-0.148506849	-0.387540642
PFDN1	-0.294679138	-0.46857023	-0.381624684
DPF1	-0.615735269	-0.135379364	-0.375557316
TGIF1	-0.517021162	-0.228866416	-0.372943789
PTK2	-0.710412846	-0.003393716	-0.356903281
CERS2	-0.140682248	-0.55578617	-0.348234209
POLR3G	-0.452759156	-0.241675958	-0.347217557
EXT2	-0.397763426	-0.294079427	-0.345921426
AUP1	-0.461570649	-0.222000342	-0.341785495
STAMBP	-0.276327131	-0.400890687	-0.338608909
UGCG	-0.61613621	-0.052946799	-0.334541504
SPNS1	0.056127257	-0.712295448	-0.328084096
RAC1	-0.569306988	-0.082078943	-0.325692965
POLR2H	-0.325346216	-0.323771005	-0.32455861
CRKL	-0.691464542	0.05147354	-0.319995501
FBXO11	-0.445407113	-0.184488337	-0.314947725
MSMO1	-0.131070232	-0.487936108	-0.30950317
VPS39	-0.313429549	-0.305304403	-0.309366976
BRAF	-0.207155527	-0.40982565	-0.308490588
CREBBP	-0.38903409	-0.216351168	-0.302692629
PLAGL2	-0.642422574	0.064473687	-0.288974444
CXCL14	-0.345401714	-0.230297025	-0.28784937
SLC25A1	-0.295761475	-0.278181277	-0.286971376
PRKCE	-0.30893687	-0.260418456	-0.284677663
STAM	-0.235430416	-0.312131756	-0.273781086
ZFPM1	-0.452584428	-0.094944117	-0.273764272
GID8	-0.579447711	0.032349777	-0.273548967
VPS18	-0.286850251	-0.260202344	-0.273526297
ITFG1	0.11462942	-0.655669486	-0.270520033
CNOT2	-0.496497967	-0.042541544	-0.269519756
RAB6A	-0.035168703	-0.502216099	-0.268692401
RABGAP1	-0.212016205	-0.322746798	-0.267381502
C9orf116	-0.401632238	-0.132809565	-0.267220901
PANX1	-0.26569844	-0.260952835	-0.263325637
IFIT5	-0.233429317	-0.287582359	-0.260505838
QPRT	-0.390439819	-0.129640162	-0.26003999
DHX8	-0.401560908	-0.117792664	-0.259676786
USP7	-0.487142818	-0.029118376	-0.258130597
MESP1	-0.251422768	-0.264228041	-0.257825405
PSMD3	-0.429002906	-0.073006414	-0.25100466
GAREM1	-0.385776668	-0.111022561	-0.248399615
PSMA6	-0.450240236	-0.043109189	-0.246674712
KRAS	-0.403869661	-0.08710477	-0.245487215
PREB	-0.5735172	0.087076788	-0.243220206
KLHDC3	-0.162269091	-0.32102938	-0.241649235

ARID5B	-0.242428717	-0.236614235	-0.239521476
C4orf48	-0.186985319	-0.288850698	-0.237918009
NLRC3	-0.411755125	-0.061895433	-0.236825279
SCD	0.079166352	-0.551493807	-0.236163728
SOX9	-0.520140199	0.056333527	-0.231903336
VPS11	-0.091203055	-0.370664951	-0.230934003
UBAP2L	-0.180375303	-0.281227652	-0.230801477
DDIT4	-0.14172771	-0.317757247	-0.229742479
FAM20B	-0.395639004	-0.06022004	-0.227929522
PSMD11	-0.273196426	-0.182186444	-0.227691435
FERMT1	-0.397922119	-0.056454089	-0.227188104
METTL5	-0.413663993	-0.040419658	-0.227041825
DDX46	-0.406199994	-0.046581553	-0.226390774
INPP5A	-0.405040591	-0.047061779	-0.226051185
SEC23B	0.025717204	-0.476823429	-0.225553112
SREBF2	-0.126947489	-0.322477011	-0.22471225
EBP	0.008029305	-0.45563312	-0.223801907
WDR26	-0.61292058	0.166619093	-0.223150744
NUDT4	-0.155885386	-0.289992449	-0.222938917
ATXN3L	-0.394116	-0.044667869	-0.219391935
SNX14	-0.124880999	-0.312905276	-0.218893138
CRYGB	-0.410789041	-0.02552048	-0.21815476
PRKAA1	-0.050646372	-0.380693884	-0.215670128
AGPAT3	-0.154163535	-0.27658954	-0.215376537
SMAD5	-0.15447522	-0.275591287	-0.215033253
LUZP4	-0.438183455	0.011675405	-0.213254025
TRAPPC2L	-0.130696534	-0.294730904	-0.212713719
DPM2	-0.517756535	0.094445244	-0.211655646
VHL	-0.436265796	0.014264582	-0.211000607
YAP1	-0.265200263	-0.155088647	-0.210144455
SLC11A2	-0.435512431	0.015411121	-0.210050655
VKORC1L1	-0.26908766	-0.150199583	-0.209643621
DCAF12L2	-0.294411306	-0.123874221	-0.209142764
CECR2	-0.443292054	0.02590867	-0.208691692
SMU1	-0.395015241	-0.018103879	-0.20655956
SLCO2B1	-0.46178075	0.049447048	-0.206166851
B3GAT3	-0.427646937	0.022242619	-0.202702159
PLEKHF1	-0.141558343	-0.262878891	-0.202218617
RIC8A	-0.091174823	-0.312183811	-0.201679317
SLC50A1	-0.33153937	-0.068545481	-0.200042425
FAU	-0.473235471	0.074050999	-0.199592236
CDC37	-0.440934988	0.046090708	-0.19742214
TMEM220	-0.429727223	0.039928007	-0.194899608
POLR2C	-0.201290014	-0.185301468	-0.193295741
SPTLC1	-0.098196821	-0.286857704	-0.192527263
SLBP	-0.412318443	0.028979904	-0.191669269
TTK	-0.244254118	-0.134933492	-0.189593805
FAXDC2	-0.248827415	-0.129909643	-0.189368529
TMEM263	-0.068078744	-0.309871667	-0.188975205

PCDH7	-0.113203837	-0.261344281	-0.187274059
CISD2	-0.445261161	0.071639139	-0.186811011
HCFC1	-0.465208237	0.092607154	-0.186300541
RUNX1	-0.418776398	0.046781264	-0.185997567
FAM71E2	-0.398698681	0.029205015	-0.184746833
MON2	0.036705082	-0.403951091	-0.183623004
MED11	-0.35670393	-0.009419818	-0.183061874
SLC22A25	-0.421345781	0.056432243	-0.182456769
SIRT1	-0.438741096	0.073929797	-0.18240565
CDK6	-0.470358165	0.105615015	-0.182371575
CAMK2D	-0.386512561	0.023049902	-0.18173133
YPEL5	-0.521308102	0.159417763	-0.18094517
CSNK2A2	-0.422846779	0.061319491	-0.180763644
PTGS1	-0.436017424	0.076659293	-0.179679065
LMTK3	-0.464194678	0.107057935	-0.178568372
GLOD4	-0.389108283	0.032187399	-0.178460442
XBP1	-0.449818415	0.093691768	-0.178063324
SPRR1B	-0.297651698	-0.056987848	-0.177319773
CDC23	-0.250958995	-0.102450674	-0.176704835
SMC1A	-0.402876118	0.049832626	-0.176521746
SNRPF	-0.434975927	0.082146563	-0.176414682
RPL31	-0.258452031	-0.092803674	-0.175627852
BSCL2	-0.069498984	-0.281563757	-0.17553137
SNX13	-0.032762198	-0.31782021	-0.175291204
CCND1	-0.524317188	0.177430775	-0.173443207
UBE2Q2L	-0.516203474	0.169799802	-0.173201836
VPS16	0.050144453	-0.393247975	-0.171551761
MAEA	-0.612871097	0.270496197	-0.17118745
CD1E	-0.069439014	-0.268942724	-0.169190869
TRAPPC4	-0.328836772	-0.008309274	-0.168573023
INTS6	-0.265853204	-0.071130133	-0.168491668
CCDC6	0.006096636	-0.341031857	-0.167467611
POLR1A	-0.503254861	0.174666336	-0.164294263
HACD2	0.066644619	-0.393225235	-0.163290308
MED12	-0.236964467	-0.085747761	-0.161356114
RHOA	-0.398592285	0.076618298	-0.160986993
RPS8	-0.471445508	0.152071585	-0.159686961
HSPE1	-0.421037367	0.10332244	-0.158857464
RPS4X	-0.41343746	0.098427827	-0.157504816
BPIFB2	-0.052981177	-0.261063672	-0.157022425
OSGIN1	-0.409259612	0.099317165	-0.154971224
ELOVL1	0.039530661	-0.348393073	-0.154431206
RTN4R	-0.454513545	0.148261645	-0.15312595
LIPK	-0.025187696	-0.279184644	-0.15218617
COX4I2	-0.40568475	0.10202827	-0.15182824
SET	-0.40768372	0.107313945	-0.150184887
INIP	-0.123067934	-0.176104903	-0.149586418
PDCD6IP	-0.020783421	-0.27607048	-0.148426951
MCCC1	-0.380807532	0.084199702	-0.148303915

PFDN5	0.020717335	-0.31470112	-0.146991892
NEDD9	-0.403852651	0.111913292	-0.145969679
ASXL3	-0.011678599	-0.280102573	-0.145890586
GINS2	-0.36163077	0.070598004	-0.145516383
VCP	-0.363815304	0.074397355	-0.144708975
TM9SF3	0.103253118	-0.392532111	-0.144639496
UBL5	-0.387986618	0.102858804	-0.142563907
DTYMK	-0.32067923	0.037687784	-0.141495723
BPTF	0.021077077	-0.302529381	-0.140726152
MMP9	0.051322116	-0.329605383	-0.139141634
RPS13	-0.216339671	-0.061770749	-0.13905521
ZNF429	-0.13612373	-0.141076774	-0.138600252
DPM1	-0.403308762	0.129258709	-0.137025027
ST3GAL6	-0.005578212	-0.268083678	-0.136830945
MAF1	-0.396357202	0.123113384	-0.136621909
TBCB	-0.096215821	-0.176655401	-0.136435611
SLC30A1	0.125727364	-0.398414059	-0.136343347
PPP1R7	-0.196435645	-0.074949335	-0.13569249
PHB	-0.318758747	0.048290635	-0.135234056
ATP2A2	-0.148458286	-0.117572083	-0.133015185
PSMB1	-0.156107542	-0.104248028	-0.130177785
UBTFL1	0.00193619	-0.260194841	-0.129129326
LGALS7B	-0.140150242	-0.114016123	-0.127083182
RIC1	-0.07386207	-0.175073122	-0.124467596
TRAPPC5	-0.214993647	-0.032945974	-0.123969811
MAGEA12	-0.100602484	-0.144166926	-0.122384705
RPL26L1	0.023559988	-0.267058329	-0.121749171
GPRC6A	0.032943021	-0.272016471	-0.119536725
RIMS4	0.028677026	-0.266379616	-0.118851295
SNRPD3	-0.355495529	0.121153604	-0.117170963
CHD2	-0.445184138	0.220165426	-0.112509356
CHCHD3	-0.38646166	0.161904799	-0.112278431
UBE2H	-0.430380769	0.217430244	-0.106475262
GMDS	0.089465232	-0.301682934	-0.106108851
IRF2	0.142710946	-0.349524572	-0.103406813
ANAPC11	-0.328498283	0.122628029	-0.102935127
RPS18	-0.281980453	0.076327502	-0.102826475
ATP6V0A1	0.106376961	-0.308507408	-0.101065223
FAM217B	0.058347357	-0.259541411	-0.100597027
CHSY1	0.080345199	-0.279641394	-0.099648097
TRIM64B	-0.126346954	-0.067045202	-0.096696078
LPCAT3	0.223566576	-0.414411169	-0.095422297
DNAJC9	-0.149550938	-0.040205121	-0.094878029
SPTLC2	0.094481601	-0.28087425	-0.093196325
CCT4	-0.312310801	0.127832415	-0.092239193
RSPH3	-0.160692652	-0.017040172	-0.088866412
FUNDC2	0.097305889	-0.275009992	-0.088852051
MZT1	-0.160302387	-0.016810372	-0.08855638
SPC25	-0.00749795	-0.164063988	-0.085780969

IFITM1	-0.180825039	0.010390383	-0.085217328
UBAP1	0.010722984	-0.1795742	-0.084425608
AURKA	-0.090373512	-0.072896856	-0.081635184
NUP37	0.166964936	-0.320313188	-0.076674126
GATA6	-0.491015324	0.346033833	-0.072490746
C12orf77	-0.111693884	-0.028817376	-0.07025563
MAGEA6	-0.114368687	-0.025336857	-0.069852772
PLCXD3	0.124291476	-0.257385713	-0.066547119
ARSI	-0.137410919	0.005648151	-0.065881384
VPS25	-0.0889904	-0.04212574	-0.06555807
ESRP1	0.289172155	-0.418808036	-0.06481794
LONRF1	-0.000868266	-0.128343919	-0.064606093
SRSF2	-0.12719572	-0.001655534	-0.064425627
NRTN	-0.104875879	-0.022915974	-0.063895926
PHAX	-0.098923891	-0.026884908	-0.0629044
RAB3GAP2	0.178528461	-0.303295381	-0.06238346
EMC3	0.014322205	-0.132241115	-0.058959455
PRPF38B	-0.253972968	0.141498915	-0.056237026
SAP18	-0.089282347	-0.019593254	-0.054437801
RGPD8	-0.174712267	0.0659895	-0.054361384
TCF7L2	-0.395385254	0.287593117	-0.053896069
KPNB1	-0.465229454	0.357485777	-0.053871838
TTC7A	0.067842732	-0.153931781	-0.043044525
VPS45	0.273316061	-0.35765977	-0.042171854
CTNNBL1	0.262779227	-0.340861313	-0.039041043
MTAP	0.190274914	-0.2613714	-0.035548243
PEX16	0.0506271	-0.108236547	-0.028804724
VTA1	0.221919999	-0.276832601	-0.027456301
TUBB	-0.100701541	0.052203076	-0.024249233
PEX3	0.221135065	-0.263839768	-0.021352351
TUBGCP2	-0.113759938	0.073584906	-0.020087516
ATL2	0.1747969	-0.200297524	-0.012750312
TSPAN13	0.260073265	-0.270361839	-0.005144287
WDR70	-0.415038996	0.40646652	-0.004286238
ACVR1B	0.254289321	-0.262184248	-0.003947464
RNF103-CHMP3	0.307654793	-0.308234021	-0.000289614
EXOSC9	-0.481222184	0.481953155	0.000365485
CHMP3	0.291410848	-0.286802345	0.002304252
TBC1D3B	-0.041466941	0.159466829	0.058999944
FNTB	-0.022229742	0.148706525	0.063238392
FLVCR1	0.456361387	-0.320856415	0.067752486
UBE2N	0.445690411	-0.270069659	0.087810376
NPRL3	0.501645998	-0.312066669	0.094789665
UAP1	-0.440034507	0.64466512	0.102315307
SREBF1	0.481968447	-0.277210987	0.10237873
NPRL2	0.544293765	-0.282923641	0.130685062
STK11	1.398400055	-0.412920809	0.492739623
TSC1	1.57897437	-0.398604518	0.590184926
TSC2	2.069535907	-0.623222692	0.723156607
