# Supplementary material for: Developing SHP2-based combination therapy for KRAS-amplified cancer
Source: JCI Insight. 2023 Feb 8;8(3):e152714. doi: 10.1172/jci.insight.152714 (PMC9977440; doi:10.1172/jci.insight.152714)
Supplement: Supplemental table 2 [file jciinsight-8-152714-s172.pdf]

NCBI Gene ID	Gene Symbol
**91**	ACVR1B
**56894**	AGPAT3
**207**	AKT1
**208**	AKT2
**10000**	AKT3
**238**	ALK
**51529**	ANAPC11
**317**	APAF1
**324**	APC
**369**	ARAF
**2909**	ARHGAP35
**9181**	ARHGEF2
**84159**	ARID5B
**340075**	ARSI
**80816**	ASXL3
**64225**	ATL2
**488**	ATP2A2
**535**	ATP6V0A1
**92552**	ATXN3L
**550**	AUP1
**6790**	AURKA
**26229**	B3GAT3
**580**	BARD1
**596**	BCL2
**598**	BCL2L1
**329**	BIRC2
**330**	BIRC3
**332**	BIRC5
**79444**	BIRC7
**640**	BLK
**80341**	BPIFB2
**2186**	BPTF
**673**	BRAF
**672**	BRCA1
**675**	BRCA2
**83990**	BRIP1
**26580**	BSCL2
**699**	BUB1
**79794**	C12orf49
**196415**	C12orf77
**401115**	C4orf48
**138162**	C9orf116
**817**	CAMK2D
**836**	CASP3
**840**	CASP7
**841**	CASP8

**867**	CBL
**868**	CBLB
**23624**	CBLC
**8030**	CCDC6
**8900**	CCNA1
**890**	CCNA2
**595**	CCND1
**894**	CCND2
**896**	CCND3
**10575**	CCT4
**913**	CD1E
**8697**	CDC23
**993**	CDC25A
**11140**	CDC37
**990**	CDC6
**1017**	CDK2
**1019**	CDK4
**1021**	CDK6
**1026**	CDKN1A
**27443**	CECR2
**29956**	CERS2
**54927**	CHCHD3
**1106**	CHD2
**51652**	CHMP3
**22856**	CHSY1
**493856**	CISD2
**10256**	CNKSR1
**22866**	CNKSR2
**4848**	CNOT2
**84701**	COX4I2
**1387**	CREBBP
**1399**	CRKL
**1419**	CRYGB
**1459**	CSNK2A2
**1499**	CTNNB1
**56259**	CTNNBL1
**9547**	CXCL14
**9266**	CYTH2
**153090**	DAB2IP
**340578**	DCAF12L2
**54541**	DDIT4
**9879**	DDX46
**1719**	DHFR
**1659**	DHX8
**23234**	DNAJC9
**8193**	DPF1
**8813**	DPM1

**8818**	DPM2
**1841**	DTYMK
**1843**	DUSP1
**1844**	DUSP2
**1845**	DUSP3
**1846**	DUSP4
**1847**	DUSP5
**1848**	DUSP6
**1869**	E2F1
**1870**	E2F2
**1871**	E2F3
**144455**	E2F7
**10682**	EBP
**1894**	ECT2
**8726**	EED
**1956**	EGFR
**1978**	EIF4EBP1
**1979**	EIF4EBP2
**8637**	EIF4EBP3
**2002**	ELK1
**64834**	ELOVL1
**55831**	EMC3
**2064**	ERBB2
**2077**	ERF
**54845**	ESRP1
**2113**	ETS1
**2114**	ETS2
**55763**	EXOC1
**55770**	EXOC2
**11336**	EXOC3
**60412**	EXOC4
**10640**	EXOC5
**54536**	EXOC6
**23265**	EXOC7
**149371**	EXOC8
**5393**	EXOSC9
**2132**	EXT2
**2146**	EZH2
**9917**	FAM20B
**63939**	FAM217B
**284418**	FAM71E2
**2175**	FANCA
**2176**	FANCC
**2197**	FAU
**10826**	FAXDC2
**80204**	FBXO11
**55612**	FERMT1

**2260**	FGFR1
**2263**	FGFR2
**2261**	FGFR3
**2264**	FGFR4
**2268**	FGR
**2322**	FLT3
**28982**	FLVCR1
**2339**	FNTA
**2342**	FNTB
**2353**	FOS
**8061**	FOSL1
**2444**	FRK
**65991**	FUNDC2
**2534**	FYN
**64762**	GAREM1
**2627**	GATA6
**54994**	GID8
**51659**	GINS2
**51031**	GLOD4
**2762**	GMDS
**222545**	GPRC6A
**2887**	GRB10
**2885**	GRB2
**201562**	HACD2
**3054**	HCFC1
**3055**	HCK
**3065**	HDAC1
**83933**	HDAC10
**79885**	HDAC11
**3066**	HDAC2
**8841**	HDAC3
**9759**	HDAC4
**10014**	HDAC5
**10013**	HDAC6
**51564**	HDAC7
**55869**	HDAC8
**9734**	HDAC9
**3265**	HRAS
**3336**	HSPE1
**23463**	ICMT
**24138**	IFIT5
**8519**	IFITM1
**58493**	INIP
**8821**	INPP4B
**3632**	INPP5A
**3643**	INSR
**3645**	INSRR

**26512**	INTS6
**3660**	IRF2
**3667**	IRS1
**8660**	IRS2
**83737**	ITCH
**81533**	ITFG1
**3716**	JAK1
**3717**	JAK2
**3718**	JAK3
**3725**	JUN
**51780**	KDM3B
**3808**	KIR2DS3
**116138**	KLHDC3
**3837**	KPNB1
**3845**	KRAS
**8844**	KSR1
**283455**	KSR2
**3932**	LCK
**653499**	LGALS7B
**643414**	LIPK
**114783**	LMTK3
**91694**	LONRF1
**10162**	LPCAT3
**51213**	LUZP4
**4067**	LYN
**10296**	MAEA
**84232**	MAF1
**4111**	MAGEA12
**4105**	MAGEA6
**5604**	MAP2K1
**5605**	MAP2K2
**5594**	MAPK1
**5595**	MAPK3
**51257**	MARCH2
**56922**	MCCC1
**4172**	MCM3
**4173**	MCM4
**4174**	MCM5
**4175**	MCM6
**4176**	MCM7
**4193**	MDM2
**400569**	MED11
**9968**	MED12
**55897**	MESP1
**4233**	MET
**29081**	METTL5
**64223**	MLST8

**4318**	MMP9
**23041**	MON2
**22808**	MRAS
**6307**	MSMO1
**4507**	MTAP
**2475**	MTOR
**4602**	MYB
**4609**	MYC
**440145**	MZT1
**4671**	NAIP
**4739**	NEDD9
**4763**	NF1
**4780**	NFE2L2
**4790**	NFKB1
**197358**	NLRC3
**27035**	NOX1
**10641**	NPRL2
**8131**	NPRL3
**4893**	NRAS
**4902**	NRTN
**7468**	NSD2
**11163**	NUDT4
**79023**	NUP37
**29948**	OSGIN1
**5058**	PAK1
**5062**	PAK2
**5063**	PAK3
**10298**	PAK4
**24145**	PANX1
**5099**	PCDH7
**10015**	PDCD6IP
**5156**	PDGFRA
**5159**	PDGFRB
**5170**	PDPK1
**8682**	PEA15
**5037**	PEBP1
**9409**	PEX16
**8504**	PEX3
**5201**	PFDN1
**5204**	PFDN5
**51808**	PHAX
**5245**	PHB
**5290**	PIK3CA
**5293**	PIK3CD
**5294**	PIK3CG
**5295**	PIK3R1
**5296**	PIK3R2

**8503**	PIK3R3
**23533**	PIK3R5
**146850**	PIK3R6
**5300**	PIN1
**5326**	PLAGL2
**51196**	PLCE1
**345557**	PLCXD3
**79156**	PLEKHF1
**5364**	PLXNB1
**25885**	POLR1A
**5432**	POLR2C
**5437**	POLR2H
**10622**	POLR3G
**5499**	PPP1CA
**5510**	PPP1R7
**10113**	PREB
**80243**	PREX2
**5562**	PRKAA1
**5563**	PRKAA2
**5564**	PRKAB1
**5565**	PRKAB2
**5571**	PRKAG1
**51422**	PRKAG2
**53632**	PRKAG3
**5578**	PRKCA
**5579**	PRKCB
**5580**	PRKCD
**5581**	PRKCE
**5582**	PRKCG
**5583**	PRKCH
**5584**	PRKCI
**5588**	PRKCQ
**5589**	PRKCSH
**5590**	PRKCZ
**55119**	PRPF38B
**5687**	PSMA6
**5689**	PSMB1
**5717**	PSMD11
**5709**	PSMD3
**5728**	PTEN
**5742**	PTGS1
**5747**	PTK2
**5781**	PTPN11
**23475**	QPRT
**25782**	RAB3GAP2
**5870**	RAB6A
**23637**	RABGAP1

**5879**	RAC1
**5880**	RAC2
**5881**	RAC3
**5893**	RAD52
**5894**	RAF1
**5898**	RALA
**5899**	RALB
**10928**	RALBP1
**253959**	RALGAPA1
**57186**	RALGAPA2
**5900**	RALGDS
**5905**	RANGAP1
**2889**	RAPGEF1
**9693**	RAPGEF2
**5921**	RASA1
**5922**	RASA2
**22821**	RASA3
**8437**	RASAL1
**9462**	RASAL2
**64926**	RASAL3
**5923**	RASGRF1
**5924**	RASGRF2
**10125**	RASGRP1
**10235**	RASGRP2
**25780**	RASGRP3
**115727**	RASGRP4
**11186**	RASSF1
**644943**	RASSF10
**9770**	RASSF2
**283349**	RASSF3
**83937**	RASSF4
**83593**	RASSF5
**166824**	RASSF6
**8045**	RASSF7
**11228**	RASSF8
**9182**	RASSF9
**5925**	RB1
**9986**	RCE1
**23179**	RGL1
**5863**	RGL2
**57139**	RGL3
**727851**	RGPD8
**6009**	RHEB
**387**	RHOA
**388**	RHOB
**389**	RHOC
**57589**	RIC1

**60626**	RIC8A
**140730**	RIMS4
**100526767**	RNF103-CHMP3
**6093**	ROCK1
**9475**	ROCK2
**6098**	ROS1
**51121**	RPL26L1
**6160**	RPL31
**6207**	RPS13
**6222**	RPS18
**6191**	RPS4X
**6195**	RPS6KA1
**6196**	RPS6KA2
**6197**	RPS6KA3
**27330**	RPS6KA6
**6198**	RPS6KB1
**6199**	RPS6KB2
**6202**	RPS8
**57521**	RPTOR
**83861**	RSPH3
**65078**	RTN4R
**861**	RUNX1
**10284**	SAP18
**60485**	SAV1
**6319**	SCD
**23513**	SCRIB
**10483**	SEC23B
**6418**	SET
**6464**	SHC1
**25759**	SHC2
**53358**	SHC3
**399694**	SHC4
**8036**	SHOC2
**23411**	SIRT1
**7884**	SLBP
**4891**	SLC11A2
**387601**	SLC22A25
**6576**	SLC25A1
**7779**	SLC30A1
**55974**	SLC50A1
**11309**	SLCO2B1
**4090**	SMAD5
**8243**	SMC1A
**55234**	SMU1
**6634**	SNRPD3
**6636**	SNRPF
**23161**	SNX13

**57231**	SNX14
**6654**	SOS1
**6655**	SOS2
**6662**	SOX9
**57405**	SPC25
**83985**	SPNS1
**161742**	SPRED1
**200734**	SPRED2
**399473**	SPRED3
**6699**	SPRR1B
**10252**	SPRY1
**10253**	SPRY2
**10251**	SPRY3
**81848**	SPRY4
**10558**	SPTLC1
**9517**	SPTLC2
**6714**	SRC
**6720**	SREBF1
**6721**	SREBF2
**6427**	SRSF2
**10402**	ST3GAL6
**8027**	STAM
**10617**	STAMBP
**6794**	STK11
**6788**	STK3
**6789**	STK4
**23512**	SUZ12
**414059**	TBC1D3B
**1155**	TBCB
**29110**	TBK1
**6934**	TCF7L2
**7027**	TFDP1
**7029**	TFDP2
**7050**	TGIF1
**7074**	TIAM1
**26230**	TIAM2
**7083**	TK1
**56889**	TM9SF3
**388335**	TMEM220
**90488**	TMEM263
**7157**	TP53
**7161**	TP73
**51693**	TRAPPC2L
**51399**	TRAPPC4
**126003**	TRAPPC5
**642446**	TRIM64B
**7248**	TSC1

**7249**	TSC2
**27075**	TSPAN13
**57217**	TTC7A
**7272**	TTK
**203068**	TUBB
**10844**	TUBGCP2
**7298**	TYMS
**6675**	UAP1
**51271**	UBAP1
**9898**	UBAP2L
**7328**	UBE2H
**7334**	UBE2N
**100505679**	UBE2Q2L
**59286**	UBL5
**642623**	UBTFL1
**7357**	UGCG
**7374**	UNG
**7874**	USP7
**7409**	VAV1
**7415**	VCP
**7428**	VHL
**154807**	VKORC1L1
**55823**	VPS11
**64601**	VPS16
**57617**	VPS18
**84313**	VPS25
**23339**	VPS39
**11311**	VPS45
**51534**	VTA1
**80232**	WDR26
**55100**	WDR70
**7494**	XBP1
**331**	XIAP
**57002**	YAE1D1
**10413**	YAP1
**7525**	YES1
**51646**	YPEL5
**146198**	ZFP90
**161882**	ZFPM1
**353088**	ZNF429
