# Supplementary material for: Developing SHP2-based combination therapy for KRAS-amplified cancer
Source: JCI Insight. 2023 Feb 8;8(3):e152714. doi: 10.1172/jci.insight.152714 (PMC9977440; doi:10.1172/jci.insight.152714)
Supplement: Supplemental table 3 [file jciinsight-8-152714-s173.pdf]

Barcode Sequence	Gene Symbol	NCBI Gene ID
**AAAAAACCAAATCGCCACGT**	ONE_NON-GENE_SITE_1	ONE_NON-GENE_SITE_1
**AAAAACCAGACAACTACCAA**	BARD1	580
**AAAAACTGCAATAGCAAACT**	YPEL5	51646
**AAAAATAAGGAACTTGAACA**	FERMT1	55612
**AAAAATCAAGAAAGATGGGA**	PRPF38B	55119
**AAAACACATCAGTATAACAT**	RHOA	387
**AAAACAGTTAGCCAGAGGTT**	PIK3CA	5290
**AAAACCAGAAGCGTACCCAG**	RGL1	23179
**AAAACCTCAAAATATGATGG**	ITFG1	81533
**AAAACGACCACCCGAAACCT**	ONE_NON-GENE_SITE_2	ONE_NON-GENE_SITE_2
**AAAACTATGTCAATAAGCGC**	ONE_NON-GENE_SITE_3	ONE_NON-GENE_SITE_3
**AAAACTGTCATACATGAATC**	RAB6A	5870
**AAAAGATACTCGACAAGACT**	RAPGEF2	9693
**AAAAGATTGTGACGAATGGG**	RASGRF1	5923
**AAAAGCAGCCTATGACCGGG**	RALBP1	10928
**AAAAGCCAAAAATACAGGAA**	UBTFL1	642623
**AAAAGCTGTTTCAGTAGAGA**	C12orf77	196415
**AAAAGCTTTCTGCTAGGGGT**	CAMK2D	817
**AAAAGGATATTGTGCAACTG**	PTEN	5728
**AAAATAGAGAATTACTGGAA**	RGPD5	84220
**AAAATAGAGAATTACTGGAA**	RGPD6	729540
**AAAATAGAGAATTACTGGAA**	RGPD8	727851
**AAAATGACAGAAACCCAAGC**	FLT3	2322
**AAAATGGATTATCCTGAGAT**	CASP3	836
**AAAATGGGCTTGCCCTGGTG**	HDAC10	83933
**AAACAATCAAAGGACCGAGA**	RB1	5925
**AAACACTCACCGATCTGTGA**	RPS13	6207
**AAACATTACCTACCAAGTAT**	RALGAPA1	253959
**AAACCAGGATAGTAATCTAT**	FANCC	2176
**AAACCATCCCAAAAACCTTC**	LUZP4	51213
**AAACCGACAACAGACTGATA**	HDAC2	3066
**AAACCTATGAATCGTCACAG**	ONE_NON-GENE_SITE_4	ONE_NON-GENE_SITE_4
**AAACGAGCTACCAAACCAAA**	RASA1	5921
**AAACGCAGAATAAAGCCAGC**	SET	6418
**AAACGCAGAATAAAGCCAGC**	SETP6	100419178
**AAACGCAGAATAAAGCCAGC**	SETSIP	646817
**AAACTCAATCGGGCAACAGA**	EXOSC9	5393
**AAACTCTGTCTATTTGTGTA**	MZT1	440145
**AAACTGGTGGTGGTTGGAGC**	NRAS	4893
**AAACTTATCGCTACCATACA**	ST3GAL6	10402
**AAACTTGTGGTAGTTGGAGC**	KRAS	3845
**AAAGAAGCTGGTGATCGTTG**	RHOC	389
**AAAGAAGTCTGATTATCCAG**	SPTLC1	10558
**AAAGACAGGTTGCTCAAAAG**	TTK	7272
**AAAGACCACAACACGTAGCG**	PIK3R5	23533
**AAAGACCAGCTGCAAGGAAT**	MAGEA6	4105

**AAAGACCCTGTGAATCAGAG**	SHC1	6464
**AAAGAGCAGACTTATTACCA**	SHC3	53358
**AAAGCAGAAATACTGTGCAT**	RAPGEF2	9693
**AAAGCAGTTACTCTTCAACA**	YAE1D1	57002
**AAAGCCAATTCAGGAGCCAG**	RALBP1	10928
**AAAGCCCGTTTGGATCCATA**	PRKCA	5578
**AAAGCTGGCTTTGATAAACA**	ST3GAL6	10402
**AAAGCTGTCTGTGAAATTAA**	SPC25	57405
**AAAGCTGTTCATGTGAACCA**	FLT3	2322
**AAAGGACCATAGTACTCTGA**	RASA2	5922
**AAAGGAGCAGAGCAAAACCG**	BPTF	2186
**AAAGGATGGAAAATACAGCC**	RPS18	6222
**AAAGGCACTCCAACTAAATG**	ONE_NON-GENE_SITE_5	ONE_NON-GENE_SITE_5
**AAAGGTGCAAGGCACCAACT**	RPS6KB2	6199
**AAAGGTTCTGAATCAAACGG**	ATP6V0A1	535
**AAAGTACTGCATGAGGCCGA**	SNRPD3	6634
**AAAGTCAAGAAATCCGATAA**	EXOC1	55763
**AAAGTCCGGAGCCGATCACA**	INTS6	26512
**AAAGTTCTTCTCCCAGAATA**	HSPE1	3336
**AAAGTTCTTCTCCCAGAATA**	HSPE1P3	100507046
**AAAGTTCTTCTCCCAGAATA**	HSPE1P4	100287369
**AAAGTTGAAGTCTTCCCGAC**	ONE_NON-GENE_SITE_6	ONE_NON-GENE_SITE_6
**AAATAACTTAATGTCACTAC**	SPTLC1	10558
**AAATAATACCTGTCCTCCCT**	LIPK	643414
**AAATAATCCGTCATTCCTAG**	PDGFRA	5156
**AAATACCATACCTTGCAAGG**	MTAP	4507
**AAATACTGCTGACGTCGTCG**	RASGRF2	5924
**AAATAGAATACCTATTATGT**	RHEB	6009
**AAATATCAAGTATATATGGT**	CBLB	868
**AAATATCATGCGTGTTATAG**	TBK1	29110
**AAATCAACAGGTTCGTCACA**	ROCK2	9475
**AAATCACTGACATTTACAGG**	RASSF8	11228
**AAATCGCTAAGGTAGCATAT**	ONE_NON-GENE_SITE_7	ONE_NON-GENE_SITE_7
**AAATCGTGATAGAATTCCAG**	EXOC6	54536
**AAATCTACAACATGGGACGG**	YAE1D1	57002
**AAATCTGCCAACTACCAAGG**	FAM20B	9917
**AAATGAACTTGCCTATCAAA**	EXOC5	10640
**AAATGAATTACAGATTGAAC**	RASSF8	11228
**AAATGCCAGGAAGCTACTGC**	MED12	9968
**AAATGTCCATAACGTTACTA**	ONE_NON-GENE_SITE_8	ONE_NON-GENE_SITE_8
**AAATGTTACTGACCTTTCAG**	PTPN11	5781
**AAATTATCTGACAAAGACCA**	RGPD5	84220
**AAATTATCTGACAAAGACCA**	RGPD6	729540
**AAATTATCTGACAAAGACCA**	RGPD8	727851
**AAATTATTCTCATCACGATA**	RGPD5	84220
**AAATTATTCTCATCACGATA**	RGPD6	729540
**AAATTATTCTCATCACGATA**	RGPD8	727851

**AAATTGAGTCCACCAACTCG**	MCM7	4176
**AAATTGATTGCTGAAGTAAA**	SPC25	57405
**AAATTGCGGGATATGCCAGA**	LOC110117498-PIK3R3	110117499
**AAATTGCGGGATATGCCAGA**	PIK3R3	8503
**AAATTGGCGACCAGTCACAC**	RASAL2	9462
**AAATTGGTGAAACACTACAC**	YES1	7525
**AACAACCTACCGAGGCTGGG**	SCRIB	23513
**AACAACTTCCACGCAGCCGG**	FYN	2534
**AACAAGACTGGACCATCCAG**	KSR2	283455
**AACAATAGGCACGTGACCGC**	ONE_NON-GENE_SITE_9	ONE_NON-GENE_SITE_9
**AACAATGGCAAGTTCTACGT**	RAD52	5893
**AACACACCTGTCTACGCTAC**	TGIF1	7050
**AACACCCCGATCCCCCAAGG**	SEC23B	10483
**AACACCGGTGAGGTATATCG**	SNRPD3	6634
**AACACCGTAGTCAGGGCACG**	ESRP1	54845
**AACACTCCAGAGATCCACGG**	CDK6	1021
**AACAGCAATCCAGACCTCTC**	EED	8726
**AACAGCATGAGAACTTGACA**	HDAC9	9734
**AACAGTGAAAGTCGTATCCA**	ONE_NON-GENE_SITE_10	ONE_NON-GENE_SITE_10
**AACAGTTATTGGAATCTCTG**	BRAF	673
**AACATAAGCAACATACCTGC**	NUP37	79023
**AACATACCAGTCATAGGAGA**	EMC3	55831
**AACATCCTCAAGATCAAATG**	WDR70	55100
**AACATGTAAAAACTGTACTT**	PTK2	5747
**AACATTGGTTGTGTGATGAC**	PSMA6	5687
**AACATTTTTCACTTCGCGTG**	ONE_NON-GENE_SITE_11	ONE_NON-GENE_SITE_11
**AACATTTTTCACTTCGCGTG**	INACTIVE_5T_1	INACTIVE_5T_1
**AACCACTGTAGGGTTAGCAT**	FBXO11	80204
**AACCAGGCTCCCCAATGCAA**	CDC23	8697
**AACCAGTTGAGTCATCTCTG**	IRF2	3660
**AACCATTAATGAAATCGGTA**	NUP37	79023
**AACCCCATCATCAGCTACGA**	MLST8	64223
**AACCCCCGATAGCAGCAACA**	BPTF	2186
**AACCGGCCAGAGGACGAGTG**	ERBB2	2064
**AACCGGTATTGACTAACCCA**	EXOC6	54536
**AACCTACAACTGCTACCATG**	RASSF4	83937
**AACCTGCAAACAATCAGGCA**	UBAP1	51271
**AACCTTAGGGAACCTCCACA**	DHFR	1719
**AACGACTCCGACTCCGGGAT**	SRSF2	6427
**AACTACCTTTCTACGGACGT**	ERBB2	2064
**AACTACTGGTGTAATCTACT**	GLOD4	51031
**AACTATGGGACGATGTTAGT**	ONE_NON-GENE_SITE_12	ONE_NON-GENE_SITE_12
**AACTCACTGAGGATGAGGTG**	RPS18	6222
**AACTCATAAGGCTCAAAGCA**	POLR1A	25885
**AACTCATAGCATGTTCCCGG**	ONE_NON-GENE_SITE_13	ONE_NON-GENE_SITE_13
**AACTCCAAAAAAGAGCGGGC**	RCE1	9986
**AACTCCACTGTACCACCGAA**	ONE_NON-GENE_SITE_14	ONE_NON-GENE_SITE_14

**AACTCCGTGGAGTCTCCATG**	RIC8A	60626
**AACTCCTACGAAGATAGTAC**	ONE_NON-GENE_SITE_15	ONE_NON-GENE_SITE_15
**AACTGCTGGATTATCTCACA**	RGL1	23179
**AACTGGCCAAGCTCAACCGG**	RASSF10	644943
**AACTGGTTCGAAAACTTGGT**	CSNK2A2	1459
**AACTTCACAGCATTAGGGAC**	CASP8	841
**AACTTCATCAAGATTGGCGA**	PAK4	10298
**AACTTGCAGCGGGTCTTGAC**	ONE_NON-GENE_SITE_16	ONE_NON-GENE_SITE_16
**AAGAAACAGTTTATTCCCTG**	FAM217B	63939
**AAGAAACCGATGGGACGCCG**	ONE_NON-GENE_SITE_17	ONE_NON-GENE_SITE_17
**AAGAAAGACACAGTAAAGGT**	STAMBP	10617
**AAGAAAGCACTCCCTGACTG**	CCNA1	8900
**AAGAAAGTGATGATAACGAT**	EMC3	55831
**AAGAAATGCTTAGCAAACAG**	GRB2	2885
**AAGAACCAGCTGGACCAGGT**	PFDN5	5204
**AAGAACCTCAAACTAATCCG**	INSRR	3645
**AAGAACGGCAGGGAGTTCCG**	FGFR3	2261
**AAGAACTGCTTCGGCAGGTG**	YAP1	10413
**AAGAAGAGAAGCTTGTCTGT**	RASSF9	9182
**AAGAAGAGCTGGCTAACCTG**	RSPH3	83861
**AAGAAGAGTAGCTGAGGAGC**	CHCHD3	54927
**AAGAAGAGTAGCTGAGGAGC**	CHCHD3P3	646572
**AAGAAGATTAACAACATATG**	PHAX	51808
**AAGAAGTATAGAAAGGTCGC**	INPP5A	3632
**AAGAAGTTTCAGGAACCAAG**	INSR	3643
**AAGAATCCGAAGATCAGAAA**	CHCHD3	54927
**AAGACAACTTCAAGTACAGA**	CNOT2	4848
**AAGACATACTGCATCCATGT**	RGL1	23179
**AAGACCACGAGAGAAAAAGT**	DPM1	8813
**AAGACCCAATAACAATGAGG**	SIRT1	23411
**AAGACGTCATAAGTACCATA**	SHC4	399694
**AAGAGAAAGTTCCTGTCGCG**	CHSY1	22856
**AAGAGAATGATCCATACCAC**	EED	8726
**AAGAGAGAGTGTAGCGCACT**	ONE_NON-GENE_SITE_18	ONE_NON-GENE_SITE_18
**AAGAGATAAAATAGCCTGTT**	NUP37	79023
**AAGAGATTATTGGCAAGGGT**	ACVR1B	91
**AAGAGCAATGCGTCGACCCC**	CBLC	23624
**AAGAGGAGTACAGTGCAATG**	KRAS	3845
**AAGAGGTATCCAGGCCAGCT**	CDC6	990
**AAGAGTTACATTGCCACACA**	LOC100421822	100421822
**AAGAGTTACATTGCCACACA**	LOC442113	442113
**AAGAGTTACATTGCCACACA**	PTPN11	5781
**AAGATAAAGTATGCCTGTAC**	ONE_NON-GENE_SITE_19	ONE_NON-GENE_SITE_19
**AAGATAGAATATTCATCCTG**	RHEB	6009
**AAGATCTGCGAAGACGTTTG**	ITCH	83737
**AAGATGATCTCAATGTCTTG**	VAV1	7409
**AAGATGCTCCGCACGTCGCG**	RASSF5	83593

**AAGCAAATAATTGTAGGTCT**	PHAX	51808
**AAGCAAATCGACCACAGCTA**	CTNNBL1	56259
**AAGCAATCACACATTCATCC**	UBAP2L	9898
**AAGCACCAGATCATGCACCG**	MAP2K2	5605
**AAGCACTAGTTAATCGCCTC**	CCDC6	8030
**AAGCAGAGAGATCTCTCGGA**	CDK2	1017
**AAGCCACACCAATTCTGGAG**	PLXNB1	5364
**AAGCCCAACTACTTACTGCG**	MCM7	4176
**AAGCCCGCGCCTACCTCCCG**	EIF4EBP1	1978
**AAGCCCGGAGCACACTATCC**	DCAF12L2	340578
**AAGCCTCGCTACGTACCTCG**	INPP5A	3632
**AAGCCTCTTCATAAACCCAA**	UBAP1	51271
**AAGCTAGGGACGATCATCAT**	ONE_NON-GENE_SITE_20	ONE_NON-GENE_SITE_20
**AAGCTGCATCCCTTTGATGC**	HDAC11	79885
**AAGCTGGTGGAGATCGCCAC**	ONE_NON-GENE_SITE_21	ONE_NON-GENE_SITE_21
**AAGGAACCGTCCTAGACGGC**	ONE_NON-GENE_SITE_22	ONE_NON-GENE_SITE_22
**AAGGAAGATCCCACTTAACG**	ONE_NON-GENE_SITE_23	ONE_NON-GENE_SITE_23
**AAGGACAGACATCTTACCTG**	PRKCI	5584
**AAGGACAGGAACCAGAACCC**	ZFPM1	161882
**AAGGACCTGTCCACCACAGG**	STAMBP	10617
**AAGGAGCACAAGATCATGGT**	PRKAB2	5565
**AAGGATACACTCTGTCATTG**	ESRP1	54845
**AAGGATACAGAACTCGTCAG**	PFDN5	5204
**AAGGATGAAGCCTCTGATGA**	RASSF6	166824
**AAGGATGTTTGCAAGAACAG**	TBK1	29110
**AAGGCAAATCAGAGTTGCGA**	APC	324
**AAGGCAGACTCGTCGAAGAG**	CHD2	1106
**AAGGCAGCGACGTCTCAACT**	ONE_NON-GENE_SITE_24	ONE_NON-GENE_SITE_24
**AAGGGACAAGGTGGAGTCAT**	PIK3R5	23533
**AAGGGACGACTATAAGAAAG**	CHD2	1106
**AAGGGACTCTGATCATCGAG**	RASSF4	83937
**AAGGGCAATGACATCAGCAG**	PEBP1	5037
**AAGGGCGCAACACTACCATT**	ONE_NON-GENE_SITE_25	ONE_NON-GENE_SITE_25
**AAGGGTTCAAAACGTGACCT**	ONE_NON-GENE_SITE_26	ONE_NON-GENE_SITE_26
**AAGGGTTGAGTACCTGCTAA**	ZNF429	353088
**AAGGTATTCCTATTGCAACG**	ONE_NON-GENE_SITE_27	ONE_NON-GENE_SITE_27
**AAGGTCATACAGCTTAACGG**	ONE_NON-GENE_SITE_28	ONE_NON-GENE_SITE_28
**AAGGTCCTCATTGCAAACAG**	MCCC1	56922
**AAGGTCCTGACACGTCACGT**	E2F1	1869
**AAGGTTACGCTCTTAAGCAG**	ONE_NON-GENE_SITE_29	ONE_NON-GENE_SITE_29
**AAGGTTATGCAAGGTCCCAG**	CTNNB1	1499
**AAGTAAAATAGTATGTCGAG**	EXOC2	55770
**AAGTAACAGGAGCAAATACT**	RPS6KB1	6198
**AAGTAACTGACGATCCGTGA**	ONE_NON-GENE_SITE_30	ONE_NON-GENE_SITE_30
**AAGTACAAAATGAAATAGAC**	SET	6418
**AAGTAGCCTGTATACCAGCA**	UBAP2L	9898
**AAGTAGGAAATCCATAGTGT**	NFKB1	4790

**AAGTATATTCGATCGGAGGT**	ONE_NON-GENE_SITE_31	ONE_NON-GENE_SITE_31
**AAGTGAAATGTAAACGACAT**	SNX14	57231
**AAGTGAACGACATCTCATCT**	RB1	5925
**AAGTGACGTGGAGGATCACT**	DTYMK	1841
**AAGTGAGCAGATCAGAATTG**	CASP8	841
**AAGTGATGAAACCTCTGCGA**	RALBP1	10928
**AAGTGGAATACGTAGACATT**	BIRC3	330
**AAGTGGCAAAGCGAATGTTG**	KDM3B	51780
**AAGTGGGGAATGGAGTACAA**	SNRPF	6636
**AAGTGGGGAATGGAGTACAA**	SNRPFP2	100874411
**AAGTTAAGATAACACCACTC**	CNKSR2	22866
**AAGTTAATGAGATCCCCCTG**	RGL3	57139
**AAGTTAGTTTAAAGTACCTG**	PLCXD3	345557
**AAGTTATGCCAAAGTACTGT**	PRKCI	5584
**AAGTTCATACCTGACCCCCA**	PTGS1	5742
**AAGTTCCAGATTGCAACATG**	MSMO1	6307
**AAGTTCCTGAACATTCCATG**	POLR1A	25885
**AAGTTGAAAAGACATCACTG**	ROS1	6098
**AAGTTGGTCAGTCTCGCACT**	STAM	8027
**AATAAAGTGACGCTTATAGG**	ONE_NON-GENE_SITE_32	ONE_NON-GENE_SITE_32
**AATAACACTCTACCCTCATG**	PLCE1	51196
**AATAATCAGGCATACCATCT**	EZH2	2146
**AATACAAACCTGGTCTACGT**	ITCH	83737
**AATACATTTGCACTTGTGAT**	RPS6KA6	27330
**AATACCTGACTTCAGGTCAA**	SIRT1	23411
**AATACGCGTGCCTCCTCACA**	CHSY1	22856
**AATAGTGCAACAGACTCGGA**	DUSP6	1848
**AATATACAAAGCAACTGTCA**	JAK2	3717
**AATATCTACAGTCCGATATG**	ONE_NON-GENE_SITE_33	ONE_NON-GENE_SITE_33
**AATATGCACAGAATGCGTTG**	ESRP1	54845
**AATATGTCATCTACATCGAG**	RPL26L1	51121
**AATCAACCCACAGCTGCACA**	TP53	7157
**AATCAAGAACTTATCTACGA**	TBK1	29110
**AATCAATGGGCCATCTTGGA**	MRAS	22808
**AATCACGAAAGATGAGTGGA**	GID8	54994
**AATCACGCTCGCTTCTAGCC**	GLOD4	51031
**AATCAGATCGTGCCCAAGAC**	MAGEA12	4111
**AATCAGTTACCTAACGGACA**	PTK2	5747
**AATCATGTAGTACCAATACT**	CERS2	29956
**AATCCAAGCCACACCACCAA**	TMEM263	90488
**AATCCCAGCCAGCGGATTGG**	RPS6KB2	6199
**AATCCTGAATATCAGAAGCT**	ATXN3L	92552
**AATCCTGAATATCAGAAGCT**	GS1-600G8.3	100093698
**AATCCTTGCGTAGCCCGAAG**	JAK3	3718
**AATCGATGGATTGCGCCATG**	LOC112268419	112268419
**AATCGATGGATTGCGCCATG**	STAMBP	10617
**AATCGTCGATGTGCGCAAGG**	KSR2	283455

**AATCTAAAGACATACTCGTC**	ONE_NON-GENE_SITE_34	ONE_NON-GENE_SITE_34
**AATCTCCCTCAGAAAAAACG**	E2F3	1871
**AATCTCCGTATGTTTCAGGT**	MRAS	22808
**AATCTTCACAAGACTTTGCA**	CHMP3	51652
**AATCTTCACAAGACTTTGCA**	RNF103-CHMP3	100526767
**AATGAAGAGTACAACCTCAG**	JAK2	3717
**AATGAGACTCACGGGCATGT**	VCP	7415
**AATGAGGATACCCATAAACA**	EXOC4	60412
**AATGCACTTCTATGAGCTTG**	RNF103	7844
**AATGCACTTCTATGAGCTTG**	RNF103-CHMP3	100526767
**AATGCCCAGCCCTTACTACA**	GINS2	51659
**AATGCTCCCTCAACCCTGAG**	PRKCB	5579
**AATGGAACCCCCGTCAACAG**	PLCE1	51196
**AATGGAAGAAGACCTAATCG**	HDAC6	10013
**AATGGCCTCGTAATCATACA**	HCK	3055
**AATGGCTGAATTAAAAGCCA**	CCNA2	890
**AATGGGCAAAGTGTATCGAC**	ATP2A2	488
**AATGGGCTTTAGTAGAAGCC**	BIRC3	330
**AATGGTCTTAAAGTAGCGCA**	RAPGEF1	2889
**AATGGTGTCAAAACAACACG**	ASXL3	80816
**AATGTAACCATCAAAGCCTG**	RASAL2	9462
**AATGTGCCATAAAATCCTCT**	SPTLC1	10558
**AATGTTCCATTTCTACTGGT**	RALA	5898
**AATTACGGTCTTCCCGGCGT**	TK1	7083
**AATTACTAAACTTCAGAACC**	PPP1R7	5510
**AATTACTGTATCGAATGACT**	METTL5	29081
**AATTATGGCAGGTTGTTACG**	BRCA2	675
**AATTCAATCTAAATGCTCCA**	GPRC6A	222545
**AATTCGAAGCAGAGTCCTCA**	EMC3	55831
**AATTCTGGAACGTGCCATTG**	UBAP1	51271
**AATTGAAGTGGAGCCAACGT**	KSR2	283455
**AATTGCTCTCCTTCTAGATG**	MDM2	4193
**AATTGGTGGAGTATTGATTG**	BPTF	2186
**AATTGGTGGATATATTTACC**	PEX3	8504
**AATTGTCCGGGGTCAAACAG**	NOX1	27035
**AATTTAAGGAACGTTGGCAA**	PDCD6IP	10015
**AATTTCTCCATCCTGAGTCA**	YAP1	10413
**AATTTGATAAGATGGACGTG**	MCM6	4175
**ACAAAAGGGGCATTACTCGT**	ONE_NON-GENE_SITE_35	ONE_NON-GENE_SITE_35
**ACAAACAATGACATGAACCA**	BRIP1	83990
**ACAAACACGCCAAATGCACC**	EED	8726
**ACAAACTCGAAGGACAGCTG**	WDR70	55100
**ACAAACTCGTCTACTTCAGG**	CCNA1	8900
**ACAAAGGACCCCGGCGAAAG**	ONE_NON-GENE_SITE_36	ONE_NON-GENE_SITE_36
**ACAAATGATTAAGTTGACAC**	BRAF	673
**ACAAATTGATAATATAGGAG**	AKT3	10000
**ACAACACAAACAGTCCCAAG**	ITCH	83737

**ACAACACACGAATTACACAA**	ITCH	83737
**ACAACCCACCTGTCGGACGA**	TTC7A	57217
**ACAACCCAGTACATGAATGA**	HDAC6	10013
**ACAACGTTGGTGATTTGGTG**	NSD2	7468
**ACAACTCAATCCGTAATGCA**	ONE_NON-GENE_SITE_37	ONE_NON-GENE_SITE_37
**ACAACTGACAGATGCCTATG**	CHURC1-FNTB	100529261
**ACAACTGACAGATGCCTATG**	FNTB	2342
**ACAAGAAATACCGTGCCCTG**	RPS8	6202
**ACAAGAAATACCGTGCCCTG**	RPS8P8	728553
**ACAAGATTGTCCTGAAGAAG**	UBL5	59286
**ACAAGATTGTCCTGAAGAAG**	UBL5P2	100287215
**ACAAGCCCCTGTATCAGATG**	TTC7A	57217
**ACAAGGGTGGAAACAGCGAC**	ASXL3	80816
**ACAAGTGTGACGATCTGGAC**	NEDD9	4739
**ACAAGTGTGATTTGCCAACA**	NRAS	4893
**ACAATATGACCATACTCAAT**	ITFG1	81533
**ACAATCTGGATCGACTCCAC**	VPS25	84313
**ACAATGCAGTCGAGACACTG**	POLR2H	5437
**ACAATGCAGTGTATAAAGGT**	EED	8726
**ACAATGTCATCACTAATGAG**	POLR2C	5432
**ACAATGTCCCTGCTCATCAG**	RAPGEF2	9693
**ACACAACAGACCTAAAGGAA**	IFIT5	24138
**ACACAAGCTGCAGACGGCCA**	CD1E	913
**ACACACGCACCCGCAACTGG**	CCND3	896
**ACACACTGGTCAGATACCAC**	RASGRF2	5924
**ACACAGCCCCCGATCTCGGG**	SCRIB	23513
**ACACAGTTCAGGCTTCTGAG**	E2F7	144455
**ACACATTGCACCGTAACTAT**	ONE_NON-GENE_SITE_38	ONE_NON-GENE_SITE_38
**ACACCAGAGCGTAGGATCGG**	INSR	3643
**ACACCGGTGAGGTATATCGG**	SNRPD3	6634
**ACACCTCTTTCAACTCCCGT**	DAB2IP	153090
**ACACGACAATATATAACATG**	SMAD5	4090
**ACACGGCTCCATCCACGATG**	MED12	9968
**ACACGTCCTCATACTCACCA**	TBCB	1155
**ACACTACAGAATTAAAAGAC**	FRK	2444
**ACACTGACCTGCATTGGTAC**	RASA1	5921
**ACACTTACGAGTATTCAGAA**	SNX13	23161
**ACACTTCGAACATTTCACCA**	NUP37	79023
**ACAGAAATGCTTGACTTCTG**	RHOA	387
**ACAGAACGAATGTCCGCAAG**	LONRF1	91694
**ACAGAAGCGACTCTACTACG**	LIPK	643414
**ACAGAAGGTATAGCCACCAT**	CHURC1-FNTB	100529261
**ACAGAAGGTATAGCCACCAT**	FNTB	2342
**ACAGAATTCTGGGTACTCGG**	DUSP4	1846
**ACAGACAGATCGGTAAGCCT**	FYN	2534
**ACAGACAGCAAGCGGAACCA**	E2F7	144455
**ACAGACCCTTACGAATACTG**	ONE_NON-GENE_SITE_39	ONE_NON-GENE_SITE_39

**ACAGAGCCACACTCACCGGG**	CRYGB	1419
**ACAGAGGAGGAGCTTACACT**	NRTN	4902
**ACAGAGTCCGTACATATTCC**	CRKL	1399
**ACAGATAAGAGTCCATGTCA**	KLHDC3	116138
**ACAGATCAGGCTTCAGCTGT**	RAB6A	5870
**ACAGATGGATAATCAAATAG**	CCT4	10575
**ACAGCAAGCCAGTGAACCTG**	RAC2	5880
**ACAGCAAGGGAATCATGCAC**	CSNK2A2	1459
**ACAGCACACCGTACCGACAG**	RPS8	6202
**ACAGCACCAGTTCAAGAAGA**	IFITM1	8519
**ACAGCAGATGTAGATACGAC**	ONE_NON-GENE_SITE_40	ONE_NON-GENE_SITE_40
**ACAGCATCCTCACACCAACT**	UBAP1	51271
**ACAGCCCCCTACAGATCTGT**	PRPF38B	55119
**ACAGCCTGCCAAAAACCCTG**	E2F7	144455
**ACAGCGTGGAAGCACACCGA**	ONE_NON-GENE_SITE_41	ONE_NON-GENE_SITE_41
**ACAGCTCAAGTCGGCCTGCA**	PEA15	8682
**ACAGCTGCTCGGTCAGAGAG**	CCNA1	8900
**ACAGCTGGTCCACACTGCAA**	TSC2	7249
**ACAGGCATGAAGTTATTGCT**	MCCC1	56922
**ACAGGCTTATATCGCTTCAG**	ONE_NON-GENE_SITE_42	ONE_NON-GENE_SITE_42
**ACAGGCTTTACATGGCGAGG**	ATL2	64225
**ACAGGTGTCACCTTGAAGGT**	MDM2	4193
**ACAGGTTTACCACCTCGCCA**	ARID5B	84159
**ACAGTAAAAGGCTGTCGAGA**	CNOT2	4848
**ACAGTAACCCTAATAACCAA**	RIC1	57589
**ACAGTAAGAGCATAATGGGG**	MON2	23041
**ACAGTACGTCATGCTCTCGG**	TTC7A	57217
**ACAGTACTTGGTGTACAATG**	EXOC8	149371
**ACAGTGACAGCCTCTGAACG**	RALGAPA2	57186
**ACAGTGGGTACATGCGCATG**	IRS2	8660
**ACAGTGTATAAACTCCCACA**	TBK1	29110
**ACATAAAAATTCCACAAGAA**	EXOC5	10640
**ACATAAGATTGAGACCTGGA**	PRKAG3	53632
**ACATACACAATTATCGGCTA**	ONE_NON-GENE_SITE_43	ONE_NON-GENE_SITE_43
**ACATACCAAGAGAATCACCC**	DPM2	8818
**ACATAGTGAATATTGATCCG**	RAPGEF2	9693
**ACATATCATAAAAGAGAACA**	PDPK1	5170
**ACATCACCCAGTCCCGAACA**	ETS1	2113
**ACATCACCTGGAAAGAAGTC**	HACD2	201562
**ACATCAGCATATCCGTAGGC**	ONE_NON-GENE_SITE_44	ONE_NON-GENE_SITE_44
**ACATCATACGCTACACACGA**	TCF7L2	6934
**ACATCATACTCCTGGGCCAC**	NPRL2	10641
**ACATCGACTGCTGGACAATG**	MED12	9968
**ACATCGCTTGCACACTGAGT**	RPS6KA2	6196
**ACATCGGGGACAGTAGCTCT**	SPNS1	83985
**ACATCTCTTGCAAACAGAGT**	RPS6KA3	6197
**ACATCTGGGGCTCTATGAGG**	FAXDC2	10826

**ACATCTTCAGTATTAGTGAG**	POLR3G	10622
**ACATGAACTCAGTGCTCATG**	CHMP3	51652
**ACATGAACTCAGTGCTCATG**	RNF103-CHMP3	100526767
**ACATGAATGTAATATAGATG**	RB1	5925
**ACATGAGAGAAGAGAACTTG**	CCDC6	8030
**ACATGATGGAGGACGTCATG**	VKORC1L1	154807
**ACATGCTGACTGTCGATGAA**	DPM2	8818
**ACATGCTGGATATGTCGATG**	RASGRP3	25780
**ACATGGGCAGCATACAGGCC**	RCE1	9986
**ACATTTGAAGTAGGACACCG**	CCND1	595
**ACATTTGTCAACCATCCACA**	SET	6418
**ACCAAAATCTGTGTTGACTG**	NFE2L2	4780
**ACCAAACGTGTGTTCTGGAA**	TYMS	7298
**ACCAAGGCCCCTCTACGTCC**	TBC1D3B	414059
**ACCAAGGCCTGTCGGTCACG**	CCND3	896
**ACCAATACTCGGTCAAAGAG**	HSPE1	3336
**ACCAATACTCGGTCAAAGAG**	HSPE1-MOB4	100529241
**ACCAATACTCGGTCAAAGAG**	HSPE1P2	326300
**ACCAATACTCGGTCAAAGAG**	HSPE1P5	100506000
**ACCACAGATAAAGTCAAACA**	LOC442113	442113
**ACCACAGATAAAGTCAAACA**	PTPN11	5781
**ACCACATAGAGAAAGATCAG**	SEC23B	10483
**ACCAGAATTTGTAGACTGCG**	XIAP	331
**ACCAGAGCAGTAACCCCCGA**	KLHDC3	116138
**ACCAGCAAATGAACAATCCC**	CNOT2	4848
**ACCAGCACCGTGATACTACG**	MCM7	4176
**ACCAGCAGCCAATGCAGCCG**	B3GAT3	26229
**ACCAGCGGGCAGTCGTCGCC**	ANAPC11	51529
**ACCAGCTGCACACCATGGGT**	SREBF1	6720
**ACCAGCTTGAATTTCCTCAG**	RAB6A	5870
**ACCAGCTTGAATTTCCTCAG**	RAB6C	84084
**ACCAGCTTGAATTTCCTCAG**	RAB6C-AS1	100131320
**ACCAGCTTGAATTTCCTCAG**	RAB6D	150786
**ACCAGGCACACAAGAGACTG**	MYB	4602
**ACCAGGCTCTTGGGACAACG**	RASGRP4	115727
**ACCAGGTACCAGATCCACAC**	ARSI	340075
**ACCAGGTACGCCGTGGCCAG**	HACD2	201562
**ACCAGTTCTGGGTTAAACTG**	HDAC6	10013
**ACCAGTTGGACAGTGCTCCA**	ALK	238
**ACCATAATCAGCCCATCCCG**	C12orf49	79794
**ACCATACCCAAATTATTCCG**	USP7	7874
**ACCATATCAAATTCACACAC**	PIK3CA	5290
**ACCATCAATTAAGTTCCCCG**	ONE_NON-GENE_SITE_45	ONE_NON-GENE_SITE_45
**ACCATGCATCCCCAAAACCA**	SPRR1A	6698
**ACCATGCATCCCCAAAACCA**	SPRR1B	6699
**ACCATTGAAAAGTAAGCTTG**	LOC653406	653406
**ACCATTGAAAAGTAAGCTTG**	LOC728519	728519

**ACCATTGAAAAGTAAGCTTG**	NAIP	4671
**ACCCAACCGTGTGACCAAAG**	FGFR1	2260
**ACCCACCTGTCGTCTCGTCT**	ONE_NON-GENE_SITE_46	ONE_NON-GENE_SITE_46
**ACCCAGCTGTCAAAGAGAAG**	SCD	6319
**ACCCCAGACTCACAACACAA**	PRKCG	5582
**ACCCGACCACCTGTTGTACA**	AUP1	550
**ACCCGATGTTGAAAAAAGGA**	TM9SF3	56889
**ACCCGGAAGTAATGAGTGTG**	BIRC3	330
**ACCCGGGGTAGCTGCATGTG**	MYB	4602
**ACCCTTAGAAACTACACAGC**	BRIP1	83990
**ACCCTTAGTCAGCGAGACAT**	BSCL2	26580
**ACCCTTAGTCAGCGAGACAT**	HNRNPUL2-BSCL2	100534595
**ACCGAAATCACCGGAATACT**	LIPK	643414
**ACCGAATATTTGTTCTGTCG**	DPM1	8813
**ACCGAGATAGAGACCACAAG**	DHX8	1659
**ACCGCACAGCCAGAGCCTCG**	CNKSR1	10256
**ACCGGATTATATATCACCTG**	ROCK2	9475
**ACCGGCAGCGTCAAACACCC**	TFDP1	7027
**ACCTACAACTTGTACTATGA**	RASSF2	9770
**ACCTAGCCAGTGATGGACCA**	GAREM1	64762
**ACCTCAACTCGGTGGTGCTG**	DUSP5	1847
**ACCTCAAGGAAGCTTATGAC**	NPRL3	8131
**ACCTCGCGGATGGTGGAGAG**	CDK6	1021
**ACCTGAAGGCCCACTCACGC**	PLAGL2	5326
**ACCTGATAGCCCGAGTAGTG**	PHAX	51808
**ACCTGGAGCACAATGCACGT**	HDAC3	8841
**ACCTGTGCCACATACTGGGG**	MARCH2	51257
**ACCTTAACCATTGCAAACCA**	NF1	4763
**ACCTTCAAGATCTGGACATA**	FGFR1	2260
**ACCTTGACAACAGTCGTATG**	ONE_NON-GENE_SITE_47	ONE_NON-GENE_SITE_47
**ACCTTGAGCACGGTAACGTA**	FGFR3	2261
**ACCTTTAATAGGCACGTCCT**	ONE_NON-GENE_SITE_48	ONE_NON-GENE_SITE_48
**ACCTTTGCATCATCCAATAG**	SREBF2	6721
**ACGAATTGCAGTATGAACAG**	EXOC8	149371
**ACGAGATCTTAACATAGTAG**	ATL2	64225
**ACGAGCACGAACACACGGTG**	TM9SF3	56889
**ACGAGCCAAAACTGATCCCG**	TGIF1	7050
**ACGAGTGTAATAGCTCACGT**	PTGS1	5742
**ACGATTATGGAATATCCAGA**	EED	8726
**ACGCAGAAAAAGCTCCGCGC**	ONE_NON-GENE_SITE_49	ONE_NON-GENE_SITE_49
**ACGCCAGTTGCATGAGCCAG**	RASSF4	83937
**ACGCCTGCAGGCACTAAGTG**	RASSF7	8045
**ACGCGGACCTACGACCGCGA**	NUDT4	11163
**ACGCGGACCTACGACCGCGA**	NUDT4B	440672
**ACGCGGACCTACGACCGCGA**	NUDT4P2	170688
**ACGCTGGCTTGTCTCAACCT**	HCFC1	3054
**ACGGCCTCGACCCCTCACAG**	MARCH2	51257

**ACGGCTTGAGGGGTTGACCC**	E2F1	1869
**ACGGGGACAGACCAGGTGGT**	DPM2	8818
**ACGGGTGGCCCTCAACACGC**	QPRT	23475
**ACGGTCCAGACTTTCCAAAG**	UBTFL1	642623
**ACGGTGTTGGAGAAGTCCAG**	NLRC3	197358
**ACGTAGAGGATCTGCGCGGT**	FUNDC2	65991
**ACGTCACAATCCAAGACCAT**	TBC1D3B	414059
**ACGTCGCGGAAGTCGATAGG**	SOX9	6662
**ACGTCGTCCTTATGCAAGGG**	MMP9	4318
**ACGTGACAGAAAGTCAATGT**	ATP2A2	488
**ACGTGCTGTTGTCATCAGAT**	UBTFL1	642623
**ACGTGTCGTAGACCACACCT**	HDAC5	10014
**ACGTTCACCCGTCACTAGTG**	HDAC5	10014
**ACTAACAATTAGCGCAGTTG**	ONE_NON-GENE_SITE_50	ONE_NON-GENE_SITE_50
**ACTAACAGGAAAGCCAGTGA**	SNRPF	6636
**ACTAACATGTATGAAGGTGT**	PFDN1	5201
**ACTAACCTGATCGTAGAGTG**	DUSP1	1843
**ACTAACGGCGCATCACCTAA**	ONE_NON-GENE_SITE_51	ONE_NON-GENE_SITE_51
**ACTAATGGGACATGAGTACA**	CDC23	8697
**ACTAATTGTTGAAGGAATGC**	RASSF6	166824
**ACTAATTTAGGATCAACCAC**	BRAF	673
**ACTACAGCCATGTCGAGCGT**	HCFC1	3054
**ACTACAGGATGAGATGTACG**	INTS6	26512
**ACTACCCGACCTTAGCGACC**	ONE_NON-GENE_SITE_52	ONE_NON-GENE_SITE_52
**ACTACTCGGAAGACTTGCCG**	MMP9	4318
**ACTAGAATAGTGCTGCACCA**	BLK	640
**ACTAGCTGCCCGATTCCCGG**	EIF4EBP3	8637
**ACTAGGATGACTGTAAAGGG**	RASGRF1	5923
**ACTATAGTATTCTTCAGAGG**	DDX46	9879
**ACTATGGCAACTACCGGCGT**	RASGRP2	10235
**ACTATTGCACCACGCCCGGG**	EIF4EBP2	1979
**ACTCACCATCAACACCCCAT**	RPS6KA1	6195
**ACTCACCGATCATGGAGAGC**	SHC3	53358
**ACTCAGGGTAGAGAATTTGC**	PREB	10113
**ACTCCAGGGATGAACTCAAG**	MCM5	4174
**ACTCCATGACTCACGATACT**	ONE_NON-GENE_SITE_53	ONE_NON-GENE_SITE_53
**ACTCCCATTATAGGTCTGTG**	CCND2	894
**ACTCCGTCAGGAAGCCGGTG**	MAPK3	5595
**ACTCGCTACAAAGGAAATGG**	DPM1	8813
**ACTCGGTTTGGAAACGCAGA**	MMP9	4318
**ACTCGTCATCAATCCCGTCT**	HDAC1	3065
**ACTCTGTAGCCACTATCTGG**	CHURC1-FNTB	100529261
**ACTCTGTAGCCACTATCTGG**	FNTB	2342
**ACTCTTCTCTCACAGACTTG**	NSD2	7468
**ACTGAGACCGCCTTTCACAT**	EXOC2	55770
**ACTGAGCAATCTGGAAGCGA**	TK1	7083
**ACTGAGGCGGAATCCATAGC**	PTK2	5747

**ACTGATACACTCCAAGCGGT**	FOS	2353
**ACTGCAGTGTACCTGACGTC**	RASA3	22821
**ACTGCCGTGTACTTCCTGCG**	DUSP2	1844
**ACTGCCTGGTGTTCAACTAC**	LONRF1	91694
**ACTGCGGTCAGTGCAGCGAG**	PRKCZ	5590
**ACTGCTATCCTGAATAGACA**	BPTF	2186
**ACTGCTGCAGAACACACCCG**	CYTH2	9266
**ACTGGAAGCACGAATGACAG**	NFKB1	4790
**ACTGGAGCCACATAATTGCT**	FYN	2534
**ACTGGAGCCTTATTGGAGGT**	HACD2	201562
**ACTGGATGGATCGATCCACA**	ONE_NON-GENE_SITE_54	ONE_NON-GENE_SITE_54
**ACTGGCACCAGCATAAGACG**	SOS2	6655
**ACTGGGAAATGGGCAGTTTG**	FYN	2534
**ACTGGGACAGGCCCTTCCTG**	RPS13	6207
**ACTGGGAGCTGAAAACTGAT**	POTENTIALLY_ACTIVE_1	POTENTIALLY_ACTIVE_1
**ACTGGGCCAGAGACTCGTGG**	PIK3R2	5296
**ACTGGTGGCCCTGGTAGTTG**	CRYGB	1419
**ACTGGTTTCAAATGTGTGAC**	UGCG	7357
**ACTGTAGTATAATCGCCGGG**	RALGAPA2	57186
**ACTGTCGCATTCAGAAGATC**	NLRC3	197358
**ACTGTCTCTGGTAGAGATGG**	MAP2K1	5604
**ACTGTGAGGTCGGTGTTCCG**	PIK3CG	5294
**ACTGTTGAACTGGGTGACAG**	RALGDS	5900
**ACTTACAGGATCCCCTCCAG**	SHC4	399694
**ACTTACAGTTTGATAATATG**	PRKAA2	5563
**ACTTACATGGGGTCGTCATC**	BIRC5	332
**ACTTACATTGGGAACATGGT**	WDR70	55100
**ACTTACCATCAGCTACACTA**	RALGAPA2	57186
**ACTTACCCCAACTTCTGGCG**	INPP4B	8821
**ACTTACCCGTACCAGTAGCG**	HDAC4	9759
**ACTTACCGAACGCGAAGAAG**	BSCL2	26580
**ACTTACCGAACGCGAAGAAG**	HNRNPUL2-BSCL2	100534595
**ACTTACCTGATTGCCCAGCA**	FLVCR1	28982
**ACTTACGCTTCATCAATGTT**	UBE2H	7328
**ACTTCTCAAACCACAGAGTG**	SOS1	6654
**ACTTCTCAGAATGACACGCT**	RASSF6	166824
**ACTTCTCTAGTAGTTAATCG**	ONE_NON-GENE_SITE_55	ONE_NON-GENE_SITE_55
**ACTTGACGAACTTGTCCACG**	INPP5A	3632
**ACTTGACTCAGGTCACCTCA**	ACVR1B	91
**ACTTGCAACACTCTTACCCA**	TFDP2	7029
**ACTTGCAGCCCAGAATATTG**	UAP1	6675
**ACTTGGCAAGAACGGACGGC**	NPRL3	8131
**ACTTGGCCCTCGGGTCCGTG**	E2F2	1870
**ACTTGTCACCTCTTAATCAG**	PRKAB2	5565
**ACTTTAGCACCTTATTCCTT**	RPL31	6160
**ACTTTATAAGCGTTGTCACA**	RASSF3	283349
**ACTTTGATAAAGTGACAGGT**	CASP7	840

**ACTTTGGAACAGGACCAACT**	MAP2K1	5604
**ACTTTGGGGTGGCAGGGCTC**	SPRR1A	6698
**ACTTTGGGGTGGCAGGGCTC**	SPRR1B	6699
**ACTTTGTGATTATGAGCTCG**	PRKCE	5581
**AGAAAACAAGTGGTTATAGA**	NRAS	4893
**AGAAAAGGGATCTAATGCTG**	PREX2	80243
**AGAAAATGTAAGGTCATCTG**	BUB1	699
**AGAAACCCAGCATCTGAGTG**	SLCO2B1	11309
**AGAAACCGTCTGGAGAATGA**	RANGAP1	5905
**AGAAACCTACAACTCATGGA**	BRCA1	672
**AGAAACGCGCCGTCTGGAAG**	GLOD4	51031
**AGAAAGGGCTGCCAGGCAGG**	BIRC5	332
**AGAAAGTGCCTCCAGCACGT**	MAF1	84232
**AGAAATAAGCTGAAAAAACA**	LONRF1	91694
**AGAAATACGGTCCGAAACGT**	MYB	4602
**AGAAATCTAGTGTCTCCAAG**	UBTFL1	642623
**AGAAATGCTGCTTCTCGTCT**	RPS6KB1	6198
**AGAAATTTATCAGTACCTTA**	CCNA1	8900
**AGAACAGACAAAACAGTCAT**	PSMB1	5689
**AGAACAGCTGGCTAGACTGG**	CHCHD3	54927
**AGAACCAAGCGCCCAATATG**	ONE_NON-GENE_SITE_56	ONE_NON-GENE_SITE_56
**AGAACCCGGCGTGAGCACAG**	GRB10	2887
**AGAACCGACCCGGCAATCCG**	ONE_NON-GENE_SITE_57	ONE_NON-GENE_SITE_57
**AGAACCTGCCGCTCATCGTG**	DPM1	8813
**AGAAGAAATTGATTGCACAG**	FERMT1	55612
**AGAAGACCGGTCCTGATGCT**	RPS6KA6	27330
**AGAAGCAGACGAGTCGCTCC**	YAE1D1	57002
**AGAAGCCGCATCATATGATG**	POLR1A	25885
**AGAAGGATTTAAATATTGAG**	PRKAA1	5562
**AGAAGGCTGTCTCCGTGTAC**	C9orf116	138162
**AGAAGGGACCTCTCTTCGAG**	MCM6	4175
**AGAAGGTGAAGAATATATGC**	POLR3G	10622
**AGAAGTTTGTAGACACACCG**	MCCC1	56922
**AGAATAAGTCATGCTCTGAG**	RASAL2	9462
**AGAATACATAAGTAGCCGCA**	RAD52	5893
**AGAATAGTGGAACAATGACA**	ICMT	23463
**AGAATATACTAGAACATCCC**	LOC110117498-PIK3R3	110117499
**AGAATATACTAGAACATCCC**	PIK3R3	8503
**AGAATATAGAGAATGACACC**	CAMK2D	817
**AGAATGGGCTGATATAGATC**	FNTA	2339
**AGAATTTGCGACACAGACGT**	RASGRF2	5924
**AGACAACTACTTCCGAAGTG**	RALA	5898
**AGACAAGACATGTACCTGCT**	SPRY2	10253
**AGACAAGTTGAAGATGACCT**	RASSF3	283349
**AGACAATCACGTCAAAACCC**	CASP7	840
**AGACACTTATACTATGAAAG**	MDM2	4193
**AGACATCAGCCATCATGTGA**	RPS6KA2	6196

**AGACATTCTGTAGAGTTCAC**	BIRC2	329
**AGACATTGACCTCACCAAGA**	RPS18	6222
**AGACATTGACCTCACCAAGA**	RPS18P12	388339
**AGACATTGACCTCACCAAGA**	RPS18P13	100271364
**AGACCACGAAGCCGCACTTG**	PLEKHF1	79156
**AGACCATATCAAAGTGACCC**	CBL	867
**AGACCGTTGGACTCACGAGT**	ERBB2	2064
**AGACTGCAGTGCCGAAACGA**	RGL1	23179
**AGACTGGTCCCTGAGCGACG**	PIK3R2	5296
**AGACTTCGGGCTGTGCAAGG**	AKT1	207
**AGACTTCTCCACGAACACAA**	RAF1	5894
**AGACTTCTGGCAAGATAAGA**	LOC100420464	100420464
**AGACTTCTGGCAAGATAAGA**	SAV1	60485
**AGACTTTGTCCACAGTGTGC**	BIRC7	79444
**AGAGAAAAGGAGAATCCGAA**	FOS	2353
**AGAGAAACCACGCACGGCAA**	ONE_NON-GENE_SITE_58	ONE_NON-GENE_SITE_58
**AGAGAATAAAATTCTTCACG**	CNKSR2	22866
**AGAGACAATAGAACCAGAGC**	STAM	8027
**AGAGACATGCTGGACGCCCT**	DUSP4	1846
**AGAGACCAGCCCGCTGACCG**	CCND2	894
**AGAGACCAGCCCGCTGACCG**	CCND2-AS1	103752584
**AGAGACTATAGTCGGCGACG**	UBAP2L	9898
**AGAGAGAGAAAATCATCATC**	SLBP	7884
**AGAGAGAGTGAAACAACTAA**	YES1	7525
**AGAGAGTCGCCTGCAACAAG**	METTL5	29081
**AGAGATCATGAAACACCTGA**	VPS45	11311
**AGAGATTCTGTGCTTCCACG**	AUP1	550
**AGAGCAAAAGATTTGTACAC**	PTK2	5747
**AGAGCCTTCCATAAAAACGA**	FBXO11	80204
**AGAGCGAGGCCACGCACCAG**	RAD52	5893
**AGAGCTCCTTAGTCTCCTCA**	TRIM64	120146
**AGAGCTCCTTAGTCTCCTCA**	TRIM64B	642446
**AGAGCTCCTTAGTCTCCTCA**	TRIM64C	646754
**AGAGCTGGCTGATATTAAGC**	DNAJC9	23234
**AGAGCTGGCTGATATTAAGC**	LOC100421490	100421490
**AGAGGACAACATGAACTGCC**	SNRPD3	6634
**AGAGGAGTACAGTGCAATGA**	KRAS	3845
**AGAGGCAGAGCGCCAGTGAG**	MESP1	55897
**AGAGGCCACGAACATGCAAG**	CCND1	595
**AGAGGCTGGAAAAGACTCTG**	RGL2	5863
**AGAGGGATACTCACACTCCA**	RPL31	6160
**AGAGGGGCGCCGGGACACAG**	RASSF5	83593
**AGAGGGTAGCTCATACCCAT**	PLAGL2	5326
**AGAGTAAGCAGGAATAGCAC**	SPTLC1	10558
**AGAGTAATAAGGCGAGCCGT**	ONE_NON-GENE_SITE_59	ONE_NON-GENE_SITE_59
**AGAGTATGGAAGACTTGCGA**	ATL2	64225
**AGAGTATGTATCCCTGGTCA**	HDAC10	83933

**AGAGTCTCCAGCGCCGGTGC**	MESP1	55897
**AGAGTCTGAGTTAATCGATC**	ONE_NON-GENE_SITE_60	ONE_NON-GENE_SITE_60
**AGAGTGCATCGACCCCTCGG**	MYC	4609
**AGAGTGGAATCACACTAACG**	ONE_NON-GENE_SITE_61	ONE_NON-GENE_SITE_61
**AGAGTTGAAGCTAAACGTCC**	ONE_NON-GENE_SITE_62	ONE_NON-GENE_SITE_62
**AGAGTTGTCTACAGACATAG**	UNG	7374
**AGATAACAGAAGTACAACCA**	ATP2A2	488
**AGATAACTACTTTCGGAGTG**	RALB	5899
**AGATACAGAGATCATGACTT**	PFDN1	5201
**AGATACATCAGCTGCCAACC**	FAM20B	9917
**AGATACCTGAGCGGTGGCCA**	DHFR	1719
**AGATAGGCTGGAGAGAATGG**	NOX1	27035
**AGATATCAAGCAAAGACATG**	LOC653406	653406
**AGATATCAAGCAAAGACATG**	LOC728519	728519
**AGATATCAAGCAAAGACATG**	NAIP	4671
**AGATATCACACATTACAGTG**	RASA2	5922
**AGATATTCACCATTATAGGT**	KRAS	3845
**AGATCCGAACATTTGAACCG**	VPS39	23339
**AGATCCTAAACAAGACGACT**	RAPGEF1	2889
**AGATCGACCCTAAGACGACA**	PRKCH	5583
**AGATCTAGAACAGCTCAAGT**	PEA15	8682
**AGATCTGTGATTGAACAGGG**	PDCD6IP	10015
**AGATCTGTGATTGAACAGGG**	PDCD6IPP1	100861430
**AGATCTGTGATTGAACAGGG**	PDCD6IPP2	646278
**AGATCTTGAACCGTCTGTGG**	RAD52	5893
**AGATGAAGCTCATAACATCG**	BRIP1	83990
**AGATGAATCTACCAGCATAG**	CCNA1	8900
**AGATGAATGAGCTTCCACTG**	SLC22A25	387601
**AGATGAGGGACAGTTTGGTT**	CBL	867
**AGATGATCCCCCTCTCGTGC**	PRKCZ	5590
**AGATGATGACTATGGACGAT**	GPRC6A	222545
**AGATGGCCATACACTAGGGT**	ONE_NON-GENE_SITE_63	ONE_NON-GENE_SITE_63
**AGATGGCTACATGAACATGC**	SNRPF	6636
**AGATGTATGATCCCAAAACG**	USP7	7874
**AGATGTTGTCCCCTTCGAAG**	STK11	6794
**AGATTACGGATGCTGCATTG**	EXOSC9	5393
**AGCAACTCAGAACTTCAAAG**	TTC7A	57217
**AGCAACTCTCCCAATACCTA**	SAV1	60485
**AGCAAGAATGCAGCCCAACA**	PRKCI	5584
**AGCAATGATGACTACTGATG**	CCT4	10575
**AGCACACAGAAACTATTCAG**	JAK2	3717
**AGCACACCCATCATCCTGGT**	RAC2	5880
**AGCACAGTAGAACTAAGGGT**	BRCA2	675
**AGCACCACAGAGACCAGCTG**	RSPH3	83861
**AGCACCAGGCTCCTCCAAGT**	HDAC5	10014
**AGCAGAACGGAATATTCTGG**	RPS6KB1	6198
**AGCAGAACTGATTAAAACAG**	PDCD6IP	10015

**AGCAGATTCGGCGGATCATG**	PPP1CA	5499
**AGCAGCAGTAGAGTTGGGTA**	PRKCE	5581
**AGCAGCGCGTTCCAAGGCAC**	DUSP2	1844
**AGCAGCGGAGAAGTTTCGAA**	GID8	54994
**AGCAGCTATCGGACAACCAG**	PLCE1	51196
**AGCAGCTCAGAGACCACGTG**	EXOC3	11336
**AGCAGCTTCCTACTAGTGGA**	SOS1	6654
**AGCAGGCCCAAAACCCAAAA**	POLR3G	10622
**AGCAGGCCCAAAACCCAAAA**	POLR3GP1	100422395
**AGCAGTACCGCATGGACAGT**	EXOC3	11336
**AGCAGTACTGCTTCGTGCTA**	C12orf49	79794
**AGCAGTGCATTAAACGAGAG**	ONE_NON-GENE_SITE_64	ONE_NON-GENE_SITE_64
**AGCAGTGCCTCTTCTGAACA**	SPRY1	10252
**AGCAGTGCTGGGTCAAGGCC**	UBE2Q2L	100505679
**AGCATGAGCATATCGAGCGT**	ARHGAP35	2909
**AGCATGTAGACCAGGACCTA**	CDK4	1019
**AGCATTCGCCTACTCCGTGA**	IFITM1	8519
**AGCATTCGCCTACTCCGTGA**	IFITM3	10410
**AGCATTCGTAGCCTGAAGGT**	MCM5	4174
**AGCATTGTAGAATGATACGT**	APAF1	317
**AGCCAGGGAGCCATTCACAA**	MAGEA12	4111
**AGCCAGTTGGTAAGCCAGGT**	DDIT4	54541
**AGCCATCAACCGGCTCCATG**	SHC2	25759
**AGCCATCAGTGCATTCGGCG**	ONE_NON-GENE_SITE_65	ONE_NON-GENE_SITE_65
**AGCCCAGACCGAATTACACC**	ONE_NON-GENE_SITE_66	ONE_NON-GENE_SITE_66
**AGCCCCACCTGAGATGTACG**	ARHGEF2	9181
**AGCCCGGACCCGGCTCAACG**	RASGRP2	10235
**AGCCCGGTTAAGGAAAACCC**	ROCK2	9475
**AGCCGACCATGTATCCTGAG**	PRKCD	5580
**AGCCGCACACGGATTCGGTA**	RPL31	6160
**AGCCGCACACGGATTCGGTA**	RPL31P11	641311
**AGCCGGGAAGTACTTCCTCT**	GRB2	2885
**AGCCGGTGATGGTCCCACGC**	BIRC7	79444
**AGCCTTTGCCAGTAAGAGAG**	E2F7	144455
**AGCGAGCAGGAGGACGAGGT**	NUDT4	11163
**AGCGCCACCACCGAAGACTT**	ONE_NON-GENE_SITE_67	ONE_NON-GENE_SITE_67
**AGCGCCATCTGGTATTCGGT**	FAM71E2	284418
**AGCGGATCACGTTCCAGGAG**	SREBF2	6721
**AGCGGCTGAAATAGGGTTGT**	CRYGB	1419
**AGCGGTACAGGTACTCACGA**	ARSI	340075
**AGCGTAAGCCAATACTGATG**	PIK3R1	5295
**AGCGTGCGTTCCGCCTCCCG**	DUSP2	1844
**AGCTAATCTTGGGACATCAG**	MET	4233
**AGCTATCTAGAACAACTCAA**	VPS45	11311
**AGCTCACATCTCGAACCATG**	YPEL5	51646
**AGCTCATTGAGCTACAGGCT**	PRKCSH	5589
**AGCTCCTAGAGACTACAGAC**	KPNB1	3837

**AGCTCCTTGTAGCGCTCGGA**	RASAL3	64926
**AGCTCGGATTCCATGAACCT**	CTNNBL1	56259
**AGCTCTCCAGGCTCGTGGAG**	DDIT4	54541
**AGCTGAACAACCTCTACCTG**	RASGRP4	115727
**AGCTGAACATAAGGCCACAA**	ROCK2	9475
**AGCTGAGCCAACGTGCTCTG**	HDAC9	9734
**AGCTGATGAACAGGAAGTTG**	VPS45	11311
**AGCTGATGTGCATTAGCACT**	RPS6KA3	6197
**AGCTGCCCTCAGCTGCACTG**	PLAGL2	5326
**AGCTGGAGCTGGGCAACGAG**	DUSP6	1848
**AGCTGGATGATGACATGGAC**	PRKCSH	5589
**AGCTGGCCAGCAATCCACGA**	ONE_NON-GENE_SITE_68	ONE_NON-GENE_SITE_68
**AGCTGGCGTCGGGATCGATG**	MLST8	64223
**AGCTGGTAGAGTGCGGTCGT**	ONE_NON-GENE_SITE_69	ONE_NON-GENE_SITE_69
**AGCTGTCAATGAGATCGTCC**	RASGRP1	10125
**AGCTGTCGGCTTCAAACAGA**	PSMD3	5709
**AGCTGTGGCAGCGTCAACAG**	MET	4233
**AGCTTGAATACTACCTAACC**	ONE_NON-GENE_SITE_70	ONE_NON-GENE_SITE_70
**AGCTTGGATTGAGTCAACAG**	FGR	2268
**AGCTTTATGAAGAGTACACA**	PIK3R2	5296
**AGCTTTGAGACTACCTGTCC**	CNKSR1	10256
**AGCTTTGATCCAATGATGTG**	HDAC9	9734
**AGCTTTGTATACGCTGCCAT**	STK4	6789
**AGGAAACATGTAATGATAGG**	BRCA1	672
**AGGAAAGGTTTGGGATTGAG**	LOC107984720	107984720
**AGGAAAGGTTTGGGATTGAG**	SNRPF	6636
**AGGAAATGAGAGTTGTTGAC**	CHMP3	51652
**AGGAAATGAGAGTTGTTGAC**	RNF103-CHMP3	100526767
**AGGAACACCAGGACATCCGT**	RGL2	5863
**AGGAAGACTATGATCGACTG**	RHOC	389
**AGGAAGAGTTGGAAGTTGCG**	ELK1	2002
**AGGAAGATTATGATCGCCTG**	RHOA	387
**AGGAAGCAGAGGAATTGGCC**	STAMBP	10617
**AGGAAGGAAACATGGAACTC**	PRKCA	5578
**AGGAAGTGGAGGTTCAAGCA**	TTK	7272
**AGGAATATCGAAAGGCCGTA**	ONE_NON-GENE_SITE_71	ONE_NON-GENE_SITE_71
**AGGACAGGCAGATACCAGAG**	OSGIN1	29948
**AGGACGCCTAGACATAGTTA**	ONE_NON-GENE_SITE_72	ONE_NON-GENE_SITE_72
**AGGACGGCGGCATGTCAAAG**	ONE_NON-GENE_SITE_73	ONE_NON-GENE_SITE_73
**AGGAGAAGATGCCCGGTGCG**	BCL2	596
**AGGAGAAGGGGGCTAACACG**	ONE_NON-GENE_SITE_74	ONE_NON-GENE_SITE_74
**AGGAGACCGAGACAACAAGA**	VAV1	7409
**AGGAGACCTCTATGCTGTGA**	PRKCH	5583
**AGGAGAGCCTGGCCCAGGCG**	DDIT4	54541
**AGGAGAGGGCAGTGAGAGAT**	C12orf49	79794
**AGGAGAGGGCAGTGAGAGAT**	LOC100132615	100132615
**AGGAGCAGGGGCCTCGACAA**	PREB	10113

**AGGAGCCTACGTGGCAATCG**	PIK3CD	5293
**AGGAGCTAAATGCCTCAGGC**	NPRL2	10641
**AGGAGGACTACGACCGCCTG**	RHOB	388
**AGGAGGAGGAATGGAACCCG**	NUDT4	11163
**AGGAGGAGGAATGGAACCCG**	NUDT4B	440672
**AGGAGGAGGAATGGAACCCG**	NUDT4P2	170688
**AGGAGGAGTACAGCGCCATG**	HRAS	3265
**AGGAGGTCTTACCTTTCGAG**	PRKAG2	51422
**AGGAGTACGCTCTCTTGTCG**	RPS6KA2	6196
**AGGAGTATCCACATCCTCAG**	EZH2	2146
**AGGAGTTAAGACAGCGCTTG**	XBP1	7494
**AGGAGTTGCAGATGGGACTT**	CCND2	894
**AGGAGTTGCAGATGGGACTT**	CCND2-AS1	103752584
**AGGATGAATAAATCGCCCGT**	ONE_NON-GENE_SITE_75	ONE_NON-GENE_SITE_75
**AGGATGTAATCAGACGACAC**	APC	324
**AGGATTAACTAGAACACTAG**	BIRC3	330
**AGGATTTGACTCAGCCATCG**	HDAC10	83933
**AGGCAGATCAGGCACACGAA**	ZFPM1	161882
**AGGCAGCACTTACATCGAAG**	POLR1A	25885
**AGGCAGTCAACCCTAAACAG**	ARID5B	84159
**AGGCATGCGCACGATTCTGG**	CCNA1	8900
**AGGCCAAGCTGACGCACCCG**	SLC25A1	6576
**AGGCCAGGGCCCGTACCTTG**	DCAF12L2	340578
**AGGCCATATTCTGTCTCAAG**	MCM3	4172
**AGGCCGGCGCGCGTGAGCAG**	ICMT	23463
**AGGCGAGTCCACCAGATCAA**	PRKCH	5583
**AGGCGATAGTGGGTAATACG**	TCF7L2	6934
**AGGCGGGATCAGTCGCGTCG**	SPRED2	200734
**AGGCGTGGCAGTCCACGCAT**	C4orf48	401115
**AGGCGTTATGAAGAGCACCT**	RALGAPA1	253959
**AGGCTATGCAGCTTGCAAAG**	NFKB1	4790
**AGGCTCAGGATATTAGGGGC**	RGPD8	727851
**AGGCTCTGTAGATAGCGCGG**	RASSF5	83593
**AGGCTGCGTAGTTGAGCCCT**	QPRT	23475
**AGGCTGGTGCACTATCTGAA**	DCAF12L2	340578
**AGGCTTCCTGATAATCATCC**	MAGEA6	4105
**AGGGACTCAAACTCAGCCCA**	CBLC	23624
**AGGGAGACCTTACGTTAAGC**	ONE_NON-GENE_SITE_76	ONE_NON-GENE_SITE_76
**AGGGATCAGAGAGACCATTG**	PRKCG	5582
**AGGGCACACTAAGCAAAGCA**	DHX8	1659
**AGGGCATTGGAATTCCTAGC**	CNOT2	4848
**AGGGCGGTGGCATGGCGATG**	ARAF	369
**AGGGCTATGGTAGACGCTGG**	SREBF1	6720
**AGGGGACTTCAGAAGCTACG**	FGR	2268
**AGGGGGAGCCACATCAGGTG**	PIK3R2	5296
**AGGGGTGGGTCCCCTCGAGT**	ONE_NON-GENE_SITE_77	ONE_NON-GENE_SITE_77
**AGGGTAACAGCCTTTCTCAA**	IFIT5	24138

**AGGGTACAAACATTGCTGCT**	ATP2A2	488
**AGGGTACAGGCCCATCACAT**	VPS39	23339
**AGGGTCAGGTGGACCACAGG**	BCL2	596
**AGGGTCTTACCTCGGCATGA**	HDAC9	9734
**AGGGTGGCTTGATCTCACGA**	RPS6KA1	6195
**AGGGTTAAGTCCAACAAGCA**	TMEM220	388335
**AGGTAACGAATTGAACGTAC**	ONE_NON-GENE_SITE_78	ONE_NON-GENE_SITE_78
**AGGTAAGTCAGACATCGGTG**	ASXL3	80816
**AGGTACTGCAGCTCCCCGTG**	TYMS	7298
**AGGTATCCGAAGAGCTATAG**	SPRED1	161742
**AGGTCAAAAAGTCGGTTCAC**	EXOC6	54536
**AGGTCCATGGAATTGTACGG**	GMDS	2762
**AGGTCCCACGAGTCATCCTG**	SLC11A2	4891
**AGGTCGATACGAGATCACAG**	PHAX	51808
**AGGTCGTATGGGTTTCTCGG**	GAREM1	64762
**AGGTGGCAAAGATGTACACG**	MAGEA6	4105
**AGGTGGTCAGACATCCACCT**	CECR2	27443
**AGGTTAATCCAGCACGTATG**	BUB1	699
**AGGTTAATCGCCAGCCACAG**	GINS2	51659
**AGGTTCCCACCACCTGAACC**	MCM3	4172
**AGGTTCCCGAACAATAGGAT**	ONE_NON-GENE_SITE_79	ONE_NON-GENE_SITE_79
**AGGTTCTTGTGAGATCAAGT**	HDAC6	10013
**AGGTTTATACTGTACCAAGG**	CECR2	27443
**AGGTTTCACAAGAAGTACTG**	PEX3	8504
**AGTAACACATAGCGTCAAAG**	HACD2	201562
**AGTACAGAGATGGCTACCCT**	RGL2	5863
**AGTACAGTGAAGTTCAAGAT**	YPEL5	51646
**AGTACATGGTAATGAACTCC**	LONRF1	91694
**AGTACCGGAGTACACTGGAA**	SOS1	6654
**AGTACCTACCTAATAAATTC**	DPM1	8813
**AGTACGAAGCCTGGTCACTT**	IFIT5	24138
**AGTACTATAATGACTTCCCG**	SHC1	6464
**AGTACTGACTGCAAATATCC**	SPC25	57405
**AGTAGAAGGGCTTCCCGGCA**	ACVR1B	91
**AGTAGAAGTCATCATCGGGA**	PRPF38B	55119
**AGTAGGTCTGATGTTCGAAG**	MAPK3	5595
**AGTAGTAACATACCTATAGA**	UBE2H	7328
**AGTATGAACATCCAAATCCC**	FBXO11	80204
**AGTATTAGGTCGGAAAGACA**	EXOC2	55770
**AGTATTCAAAAGTACCACAA**	SNX14	57231
**AGTATTGGACATATAACCCT**	SLC22A25	387601
**AGTATTTGATGCTTTAATGG**	PRKAB1	5564
**AGTCAAAGTTGGAACTCTCA**	HDAC6	10013
**AGTCAACCACCATAGTGAGT**	PEA15	8682
**AGTCAGATGCAGACGCAGTG**	HDAC10	83933
**AGTCATACCTGAACAACCCA**	ROCK1	6093
**AGTCATCAATTGTCCCACTA**	CCT4	10575

**AGTCCAAGGAACTTCGCACT**	ONE_NON-GENE_SITE_80	ONE_NON-GENE_SITE_80
**AGTCCACCAGGTCTCCGTAG**	PDGFRB	5159
**AGTCCAGACACTATCAGCAG**	PIK3CG	5294
**AGTCCCATGAGTCTCAGCTG**	KSR1	8844
**AGTCCCCTCTACAATACACC**	ONE_NON-GENE_SITE_81	ONE_NON-GENE_SITE_81
**AGTCCTTAATGACCACCGAG**	HDAC7	51564
**AGTCGAAGTTCCATCGCTCA**	CDKN1A	1026
**AGTCTAAAAACAACGTCCAA**	E2F3	1871
**AGTCTCGACATCCGCCATTG**	ONE_NON-GENE_SITE_82	ONE_NON-GENE_SITE_82
**AGTCTCTGATCCCAACGAAA**	INTS6	26512
**AGTCTGAGGAGTCCACGTAG**	SPRED2	200734
**AGTCTGCATACCTGTCCCGC**	MED12	9968
**AGTCTGGAAAGCGTCACTTG**	MYB	4602
**AGTCTGTCAGTCGTTCCAAG**	RASGRF2	5924
**AGTCTTAATGCCTTCAACGT**	HDAC3	8841
**AGTGAAGTCAGCCGCCTGTC**	QPRT	23475
**AGTGACACCGTAGCTGTGAG**	DCAF12L2	340578
**AGTGACCTCGTCATAAGCGG**	ONE_NON-GENE_SITE_83	ONE_NON-GENE_SITE_83
**AGTGAGGTCTCTTCCAAAAA**	UBTFL1	642623
**AGTGAGGTCTCTTCCAAAAA**	UBTFL6	643615
**AGTGCAGTACCCGTAGAAGG**	PTK2	5747
**AGTGCATTTATACTATCCGA**	ONE_NON-GENE_SITE_84	ONE_NON-GENE_SITE_84
**AGTGCGGTGGTCTTACAGTG**	HDAC1	3065
**AGTGCTGGCCATATTGCTCC**	CTNNBL1	56259
**AGTGCTGGGTCAAGGCCAGG**	UBE2Q2L	100505679
**AGTGCTTGTCCAGCTCGGGT**	SPRY4	81848
**AGTGCTTTAGTGCTACACGT**	ONE_NON-GENE_SITE_85	ONE_NON-GENE_SITE_85
**AGTGGATAGTGCTCTGAGCA**	TRIM64	120146
**AGTGGATAGTGCTCTGAGCA**	TRIM64B	642446
**AGTGGCAATGTGTGCTGACA**	SOS1	6654
**AGTGGCAGACGAGCTGGTTG**	TFDP1	7027
**AGTGGCCAAATCACGCACTT**	ONE_NON-GENE_SITE_86	ONE_NON-GENE_SITE_86
**AGTGGCCTGGAGACAGGATG**	AURKA	6790
**AGTGGCGCCTGTGAAACGAG**	YPEL5	51646
**AGTGGTACAGACCAAGTATG**	PFDN5	5204
**AGTGGTAGATTTAGAAGAAG**	XBP1	7494
**AGTGTACTCAGCGAGGTCAA**	GMDS	2762
**AGTGTCATAAAACGCATGGT**	ONE_NON-GENE_SITE_87	ONE_NON-GENE_SITE_87
**AGTGTCCAGGATTTGTCCGG**	ARAF	369
**AGTGTGAGAGTCCCCAATGG**	CDK4	1019
**AGTGTGCCTCATGTTAATCG**	ONE_NON-GENE_SITE_88	ONE_NON-GENE_SITE_88
**AGTGTGTGCCCACACGACCC**	MCM4	4173
**AGTGTGTGGGTTCATTCACC**	EBP	10682
**AGTTAAAATAAGCAATTGAG**	LPCAT3	10162
**AGTTAATGGGTGCGTTCACC**	MLST8	64223
**AGTTACCGACTACCTCAGGA**	BRIP1	83990
**AGTTATTGACACTCAACAGA**	PFDN1	5201

**AGTTCTGGACACTTTGCATG**	FRK	2444
**AGTTCTTGAATGTAGAGATG**	BIRC5	332
**AGTTGACAGCCTGAACCGGT**	ONE_NON-GENE_SITE_89	ONE_NON-GENE_SITE_89
**AGTTGATCTTTGGAGCATTG**	TBK1	29110
**AGTTGCTAAACAGTTGGCAT**	JAK2	3717
**AGTTGGACAGCTACGCGCCA**	TRAPPC4	51399
**AGTTGGATGATATGACACTG**	SIRT1	23411
**AGTTTACCTGATAGTCCACG**	CSNK2A2	1459
**AGTTTCAGAGGATCCGATGA**	SNX13	23161
**AGTTTCCTGAAAGTAGATAG**	RGL3	57139
**ATAAACCTGATCCCAAAACA**	IRF2	3660
**ATAAATGCACAACCAACGGG**	ONE_NON-GENE_SITE_90	ONE_NON-GENE_SITE_90
**ATAAATTGAAATAAAAATCC**	MSMO1	6307
**ATAACGAAGGACGTAAGGCT**	IFIT5	24138
**ATAAGAGACTGGACGCTGTG**	MAEA	10296
**ATAATAAGGCCATGACTACG**	PSMB1	5689
**ATAATGGCATAATAAAGGTT**	PEX3	8504
**ATACAAAGATACTCTCCCCA**	PDCD6IP	10015
**ATACACAGAGATATAAAAGC**	STK3	6788
**ATACACCGAGATATCAAGGC**	STK4	6789
**ATACACCGTGCCGAACGCAC**	EGFR	1956
**ATACAGCTCCAGTTCCATGC**	TBCB	1155
**ATACATGAACTGAAGCGTCA**	RALB	5899
**ATACATTCACGGAAAACGGC**	RALBP1	10928
**ATACCCAATAGAGTCCGAGG**	BRAF	673
**ATACCGTTTCATTGAGAGGG**	UBAP2L	9898
**ATACGACGCATAGTCAAAGA**	RAF1	5894
**ATACGACTCTCATGGTTCAG**	PLCE1	51196
**ATACTAGACTCACCTGAAGC**	C12orf77	196415
**ATACTCGTAAGGATGTACGG**	PLAGL2	5326
**ATACTGGACATAATCCCTCA**	SNX13	23161
**ATACTTACAGGAAATAGTGG**	POLR3G	10622
**ATACTTACCATATTCGACTG**	EXOC6	54536
**ATACTTTAGTAATTCAAGGA**	HDAC2	3066
**ATAGAATCCCTTCGTAACAG**	ONE_NON-GENE_SITE_91	ONE_NON-GENE_SITE_91
**ATAGACAACTATGGGCAGGT**	INTS6	26512
**ATAGACTTATTCAGCTTGAG**	FAM217B	63939
**ATAGAGGTGAGGTTTCCGCA**	TTC7A	57217
**ATAGATGATTTACCAATATG**	TM9SF3	56889
**ATAGATGTCATAACAGTATT**	VTA1	51534
**ATAGCCTTGTCAGATAAGGA**	SIRT1	23411
**ATAGCGTGTACAATACATCC**	CDC6	990
**ATAGGCGAGTATTAGTGGAA**	FNTA	2339
**ATAGTATGAGACTAACCCCG**	ONE_NON-GENE_SITE_92	ONE_NON-GENE_SITE_92
**ATAGTCAAATATCCCTTCAG**	HDAC8	55869
**ATAGTCATAGAGGGCCACAA**	SRC	6714
**ATAGTCATGAGGTAGCTGCG**	EIF4EBP2	1979

**ATAGTTAGATAAGACTGCTA**	EGFR	1956
**ATAGTTTCCCAACAAAATCG**	RALGAPA2	57186
**ATATAATTCATATACGGTAC**	ONE_NON-GENE_SITE_93	ONE_NON-GENE_SITE_93
**ATATAGGGCTTATAAACCCG**	ONE_NON-GENE_SITE_94	ONE_NON-GENE_SITE_94
**ATATATAACAGATCATGCAC**	FBXO11	80204
**ATATATTCAGGATTTCCGGA**	UBTFL1	642623
**ATATATTTCTAACTCGGAGG**	ONE_NON-GENE_SITE_95	ONE_NON-GENE_SITE_95
**ATATCATGCCAAGAACGAAT**	SEC23B	10483
**ATATCCGTAACACCGCCAGT**	NEDD9	4739
**ATATTATTTCAACAACCCAG**	EMC3	55831
**ATATTGTAGTAACCATGAGA**	PREX2	80243
**ATATTTCTGGTGGCATTCGG**	C12orf77	196415
**ATCACACATCATGTAAAGGA**	RPS6KA3	6197
**ATCACCATAAGATCATTGGT**	RAB3GAP2	25782
**ATCACCATGAAAATATCAGA**	PRKAA1	5562
**ATCAGATCCTCACCAAGACA**	HDAC5	10014
**ATCAGCTAGATGAAGAAGAG**	ATXN3L	92552
**ATCAGCTAGATGAAGAAGAG**	GS1-600G8.3	100093698
**ATCATCACACAGTGGTGTGA**	ARAF	369
**ATCATTAGCAACGTCTTCGA**	FLVCR1	28982
**ATCCAAACGTTTCACACCCC**	ONE_NON-GENE_SITE_96	ONE_NON-GENE_SITE_96
**ATCCAGACCATGATCACACA**	MAPK1	5594
**ATCCAGAGGGCACCTTACCG**	ONE_NON-GENE_SITE_97	ONE_NON-GENE_SITE_97
**ATCCCCAAGACATGCTCGGT**	ONE_NON-GENE_SITE_98	ONE_NON-GENE_SITE_98
**ATCCCCAGGAACTCACACGG**	RAC2	5880
**ATCCCGAACCTGCATTCTGA**	MED11	400569
**ATCCCGCGTCAGCACGTACA**	VPS11	55823
**ATCCGGGAGATGATACTGAA**	GID8	54994
**ATCCGGGCGTCCAGGGTTCG**	NRTN	4902
**ATCCGGGTCTGTCAGGACCA**	PEBP1	5037
**ATCCTAAGAAGAAATATACA**	PAK1	5058
**ATCCTAATCGAGATCCCTAG**	ONE_NON-GENE_SITE_99	ONE_NON-GENE_SITE_99
**ATCCTTAGAAAAGCTTAGAC**	SMU1	55234
**ATCGAATGATATCTTCTGTG**	EXOC6	54536
**ATCGAGCCAAGAAATTTGCA**	ATXN3L	92552
**ATCGAGCCAAGAAATTTGCA**	GS1-600G8.3	100093698
**ATCGCACTAGCCCAAGCAAT**	RASSF7	8045
**ATCGCCAGGAGTATTATCTG**	RASA1	5921
**ATCGCTCCGCTAGGTGTCAG**	ERBB2	2064
**ATCGGGGACCGGCACGCGGG**	C4orf48	401115
**ATCTACATATTCCACAGCCA**	MSMO1	6307
**ATCTCCGAGCACAAGTACCC**	PIK3R6	146850
**ATCTCTATGATCTGTATACA**	EXOC5	10640
**ATCTCTCGGGCAGACAACAG**	PRKCD	5580
**ATCTGCACAGACCGAATATG**	PRKCI	5584
**ATCTGCAGGATGGCATTTAA**	ANAPC11	51529
**ATCTGCGCATGAGTTCAGCA**	MCM7	4176

**ATCTGTCCGCAGAGGCCGCG**	PIK3R2	5296
**ATCTTGGCAAATATAACCGA**	KPNB1	3837
**ATCTTTCTTTCAACGTACAG**	RGL1	23179
**ATGAAAGAGTGCGTACACAC**	TSC1	7248
**ATGAACCGGGACGAGAATGG**	DUSP4	1846
**ATGAACCGGGGCATCAACGA**	CYTH2	9266
**ATGAATTTCAGGTGGAGGCG**	BPIFB2	80341
**ATGACAACTAAAGCACCGCA**	XIAP	331
**ATGACAGTACAGACGCTGCT**	HDAC10	83933
**ATGACCCAGATGCAGAGGTG**	TMEM220	388335
**ATGACGTTAGCATCATAGGA**	PRKAA2	5563
**ATGACTACCGGTCATCACAG**	ONE_NON-GENE_SITE_100	ONE_NON-GENE_SITE_100
**ATGACTAGGCCAGTCATTCG**	ONE_NON-GENE_SITE_101	ONE_NON-GENE_SITE_101
**ATGAGAACGCCACCCCACAG**	SLC11A2	4891
**ATGAGAAGACATTCATAACC**	RASSF6	166824
**ATGAGTATATCAAATTTCTA**	HDAC2	3066
**ATGATACCCATGAACGACTC**	ONE_NON-GENE_SITE_102	ONE_NON-GENE_SITE_102
**ATGATATTGTCGTCGTGCTT**	PEX3	8504
**ATGATCAGAAAGAGCCGTAG**	SCD	6319
**ATGATCAGACAGTATCCCCG**	CASP8	841
**ATGATGGAGGAGGATTTGCA**	CREBBP	1387
**ATGATGGTCTGCCAAGTGGG**	CTNNB1	1499
**ATGCACTGCATAAGCAGATG**	HDAC8	55869
**ATGCAGCCAATTATACAGTG**	FNTA	2339
**ATGCAGGCATCGTATCACTC**	ONE_NON-GENE_SITE_103	ONE_NON-GENE_SITE_103
**ATGCCTCCAGAAGTACACGG**	SREBF1	6720
**ATGCCTGGCAGCATCGACAA**	FNTA	2339
**ATGCCTGGCAGCATCGACAA**	FNTAP1	283226
**ATGCGCTTCTCCCAGCCGGG**	PIN1	5300
**ATGCGGGTATTGAGTCCCTA**	ONE_NON-GENE_SITE_104	ONE_NON-GENE_SITE_104
**ATGCGTATCTGTCCACGACG**	DTYMK	1841
**ATGCTAAAATCAAACGAGAG**	EXOC8	149371
**ATGCTAACGGGCCCATACGA**	ONE_NON-GENE_SITE_105	ONE_NON-GENE_SITE_105
**ATGCTAGTTCATTACGATGC**	ONE_NON-GENE_SITE_106	ONE_NON-GENE_SITE_106
**ATGCTATGTCAGAACACCGG**	BIRC2	329
**ATGCTGATATCCATCCAATG**	STK4	6789
**ATGCTTTGGCGGGTATGCCG**	ATP6V0A1	535
**ATGGACACTTTAAAAAGTCA**	RAB3GAP2	25782
**ATGGACCTAATCAAGCTCAC**	UNG	7374
**ATGGACTTGGTATAGCCCTG**	MAPK3	5595
**ATGGAGAAGAGGGTCATCCT**	CASP8	841
**ATGGAGACAGTGGTAAACAG**	SOS1	6654
**ATGGATATACTCAGTTAACA**	XIAP	331
**ATGGATTGTGCTCCCCGCGT**	ONE_NON-GENE_SITE_107	ONE_NON-GENE_SITE_107
**ATGGCACATGTATTTACGAC**	ONE_NON-GENE_SITE_108	ONE_NON-GENE_SITE_108
**ATGGCATCATAGATGGACTG**	RASSF5	83593
**ATGGCCTCACACATGTACTG**	TTC7A	57217

**ATGGCGACAGCATAGGCTCG**	DPM2	8818
**ATGGCTCGTATTGCTATCAT**	EED	8726
**ATGGGATTATCGCACCCCCT**	ONE_NON-GENE_SITE_109	ONE_NON-GENE_SITE_109
**ATGGGCATCAAGATCAATGA**	HDAC11	79885
**ATGGGGATGAAGAACCAGCT**	CHMP3	51652
**ATGGGGATGAAGAACCAGCT**	RNF103-CHMP3	100526767
**ATGGGTCACCATACATGGAA**	APAF1	317
**ATGGTTCCTGAGGTGGAGGC**	SPRR1B	6699
**ATGGTTCTGGATCAGCTGGA**	HRAS	3265
**ATGTACTGAACGTAGCAGTG**	EXOC3	11336
**ATGTAGATGGCGCCCCCTTG**	ONE_NON-GENE_SITE_110	ONE_NON-GENE_SITE_110
**ATGTATGGTTATGACAAGAA**	LIPK	643414
**ATGTATTGCCTCCGACCTGG**	HCK	3055
**ATGTCATCCAATTCGTGTGC**	ONE_NON-GENE_SITE_111	ONE_NON-GENE_SITE_111
**ATGTCCAGTGTGTTACACCC**	BARD1	580
**ATGTCCCAAATTGCTTCCAA**	ZFP90	146198
**ATGTCGGAGTAAGAGACACC**	VPS16	64601
**ATGTCGTCTTCCAAGTAGAG**	SCD	6319
**ATGTCTGAGGTGGATCGTTG**	RASAL2	9462
**ATGTGAGAAATGTATCAGCA**	TRIM64	120146
**ATGTGAGAAATGTATCAGCA**	TRIM64B	642446
**ATGTGATCAGAACTGGTACA**	GRB2	2885
**ATGTGCAGGTCTGAATAGCG**	KSR2	283455
**ATGTGCGCTTGGAATCCAAG**	TYMS	7298
**ATGTGCTCAATGAAGTCATG**	CCND2	894
**ATGTGGGAGATACTGATGCA**	PTK2	5747
**ATGTGTAACAGTTCCTGCAT**	TP53	7157
**ATGTGTCTGAAGGAATTGTG**	RABGAP1	23637
**ATGTGTGGAGATTACTACAT**	RASA1	5921
**ATGTTAATGGGCCCAAGTAT**	ONE_NON-GENE_SITE_112	ONE_NON-GENE_SITE_112
**ATGTTATAAACGGACGGGTT**	ONE_NON-GENE_SITE_113	ONE_NON-GENE_SITE_113
**ATGTTGACTGTTACGCACAG**	RAPGEF1	2889
**ATGTTGATCACGGTGGACCT**	IFITM1	8519
**ATGTTGGGGGTACATTCAGG**	EZH2	2146
**ATGTTGTTTCTGTGGAAAAG**	MYC	4609
**ATTAAATCCTGGGTCCAGTG**	DDX46	9879
**ATTAAATTGAGTGACAATAT**	SNX13	23161
**ATTAACGTACCTATTCAACT**	ONE_NON-GENE_SITE_114	ONE_NON-GENE_SITE_114
**ATTACAGAGTTGATGAGACA**	FRK	2444
**ATTACTACAACAGCATCCCG**	SHC2	25759
**ATTAGCAAATAAAGTAGACA**	DHFR	1719
**ATTAGCAAATAAAGTAGACA**	DHFRP1	573971
**ATTAGTACACCGAACGACAA**	ONE_NON-GENE_SITE_115	ONE_NON-GENE_SITE_115
**ATTATACCCACGACGAGCTG**	ONE_NON-GENE_SITE_116	ONE_NON-GENE_SITE_116
**ATTATGCTGAAGTAGAAACA**	EXOC2	55770
**ATTATGTAACCTCATTACGC**	SHOC2	8036
**ATTCATAGTCGGGATCATAG**	RAPGEF1	2889

**ATTCCTGCTGCGTTCCCGCG**	SPNS1	83985
**ATTCTATGGCTTGTATCGCA**	ONE_NON-GENE_SITE_117	ONE_NON-GENE_SITE_117
**ATTCTGCCGACTCACTACTG**	ONE_NON-GENE_SITE_118	ONE_NON-GENE_SITE_118
**ATTCTGGAATATGCACCACT**	AURKA	6790
**ATTCTGTTACATCAATCAAA**	RHEB	6009
**ATTGACATGTCCATAGTATG**	CCNA2	890
**ATTGAGCAATTGGGAACCGA**	BIRC3	330
**ATTGATGAAGCCTCTCCAGT**	PLCXD3	345557
**ATTGCATCATCAGCAGATGG**	ATP6V0A1	535
**ATTGCCACAAGCATACTGCA**	SMC1A	8243
**ATTGCCCCCCTCCAAACACG**	CREBBP	1387
**ATTGGAACTAAGCATTAGAC**	CTNNBL1	56259
**ATTGGTACTAGAAACCGATT**	RNF103	7844
**ATTGGTACTAGAAACCGATT**	RNF103-CHMP3	100526767
**ATTGGTGCTCTCCCTCAGGG**	PRKAB2	5565
**ATTGGTGTTGACGTGCATGA**	DUSP3	1845
**ATTGGTTTATTATAGCCGGT**	ONE_NON-GENE_SITE_119	ONE_NON-GENE_SITE_119
**ATTGTAGTCTCCAATATGAA**	ITFG1	81533
**ATTGTCCAAAATGTTCTCAA**	CDC25A	993
**ATTGTGCATCATTGGCACCG**	CHURC1-FNTB	100529261
**ATTGTGCATCATTGGCACCG**	FNTB	2342
**ATTGTGGAATTGATGCGTGA**	CASP3	836
**ATTGTTATGTGTCTGATGTG**	ARHGAP35	2909
**ATTGTTGGGGCTACTGGACA**	RAF1	5894
**ATTTAACTTACTGCTATATG**	RB1	5925
**ATTTAAGATACTTACACAGT**	RAC1	5879
**ATTTACAAGCACCCATCATG**	RIC1	57589
**ATTTACGGAAAAGTAGTCCA**	RASA1	5921
**ATTTAGCATGTTGTTCCCAA**	XIAP	331
**ATTTAGCATGTTGTTCCCAA**	XIAPP1	100996851
**ATTTAGGGAACGGACCATCC**	ONE_NON-GENE_SITE_120	ONE_NON-GENE_SITE_120
**ATTTATAGCAACCTGCTCAG**	APAF1	317
**ATTTATAGTCACAATCGAAT**	ONE_NON-GENE_SITE_121	ONE_NON-GENE_SITE_121
**ATTTATGCTCCAGAAAAGTG**	RASGRP3	25780
**ATTTCAATATTCCTTATGAT**	MSMO1	6307
**ATTTCACCAGGCGACCTGGG**	KIR2DL1	3802
**ATTTCAGCACCATATGAAGG**	UBE2H	7328
**ATTTCATCATTTGATAATTC**	SNX14	57231
**ATTTCATGTAGATACTCCAG**	AKT3	10000
**ATTTCCTGAAGACAGATGAA**	BLK	640
**ATTTCGCAGATCATCGACAT**	ONE_NON-GENE_SITE_122	ONE_NON-GENE_SITE_122
**ATTTCGGTGGACTTCGAAGA**	SET	6418
**ATTTCGGTGGACTTCGAAGA**	SETP14	389168
**ATTTCTACAACCATAAGGTA**	RASSF2	9770
**ATTTGAAAGAAGATAAACCT**	BARD1	580
**ATTTGAGCGTATTCTCTACG**	HDAC8	55869
**ATTTTTTCGTCTCGTGCTAA**	ONE_NON-GENE_SITE_123	ONE_NON-GENE_SITE_123

**ATTTTTTCGTCTCGTGCTAA**	INACTIVE_6T+_2	INACTIVE_6T+_2
**CAAAAATAGAGTTGCAATCT**	INIP	58493
**CAAAAGCACTCGCACAACGT**	RASSF10	644943
**CAAAATAGAGTTCATTGTCG**	PLCXD3	345557
**CAAAATCAGATTTCTTGACA**	PRKAB2	5565
**CAAAATCATGCTCAACACGT**	PSMD11	5717
**CAAAATCCTGCACATCGAAG**	VAV1	7409
**CAAAATGAGCAGCAAAACCA**	TFDP2	7029
**CAAAATGTAGTGACCGTTGG**	IRS1	3667
**CAAACACATCCAGTCGAGTT**	ONE_NON-GENE_SITE_124	ONE_NON-GENE_SITE_124
**CAAACACCTGAACCTGTGGA**	RSPH3	83861
**CAAACAGATAGAGTGTCATG**	RTN4R	65078
**CAAACCCCAGGAAACAAGCT**	ICMT	23463
**CAAACCTAAGTTGTACTGGT**	SOS2	6655
**CAAACGCATCACAGCCTCAG**	CAMK2D	817
**CAAACTGGTACTCGAGAACG**	CTNNBL1	56259
**CAAACTTGGACAGCTCTCTA**	UBE2H	7328
**CAAAGAAGTATCGAACGATG**	CERS2	29956
**CAAAGAGATTATACGCCTCA**	GMDS	2762
**CAAAGAGGGCATCAGTGACG**	AKT2	208
**CAAAGCCAACCGCCCATATG**	ONE_NON-GENE_SITE_125	ONE_NON-GENE_SITE_125
**CAAAGCCTGGTCAATGAAGT**	DUSP3	1845
**CAAAGGCTACGACTGGACGC**	SOX9	6662
**CAAAGTACTCCACCACACCC**	SPTLC2	9517
**CAAAGTAGTTCTAGATGACA**	HSPE1	3336
**CAAAGTAGTTCTAGATGACA**	HSPE1P2	326300
**CAAAGTAGTTCTAGATGACA**	HSPE1P3	100507046
**CAAAGTAGTTCTAGATGACA**	HSPE1P4	100287369
**CAAAGTAGTTCTAGATGACA**	HSPE1P5	100506000
**CAAAGTAGTTCTAGATGACA**	HSPE1P8	100292290
**CAAAGTCCCTGACTCCCGTG**	FANCA	2175
**CAAAGTCGCTCGCAAGTCGC**	RIMS4	140730
**CAAAGTCGGTCAGTTTGATG**	RPS6KB2	6199
**CAAAGTGAGAAAGATCTTTG**	RPS4X	6191
**CAAATAGTGACCCAACAATA**	FBXO11	80204
**CAAATATGTCACCTGTTGGC**	LPCAT3	10162
**CAAATATTATTCCACAGCTG**	CDK2	1017
**CAAATGAGATAGATTGCATG**	SHC1	6464
**CAAATGATGCAGGACTCACA**	RAC1	5879
**CAAATGATGCAGGACTCACA**	RAC1P2	442775
**CAAATGCAAGTGCTCCCGGA**	CXCL14	9547
**CAACAAACCCACGTACAACG**	PRKCH	5583
**CAACAATCTTTACGTTGCCA**	ONE_NON-GENE_SITE_126	ONE_NON-GENE_SITE_126
**CAACACGCCCGAGTCCATCG**	IRS2	8660
**CAACACTCCCATCATCCTAG**	RAC1	5879
**CAACACTCCCATCATCCTAG**	RAC1P2	442775
**CAACATCTGTTGCAATGTGA**	MDM2	4193

**CAACCAGTTAGCGTGAAAGT**	HSPE1	3336
**CAACCAGTTAGCGTGAAAGT**	HSPE1P3	100507046
**CAACCAGTTAGCGTGAAAGT**	HSPE1P4	100287369
**CAACCAGTTAGCGTGAAAGT**	HSPE1P5	100506000
**CAACCATGAGCAGTCCTACG**	PEX16	9409
**CAACCCCCGGCTGGACACGT**	LGALS7	3963
**CAACCCCCGGCTGGACACGT**	LGALS7B	653499
**CAACCCGGAATATGGCAGGG**	FANCC	2176
**CAACCGCCACATCTACCCAG**	HDAC11	79885
**CAACCGTGATACTCACCCCA**	NOX1	27035
**CAACCTCCCCACATACAAGC**	MRAS	22808
**CAACCTCTCGTACATCGGCG**	MAPK1	5594
**CAACGGAAACTCACCGTCCA**	MZT1	440145
**CAACGGACCTGGACGTAGAG**	TBC1D3B	414059
**CAACGTCCAATAAACAGCAA**	LYN	4067
**CAACTCATTGATAAAAGTCG**	ARHGAP35	2909
**CAACTGCAGATGAACAAGGT**	HDAC4	9759
**CAACTGGTAGTCCATAGTGA**	CTNNB1	1499
**CAACTTTCCGCATAAGACGG**	FAM71E2	284418
**CAAGAAACTGAACGCCTCGG**	NEDD9	4739
**CAAGAACGTGACAGATGAGC**	JUN	3725
**CAAGAAGATTGAGAATCTGG**	PPP1R7	5510
**CAAGAAGTGCGAGTACACCA**	CISD2	493856
**CAAGAAGTGCGAGTACACCA**	SLC9B1	150159
**CAAGACAAGAAAAGCATGAA**	SPRY1	10252
**CAAGATGGTCACGTGCATTG**	PANX1	24145
**CAAGATGTGCCAATTCTGCG**	PIK3CD	5293
**CAAGCAAGATTTCTTAAAAG**	RGPD5	84220
**CAAGCAAGATTTCTTAAAAG**	RGPD6	729540
**CAAGCAAGATTTCTTAAAAG**	RGPD8	727851
**CAAGCACCGCGTTAGCATGG**	AGPAT3	56894
**CAAGCAGGGTCAGTACTGCA**	TMEM220	388335
**CAAGCTCGGCCGGTACAACG**	TBCB	1155
**CAAGCTGGTCAAACTTCATG**	HCK	3055
**CAAGCTGGTTGAGACTACCA**	NFE2L2	4780
**CAAGGAATGACATCTCGGTC**	CASP3	836
**CAAGGACACAGCACACACAC**	RPS6KB2	6199
**CAAGGAGAAGCTTATTCGTG**	BUB1	699
**CAAGGAGCCCTGCCACCCCA**	SPRR1A	6698
**CAAGGAGCCCTGCCACCCCA**	SPRR1B	6699
**CAAGGCAAAATACGCTCCAA**	ONE_NON-GENE_SITE_127	ONE_NON-GENE_SITE_127
**CAAGGCCAGCTCACCACAGG**	LOC653406	653406
**CAAGGCCAGCTCACCACAGG**	LOC728519	728519
**CAAGGCCAGCTCACCACAGG**	NAIP	4671
**CAAGGGGAAGTGCATCAGAG**	E2F2	1870
**CAAGGTGAAATATCCCAGAG**	ROS1	6098
**CAAGTACCTTCCGTTGAGAG**	RASGRF2	5924

**CAAGTCTACGCAGCCGTAAG**	ONE_NON-GENE_SITE_128	ONE_NON-GENE_SITE_128
**CAAGTCTAGAGTACGTTACG**	ONE_NON-GENE_SITE_129	ONE_NON-GENE_SITE_129
**CAAGTCTGGCTCGTTCTCAG**	BIRC5	332
**CAAGTGGTATGACATGCCAA**	MED12	9968
**CAAGTTCATTCACGTGGCAC**	RASGRP4	115727
**CAAGTTCTGGAATTGAAGCA**	ASXL3	80816
**CAAGTTTGGGAAAATCCTTG**	PDPK1	5170
**CAATAATAGCAAGTCATTTG**	NRAS	4893
**CAATGACCACATGAAAATAA**	LOC100506133	100506133
**CAATGACCACATGAAAATAA**	UBE2N	7334
**CAATGACCACATGAAAATAA**	UBE2NL	389898
**CAATGACCACTCATTGACCT**	CHMP3	51652
**CAATGACCACTCATTGACCT**	RNF103-CHMP3	100526767
**CAATGACGGGATCCTACGGA**	PEX16	9409
**CAATGTAGGGAGCCTAGCGC**	ONE_NON-GENE_SITE_130	ONE_NON-GENE_SITE_130
**CAATGTAGGTACTACAAACC**	SCD	6319
**CAATGTGCCCATCATCCTGG**	RHOB	388
**CAATTACTGCAATTACCGCA**	RASGRP3	25780
**CAATTAGCTTACTTCTCCCA**	FAM217B	63939
**CAATTCTTTGAATGAGCTGG**	GRB2	2885
**CAATTCTTTGTAAAACTCTG**	DPF1	8193
**CAATTGGGATGACGCACCCC**	ONE_NON-GENE_SITE_131	ONE_NON-GENE_SITE_131
**CACAAGAAGCCTACGACCAG**	TBCB	1155
**CACAAGATAGAAACTTGGAG**	PRKAG1	5571
**CACAATTCCCTTAGCGACGT**	ONE_NON-GENE_SITE_132	ONE_NON-GENE_SITE_132
**CACACAAGTGTTCATCCTCA**	DDIT4	54541
**CACACAGTGTCCAGATACGA**	KPNB1	3837
**CACACATGGTCCACCCACAG**	AGPAT3	56894
**CACACCTTGTTTGCTCTCTG**	PDPK1	5170
**CACACGCCCATCCTCCTGGT**	RAC3	5881
**CACACGTGGTGCGACCTCTG**	RASSF1	11186
**CACACTCCAAGAAGGCCCCG**	RGL2	5863
**CACACTTACTCTCCACGTCG**	JAK1	3716
**CACACTTGTGAAATCTACCC**	PDCD6IP	10015
**CACAGAGATTCCGACCCTTG**	BRIP1	83990
**CACAGGTGTGAAATCCCCGG**	E2F1	1869
**CACAGGTTAGTACGCACCCA**	ONE_NON-GENE_SITE_133	ONE_NON-GENE_SITE_133
**CACAGGTTTCATATTCCAGA**	FUNDC2	65991
**CACAGTAATAAGCCACCACA**	VTA1	51534
**CACAGTCATGACCCGGTCCG**	HDAC1	3065
**CACAGTGATCCACTGCGAAG**	VCP	7415
**CACAGTTGTAACAGCTGTTT**	TMEM263	90488
**CACATACAGAGATGGCCGAG**	SNRPD3	6634
**CACATCACCACAGATGTACA**	INPP4B	8821
**CACATCCAGTCAGAAACCAG**	NFE2L2	4780
**CACATCCCTCACTCAACACG**	ZFP90	146198
**CACATGGACCGGCATCAACG**	PLXNB1	5364

**CACATGGCGTGCATGTACGT**	RPTOR	57521
**CACATTACCTTCTACTGAAG**	DHFR	1719
**CACATTGCGAAACGGACGGT**	ONE_NON-GENE_SITE_134	ONE_NON-GENE_SITE_134
**CACCAAATCATACTGTCCCT**	JAK1	3716
**CACCAATAAGAATCATAGGG**	EMC3	55831
**CACCAATTCAAATAACCATT**	TFDP2	7029
**CACCCCGCACTGGCCCAGAG**	FAU	2197
**CACCGGGCTGGCGCTCTACG**	ICMT	23463
**CACCTACCAACAATCCCGGA**	RASGRP2	10235
**CACCTGCACATGGAGATCGT**	ACVR1B	91
**CACCTGCATCAACCAGACAG**	PIK3CD	5293
**CACGCTCCCGCATACCACCA**	PLXNB1	5364
**CACGGAGCTGCAGAGCACTG**	NRTN	4902
**CACGGCGGCCTGAACAGTAG**	ZFPM1	161882
**CACGTCACAATCCAAGACCA**	TBC1D3B	414059
**CACGTGGACAGTTGTGCCGT**	RPL26L1	51121
**CACTACACCAAGACAAGTGA**	FRK	2444
**CACTACCACGCAGGCCAGTG**	HCFC1	3054
**CACTACTAGCTCAAGGACCG**	ONE_NON-GENE_SITE_135	ONE_NON-GENE_SITE_135
**CACTCACCCAGTCCTCGAAG**	PRKCE	5581
**CACTCCTTTCAACCAATACT**	HSPE1	3336
**CACTCCTTTCAACCAATACT**	HSPE1-MOB4	100529241
**CACTCCTTTCAACCAATACT**	HSPE1P2	326300
**CACTGAGAAGATCATCAGAG**	EXOC7	23265
**CACTGCTACAGCATGCTCTG**	NPRL3	8131
**CACTTACCCTGCCGTAGACA**	MCM3	4172
**CACTTCCAGATCATGCTGCC**	PRKAB1	5564
**CACTTGAAGTAGGAGCACTG**	CCND2	894
**CACTTGGCCGCACGTCCTGA**	SLCO2B1	11309
**CACTTTGATGAAACCCGTGT**	RASSF5	83593
**CAGAAACAAGTTGACGGGAG**	CDK2	1017
**CAGAAACTTGCGGATCACGT**	VPS11	55823
**CAGAAATAATATTCAAATTC**	C12orf77	196415
**CAGAACCTGCAAGTAATCCG**	ERBB2	2064
**CAGAACCTTAGTCCCGGGAA**	TFDP1	7027
**CAGAACGCCACAGAGCACGT**	INSRR	3645
**CAGAAGAAAACGAAATCGTG**	SUZ12	23512
**CAGAAGAACTTCAACCTGCC**	KSR2	283455
**CAGAAGCACCGTCACCCATG**	FAM20B	9917
**CAGAATACTTTGGCCGTTGG**	ONE_NON-GENE_SITE_136	ONE_NON-GENE_SITE_136
**CAGAATGCAAGAAGCCCAGA**	TP53	7157
**CAGACACAGAATTACCCCAA**	ETS2	2114
**CAGACACCCAGAGCAAAAGG**	BSCL2	26580
**CAGACACCCAGAGCAAAAGG**	HNRNPUL2-BSCL2	100534595
**CAGACAGGGCCTTTGCCGGT**	SREBF1	6720
**CAGACAGTTAATATCACTGC**	BRCA1	672
**CAGACATTGACCTCACCAAG**	RPS18	6222

**CAGACATTGACCTCACCAAG**	RPS18P12	388339
**CAGACATTGACCTCACCAAG**	RPS18P13	100271364
**CAGACATTGACCTCACCAAG**	RPS18P6	100271076
**CAGACCAAAAGTTCATCCAG**	RGL1	23179
**CAGACCCCATCGAACACTTG**	ONE_NON-GENE_SITE_137	ONE_NON-GENE_SITE_137
**CAGACTCTCAATGTACCTTG**	CDC6	990
**CAGACTTCGGAACAGCTGCG**	RASAL3	64926
**CAGACTTCTGGGCATATTTG**	NUDT4	11163
**CAGAGATTCAGGCCACCATG**	CHMP3	51652
**CAGAGATTCAGGCCACCATG**	RNF103-CHMP3	100526767
**CAGAGCATCATCAGCACCGT**	SHC2	25759
**CAGAGCCACATAGACGGGGG**	PRKAG1	5571
**CAGAGCCATTCATCGCAGGG**	ONE_NON-GENE_SITE_138	ONE_NON-GENE_SITE_138
**CAGAGCTACATACTTGCTAG**	SMC1A	8243
**CAGAGCTCGATTCAGGACTG**	RGL1	23179
**CAGAGCTCTGGGTACCGCTA**	FYN	2534
**CAGAGGACTTGCAATATGAC**	PEX3	8504
**CAGAGGGCCAAGATCAGTCA**	PRKCB	5579
**CAGAGGTCTTCGCAACAGCC**	OSGIN1	29948
**CAGATCCGGACTATGGAAAG**	RASSF8	11228
**CAGATGAAAGTGTCCTAATG**	SLBP	7884
**CAGATTAAGTTCGGGCTAAG**	ONE_NON-GENE_SITE_139	ONE_NON-GENE_SITE_139
**CAGCAACCAGAAACTCAAGC**	SREBF1	6720
**CAGCAACCGGTTATTAACTC**	ONE_NON-GENE_SITE_140	ONE_NON-GENE_SITE_140
**CAGCAAGAAGGCGCGATCTG**	ONE_NON-GENE_SITE_141	ONE_NON-GENE_SITE_141
**CAGCAAGGATCAGTTTCCGG**	RHOC	389
**CAGCAATGAACACTTCACGC**	TCF7L2	6934
**CAGCACACCTTACCGCTACC**	PREB	10113
**CAGCACCATGACCACCCCAC**	RASSF2	9770
**CAGCACCCGCTTCTCAACCA**	PRKCD	5580
**CAGCACCCGGTAAAGGTCGG**	DNAJC9	23234
**CAGCACGGGCCAGGGAACCA**	FAU	2197
**CAGCACGGGCCAGGGAACCA**	FAUP1	140623
**CAGCAGCACAAACACTGTCG**	RASSF3	283349
**CAGCAGGCAGGCGAGAACGG**	DUSP2	1844
**CAGCATCGGATGCTTAGGAG**	MTOR	2475
**CAGCATCTCATACACACGCG**	TSC2	7249
**CAGCATTAAAGACGACTCGG**	PRKCZ	5590
**CAGCATTGCCTGGCACAGCG**	DCAF12L2	340578
**CAGCCACAGATACTATTAGG**	ITFG1	81533
**CAGCCATGCTGTGATACCGG**	RAD52	5893
**CAGCCCAAACTGGTACCCGT**	UBL5	59286
**CAGCCCGGAACTCAACAAAG**	EXOC7	23265
**CAGCCCGTGGCTTTACAGGG**	RASSF7	8045
**CAGCCCTGCTTTACAGGACG**	AKT1	207
**CAGCCTATTCAAGAGAATAA**	RALGAPA2	57186
**CAGCCTCTGAGAGCACACAA**	NAIP	4671

**CAGCCTGAATGTCAATAACG**	WDR26	80232
**CAGCCTTATACAGAACATAG**	PSMD3	5709
**CAGCCTTTCAACAAACCGAG**	PLXNB1	5364
**CAGCGGCCAGTCATAGAAGG**	BIRC7	79444
**CAGCGGCTGCCGGAACACTG**	PIK3CD	5293
**CAGCGGGAGCCCTTACACAT**	ERBB2	2064
**CAGCTCCCTCTGGTCGACCA**	TRAPPC2L	51693
**CAGCTCGTTAAGGATCAACA**	MTOR	2475
**CAGCTGACCCACTCACTGGG**	TUBB	203068
**CAGCTGCACCTGCTCGAGCA**	CNKSR1	10256
**CAGCTGGAACCGATTGAACG**	ONE_NON-GENE_SITE_142	ONE_NON-GENE_SITE_142
**CAGCTGGAACTACATGACAA**	YAE1D1	57002
**CAGCTGGCACATGCGAGATG**	RTN4R	65078
**CAGCTTGAAACATAGTCCAA**	ARID5B	84159
**CAGCTTGACAGACACTATGG**	RASGRP1	10125
**CAGCTTGATAGCTACAAATG**	APC	324
**CAGCTTGCCGATGTATCGAA**	VPS11	55823
**CAGGAACCTTAATCTCACCT**	RASA2	5922
**CAGGAAGTATGCCAGATGGA**	VTA1	51534
**CAGGACCAGCGAGCACCAGG**	VPS25	84313
**CAGGACCATTATTCATTCTG**	ST3GAL6	10402
**CAGGACGGTACTTACGTCTG**	CREBBP	1387
**CAGGAGAACACGTTCCCCAA**	SOX9	6662
**CAGGATGATTTGTTGAAGAC**	INIP	58493
**CAGGATGATTTGTTGAAGAC**	LOC112268470	112268470
**CAGGCAACTTAGCATTATGC**	ONE_NON-GENE_SITE_143	ONE_NON-GENE_SITE_143
**CAGGCAATTACCTTCTGGTG**	PRKAB1	5564
**CAGGCACAACCAGCCTGAGA**	MARCH2	51257
**CAGGCCATCAGGACAAGGTG**	BIRC7	79444
**CAGGCCTGGGGGAATATGCA**	PRKAA2	5563
**CAGGCGACGAGTTTGAACTG**	ABALON	103021294
**CAGGCGACGAGTTTGAACTG**	BCL2L1	598
**CAGGCGCAGCCCTCCAAGAA**	BIRC5	332
**CAGGCGTACCTTCACTCGCA**	RASAL1	8437
**CAGGCTGCGCGCAAAGACAG**	MYC	4609
**CAGGGAGTTAGAACGGAAGA**	RASAL1	8437
**CAGGGCTCCTGTCTACGACC**	DUSP2	1844
**CAGGGCTGTGTTATAATCTG**	EXT2	2132
**CAGGGGCTTATAGCCAGTGT**	PRKCSH	5589
**CAGGGGTCTCTGAACTTGCA**	AUP1	550
**CAGGGTACCATCTTCATACA**	POLR2H	5437
**CAGGGTGCAGAGGAATGCTG**	RASSF7	8045
**CAGGGTGTATTCATCCAGCG**	IRS2	8660
**CAGGTACATTATGACATGCA**	ELOVL1	64834
**CAGGTCCCGATCATTCATAG**	BLK	640
**CAGGTCGTAGCGGTGCGAGG**	QPRT	23475
**CAGGTGAAGACTTGGTGTGG**	UNG	7374

**CAGGTGGCATAACGGACCTG**	FGFR1	2260
**CAGGTGTAGAACTTGCAGGT**	SCD	6319
**CAGGTTGTACTCGTAAAACA**	RPS8	6202
**CAGTAACTTTGTGGCCCGAG**	BLK	640
**CAGTAAGGACGAGTTCCCCG**	RHOB	388
**CAGTAATCCAGAAACAGTCC**	EXOC5	10640
**CAGTACAACATCCATGTCTG**	RIC8A	60626
**CAGTATCCGCAATTAAGCCA**	ONE_NON-GENE_SITE_144	ONE_NON-GENE_SITE_144
**CAGTATGAGGAGTTCTACGT**	MCM3	4172
**CAGTCCCTGAAAGCATTACC**	CISD2	493856
**CAGTCCTTCTAACTACAAGT**	IFIT5	24138
**CAGTCGTAGACGAAGATCGA**	RPTOR	57521
**CAGTCTTCATTTGATCAAAG**	RAB3GAP2	25782
**CAGTGATGACGAGGACGAGG**	RANGAP1	5905
**CAGTGATGGTGAAACTTAAG**	SNRPF	6636
**CAGTGCAAGGCCATGAAGTG**	INPP5A	3632
**CAGTGCTTTAAACTACGCCA**	RABGAP1	23637
**CAGTGGAAGATAAATACCAT**	RASGRP3	25780
**CAGTGGCCAGCAAAGATGCT**	BUB1	699
**CAGTGGTCACCTCTACAACT**	IRF2	3660
**CAGTGGTGCATTCGTAGCTG**	CRYGB	1419
**CAGTGGTGTTGATGATGACA**	CASP3	836
**CAGTGTGATCCATTCCGCAG**	PRKCA	5578
**CAGTTCCATCGAACCGCCCA**	ONE_NON-GENE_SITE_145	ONE_NON-GENE_SITE_145
**CAGTTGAGCCGATTACAGAA**	RASA1	5921
**CATAAAGGACCCCCGATGGG**	SLCO2B1	11309
**CATAAGCTTCCTTCGTCCAG**	TBK1	29110
**CATACATGCAACTACCAACA**	RPS6KA6	27330
**CATACATTACATACTCAAAC**	POLR2H	5437
**CATACCCTGTAAAAAGTGTG**	ST3GAL6	10402
**CATACCGTCTAAATCAACAG**	NFE2L2	4780
**CATACCTGGCCTGAAGCACA**	RASAL1	8437
**CATACTCAAATAGTCGCAGA**	PPP1CA	5499
**CATAGACATCGCCATCGGAG**	LUZP4	51213
**CATAGACCTGGACCAGCCAG**	RPS6KB1	6198
**CATAGATAGGTAGCTCTGCT**	MCCC1	56922
**CATAGGAGGGAACACGCTGC**	ETS1	2113
**CATATTTCTTTCTGTACACC**	RPL26L1	51121
**CATCAAACCAACCAAACCAA**	SNX13	23161
**CATCAAGGCCAACGACACAC**	DUSP3	1845
**CATCAATGCAAACCACGCCT**	MCM3	4172
**CATCACACACTTTGTCCACG**	RASGRP2	10235
**CATCACGCTGCAGGACAGCT**	FNTA	2339
**CATCACTAACGCCAGCCAGT**	PIN1	5300
**CATCAGCTCCTGGTTCAACG**	DUSP1	1843
**CATCAGTGTGTCTGAACATG**	TSC2	7249
**CATCATGATCTTGTACACAA**	UGCG	7357

**CATCATGTTACACAAAATGG**	SPTLC2	9517
**CATCCAGGCCCACATCGACA**	PRKCB	5579
**CATCCCAATACCCCTCATGT**	DHX8	1659
**CATCCCCAGCGAAAAGAGTG**	PEA15	8682
**CATCCCTCCAATTGTAAACG**	ONE_NON-GENE_SITE_146	ONE_NON-GENE_SITE_146
**CATCCCTTTATAAACATATG**	ZFP90	146198
**CATCCGGCAGACGTACACGC**	FGFR3	2261
**CATCCGTCCAGATAACATGT**	HDAC1	3065
**CATCCTAGTCAACTCCCGTG**	MAP2K1	5604
**CATCCTGCTTCTTTAGCACT**	CHCHD3	54927
**CATCCTGCTTCTTTAGCACT**	CHCHD3P3	646572
**CATCGCGGTGATTGCCAAGG**	TRAPPC2L	51693
**CATCGGCCCGAGAATCACCT**	TK1	7083
**CATCTACAGAGACCTGAAGC**	RPS6KB1	6198
**CATCTCGCCGTTCCGCCTGG**	CCDC6	8030
**CATCTGGAAGTCATGCCCGT**	VAV1	7409
**CATGAACTCCTTCGACGACG**	RIMS4	140730
**CATGAGACCACTGTCTACGT**	NF1	4763
**CATGAGATAACGCTGAAGCA**	WDR70	55100
**CATGAGGTTACGATCCTGGG**	UBE2H	7328
**CATGAGTGGACAGCTCCCAT**	FAXDC2	10826
**CATGCCTGTTTACAAGAAAG**	TP73	7161
**CATGCGCAAGAGGAACCAGG**	RASGRF1	5923
**CATGCTCAGATCCCTACCAT**	ONE_NON-GENE_SITE_147	ONE_NON-GENE_SITE_147
**CATGCTTCCAACACTGAGAG**	PEX3	8504
**CATGGAATGAAACATGCCAC**	DPM1	8813
**CATGGACCATGTCATTGCAA**	RALBP1	10928
**CATGGATGACAAGCATACCT**	SLCO2B1	11309
**CATGGCATATTCTAACACTC**	MTOR	2475
**CATGGCATGTCCGAACGAGG**	TSC2	7249
**CATGGCCGTTTCTAGTTCGA**	ONE_NON-GENE_SITE_148	ONE_NON-GENE_SITE_148
**CATGGCGTAGTAGCGGCCAG**	AKT2	208
**CATGGGAGCGCAGCCATTTG**	LOC107985050	107985050
**CATGGGAGCGCAGCCATTTG**	TK1	7083
**CATGGGCCCAGCATCACACG**	PLXNB1	5364
**CATGGGTAGAACATGCCAAG**	BIRC2	329
**CATGGGTTCAACATGCCAAG**	BIRC3	330
**CATGGTCAAGAGAATCAGAA**	CDC25A	993
**CATGGTCTTGGATTGTGACG**	TBC1D3B	414059
**CATGGTGCCAGCCCAGAGAG**	XBP1	7494
**CATGGTTCTATAGAGCACTG**	RASSF6	166824
**CATGTACCATCTATCCACGG**	NO_SITE_1	NO_SITE_1
**CATGTACCCATTAGTAGACC**	ONE_NON-GENE_SITE_149	ONE_NON-GENE_SITE_149
**CATGTATACCCAAGTCAAGC**	ONE_NON-GENE_SITE_150	ONE_NON-GENE_SITE_150
**CATGTGACTTCCCAGCATTG**	KLHDC3	116138
**CATGTGCAATCACAGCAGCC**	CCND3	896
**CATGTGTACATCGGAACAGG**	SREBF2	6721

**CATGTTTCCCATATCCAACA**	EXOC1	55763
**CATGTTTGACTATGGGCTCA**	RABGAP1	23637
**CATTAGCAGACCGAAACAGG**	CNOT2	4848
**CATTCAGAAAGCCAGAACTT**	STK3	6788
**CATTCATATCCGAGCAGCAG**	NOX1	27035
**CATTCCAAAATGTGACAAGC**	SHC3	53358
**CATTCCTGAGCAGAATTGGT**	TFDP2	7029
**CATTCTGGGTGACACGCTGG**	PRKAA1	5562
**CATTGAGTACGTCAACGGCG**	PRKCZ	5590
**CATTGGAGAGCACATTGATG**	NO_SITE_2	NO_SITE_2
**CATTGGCAGCTAGGCGTTTG**	RTN4R	65078
**CATTGTAGTACACAGAGATG**	TUBB	203068
**CATTGTTACGTACAGTTTGG**	RAC3	5881
**CATTTATGTTATAACACGCA**	RIC1	57589
**CATTTCACATACAGACCTGA**	FUNDC2	65991
**CATTTCATTCCTGTACAGCT**	TGIF1	7050
**CCAAACTTTCCTTTACCCAG**	AURKA	6790
**CCAAAGAGTATTGTCGAGAG**	VPS18	57617
**CCAAAGCCTCCATAGCTCTG**	DDX46	9879
**CCAAATGTCCCTCGTGCGAG**	ONE_NON-GENE_SITE_151	ONE_NON-GENE_SITE_151
**CCAACAGTTGACCAATTGTG**	FANCC	2176
**CCAACGGCTCTTAGACCACA**	SPRY4	81848
**CCAACTACGACACCGACGAC**	MMP9	4318
**CCAACTTGATGGACTCCCGG**	LYN	4067
**CCAAGAAGTCCTCCCACTCG**	FAM217B	63939
**CCAAGAGAGTTAGCAAGTGC**	XIAP	331
**CCAAGCATCAACACCATGAG**	FOSL1	8061
**CCAAGCTCTACCTTCCCACG**	CDKN1A	1026
**CCAAGGAAAAAACTAACGAG**	MED11	400569
**CCAAGGCTGATTTATAGTAG**	PRKAG1	5571
**CCAAGTGGAACCCTACTGCA**	POLR2C	5432
**CCAATTCAGGACCCACACGA**	PTEN	5728
**CCAATTCAGGACCCACACGA**	PTENP1	11191
**CCAATTGCTTTCTCAGTGTG**	NOX1	27035
**CCACAAATAACAGCACGTCG**	TTC7A	57217
**CCACAAGCTGTAGAATGAAG**	EBP	10682
**CCACAATGTCCTGCACTCCA**	PHB	5245
**CCACACCTTGAACTCAGCGA**	TBCB	1155
**CCACAGTGGTCACAAAACGT**	PRKCB	5579
**CCACAGTTACCTCAGCAAAG**	RASSF3	283349
**CCACCGCATCGACTCCACCG**	STK11	6794
**CCACCGCCATCTTCCTCCTA**	SAP18	10284
**CCACCGCCATCTTCCTCCTA**	SAP18P2	442060
**CCACCGTCTGAAGAAGACGT**	RALGDS	5900
**CCACCTCACAGGATACACGA**	RGL2	5863
**CCACCTCATAGACAATGTAG**	ELOVL1	64834
**CCACCTTGATGGTCTCCATG**	SLC25A1	6576

**CCACGGTGGCGCGCACAGTG**	RGL3	57139
**CCACGTCACGCCAACAACAG**	CNOT2	4848
**CCACGTGGACAGGCTTACCG**	KSR2	283455
**CCACGTTGGTCTCACATCGA**	PRKCE	5581
**CCACGTTTCCGCTTGCCTCG**	ONE_NON-GENE_SITE_152	ONE_NON-GENE_SITE_152
**CCACTCCAGTCCAGGAAATG**	BPIFB2	80341
**CCACTCCTGCAGGTACACGC**	KSR1	8844
**CCACTCTTACCTGTTCATAG**	SMU1	55234
**CCACTGAAGCTCATGCGTCG**	PRKAG2	51422
**CCACTGACGGAAGTTGCAGA**	GLOD4	51031
**CCACTGGCAGAAGCTGCGAG**	DUSP5	1847
**CCACTTGCCCACCATATGGG**	ARSI	340075
**CCAGAACTCCAACCTGAGCG**	ALK	238
**CCAGAAGCAGGTTTGAAAGG**	RPS6KA6	27330
**CCAGAAGTAGTTAATCGTCG**	RPS6KA3	6197
**CCAGAAGTTGACGTAGAATG**	RIC1	57589
**CCAGAATCTATCTACCGCCG**	PRKCZ	5590
**CCAGAATGTCTGTAAACCTG**	RASSF5	83593
**CCAGACCTCAGGCGGCTCAT**	TP53	7157
**CCAGACGCGGTCGTAGAAAG**	PRKCSH	5589
**CCAGAGAATACATAACACCT**	APAF1	317
**CCAGAGCAAGAACCTGACGG**	IRS2	8660
**CCAGAGCATGCGCGCGCTTC**	UBE2Q2L	100505679
**CCAGAGCCCATACCTGGCAT**	B3GAT3	26229
**CCAGAGCCGCGTCTTCTCCG**	TRAPPC5	126003
**CCAGAGCGGAAGTACCGACG**	STAMBP	10617
**CCAGAGGTCACTTACTTGCC**	RHEB	6009
**CCAGAGTTTCATCTGCGACC**	MYC	4609
**CCAGATCCAACGTCTAACCT**	ONE_NON-GENE_SITE_153	ONE_NON-GENE_SITE_153
**CCAGATCCAAGGATGAAACA**	ITCH	83737
**CCAGATGGCACTTACACCCG**	CDK4	1019
**CCAGCAACGAAGAACAGGAA**	MAGEA12	4111
**CCAGCAACGCCCATTTCACC**	RUNX1	861
**CCAGCAATATGAATCCACAA**	PANX1	24145
**CCAGCACATCATCCCCCTGG**	PLEKHF1	79156
**CCAGCACGGATGATACCATC**	UBL5	59286
**CCAGCACGTCCGACAGCATG**	SLC30A1	7779
**CCAGCCAGCCATGCGCACCA**	TUBGCP2	10844
**CCAGCCAGTATATGCCAGGT**	EBP	10682
**CCAGCCCAAGCAACTGATTG**	SPRY1	10252
**CCAGCGCGCTACTTACAGTG**	RPS13	6207
**CCAGCTCAGGATCCACGAAG**	PIK3R6	146850
**CCAGCTTCCTGATCATGTGG**	VPS25	84313
**CCAGGACAGTGCTATCACCC**	RABGAP1	23637
**CCAGGACATGACGGATTACG**	SHC2	25759
**CCAGGAGCTACACACCTTCG**	FAU	2197
**CCAGGATATATCCAAAGACG**	NF1	4763

**CCAGGATCTGCCCATCCACG**	BIRC7	79444
**CCAGGCCATCTCGGAAACGC**	INSR	3643
**CCAGGCCGCACATTGCAGGT**	METTL5	29081
**CCAGGCTCACCTCTATAGTG**	HRAS	3265
**CCAGGGCACGCATTGACTGG**	SHC1	6464
**CCAGGGCAGACAGCTCATGG**	OSGIN1	29948
**CCAGGGCCCTGGAGACAAAG**	YAE1D1	57002
**CCAGGTAGTTCATGGCCAGG**	CCND3	896
**CCAGGTCTTGGGACGACAGT**	SPRY2	10253
**CCAGTACTTCCTGCGGCGTG**	CRYGB	1419
**CCAGTCATCACTGGTAGCAA**	PHB	5245
**CCAGTGGCAGAGCCTGATCG**	PAK4	10298
**CCAGTGTTTCTTCTGCTTCA**	BIRC5	332
**CCAGTTCAATGAGACCTTTG**	COX4I2	84701
**CCATAAGCACGGTCAGCTCA**	SPRY2	10253
**CCATACACAATCGTTTCCGC**	ONE_NON-GENE_SITE_154	ONE_NON-GENE_SITE_154
**CCATACCTTAAATACGTAGG**	ALK	238
**CCATAGTGAGTAGGTCAGGA**	PEA15	8682
**CCATATAGAAAATAATCCTG**	AURKA	6790
**CCATCAAAGGTGTCAAAGGC**	ETS2	2114
**CCATCAACAAGAGCCCCCAA**	INSRR	3645
**CCATCAGAATTGGCAAGAGC**	BIRC2	329
**CCATGAGTAGAGCTCGCACA**	NLRC3	197358
**CCATGATTAGAGTCCCAGCT**	CERS2	29956
**CCATGGAAACTTCAGCAAGC**	CD1E	913
**CCATGGTTTAGACACAACGG**	ONE_NON-GENE_SITE_155	ONE_NON-GENE_SITE_155
**CCATGTAAACCTGCTGTGCG**	LGALS7	3963
**CCATGTAAACCTGCTGTGCG**	LGALS7B	653499
**CCATGTCACTGAGTTCCAGA**	FAXDC2	10826
**CCATGTTGACTGCCAACTTG**	TUBB	203068
**CCATGTTGACTGCCAACTTG**	TUBBP9	442210
**CCATGTTTGGACCCGCTCCA**	CECR2	27443
**CCATTAGCAAACACGTCCAG**	RASGRP1	10125
**CCATTAGCGCATCACAGTCG**	CDKN1A	1026
**CCATTGTTCAATATCGTCCG**	TP53	7157
**CCATTGTTTATGGTCAGCCT**	SPTLC2	9517
**CCCAAATCTTCCTTGCTCAG**	SPRY2	10253
**CCCACAGGCATATGGCATCG**	DPF1	8193
**CCCACCAGCATTTCGTGGGA**	PEBP1	5037
**CCCACCAGCATTTCGTGGGA**	PEBP1P1	326614
**CCCACCAGGGACATTGCCCG**	ONE_NON-GENE_SITE_156	ONE_NON-GENE_SITE_156
**CCCACGTGCCGGAAGCCAAG**	FUNDC2	65991
**CCCAGAAAAAGATTCAGCCT**	ARID5B	84159
**CCCAGCACGGATGATACCAT**	UBL5	59286
**CCCAGCGCATCCGGCGACAC**	RASSF4	83937
**CCCAGCGCCTGGACACTCGA**	PRKCG	5582
**CCCAGCGCGCTACTTACAGT**	RPS13	6207

**CCCAGCTACACTCTGAATAG**	CHD2	1106
**CCCAGCTCCACATCACCCCA**	ABALON	103021294
**CCCAGCTCCACATCACCCCA**	BCL2L1	598
**CCCAGCTGCCAACACCAAGG**	RPS8	6202
**CCCAGGACCCGCATTCAAAG**	IRS1	3667
**CCCAGGTCCGCGCCGTCGGG**	NRTN	4902
**CCCAGGTCGAACATATCCAG**	DHX8	1659
**CCCAGTGAAGTAAAACCCAG**	NAIP	4671
**CCCAGTGGTTCCCAGAAGTG**	RAC2	5880
**CCCATAAAGTTGGCTAACGC**	ONE_NON-GENE_SITE_157	ONE_NON-GENE_SITE_157
**CCCATCTGCAAAATCCCGGA**	FOSL1	8061
**CCCATGCAGAAGTTCTCACC**	CHSY1	22856
**CCCATTACCATGTAGCCTCT**	SEC23B	10483
**CCCCAGCCCCACCATGTCCG**	SOX9	6662
**CCCCAGGTGACCCATCAAGG**	FAM71E2	284418
**CCCCCAAGCCGGCTACCTGG**	VKORC1L1	154807
**CCCCCAGCACCATCCTTCCA**	IFITM1	8519
**CCCCCCGGGGTTATAAGGTG**	ONE_NON-GENE_SITE_158	ONE_NON-GENE_SITE_158
**CCCCCGGAGGCACTTGACTG**	AGPAT3	56894
**CCCCGAAGAAGCAGCGCTTG**	PRKCG	5582
**CCCCTACTCGCCCTACGTGG**	GATA6	2627
**CCCCTCTGAAAGGCCACGAG**	VPS39	23339
**CCCCTGAGTCGGCTTGACAC**	ONE_NON-GENE_SITE_159	ONE_NON-GENE_SITE_159
**CCCCTGTTGGCGGCAGCGCA**	DUSP2	1844
**CCCCTTCGGACCCAGACAGG**	HDAC11	79885
**CCCGAAGAAGAAACAACAGA**	CISD2	493856
**CCCGAAGAAGAAACAACAGA**	SLC9B1	150159
**CCCGACGGAATATCGAGCGA**	RASAL3	64926
**CCCGCAGTGCTATTGCATCG**	RASSF10	644943
**CCCGCGCTGTGCGCCAGGAA**	DUSP4	1846
**CCCGGCACCTACTCTGCGCG**	C4orf48	401115
**CCCGGCGTACAGCCTATCCG**	ONE_NON-GENE_SITE_160	ONE_NON-GENE_SITE_160
**CCCGGCTGAGAGAATACCCA**	FANCA	2175
**CCCGTAGTCTCCGATGCGCA**	LMTK3	114783
**CCCGTATGGCTCAACTTCGA**	VHL	7428
**CCCGTCCATCGAAACACCGT**	PRKAB1	5564
**CCCTACCCCACAGATCCCTG**	RASAL3	64926
**CCCTATGAGCCACCACAGGT**	TP73	7161
**CCCTCAAACCCCACGTGACG**	ONE_NON-GENE_SITE_161	ONE_NON-GENE_SITE_161
**CCCTCAAAGGGGGAATCCTG**	UBE2N	7334
**CCCTCAAAGGGGGAATCCTG**	UBE2NP1	100288613
**CCCTCAACCTGGACATCACA**	BPIFB2	80341
**CCCTCCAAGCCACAAAACTG**	SPTLC1	10558
**CCCTGACCACCGGCCCAAAG**	SPRY4	81848
**CCCTGACTACCAGCAATGGA**	CRYGB	1419
**CCCTGCAGAGCAGCTATACG**	ARHGAP35	2909
**CCCTGCCTCCAATCAAACCT**	ARID5B	84159

**CCCTGCTCAAGCGAAAGCAA**	ARSI	340075
**CCCTGGGGACTATGTGCTGT**	CRKL	1399
**CCCTTTATGACTATGAAGCA**	FYN	2534
**CCGAACCTTTCTGGGCTGGG**	RGL3	57139
**CCGAAGCACTAGATCGCCGT**	IRS1	3667
**CCGACAATACTAAGCCCCAG**	SCRIB	23513
**CCGACCCCAAGGACCACCAG**	SLC30A1	7779
**CCGAGCGCATGTACTCGTTC**	ELK1	2002
**CCGATCGCACACATTTGTCG**	MET	4233
**CCGATGCCCGAGTTGCAGGT**	HDAC7	51564
**CCGATGGTATCATCCGTGCT**	UBL5	59286
**CCGATTACACCTCAACAAGA**	UGCG	7357
**CCGCAGACTCAAGGATACAG**	NLRC3	197358
**CCGCAGCCCTGGATCGCCTG**	HDAC10	83933
**CCGCCACTATGAGAGCCGAA**	SPNS1	83985
**CCGCCCCTCTAGCCTAGGCG**	DPF1	8193
**CCGCCGGGCATCCTGCAAGG**	RASSF1	11186
**CCGCGAGTGCAAGCGCAAGG**	CDC37	11140
**CCGCGCTGGAAAGGAACGCC**	LGALS7	3963
**CCGCGCTGGAAAGGAACGCC**	LGALS7B	653499
**CCGCTATATCGGCCACCTGT**	MESP1	55897
**CCGCTATGGTTACACTCGGG**	MMP9	4318
**CCGCTGCAACTCCATCAGGG**	CRYGB	1419
**CCGCTGGCGTTATGGTATAG**	ONE_NON-GENE_SITE_162	ONE_NON-GENE_SITE_162
**CCGCTTAATTTGGCGACGTG**	ONE_NON-GENE_SITE_163	ONE_NON-GENE_SITE_163
**CCGCTTTCTACCGCCGTCCG**	MESP1	55897
**CCGGAAGCGCGCGCATGCTC**	UBE2Q2L	100505679
**CCGGAAGTCCTTATTAAGAG**	INIP	58493
**CCGGCCACTCACCCACAGAC**	RHEB	6009
**CCGGCGCAACCTAAACAGTG**	SNX13	23161
**CCGGCTCCCATGCGGCAACC**	ONE_NON-GENE_SITE_164	ONE_NON-GENE_SITE_164
**CCGGCTGCTCGGGTGCGAAG**	GATA6	2627
**CCGGGCAGGTTCAACAACCC**	C9orf116	138162
**CCGGGGGTTGAAATGCAGGG**	LGALS7B	653499
**CCGGTTCAGATAATCAAACA**	ACVR1B	91
**CCGGTTCGCCGGTCACAGCG**	PAK4	10298
**CCGTCTTTCCTGCGTAACAG**	HDAC4	9759
**CCGTGGAGTGCAGGACATTG**	PHB	5245
**CCGTGTGGGTATATGACTCG**	ONE_NON-GENE_SITE_165	ONE_NON-GENE_SITE_165
**CCGTTCCAGATGCTCTGACT**	SLBP	7884
**CCTAAATGGCATAGTTCACA**	CAMK2D	817
**CCTAACTAGTTGGTAACCGT**	PREB	10113
**CCTAAGTTCTCGGTAACACC**	PANX1	24145
**CCTACAAGCAAGCTGAATGG**	RIC1	57589
**CCTACTACTGTAGCCAACAA**	PIK3R1	5295
**CCTACTGCCAGAGAACCCTG**	MAPK1	5594
**CCTATCAGTGTAGTCTCTGT**	ZFP90	146198

**CCTCAAAGGGGGAATCCTGA**	UBE2N	7334
**CCTCAAAGGGGGAATCCTGA**	UBE2NP1	100288613
**CCTCACAGATCACCATAAGG**	PLCXD3	345557
**CCTCACGCACCTGAGCCCCG**	XBP1	7494
**CCTCACTTCATTCAGGGTGT**	RPS18	6222
**CCTCATCTACTTGAACAGGT**	BIRC3	330
**CCTCCACTATCGAACCAGCA**	RGL3	57139
**CCTCCACTCACAAGATCGTG**	FANCA	2175
**CCTCCAGACAGGCCTCCAAG**	ELK1	2002
**CCTCCATGAAGAGACATGCA**	DDX46	9879
**CCTCCGAAGAGTCCACGGCT**	E2F1	1869
**CCTCCGTAGGGACCATCCCG**	RCE1	9986
**CCTCCTCAAAGGCTTTACGA**	VCP	7415
**CCTCCTCACCTCGCCCGACG**	JUN	3725
**CCTCCTCCAAGCATCAGTAA**	HDAC2	3066
**CCTCGAAGGCCGCAGCGATA**	VPS11	55823
**CCTCGACTCGCAGACCGGCG**	DPF1	8193
**CCTCGTAGTGATCGATCTCG**	RASSF10	644943
**CCTCGTGTGACGCAAATCGA**	ONE_NON-GENE_SITE_166	ONE_NON-GENE_SITE_166
**CCTCTACAGGAACTTTGCCG**	TSC2	7249
**CCTCTATCTTTGCCTCTGAG**	HDAC9	9734
**CCTCTTACCACAATAGCATG**	WDR26	80232
**CCTGAAGCAGGGCTCGAACC**	SLC25A1	6576
**CCTGAAGTAGTAAATAGGAG**	RPS6KA6	27330
**CCTGACAACTGAGACCAGCA**	EXOC3	11336
**CCTGATACTTACTTGCAGTG**	POLR1A	25885
**CCTGATGTTCTCATCCCATA**	ST3GAL6	10402
**CCTGCACTCGGAGAAGAACG**	AKT1	207
**CCTGCCAGAAGCGCACGGTG**	MLST8	64223
**CCTGCCCTCGATTTCTAGCG**	CD1E	913
**CCTGCGAGTACAAGATCGGT**	DPF1	8193
**CCTGCGTTCTCAAAGAGATT**	DUSP6	1848
**CCTGCTTGAAGGTGCTGCTG**	C12orf49	79794
**CCTGGACCGCTTCCTGTCGC**	CCND1	595
**CCTGGACCTACCTGACAGCG**	GRB10	2887
**CCTGGACGCCCCTGACAACG**	FAM71E2	284418
**CCTGGACGGAGCATGCCAAG**	BIRC7	79444
**CCTGGATCCATTCCATGGAG**	EXOC8	149371
**CCTGGATCTTTACTACACTG**	RASGRP1	10125
**CCTGGCCCCTCAGATCAAGA**	PRKAG3	53632
**CCTGGCGGGAGACCCTGCCG**	LMTK3	114783
**CCTGGCTCCGGAATTCACTG**	PTGS1	5742
**CCTGGTAAACTCCATCCACT**	UAP1	6675
**CCTGGTGCCTGAGCTCGATG**	VPS16	64601
**CCTGTAAGCTGTAGGGAACG**	VPS11	55823
**CCTGTAGAGAAGCCTCCCGG**	SREBF1	6720
**CCTGTCACATTGCTGCATTA**	STK3	6788

**CCTGTCCAAAACATACCTTG**	SET	6418
**CCTGTCCATCCTTAAGCCAG**	FGFR4	2264
**CCTGTCGAAAGCTCTTCCAA**	CBL	867
**CCTGTGCTAGCGCTTGACGT**	ONE_NON-GENE_SITE_167	ONE_NON-GENE_SITE_167
**CCTGTTAGCAAATCGAGCAC**	ONE_NON-GENE_SITE_168	ONE_NON-GENE_SITE_168
**CCTGTTCCTCCAGTTACAGC**	DTYMK	1841
**CCTTACCAAGAGAAACCAGG**	ZFP90	146198
**CCTTATAATCAGGACCAAGG**	JAK3	3718
**CCTTCAACACAATACACCCG**	APC	324
**CCTTCAACAGCAACCCAAGA**	PAK3	5063
**CCTTCAGGCTCTGTTCACTG**	PPP1R7	5510
**CCTTCATGGAGAAATGCCGT**	TFDP1	7027
**CCTTCCAGAAGAATACCCAA**	UBE2N	7334
**CCTTCCAGTTGGCTACAATG**	KLHDC3	116138
**CCTTGACCGAGTGAAACTCA**	PRKCA	5578
**CCTTGACTAAGGCAGAATGC**	SLC11A2	4891
**CCTTGAGCAGGCAACACCCT**	RANGAP1	5905
**CCTTGCGGATGGGCATGGAG**	RPL26L1	51121
**CCTTGGCAACACAAAAGGGG**	BPIFB2	80341
**CCTTGTTCAGTAACACGCGT**	ONE_NON-GENE_SITE_169	ONE_NON-GENE_SITE_169
**CCTTGTTCTCCACCAAAGCA**	CDC6	990
**CCTTTCAAGTCGTACCAGAT**	ONE_NON-GENE_SITE_170	ONE_NON-GENE_SITE_170
**CCTTTCAGAGTCCTGTTGGT**	CNKSR1	10256
**CCTTTCCAGTGTAAAAAATG**	ZNF429	353088
**CCTTTCTCAGCATGTCAGGG**	TGIF1	7050
**CCTTTGAGGAAGTAGACCCG**	DUSP5	1847
**CGAACAGAGTCCATGGTCCC**	TUBB	203068
**CGAACAGAGTCCATGGTCCC**	TUBBP1	92755
**CGAATCGGCGAATCGACCTG**	PEX16	9409
**CGAATGCAGAGAGGTTGAAA**	SPC25	57405
**CGACAAGTTCTACAGCAAGC**	TBCB	1155
**CGACCAGCTTCGCAATCATG**	MCCC1	56922
**CGACCGCACAGCCATCCACG**	MCM7	4176
**CGACGACACCGTCAGCCGAG**	E2F1	1869
**CGACGGACTTGGAATCCAGC**	INPP5A	3632
**CGACTACTACCGCGTGAGCG**	C9orf116	138162
**CGAGAATGAGAACATCACCG**	CHCHD3	54927
**CGAGACTACGACCCTCGGCA**	HCK	3055
**CGAGAGATGAATATGCCCTA**	SCRIB	23513
**CGAGAGCTCCGCCAAGAACG**	TP73	7161
**CGAGCTCAAGACCTACCACG**	PRPF38B	55119
**CGAGCTGAGCTTCAAAAGGG**	GRB2	2885
**CGAGCTGTACCAGCGCGTAG**	B3GAT3	26229
**CGAGGAGAGCAGAGAATCCG**	MYC	4609
**CGAGGAGTCACTGAACACTA**	SPTLC1	10558
**CGAGGCCGGGATGAGTTGGG**	CDKN1A	1026
**CGAGGCTGGGCCATCAACGT**	HDAC11	79885

**CGAGGGCGATGACAAGCCAG**	PEX16	9409
**CGAGGTGATAGTGGTCTGTG**	SPRED2	200734
**CGAGTATTACAATGTGCCGG**	PRKCG	5582
**CGAGTCCTGGATGCTCTCTG**	MESP1	55897
**CGAGTCGTCGCACATCGAGT**	DUSP6	1848
**CGAGTGTGTGCATGTCACCG**	PIK3CD	5293
**CGAGTTACACGAGCCTTGCG**	SPTLC1	10558
**CGAGTTGAACTGAACCCGTG**	FGR	2268
**CGATCAGACAACAACATGGC**	YAP1	10413
**CGATCATACTGGGAGATGCG**	PTK2	5747
**CGATCCCACAGCATGCATGT**	FANCA	2175
**CGATCTTCTGTCCATCGAGG**	RGPD5	84220
**CGATCTTCTGTCCATCGAGG**	RGPD6	729540
**CGATCTTCTGTCCATCGAGG**	RGPD8	727851
**CGATGACAACACCTACAGCC**	B3GAT3	26229
**CGATGAGCCCCACAGAGCTG**	E2F2	1870
**CGATGATGTTGAGTGCACGA**	PRKCQ	5588
**CGATGCCCACGATGACCGAA**	SLCO2B1	11309
**CGATGCCTTAAACGTGCTAA**	TFDP1	7027
**CGATGGCAGTTTCATAGTAT**	RALGAPA1	253959
**CGATTCAACAGACTTACTCC**	BRIP1	83990
**CGCAACACACCTGGGCGATG**	RGL3	57139
**CGCACAGGTCACACCAGCCG**	RASSF5	83593
**CGCACGCCCTACCTTGTACG**	MAEA	10296
**CGCAGCCGAACACAAACCCC**	ICMT	23463
**CGCATGTGTTATAACGATCG**	ONE_NON-GENE_SITE_171	ONE_NON-GENE_SITE_171
**CGCATTCAGATTCGCCGCCG**	MZT1	440145
**CGCCATCCCCACCCGCACCG**	EIF4EBP2	1979
**CGCCGCATCCACAGCTACCG**	VHL	7428
**CGCCGCGCCTACCTACGGAG**	MESP1	55897
**CGCCGGCACCTCACGCTCTG**	SIRT1	23411
**CGCGGACCTTCTTCCCCAGA**	UBL5	59286
**CGCGGACGAGGAACATACCG**	CRKL	1399
**CGCGGCGGCGAATCTGAATG**	MZT1	440145
**CGCGGGGACGGCACTCCCGG**	C4orf48	401115
**CGCTCGCACACCCTGAGCGG**	IRS2	8660
**CGCTGAAGGAGATCCAATGG**	SPRY4	81848
**CGCTGAGACTTCGAACCAGA**	CHCHD3	54927
**CGCTGAGACTTCGAACCAGA**	CHCHD3P3	646572
**CGCTGTGTGGTAACTCAGTG**	NSD2	7468
**CGCTTCAGTGCAACACGTGG**	MED11	400569
**CGCTTGTTTAGCCCTGAACC**	TSPAN13	27075
**CGGAAACTTTCGGAGCGAGG**	TCF7L2	6934
**CGGAAGTATCTCATGTGCGG**	SPNS1	83985
**CGGAAGTCCTTATTAAGAGA**	INIP	58493
**CGGAAGTGGCTACCACCCGA**	ERF	2077
**CGGACGACTGCTGTTCAATG**	UAP1	6675

**CGGAGCTGAGCCGAGACTTG**	ETS2	2114
**CGGATGATGTCCAGCACGCG**	MTOR	2475
**CGGCAGGTTACAACCGAATG**	VPS25	84313
**CGGCAGTTCAGAGTACACCA**	SUZ12	23512
**CGGCCAAGCATCAATCAGGT**	TIAM1	7074
**CGGCCAAGTGCAGGAACCGG**	FOSL1	8061
**CGGCCCGGAAGAGTCCGGCC**	VHL	7428
**CGGCCCGGCAGATACCTGAG**	DHFR	1719
**CGGCCCTGAAGAAGACGGCG**	VHL	7428
**CGGCCGCCTGACCTGCACCA**	TMEM220	388335
**CGGCCGCCTGACCTGCACCA**	TMEM220-AS1	101101775
**CGGCCTGAGATGTGTACAGA**	CTNNBL1	56259
**CGGCGAATCTGAATGCGGTG**	MZT1	440145
**CGGCGGCGGCGGTGACATCG**	C4orf48	401115
**CGGCGTACAGCCTATCCGCG**	ONE_NON-GENE_SITE_172	ONE_NON-GENE_SITE_172
**CGGCGTCTTTGACGTCCTTG**	EXOC3	11336
**CGGCTACGTCACCAACTCCA**	TRAPPC2L	51693
**CGGCTGGTAAAAAGTCGCGA**	ONE_NON-GENE_SITE_173	ONE_NON-GENE_SITE_173
**CGGGAAAGCGAGACCACCAA**	FGR	2268
**CGGGAAGGACTGCGGCACAA**	RSPH3	83861
**CGGGACGCCTCAGCTCGACA**	GATA6	2627
**CGGGCAACGACACCCCATCC**	DDIT4	54541
**CGGGGAGCGCCACGGCGCCA**	PRKAB2	5565
**CGGGGAGTACACTCCCCCTG**	LMTK3	114783
**CGGGGCAGAGGGGGTGCTGT**	B3GAT3	26229
**CGGGGTGAGCAGGAGCAGCG**	CD1E	913
**CGGGGTTGCCAGGGTCCACG**	SPNS1	83985
**CGGGTATCCCTTCGACGGGA**	MMP9	4318
**CGGGTCAGGAGGACTACGAT**	RAC3	5881
**CGGGTGAGTGGTAGTAAGAG**	FOS	2353
**CGGGTGCAAATGGCGCGCTA**	MFSD11	79157
**CGGGTGCAAATGGCGCGCTA**	SRSF2	6427
**CGGTATCCAGGATGTCCAAC**	HRAS	3265
**CGGTATGCAGTGTGCGCTGC**	CCT6P3	643180
**CGGTATGCAGTGTGCGCTGC**	LOC441241	441241
**CGGTATGCAGTGTGCGCTGC**	VKORC1L1	154807
**CGGTCGCCCAGATCAAGGTA**	FAU	2197
**CGGTGGGGATGTACTCTCCG**	RAC3	5881
**CGGTTCTCTGCGGTGAAGGG**	RPS6KB2	6199
**CGGTTTTGGAATATTCGGCG**	ONE_NON-GENE_SITE_174	ONE_NON-GENE_SITE_174
**CGGTTTTGGAATATTCGGCG**	INACTIVE_4T_3	INACTIVE_4T_3
**CGTAGACATTAGCATCAAGC**	JAK3	3718
**CGTCACAATCCAAGACCATG**	TBC1D3B	414059
**CGTCACTGGAGGCGCGGACA**	IRS1	3667
**CGTCCAGCAACACCACGGCG**	DUSP1	1843
**CGTCCATATGGTCAGCACCA**	ARAF	369
**CGTCCGAGACCTCTGGATAG**	RPTOR	57521

**CGTCCGCTGGACAAGCACGA**	PDCD6IP	10015
**CGTCGGAAGGTCGAACAGGA**	MYB	4602
**CGTCTACTCCGTCACTGACA**	MRAS	22808
**CGTCTGGAAGGCAACACAGT**	RANGAP1	5905
**CGTCTTCCCCCCCGGACACA**	PRKAG2	51422
**CGTGAACTACTTCATAGATC**	TRIM64	120146
**CGTGAACTACTTCATAGATC**	TRIM64B	642446
**CGTGCACATTTCACTCCCCC**	TSC1	7248
**CGTGCCTCCGTAGGTCTGCG**	CCND1	595
**CGTGCTCACTTGAACCACGA**	LMTK3	114783
**CGTGCTGAATCACTGATGGC**	ARID5B	84159
**CGTGGATCTAGAGCTGAAGA**	PFDN1	5201
**CGTGGCAAAGCGTCCCCGCG**	BCL2	596
**CGTGGCCACTTGGCTCTGGG**	ANAPC11	51529
**CGTGGCCGGCCACGGCAACG**	PIK3CG	5294
**CGTGTACAACCTCGTCCCCA**	MCM4	4173
**CGTGTCGGAAAACCGGCTGG**	SCRIB	23513
**CGTGTGCTGCTTGGTCACTT**	PRKAG2	51422
**CGTTATAAGGGGTTGACGGG**	ONE_NON-GENE_SITE_175	ONE_NON-GENE_SITE_175
**CGTTGACAGAGCCGGCGATG**	DUSP1	1843
**CTAAAGTTCTGCTTCTGTCG**	XBP1	7494
**CTAATATCTGGATCTCGAAG**	CD1E	913
**CTAATCAGAGTCATCGGGCG**	PRKCZ	5590
**CTACAACAAGCACACCAAGG**	HCK	3055
**CTACAACGGGCACACGAAGG**	LCK	3932
**CTACACCGAGACAACAACGT**	RASAL2	9462
**CTACACGTGTGAGACAGATG**	ACVR1B	91
**CTACAGGCCAGCTTATGACC**	DUSP5	1847
**CTACATCCAGAAGATCAAGT**	PIN1	5300
**CTACATCCATCCACTCTGGA**	GINS2	51659
**CTACATCTGTAAGCATGGAC**	PRKAA2	5563
**CTACATGTTTAGTTTCAGAA**	CISD2	493856
**CTACATGTTTAGTTTCAGAA**	SLC9B1	150159
**CTACCATAGTCCAGTGGCTC**	PLCXD3	345557
**CTACCCAGAAGCTAGAAAGA**	SAP18	10284
**CTACCCAGAAGCTAGAAAGA**	SAP18P2	442060
**CTACCCCGATCCCCTCATCA**	RPS4X	6191
**CTACCTAAACACTAGAAAGG**	CASP8	841
**CTACGACCGAAAGTTCCTGC**	ANKHD1-EIF4EBP3	404734
**CTACGACCGAAAGTTCCTGC**	EIF4EBP3	8637
**CTACGTCACATGCCAAACTT**	ONE_NON-GENE_SITE_176	ONE_NON-GENE_SITE_176
**CTAGAAAGATCAACTGAAAT**	DTYMK	1841
**CTAGAGATTCATTCCGGTAG**	PIK3R1	5295
**CTAGATGAATATATGCATGG**	PHAX	51808
**CTAGATGCAGTTAAGACTTG**	GMDS	2762
**CTAGCACCTTGGTCTCACGC**	TRAPPC5	126003
**CTAGGAACTATCGTGAGCAT**	PAK2	5062

**CTAGGTAACTCTATAAGACC**	ONE_NON-GENE_SITE_177	ONE_NON-GENE_SITE_177
**CTAGGTCAGTCTACGACCAG**	ONE_NON-GENE_SITE_178	ONE_NON-GENE_SITE_178
**CTAGTCGCAAAGATTCTCGA**	YES1	7525
**CTAGTTCTCAGGTCCTAGTG**	ARHGEF2	9181
**CTATACGCCACTACCCCCGG**	EIF4EBP3	8637
**CTATACTGCACTATCTACGT**	ONE_NON-GENE_SITE_179	ONE_NON-GENE_SITE_179
**CTATATTCCTTCTAACTACG**	FRK	2444
**CTATCTTGTCAAGATATGAG**	CBL	867
**CTATGACCTCGACTACGACT**	MYC	4609
**CTATGCTCTGAAAAAGTACG**	EXT2	2132
**CTATGCTGTCGCCATCCCAC**	DPM2	8818
**CTATGTCTGGAATCTGACGG**	MLST8	64223
**CTATGTGGCAGATATCGAGG**	RHOA	387
**CTATTGGTGGTTGACACAAC**	FAXDC2	10826
**CTCAACGACCTGCTCTCGGA**	DUSP3	1845
**CTCACACAGTCACCGAGAGC**	AKT2	208
**CTCACACCTTCACCTACACG**	MED12	9968
**CTCACCACGGAAGTCAAGTG**	SLC50A1	55974
**CTCACCATGGTCAGCAACAG**	RCE1	9986
**CTCACCCAGTGACAACTCAG**	AKT1	207
**CTCACCCGAGTGCTCGCCGG**	SOX9	6662
**CTCACCTGACACACCCCTGA**	FGR	2268
**CTCACGGCTCCAGAGTGAGT**	AGPAT3	56894
**CTCACGGGGTTCACCTGTAC**	HRAS	3265
**CTCACGTGGAGCCGACCGAC**	ONE_NON-GENE_SITE_180	ONE_NON-GENE_SITE_180
**CTCACTCACCTCCAAGCATG**	FAU	2197
**CTCACTGATATCGAATGCAA**	MET	4233
**CTCAGAAGACAGGAATCGAA**	BRAF	673
**CTCAGCCGCGTTCAGCACAT**	DUSP3	1845
**CTCAGGCAGCAGAACAAGGA**	KPNB1	3837
**CTCAGGCTCACCTCGGTGCG**	TRAPPC5	126003
**CTCAGTCAGACAGATCTCAA**	LUZP4	51213
**CTCATCCTTGAGTATCTCAG**	LOC100287072	100287072
**CTCATCCTTGAGTATCTCAG**	LOC100996361	100996361
**CTCATCCTTGAGTATCTCAG**	RPS6KB1	6198
**CTCATCGTCGTCAACACAGT**	SLC50A1	55974
**CTCATCGTGGGTGACCAGAG**	MLST8	64223
**CTCATCTTGGATCTGATCAG**	EXOC7	23265
**CTCATGACTCACTTTGCCAA**	RPS6KA1	6195
**CTCCAAGTGGTCGAAAAGCA**	RASGRP4	115727
**CTCCACCAAGGGAACTTCAA**	GRB10	2887
**CTCCACGCAGGAGATCGGTG**	RALGDS	5900
**CTCCACTCCATCTCTGCCAG**	MYB	4602
**CTCCAGACAGCTCTTATGCA**	MARCH2	51257
**CTCCAGAGCAGCACGCACAG**	RCE1	9986
**CTCCAGGTGGTCAAACAACA**	RASGRP2	10235
**CTCCAGTGCTGCCCAACCAG**	SHOC2	8036

**CTCCATTGCTGTCGAGTACG**	ONE_NON-GENE_SITE_181	ONE_NON-GENE_SITE_181
**CTCCCAGCGCTCCGACTCCG**	LONRF1	91694
**CTCCCGAGACAGGAGTACCG**	PDGFRA	5156
**CTCCCGTGTCTAGCCCAGTG**	PDGFRB	5159
**CTCCGAGTGGGAACTAACAC**	ONE_NON-GENE_SITE_182	ONE_NON-GENE_SITE_182
**CTCCGATTCAGTCCCTTCTG**	ABALON	103021294
**CTCCGATTCAGTCCCTTCTG**	BCL2L1	598
**CTCCGATTCTCGGCAATGTG**	ONE_NON-GENE_SITE_183	ONE_NON-GENE_SITE_183
**CTCCGCTTCTACCGTTCACG**	SPRED3	399473
**CTCCTCATAAACTTCCCTCA**	NUDT4	11163
**CTCCTCATAAACTTCCCTCA**	NUDT4B	440672
**CTCCTCATAAACTTCCCTCA**	NUDT4P2	170688
**CTCCTCGGCCAGGCGCTGGG**	TBCB	1155
**CTCCTGCCATGCTACGTCAT**	ONE_NON-GENE_SITE_184	ONE_NON-GENE_SITE_184
**CTCCTGTGAAAAATCTGCCG**	KDM3B	51780
**CTCCTTGCTGGCGATCAATG**	ONE_NON-GENE_SITE_185	ONE_NON-GENE_SITE_185
**CTCGCACAGTCAAGCCAGCG**	GAREM1	64762
**CTCGGCGAGTAAGTCCATCA**	MCM5	4174
**CTCGGCTGGGAAGAAGCGTG**	FAM217B	63939
**CTCGGGCCGTGTCCAGTAAG**	FGFR3	2261
**CTCGGGGCAGGAAATACTTG**	DPM2	8818
**CTCGGTATGACACTTCGCTG**	E2F2	1870
**CTCTAATTGCAGATGGAGGA**	MCCC1	56922
**CTCTACGGGCTTGTGCACCA**	PRKCG	5582
**CTCTACTACGAAGACCTGCT**	EBP	10682
**CTCTAGTTGAAGTCACCCTG**	MAGEA3	4102
**CTCTAGTTGAAGTCACCCTG**	MAGEA6	4105
**CTCTAGTTTGGTGATATTGG**	PAK2	5062
**CTCTGACGGCTATCCAAAGA**	SPRY1	10252
**CTCTGAGCCATACCTATCCG**	SIRT1	23411
**CTCTGCACTTTGGTCGCTGG**	ONE_NON-GENE_SITE_186	ONE_NON-GENE_SITE_186
**CTCTGCCTTATATAATGGTG**	PHB	5245
**CTCTGGAAGACTTTAGCCGC**	ONE_NON-GENE_SITE_187	ONE_NON-GENE_SITE_187
**CTCTGGGGGGATCAGACTGG**	NPRL3	8131
**CTCTGTGGCTGGGAACTCCG**	PRKAG3	53632
**CTCTTACCGCAATGGTAAGC**	ONE_NON-GENE_SITE_188	ONE_NON-GENE_SITE_188
**CTCTTAGCCCCCATTCACTG**	RPS6KB1	6198
**CTCTTCTCCACCACACCGGG**	EIF4EBP2	1979
**CTCTTGAGTACTTGCACTCG**	AKT2	208
**CTCTTGATATCATTAATGCG**	SMC1A	8243
**CTCTTGCTACCATTGTCCCG**	RASA3	22821
**CTGAACAAACTCAGCAAGCG**	USP7	7874
**CTGAACGGGTCACCAGTCTA**	ONE_NON-GENE_SITE_189	ONE_NON-GENE_SITE_189
**CTGAAGCAGGCTTGAAACGG**	RAPGEF2	9693
**CTGAAGCTTTGGAAAGCGTG**	RASSF9	9182
**CTGAAGGATAGGCATTGTAA**	TGIF1	7050
**CTGAAGTCCGGGAACCCGCG**	CCNA2	890

**CTGAATGTGACATCTACAGG**	MAF1	84232
**CTGAATTAGCTGTATCGTCA**	KRAS	3845
**CTGACAGAGAACGAGATCCG**	PPP1CA	5499
**CTGACAGCGAATCATAACAT**	PTPN11	5781
**CTGACATTCCTGAGCCGAGT**	LOC441488	441488
**CTGACATTCCTGAGCCGAGT**	TFDP1	7027
**CTGACATTCCTGAGCCGAGT**	TFDP1P	391158
**CTGACTGTGAATTGCTTCCT**	PFDN1	5201
**CTGACTTACAATTATGGCGT**	GLOD4	51031
**CTGAGAATCTGAAAAGCATA**	PIK3R1	5295
**CTGAGACAGATCCCATTGGT**	CRKL	1399
**CTGAGATGCAGTAAAATCGC**	EXOSC9	5393
**CTGAGCCCGATGTACCCCAG**	ERF	2077
**CTGAGGAGATATGATAAACG**	ZNF429	353088
**CTGAGGTTTGCGTAGACCGG**	JUN	3725
**CTGATAATCCAGTCCAGCAA**	JUN	3725
**CTGATCGTAGGACCACGGTG**	RUNX1	861
**CTGATGAATAGTATGGTCCT**	RASSF9	9182
**CTGATGCGCCAGTTTCTAAG**	CDK4	1019
**CTGATGCTGTGAGTTCTGAT**	XIAP	331
**CTGCAAACTTTCCGATAACG**	ONE_NON-GENE_SITE_190	ONE_NON-GENE_SITE_190
**CTGCAAAGCCAAATAGCAGG**	BSCL2	26580
**CTGCAAAGCCAAATAGCAGG**	HNRNPUL2-BSCL2	100534595
**CTGCAAATGGGCAGTACAGG**	CCNA1	8900
**CTGCAACAAAGCCTGGACGC**	RALGAPA2	57186
**CTGCACACATTTGAGTCCCA**	MTOR	2475
**CTGCACAGAACTCAACGCTG**	COX4I2	84701
**CTGCACCATAGAAACGTGTG**	AKT3	10000
**CTGCAGGCAGCGTATGACAA**	AKT2	208
**CTGCAGGTAGAGGTACTGCA**	RTN4R	65078
**CTGCAGTCTGTTGCTTTAGG**	SNX14	57231
**CTGCATGCCGAATACCGACA**	TYMS	7298
**CTGCATGCCGAATACCGACA**	TYMSOS	494514
**CTGCATTCCAGGTCAGTCTG**	TUBB	203068
**CTGCCACCAGGAATGCCAGG**	FLJ42627	645644
**CTGCCACCAGGAATGCCAGG**	PDPK1	5170
**CTGCCACTGCAATACCAACG**	JAK2	3717
**CTGCCTGCCCTAACGTTGGA**	ONE_NON-GENE_SITE_191	ONE_NON-GENE_SITE_191
**CTGCGCACCCACCAAGCCCA**	MAGEA12	4111
**CTGCGGAAGGAGGAGTACCA**	ERF	2077
**CTGCGGGGGATACTCCCTCG**	MCM6	4175
**CTGCTCACCTCGACCCAGTG**	SPRED3	399473
**CTGCTGAGTCTGGCGCTGCT**	C4orf48	401115
**CTGCTGCTGCTGAAGCTGAG**	SPRR1B	6699
**CTGCTGTCCAATGTGGACGA**	ARHGEF2	9181
**CTGCTTGGCAGAACGGCGGA**	RABGAP1	23637
**CTGCTTGTCGGCCCAGAACG**	WDR26	80232

**CTGGAAAAACTGGATGGTGA**	MRAS	22808
**CTGGAAGGCCTATCATCAGT**	POLR2H	5437
**CTGGACACAGTCACCTTGGA**	PSMD3	5709
**CTGGAGAAATATAACGTCAC**	ONE_NON-GENE_SITE_192	ONE_NON-GENE_SITE_192
**CTGGAGATTTATCGAAAGCG**	ONE_NON-GENE_SITE_193	ONE_NON-GENE_SITE_193
**CTGGAGCAGGTATACATCCG**	SNRPD3	6634
**CTGGAGCTACAGGGCGTCAA**	GLOD4	51031
**CTGGAGTGACAGCAAACGGG**	TIAM1	7074
**CTGGATGCCCTCAAGGACGT**	E2F1	1869
**CTGGCAACTCCCACTAGCAA**	ONE_NON-GENE_SITE_194	ONE_NON-GENE_SITE_194
**CTGGCAGCGTATCTGCTACG**	NPRL3	8131
**CTGGCAGGGCTCGGGCACTT**	SPRR1B	6699
**CTGGCAGTGCTGTACCAGCT**	TRAPPC5	126003
**CTGGCCCACTCGCTCAGCAC**	SRC	6714
**CTGGCCGCCTGAACGACAGG**	RPTOR	57521
**CTGGCCTACAGCGAGCCGTG**	DDIT4	54541
**CTGGCCTCAGCACCTAAGAC**	EBP	10682
**CTGGCGCCCGGGAACACTCA**	LCK	3932
**CTGGCTATCAAAGGCACCCT**	ATL2	64225
**CTGGCTGTAGAGTTCTACCG**	PLCE1	51196
**CTGGGAGTGCCGTATGTCAG**	MCM4	4173
**CTGGGCACGTGGCAGGCACG**	QPRT	23475
**CTGGGCTTATGGGATACAGC**	RAC1	5879
**CTGGGCTTATGGGATACAGC**	RAC1P2	442775
**CTGGGGCATAACTTTGTGTC**	OSGIN1	29948
**CTGGGGCTATAAATCAACTA**	ONE_NON-GENE_SITE_195	ONE_NON-GENE_SITE_195
**CTGGGTGCCTGGATCAAGTG**	FLVCR1	28982
**CTGGTATAATTACCTGACTG**	GLOD4	51031
**CTGGTCATTTATAGAAACCG**	PDGFRA	5156
**CTGGTGCATGATGACGAGGT**	PRKCZ	5590
**CTGTAATAGAGAAAACACAG**	RALA	5898
**CTGTACCAAGGTCAAGCAGA**	ARHGEF2	9181
**CTGTACCATAAATTTGGACC**	RASA2	5922
**CTGTAGCACTTTCAGAAGCG**	ALK	238
**CTGTCATAGGCATCGACGAG**	TK1	7083
**CTGTCATGTAGACGTAGGCC**	ONE_NON-GENE_SITE_196	ONE_NON-GENE_SITE_196
**CTGTCTCCAGAGAGTCAACG**	HCK	3055
**CTGTGAACTCGGGGTCAAAG**	RPS6KA2	6196
**CTGTGCAAGCCATCCCATGG**	INSRR	3645
**CTGTGCAGATAAGTCAGCTG**	PAK1	5058
**CTGTGCCCAGATCTCACTAG**	RANGAP1	5905
**CTGTGGCCAAAAAGCTCATG**	PLCXD3	345557
**CTGTGGGATGAAGAGCCTCG**	PLXNB1	5364
**CTGTGTGCTTGAACCCTACG**	ONE_NON-GENE_SITE_197	ONE_NON-GENE_SITE_197
**CTGTTACTGAAATGTGCGTG**	LOC653406	653406
**CTGTTACTGAAATGTGCGTG**	NAIP	4671
**CTGTTAGCGTGTATCTACCT**	PANX1	24145

**CTGTTCAATTCGCTTAGCGT**	ONE_NON-GENE_SITE_198	ONE_NON-GENE_SITE_198
**CTGTTCTCTGTCACTGAGAA**	DNAJC9	23234
**CTGTTGACTACTTTGACGGA**	LPCAT3	10162
**CTGTTGAGTGTCAATAACTT**	PFDN1	5201
**CTGTTGCCTTCATAGATAAG**	UBE2N	7334
**CTGTTTGCGGATAGGATAGG**	RAC1	5879
**CTGTTTGCGGATAGGATAGG**	RAC1P2	442775
**CTTAAGTCGAGAGCCGACCG**	PDCD6IP	10015
**CTTAATAAGAAGGATTCACA**	SNX14	57231
**CTTAATGATGAACCACCAGA**	TMEM263	90488
**CTTACCCCAAGATAGTAGGG**	INTS6	26512
**CTTACCTACCATGCGAGTCA**	ONE_NON-GENE_SITE_199	ONE_NON-GENE_SITE_199
**CTTACGTACCATATGTACGC**	SOS1	6654
**CTTACTAATGAAGTAATCCG**	ETS1	2113
**CTTACTTGATACACCGTGAA**	AUP1	550
**CTTAGCCATAGAAGTTCAAG**	CAMK2D	817
**CTTAGCCCACTGATGAACGA**	CREBBP	1387
**CTTAGTCCAACTGATCACGG**	FGFR2	2263
**CTTATGGCTAGTAAGGGTTG**	ZNF429	353088
**CTTATGGTTAGTAAGGGCTG**	ZNF429	353088
**CTTCAACTTGTAGACACAGC**	RHEB	6009
**CTTCAAGAAGCGTGCACCTC**	RPL31	6160
**CTTCAGCTCGTTCTCCGTAG**	TRAPPC2L	51693
**CTTCAGTGTGGTCAAAACAA**	SREBF2	6721
**CTTCATCTCTTGGATCAAAG**	CBLB	868
**CTTCATTCACAAATTTCCGT**	ATP6V0A1	535
**CTTCCACTTCAGAATCACTG**	NFE2L2	4780
**CTTCCACTTGTGGACCGGTG**	PIK3R6	146850
**CTTCCCAAAGCGCTCTACCG**	AGPAT3	56894
**CTTCCCCCGATATACCTCAC**	SNRPD3	6634
**CTTCGAATGACAGTAAGACA**	AURKA	6790
**CTTCGACACTGATGATCTGC**	ATXN3L	92552
**CTTCGACACTGATGATCTGC**	GS1-600G8.3	100093698
**CTTCGGGGAGACAACGACGG**	MYC	4609
**CTTCGGGGCCAATCATGGCA**	CTNNBL1	56259
**CTTCGTAAGTTGGTAAAGCC**	ONE_NON-GENE_SITE_200	ONE_NON-GENE_SITE_200
**CTTCGTCCAGCTGTGCACGC**	AGPAT3	56894
**CTTCTCAGAATGACACGCTG**	RASSF6	166824
**CTTCTGAGCCATGGTGAAGA**	CASP3	836
**CTTCTTACTATCCCATAAAG**	PRKAG1	5571
**CTTGAATCTCTCTGTCCACG**	RAF1	5894
**CTTGACAGCATACTCCATGT**	RPS6KA1	6195
**CTTGACGGCCACTTTCATCG**	PDGFRB	5159
**CTTGAGGATCTCAGTTGTGA**	PHB	5245
**CTTGATCGAGTAGAAAATGA**	RASSF9	9182
**CTTGATGAGTGCCTCACCAT**	ONE_NON-GENE_SITE_201	ONE_NON-GENE_SITE_201
**CTTGATGTAATAAAAGGTTG**	CTNNB1	1499

**CTTGCCGTCCACCTCAATGT**	RHOB	388
**CTTGCCTCGAAACAAATCTA**	SMU1	55234
**CTTGCGCAGCTGCTGTGCCT**	CDC37	11140
**CTTGCGGTACTACTATGACA**	ELK1	2002
**CTTGCGTCCGTTGCACACAT**	PRKCE	5581
**CTTGCTCATAGTCTTCAGCA**	RHOA	387
**CTTGCTTTACTGCTGCCATG**	ABALON	103021294
**CTTGCTTTACTGCTGCCATG**	BCL2L1	598
**CTTGGCGAAGCGCACGACGC**	SPRED3	399473
**CTTGGGCTCCGTCAAGTCAG**	PRKCSH	5589
**CTTGGTCAGACCAGCGAATG**	RAPGEF1	2889
**CTTGGTGTTCTCGTCATACA**	JAK1	3716
**CTTGTATAACACGACTCCTT**	ONE_NON-GENE_SITE_202	ONE_NON-GENE_SITE_202
**CTTGTCCAGGACCACGATGG**	RASGRF1	5923
**CTTGTCGTGAAAGCGAACGA**	MFSD11	79157
**CTTGTCGTGAAAGCGAACGA**	SRSF2	6427
**CTTGTCTTGAGTAAGTGTTG**	ZNF429	353088
**CTTTACATGCACCCTACTAG**	ONE_NON-GENE_SITE_203	ONE_NON-GENE_SITE_203
**CTTTATTACAGCATTCAACC**	MON2	23041
**CTTTCAAACATGATGTCAGA**	PRKAA1	5562
**CTTTCCAAAAAGTGACACTG**	ZNF429	353088
**CTTTCTATAACTGTCAGCTT**	RALB	5899
**CTTTGATACCAAGAGGTACC**	PSMD11	5717
**CTTTGTACAGGAACACAGAT**	PAK3	5063
**GAAAAAGGACGTGATCGTCC**	PRKCG	5582
**GAAAAATAGCGTGCATCCTG**	TIAM1	7074
**GAAAACTTTCAGTTATCCGC**	TRAPPC4	51399
**GAAAAGGGCCAGGTAAACGG**	CECR2	27443
**GAAAAGTCATCCCACGAAGT**	ONE_NON-GENE_SITE_204	ONE_NON-GENE_SITE_204
**GAAAATCAGAGTGTGACAGG**	EXOC1	55763
**GAAAATCATTAAAGCTGATG**	FAM20B	9917
**GAAACACCTGAAGAAAGACA**	POLR3G	10622
**GAAACAGTGTCTCACGCATA**	FGFR1	2260
**GAAACCACAGCTGTGGAACG**	MAF1	84232
**GAAACCCATTTGCCAGCTCA**	BUB1	699
**GAAACCGATCGACCGCGAGA**	SAP18	10284
**GAAACGGTATAAGAACCGGC**	DPF1	8193
**GAAACTGGCTAAAATTAAGC**	RASSF9	9182
**GAAACTTAATAAGCAGCAGA**	HDAC1	3065
**GAAACTTGAAATATGAAGTC**	CASP3	836
**GAAAGAAGTCATTATTGCAA**	AKT3	10000
**GAAAGAAGTGGAGAGAGGAA**	PTPN11	5781
**GAAAGACTTGAACTCAAGGT**	RASGRP1	10125
**GAAAGGCGATCACCACCCAC**	EBP	10682
**GAAAGTCGTATGTTATTACG**	RABGAP1	23637
**GAAATACTTACTTGACAGAG**	GRB2	2885
**GAAATATCACCATGAAAAAC**	PLCXD3	345557

**GAAATCGGAAGAGCTTAATG**	ELK1	2002
**GAAATGATCAAACGCCGTGT**	ONE_NON-GENE_SITE_205	ONE_NON-GENE_SITE_205
**GAAATGCTGGGGAGTGTCGT**	MAGEA3	4102
**GAAATGCTGGGGAGTGTCGT**	MAGEA6	4105
**GAAATGGAGTAAAACAATGG**	GLOD4	51031
**GAACAATAGTCGAACTAGGT**	ONE_NON-GENE_SITE_206	ONE_NON-GENE_SITE_206
**GAACAATCACTGCCACATCG**	VPS39	23339
**GAACAATTTAATACAACTGG**	ECT2	1894
**GAACACAGTGCTAAACATCT**	ATP6V0A1	535
**GAACACGTAAGACTAGACTC**	ONE_NON-GENE_SITE_207	ONE_NON-GENE_SITE_207
**GAACACGTGCTCATCGATGT**	PFDN5	5204
**GAACAGCAAGCGCTACAAAG**	PSMD3	5709
**GAACAGCCTATGGATTAAGC**	PDGFRA	5156
**GAACATAAAAAGCATTCGTC**	BIRC5	332
**GAACATCTAGAACAGCTTGT**	PAK3	5063
**GAACATTCATATGGTTTGGA**	ATL2	64225
**GAACATTGGCTATGTCATAG**	E2F7	144455
**GAACATTTCTACCTGACCAG**	CERS2	29956
**GAACGAGAGACTACGCGCTC**	MED11	400569
**GAACGGATCTGCCTTTACAC**	KDM3B	51780
**GAACGGCGGGTTGACAACAT**	ITCH	83737
**GAACGGGGGTTTGTCAAGCA**	PRKCA	5578
**GAACTATATTGCGGACATTG**	RHOC	389
**GAACTCAAGCTCAACAACTG**	RANGAP1	5905
**GAACTGCAATGTTAGGAGCA**	METTL5	29081
**GAACTTGTGTCCAACCAACC**	ONE_NON-GENE_SITE_208	ONE_NON-GENE_SITE_208
**GAAGAAATGACTGAAATGCA**	ECT2	1894
**GAAGAAGGCCTTGAGGCCCG**	MAGEA2	4101
**GAAGAAGGCCTTGAGGCCCG**	MAGEA2B	266740
**GAAGAAGGCCTTGAGGCCCG**	MAGEA3	4102
**GAAGAAGGCCTTGAGGCCCG**	MAGEA6	4105
**GAAGAAGGTAGTGCTAGATG**	RALA	5898
**GAAGAATATTGATGGTTGGG**	SOS1	6654
**GAAGAATATTGATGTCCCCA**	MCM3	4172
**GAAGACATGAAGTTGCACTA**	GMDS	2762
**GAAGACATTGAACGGGAAAT**	MED11	400569
**GAAGACCTGCCCTTTAAAAA**	CRKL	1399
**GAAGACGTCGAACTCGAAGA**	CBLC	23624
**GAAGACGTGGCAATTTTCGT**	ONE_NON-GENE_SITE_209	ONE_NON-GENE_SITE_209
**GAAGACGTGGCAATTTTCGT**	INACTIVE_4T_4	INACTIVE_4T_4
**GAAGAGACATCTGGGATCAG**	ZFP90	146198
**GAAGAGCACAGAAGTGATGA**	CHMP3	51652
**GAAGAGCACAGAAGTGATGA**	RNF103-CHMP3	100526767
**GAAGAGCTCCGAAAACTCCT**	TCF7L2	6934
**GAAGAGGCTTCCATTAACGT**	EXOC2	55770
**GAAGAGGGACGGTACAAACG**	CASP7	840
**GAAGAGGTAAGACTCACCAT**	GRB10	2887

**GAAGAGGTTGCTGTTGATCT**	RASSF1	11186
**GAAGATACCCCCAGTCACAC**	TMEM263	90488
**GAAGATCACCCGGACCAAGG**	PIN1	5300
**GAAGATGCGAGGAAGCTGGC**	PHB	5245
**GAAGATTCGGAGCCTTGATG**	PRKAA1	5562
**GAAGCAAAAGAAAAACTATG**	PLCE1	51196
**GAAGCACATGAGAATGACGT**	RHOB	388
**GAAGCAGGGCCATATCTCGT**	FAM217B	63939
**GAAGCCCAAAGCCTTTGTTG**	TIAM2	26230
**GAAGCCCCGGAACAGCTGAT**	RPS6KA1	6195
**GAAGCTGTATAATGCTTGGG**	PIK3CA	5290
**GAAGCTTATTTAGTGATCTC**	RAD52	5893
**GAAGGACTGAGGTCTCATAG**	MTAP	4507
**GAAGGAGCGATACTCCAAGT**	RASGRP2	10235
**GAAGGCAGGCCCCCTGGACA**	BIRC7	79444
**GAAGGCTCAAACCCTAGGTT**	ONE_NON-GENE_SITE_210	ONE_NON-GENE_SITE_210
**GAAGGCTGGCTAAATAACAG**	TIAM1	7074
**GAAGGTCAGCGCCCAAGACA**	BLK	640
**GAAGGTGCGCGCGTCATCGT**	TRAPPC5	126003
**GAAGGTGCGTTCGATGACAG**	AKT1	207
**GAAGTAAACCCACTTAGCAA**	ONE_NON-GENE_SITE_211	ONE_NON-GENE_SITE_211
**GAAGTACCTAGAATACCTGG**	WDR26	80232
**GAAGTATGGAATACGTACAG**	SPRED1	161742
**GAAGTATTAAAATCCCAAGG**	ROCK1	6093
**GAAGTCAGCTGTAGCTGCAA**	RALA	5898
**GAAGTCGGGGTATAACAGTC**	ONE_NON-GENE_SITE_212	ONE_NON-GENE_SITE_212
**GAAGTGACCGATCCTGAGAA**	USP7	7874
**GAAGTGGATCCGCGTGGCAG**	RGL2	5863
**GAAGTGTACTCAAAAAGGAT**	POLR1A	25885
**GAATAAGTGAGTCACTGTGC**	PSMA6	5687
**GAATACCTATTATGTTGGTT**	RHEB	6009
**GAATCGGTGCCAAACATGAA**	SOS2	6655
**GAATCTTACCATCTCCAAGT**	PEX3	8504
**GAATCTTCAGACTCGAATGG**	INSR	3643
**GAATGAGAACATTTATGTAC**	BIRC2	329
**GAATGCTGCCCCTATCAGAA**	IRF2	3660
**GAATGGCATATCAGCAAACC**	SOS2	6655
**GAATGTTAGTAACCCGACCT**	CNOT2	4848
**GAATTCAATGATTCTGACTC**	NFE2L2	4780
**GAATTCATCATGAAGAACTG**	POLR2C	5432
**GAATTCGGCAGTTGCTGTAA**	RALB	5899
**GAATTCTAAGAGTGGCCCAG**	UBAP1	51271
**GAATTCTCAGCACCGTGCCA**	LGALS7	3963
**GAATTCTCAGCACCGTGCCA**	LGALS7B	653499
**GAATTTACAGCTCATGCCAC**	GPRC6A	222545
**GAATTTCTGAGAACTAGTTG**	PRKAA2	5563
**GACAACTATCTTGTTGCTTG**	WDR26	80232

**GACAAGAAACAATCTAAATC**	TRIM64	120146
**GACAAGAAACAATCTAAATC**	TRIM64B	642446
**GACAAGTGCCATAATCGAGG**	SPRY3	10251
**GACAATAATCCACACCAGAA**	PRKAA1	5562
**GACAATCCAGAATTTAACAT**	IFIT5	24138
**GACACAAGTGTGAACGTTCG**	ONE_NON-GENE_SITE_213	ONE_NON-GENE_SITE_213
**GACACACAGAACCTTCCGAG**	FANCA	2175
**GACACGGACCACCCATCCCG**	PAK4	10298
**GACAGAAGTTGTGATCACAT**	TBK1	29110
**GACAGACCTGATGATGTCAG**	DAB2IP	153090
**GACAGCCACTTTGTCTGATG**	CDC25A	993
**GACAGCCCTCCATACGGCAG**	GATA6	2627
**GACAGGCATAAAGAATGGCG**	PREB	10113
**GACAGTCATTGTAGAACCAC**	BRIP1	83990
**GACATCAAATATCACTAAGT**	PAK1	5058
**GACATCACCAACGTGCTGGA**	E2F2	1870
**GACATCCGGCTGTCAAGAAG**	SPRY4	81848
**GACATCCTGACTCTTGCATG**	WDR26	80232
**GACATCGAAATCCATTGAGC**	RAF1	5894
**GACATGGACGGATCGTAGGT**	ONE_NON-GENE_SITE_214	ONE_NON-GENE_SITE_214
**GACATGTTAGCCGCTAACAG**	DDX46	9879
**GACCAAAGAGTGCCGCAAGA**	PLEKHF1	79156
**GACCAAGGTGAGGAGTCGGA**	LOC390617	390617
**GACCAAGGTGAGGAGTCGGA**	TFDP1	7027
**GACCAAGGTGAGGAGTCGGA**	TFDP1P	391158
**GACCACCATTCAAAATTGAG**	BRIP1	83990
**GACCACGTTGAACTCCCGGG**	PCDH7	5099
**GACCATCAAGATTGTCATCG**	PTGS1	5742
**GACCATGAGATCTCTTGTAT**	LUZP4	51213
**GACCATGTGGACATTAGGTG**	RAF1	5894
**GACCCACTGGTTACCTACGA**	LCK	3932
**GACCCATGATCCTAACAAAG**	PLAGL2	5326
**GACCCCAGTTTACCCCATCC**	ABALON	103021294
**GACCCCAGTTTACCCCATCC**	BCL2L1	598
**GACCCCATGGACTACAAGTG**	AKT2	208
**GACCCTGACAAGGACGTGCA**	PPP1CA	5499
**GACCGACTTGCGAATCACCA**	GRB10	2887
**GACCGGATGACGGAGATCCG**	NLRC3	197358
**GACCGGCCAGAGGCGACACA**	ERF	2077
**GACCTACCGTGCAGGACCAG**	RALGAPA1	253959
**GACCTACCTTAAGCCGAAAG**	ONE_NON-GENE_SITE_215	ONE_NON-GENE_SITE_215
**GACCTCAAGTGATCCGGACA**	ONE_NON-GENE_SITE_216	ONE_NON-GENE_SITE_216
**GACCTCAGAGGACATATGGT**	CNKSR2	22866
**GACCTCATAGACATTTGGGA**	MCM5	4174
**GACCTGATCAAATGGGATGG**	PRKCSH	5589
**GACCTGGAACGGTACCACCA**	SRC	6714
**GACCTGGACTACCTGTCCGA**	OSGIN1	29948

**GACCTTGGCAATCAGTGTGA**	RASAL3	64926
**GACGACTCCGAGGAACATTG**	YAE1D1	57002
**GACGCACCCCAACATCGACA**	CDC37	11140
**GACGCGGACCTACGACCGCG**	NUDT4	11163
**GACGCGGACCTACGACCGCG**	NUDT4B	440672
**GACGCGGACCTACGACCGCG**	NUDT4P2	170688
**GACGCTACAACATCAAACTG**	PPP1CA	5499
**GACGGCTGGACAGCACGCGT**	SLC25A1	6576
**GACGTATTAAGATATCTCGA**	SPRED1	161742
**GACGTGGAGATGACGTCTTG**	INSRR	3645
**GACTAACGTGACGTACTGGG**	PDGFRB	5159
**GACTACAGCACGACCCCCGG**	EIF4EBP1	1978
**GACTACCGGCCTAAAAGAGC**	ONE_NON-GENE_SITE_217	ONE_NON-GENE_SITE_217
**GACTATGAAGAATTCAGCAA**	CASP8	841
**GACTCCCAGGAAATGCCCTG**	PRKCQ	5588
**GACTCTCTACCGAGAAACCA**	VPS18	57617
**GACTGACATGTCTCGACCTG**	CCT4	10575
**GACTGAGAATCATCGATTGA**	ONE_NON-GENE_SITE_218	ONE_NON-GENE_SITE_218
**GACTGCCAGAGATCGAAAGA**	XBP1	7494
**GACTGGAAAGTCTATTGTGA**	BARD1	580
**GACTGGAACGAGAATACGGG**	DUSP6	1848
**GACTGGGAATAGTTACTCCC**	PTEN	5728
**GACTTACGAGTGGAACATGG**	TRAPPC4	51399
**GACTTGTATAAAGATCGACT**	SPC25	57405
**GAGAAACAGATCAAGCACTT**	CDC37	11140
**GAGAAAGCAGTCTTTACCCA**	ETS1	2113
**GAGAACATTACCGGATTCGA**	MDM2	4193
**GAGAACATTCTGGTGACAAG**	CDK4	1019
**GAGAACCTAGAAATCATACG**	EGFR	1956
**GAGAACGTCAGGATGCAGAT**	ROCK2	9475
**GAGAAGAATCGACCTTTAAA**	DHFR	1719
**GAGAAGAATCGACCTTTAAA**	DHFRP1	573971
**GAGAAGGGGCAACCTACCCA**	TGIF1	7050
**GAGAATACGTCCTCCACATG**	PIK3CG	5294
**GAGAATGTGACAATCACTGC**	UBAP2L	9898
**GAGACCAGAACGATCTCAAG**	CDC25A	993
**GAGACCCCCAGTGCCATCAA**	ABALON	103021294
**GAGACCCCCAGTGCCATCAA**	BCL2L1	598
**GAGACCCTCTCCAATACAGC**	DAB2IP	153090
**GAGACCTACGGAGCCCTCCA**	RASGRP4	115727
**GAGACGAAGCATGCCAATGC**	ONE_NON-GENE_SITE_219	ONE_NON-GENE_SITE_219
**GAGACGACCACCCGAACTCA**	ONE_NON-GENE_SITE_220	ONE_NON-GENE_SITE_220
**GAGAGAAGAAACCAATTGGT**	BRAF	673
**GAGAGACACATCTCACATGA**	ACVR1B	91
**GAGAGCACGATGAATACGTA**	MET	4233
**GAGAGCCCAGAAACGGCCGA**	RALGDS	5900
**GAGAGCGCACGTGTGTGCGT**	HDAC9	9734

**GAGAGGCCCAGAAAATTGAC**	CYTH2	9266
**GAGAGGGATTACTATAGGCA**	SLC11A2	4891
**GAGAGGGTTAAGTGGATCAG**	PIK3CG	5294
**GAGAGGTGGCCCATTCTCGG**	CBLC	23624
**GAGAGTAAGGGGCTCGACCC**	ONE_NON-GENE_SITE_221	ONE_NON-GENE_SITE_221
**GAGAGTCTGTAAACGCCGTG**	WDR26	80232
**GAGAGTGATACCTTTGGCGG**	CCT4	10575
**GAGATATCTCTAGGCGATGG**	CCDC6	8030
**GAGATGCAGGGACACACGAT**	VHL	7428
**GAGATGCCCACATCATTGGC**	MAF1	84232
**GAGCAACTACCTCTTTCTGG**	PPP1CA	5499
**GAGCACCACGAAGCGCCGCG**	FLVCR1	28982
**GAGCACGACTTGATCTTCCG**	FAU	2197
**GAGCACGACTTGATCTTCCG**	FAUP1	140623
**GAGCACTCACTCGATCTGGT**	VAV1	7409
**GAGCACTGGCCCCACACCAG**	ANAPC11	51529
**GAGCAGCCGATAAAGAAGAG**	SLCO2B1	11309
**GAGCAGCTACTGTATACTCC**	ICMT	23463
**GAGCAGGTTGCGGTCCCGCA**	CCND2	894
**GAGCAGTATACGAGGATGGT**	SLC22A25	387601
**GAGCCACGGGAACACATGGT**	FANCA	2175
**GAGCCCAGGCAAGATGAGCG**	RUNX1	861
**GAGCCCATAGAGGTCCACTC**	ONE_NON-GENE_SITE_222	ONE_NON-GENE_SITE_222
**GAGCCTCACCTAACACCCCA**	PIK3R5	23533
**GAGCCTCATGCTGACCGGAG**	CXCL14	9547
**GAGCCTCCAGATATAGTAAG**	ITCH	83737
**GAGCCTCTGGCCCAGAATGG**	ZFPM1	161882
**GAGCGACAAGAAGCTCACCC**	CIDECP	152302
**GAGCGACAAGAAGCTCACCC**	EMC3	55831
**GAGCGACAGCCCGAAGACCA**	C12orf49	79794
**GAGCGCTGCTCAGATAGCGA**	TP53	7157
**GAGCGTGGTGAAGCTGACGG**	TK1	7083
**GAGCGTGTGTCGGGTCCGAG**	SPRED3	399473
**GAGCTCAACTCCAACCACGA**	SET	6418
**GAGCTGCAATTGGAGATCAG**	LMTK3	114783
**GAGCTGGAGAAGACGTTGAG**	MAEA	10296
**GAGCTTCCCGGACGACGACG**	SPRED2	200734
**GAGGAAGCAGATATCCGGTG**	NF1	4763
**GAGGAAGGGCTCATGCCTCA**	RGL3	57139
**GAGGAATTGCAATACGCATG**	RAB3GAP2	25782
**GAGGACAGACTACTAAAGCC**	TTK	7272
**GAGGACCTGAGCGAAAGTGA**	RASSF9	9182
**GAGGACTTGGGAAATTCAAG**	BPTF	2186
**GAGGATCAAGTGACTACTGC**	ONE_NON-GENE_SITE_223	ONE_NON-GENE_SITE_223
**GAGGATGAGACAGTTACGCC**	ONE_NON-GENE_SITE_224	ONE_NON-GENE_SITE_224
**GAGGATGCAGGCGAAGCAGG**	CASP7	840
**GAGGATTTCCCAGCTGCAAG**	B3GAT3	26229

**GAGGATTTCCCAGCTGCAAG**	B3GAT3P1	402146
**GAGGCACCTCTTTGGTACCC**	SHC4	399694
**GAGGCAGCGAAGGCCAGATA**	DUSP5	1847
**GAGGCCCAACTGCTGAAGCC**	SLC50A1	55974
**GAGGCCTGTAATTAACGCAA**	ONE_NON-GENE_SITE_225	ONE_NON-GENE_SITE_225
**GAGGCGGGCAGTGTGTATGC**	FGFR3	2261
**GAGGCGGGGCAATGTCAGGA**	PIK3R1	5295
**GAGGCTCAACGACAACCCCT**	RTN4R	65078
**GAGGCTCTCTACTCGGTCGT**	EXT2	2132
**GAGGCTGTTGGATTTAGCAA**	POLR3G	10622
**GAGGCTGTTGGATTTAGCAA**	POLR3GP1	100422395
**GAGGCTGTTGGATTTAGCAA**	POLR3GP2	100422391
**GAGGGGGCGTTTGTCCCGGG**	PAK4	10298
**GAGGGTCGGCTCTACCAAGT**	PSMA6	5687
**GAGGTAAACACCGTCTATGC**	ONE_NON-GENE_SITE_226	ONE_NON-GENE_SITE_226
**GAGGTAGATCTAGACTCACG**	FGFR4	2264
**GAGGTCCCCGAAAGCCGCAA**	ITFG1	81533
**GAGGTCTGGAGTGATGATGT**	CHURC1-FNTB	100529261
**GAGGTCTGGAGTGATGATGT**	FNTB	2342
**GAGGTGCTGGCCGACCACCC**	RUNX1	861
**GAGGTGGTCCCTGCACAAGA**	PIK3R6	146850
**GAGTACCCGACCCTCAAGGT**	MAEA	10296
**GAGTACGTCAGTGACAATCT**	ONE_NON-GENE_SITE_227	ONE_NON-GENE_SITE_227
**GAGTACGTGTACTTAGCCGG**	ONE_NON-GENE_SITE_228	ONE_NON-GENE_SITE_228
**GAGTAGCAGCGGTGCTGGGG**	MZT1	440145
**GAGTAGGAGCTTTCAATGTG**	RAB3GAP2	25782
**GAGTAGGCCTAACATGTCCA**	PLAGL2	5326
**GAGTATAGTTGATGGTAGTG**	MAGEA12	4111
**GAGTCCACGAAGAAGCAGGA**	SLCO2B1	11309
**GAGTCCATTCCTCTTCGAGG**	CHSY1	22856
**GAGTCCCTTGAGTCCCCTCG**	ARHGEF2	9181
**GAGTCGTCCACACCCCGTAG**	CHSY1	22856
**GAGTCTAGCATTAACTATGG**	ECT2	1894
**GAGTGCTCATCAAAACGCTG**	TIAM2	26230
**GAGTGGACGATCAGTCTAAG**	ONE_NON-GENE_SITE_229	ONE_NON-GENE_SITE_229
**GAGTGGCTTCAAATGTGTGC**	KPNB1	3837
**GAGTGGTTCAGGGAGGCACG**	RUNX1	861
**GAGTGTCTCATGGACGATAC**	ONE_NON-GENE_SITE_230	ONE_NON-GENE_SITE_230
**GAGTTAAAAAATTGGCCCCA**	DPM1	8813
**GAGTTAACAAAGAAATGGCA**	SLBP	7884
**GAGTTCACCGAGCGCAGCAC**	VHL	7428
**GAGTTGCCGAGATCTCTGCG**	KSR2	283455
**GAGTTTGGGAAGGTAGACCC**	STAMBP	10617
**GATAAAAATTTGCGACATTC**	VTA1	51534
**GATAAAGAAGAGATCGAATC**	MCM6	4175
**GATAACAAGTGTAGTCCCTG**	ITFG1	81533
**GATACAAACTCTTTGTCCCC**	PRKAB2	5565

**GATACCTTTATGGTTTCAGT**	CDK6	1021
**GATAGTACGGTGAACATCAT**	IRF2	3660
**GATATACTGGGCATAATCTG**	SMAD5	4090
**GATATCCTGGTAAGTCACAT**	E2F2	1870
**GATATCGATCCACTTCGATC**	ONE_NON-GENE_SITE_231	ONE_NON-GENE_SITE_231
**GATATCTTCGAGATTTCTTG**	SPTLC2	9517
**GATATTGCCATTAACTGGGC**	HDAC3	8841
**GATATTGTCAAAAGCCACGA**	ALK	238
**GATATTTATCAATCTCACAG**	CDC23	8697
**GATCAAAGCTAAAAGACATG**	STK3	6788
**GATCAACAGCCAGCGCATCG**	PLEKHF1	79156
**GATCAAGAAGAGATCGGACC**	PSMD11	5717
**GATCACACTTATATACCTGA**	PSMA6	5687
**GATCACCATGGCTCAACTGG**	PDGFRA	5156
**GATCACCCACAGCAGCAGCC**	TMEM263	90488
**GATCATGCGATACTTTGTCA**	SNX13	23161
**GATCCACTCACAGTTTCCAT**	TP53	7157
**GATCCAGCGGTCTCTCTCAG**	RASAL3	64926
**GATCCCGAAGTATTGACCGG**	PRPF38B	55119
**GATCCCGAATGGCATAACCA**	GPRC6A	222545
**GATCCCTGCAAGATGGACCT**	RASAL1	8437
**GATCCGAGCCGAGGTAATGG**	SLC30A1	7779
**GATCCTCATAATTCCCGACG**	SCD	6319
**GATCCTTCTTATTCCCAACC**	RHOA	387
**GATCCTTGGCTACAAGAGCT**	STAM	8027
**GATCGATACATATGGCTGGG**	CECR2	27443
**GATCGTTATGGAGTACTGTG**	STK4	6789
**GATCTCAATGATCGCTCCAG**	BSCL2	26580
**GATCTCAATGATCGCTCCAG**	HNRNPUL2-BSCL2	100534595
**GATCTGGAGGGAACACCAAG**	RALGDS	5900
**GATCTGGATGTACGCGCCCG**	E2F3	1871
**GATCTGGCAGAACTTCGACT**	RPTOR	57521
**GATCTTCAGATGGCCAACGG**	DAB2IP	153090
**GATCTTCCTGCGCACGTGTG**	RPL26L1	51121
**GATCTTGCTGACAAGAGGTA**	RAB6A	5870
**GATGAACCTTTACCAAAACG**	YAP1	10413
**GATGAAGCGACATGAAATGG**	ZNF429	353088
**GATGAATTCTATGCTAATGT**	VPS45	11311
**GATGAATTGCAGTTGTTCCG**	VCP	7415
**GATGACAACTTAATTGAAGG**	EXOC4	60412
**GATGACCTCACCTTCACCAA**	FGR	2268
**GATGAGACTAACATGTATGA**	PFDN1	5201
**GATGAGGCACTTTCTAAGGT**	MON2	23041
**GATGAGGGATCATGAACGAA**	GINS2	51659
**GATGAGGTGCTTCCCTTGAA**	C12orf49	79794
**GATGATAAGATAGACACCTG**	EXOC7	23265
**GATGATCACAGGAACTGACT**	MCCC1	56922

**GATGATGGCAAACAACCCAG**	TTK	7272
**GATGATGTCAGAACAGAAAG**	RAB6A	5870
**GATGATGTCAGAACAGAAAG**	RAB6C	84084
**GATGATGTCAGAACAGAAAG**	RAB6C-AS1	100131320
**GATGCAACATTTGTCAGTGA**	UBTFL1	642623
**GATGCAGCCGTATCTAGAGG**	RALGDS	5900
**GATGCAGGACGTGATTTCAA**	PREX2	80243
**GATGCCACATCTGCGTGACG**	INSRR	3645
**GATGCCATTCTCTAGCAGGT**	RIMS4	140730
**GATGCCCGAGCCTTTGACAG**	SPRED2	200734
**GATGCCTATATTTCTCGATG**	TRIM64	120146
**GATGCCTATATTTCTCGATG**	TRIM64B	642446
**GATGCGCACCAGTGGTGTGT**	FGFR3	2261
**GATGCTCTCATAACTATAGC**	ONE_NON-GENE_SITE_232	ONE_NON-GENE_SITE_232
**GATGCTGGAACCCTAAACCA**	PAK1	5058
**GATGCTGGAAGAGCTACACT**	ARHGEF2	9181
**GATGCTTCTAGCAAGATCCA**	PIK3R2	5296
**GATGGCCCGTGCACAGACTG**	NLRC3	197358
**GATGGGGACAGCATCACTCA**	VPS16	64601
**GATGGTGAAGGATAACCATG**	QPRT	23475
**GATGGTTGGCGACGTGACCG**	IFITM1	8519
**GATGGTTGGCGACGTGACCG**	IFITM2	10581
**GATGGTTGGCGACGTGACCG**	IFITM3	10410
**GATGTACCTATGGTCCTAGT**	KRAS	3845
**GATGTATCAACCTAGTGCTG**	HDAC2	3066
**GATGTCCGTCAGAACCCATG**	CDKN1A	1026
**GATGTCGGTGAAGCTAAAGG**	UBAP2L	9898
**GATGTTAATCTAACTAGTGA**	TM9SF3	56889
**GATGTTCCTGACGGCCAAAG**	MAEA	10296
**GATGTTCTAAGATTGAGTCA**	MCM4	4173
**GATGTTTGGTTACAATGGCT**	RAD52	5893
**GATTAAGCGAGTAAAAGACT**	NRAS	4893
**GATTAAGTGCTGGAACGGCG**	ANAPC11	51529
**GATTACCTTACGGAAAAGTG**	ROCK2	9475
**GATTACTATGACCTGTATGG**	PTPN11	5781
**GATTATAGCCTTAGTGAAGA**	MDM2	4193
**GATTATTGAAGCAAGACTAA**	PSMB1	5689
**GATTATTTCCTATTTAGAGA**	HSPE1	3336
**GATTATTTCCTATTTAGAGA**	HSPE1P2	326300
**GATTATTTCCTATTTAGAGA**	HSPE1P3	100507046
**GATTATTTCCTATTTAGAGA**	HSPE1P4	100287369
**GATTATTTCCTATTTAGAGA**	HSPE1P7	100132346
**GATTATTTCCTATTTAGAGA**	HSPE1P8	100292290
**GATTCAATTCCCATCGCAAT**	ONE_NON-GENE_SITE_233	ONE_NON-GENE_SITE_233
**GATTCTCCACCAGAGAAGAA**	TYMS	7298
**GATTGTAAAAATTTACAGAG**	LONRF1	91694
**GATTTCGTATGATCCGGTCG**	PAK2	5062

**GATTTGATATTGCTTACTGC**	UBAP1	51271
**GCAAAACAGAGTTACCCAAG**	NFKB1	4790
**GCAAAACGCAATACTGTAAT**	STK3	6788
**GCAAAAGAAATTCGTCACGT**	RAPGEF2	9693
**GCAAACAGTCGACCCCCATG**	DUSP1	1843
**GCAAACGTTCGCTTCAAGGG**	ONE_NON-GENE_SITE_234	ONE_NON-GENE_SITE_234
**GCAAAGTCTGTGGCAATGTG**	ROCK1	6093
**GCAACAGCAAAGGACCACTG**	HDAC11	79885
**GCAACCGCACCCGCATCACA**	FGFR1	2260
**GCAACGTCTACCAGAACTAC**	EXOC8	149371
**GCAACTAAAGTCTGTCACCT**	EXOC5	10640
**GCAACTTTGATGCAGCACGC**	NF1	4763
**GCAAGAACTCACCCATTGCC**	ANKHD1-EIF4EBP3	404734
**GCAAGAACTCACCCATTGCC**	EIF4EBP3	8637
**GCAAGACACCTGTCTTCACG**	FANCC	2176
**GCAAGATTGGACACTTAATC**	ONE_NON-GENE_SITE_235	ONE_NON-GENE_SITE_235
**GCAAGCAAGACATCAGCGAG**	RASAL2	9462
**GCAAGCTATTGACGCCGGAG**	DNAJC9	23234
**GCAAGCTATTGACGCCGGAG**	LOC100421490	100421490
**GCAATAGCAGCGGCAATACG**	UBAP2L	9898
**GCAATCAAAACACTAAAACC**	YES1	7525
**GCAATCAAAACACTAAAACC**	YES1P1	7526
**GCAATGCATGTGGAAGCAGT**	ANAPC11	51529
**GCAATTCCAAGTATTCGCCC**	ONE_NON-GENE_SITE_236	ONE_NON-GENE_SITE_236
**GCAATTTGGGTAGAATTTCG**	PIK3CA	5290
**GCACAAACAGTCCAGCATGA**	VPS25	84313
**GCACAAGCGCCGCAAAACCG**	RPS8	6202
**GCACAAGGAGGAACATGAGG**	IFITM1	8519
**GCACAATCTCCCTAACCCCG**	SLC25A1	6576
**GCACAATTTGAACTGTCACA**	TTK	7272
**GCACATAAGGAAATTAGTGG**	RASGRP3	25780
**GCACATAGTGCTCCTAACAG**	GPRC6A	222545
**GCACATCCCAGTCCACGACG**	HDAC10	83933
**GCACCAGCTCACGTTGACGT**	NSD2	7468
**GCACCAGCTTCGATCCCTCG**	QPRT	23475
**GCACCATAAATGCCGGGAGA**	PRKCD	5580
**GCACCCGCTCACCCTCAAGT**	PRKCZ	5590
**GCACCGCGTGCAACCCCACA**	RASSF1	11186
**GCACCGGGCAACGTTACGAA**	HDAC1	3065
**GCACCTCGCCCGACACGCTG**	MFSD11	79157
**GCACCTCGCCCGACACGCTG**	SRSF2	6427
**GCACGAATCAGGGGTTAGGG**	SHC4	399694
**GCACGATGCTGCCATACACC**	PLEKHF1	79156
**GCACGCGCGCGCCCACCTGG**	TRAPPC5	126003
**GCACGGGCGAAGGCACTACA**	IRS2	8660
**GCACGTTCAGACAGTAGTAG**	HDAC3	8841
**GCACTCACCGTCCACACGCG**	CXCL14	9547

**GCACTCTGGCTGTAACCTGG**	RASSF2	9770
**GCACTGAAACTGATCGGGCA**	PANX1	24145
**GCACTGACCGGAATGACCCG**	POLR2C	5432
**GCACTGAGACAAGACATCAT**	CNKSR2	22866
**GCACTGCCTAATACCTCTCG**	UAP1	6675
**GCACTTCATGGACCTCGCGG**	TUBGCP2	10844
**GCACTTCCACGGGTAAACCA**	GINS2	51659
**GCAGAACCTGAGTTTGACAG**	RPTOR	57521
**GCAGAACTAGGTAGAAACAG**	BRCA1	672
**GCAGACAATGAAGATCGTGC**	VTA1	51534
**GCAGACACTTTATAAAACCC**	ROCK1	6093
**GCAGACCATCTCTATCGCCA**	MCM5	4174
**GCAGACCCCAAAGGTCCTTG**	ARID5B	84159
**GCAGAGAGCTAATCAGCCGA**	ONE_NON-GENE_SITE_237	ONE_NON-GENE_SITE_237
**GCAGAGGGCACATACACTAG**	E2F7	144455
**GCAGATCGAATACGTGTTCG**	PCDH7	5099
**GCAGATCTAGGATGTGGTTG**	METTL5	29081
**GCAGATGCCATAGGTGAGGG**	RASSF7	8045
**GCAGATTATTGACAGAGATG**	CTNNBL1	56259
**GCAGCAAACTCTTCAGGACA**	INIP	58493
**GCAGCAATGAATACACAGAA**	SPRY1	10252
**GCAGCACCACGTCAGGCATG**	TRIM64B	642446
**GCAGCACGGTGGCGATACGA**	NPRL3	8131
**GCAGCAGCGAAAGCGCCTTG**	MAP2K1	5604
**GCAGCATTTGAAAACCCCAA**	BRCA1	672
**GCAGCCAACAGTATCTATCC**	ONE_NON-GENE_SITE_238	ONE_NON-GENE_SITE_238
**GCAGCCAACGCATGTCTGTG**	RCE1	9986
**GCAGCCATGAGTCTCCAAGG**	CBL	867
**GCAGCCGGTACCCAGACCAG**	NUDT4	11163
**GCAGCCGGTACCCAGACCAG**	NUDT4B	440672
**GCAGCCGGTACCCAGACCAG**	NUDT4P2	170688
**GCAGCCGTGTGACTTACAGA**	INSR	3643
**GCAGCGAGGAGTCGTCCTCG**	LMTK3	114783
**GCAGCGATGAGACTTCATGA**	PRKAG1	5571
**GCAGCGCTGAGCATTCCGGT**	ONE_NON-GENE_SITE_239	ONE_NON-GENE_SITE_239
**GCAGCGTTTATGGACGACAC**	PRKCH	5583
**GCAGCTCAGAAAGAATCCGT**	PRKAG3	53632
**GCAGCTCCACGAGCACATCA**	HDAC4	9759
**GCAGCTGTATTGTGCGATGG**	CHSY1	22856
**GCAGCTTCTGCACACATGAC**	TK1	7083
**GCAGGAAGGATCCCAAATAC**	PEBP1	5037
**GCAGGAAGGCAACGTCCGGG**	SPRY4	81848
**GCAGGACACTTACTTGGTTG**	RASSF10	644943
**GCAGGACAGACGACCCGCGT**	ONE_NON-GENE_SITE_240	ONE_NON-GENE_SITE_240
**GCAGGACAGCTACTGTCTGG**	RASAL1	8437
**GCAGGAGACACAGTACTCAT**	C12orf49	79794
**GCAGGATCTGATCCTCGCAG**	CECR2	27443

**GCAGGCGCTACATAATTGCT**	YES1	7525
**GCAGGGAAGGTACTAGGATC**	FLT3	2322
**GCAGGGAGTTGAGCTGTAGG**	MYB	4602
**GCAGGGGAAGACGTCTTCTG**	ZFPM1	161882
**GCAGTAAATCTACTTGACAC**	RIC1	57589
**GCAGTAAGAGCCAATCGTCG**	IRS2	8660
**GCAGTGAAAACATTAAAACC**	FRK	2444
**GCAGTGTGTCTGGTCTTATG**	CHSY1	22856
**GCAGTTGCAGTACATCGGCG**	MAPK3	5595
**GCATACCCGGCCGCATTGGG**	ONE_NON-GENE_SITE_241	ONE_NON-GENE_SITE_241
**GCATACTTACAACAACCAAG**	PSMA6	5687
**GCATAGCAGCGACCAGCCCG**	MAEA	10296
**GCATATATTCTAGAACCACA**	EXOC1	55763
**GCATATTGATAGCCATGCCG**	SHC4	399694
**GCATATTGCAGCCCAACAGA**	INIP	58493
**GCATCACTCCTCCAACATGA**	FERMT1	55612
**GCATCAGCGTGTCATACACG**	HDAC4	9759
**GCATGAAACTTACCTCACAA**	SLC22A25	387601
**GCATGAGGCAAGACCTGGCA**	NO_SITE_3	NO_SITE_3
**GCATGATCTTCACTTATACG**	ONE_NON-GENE_SITE_242	ONE_NON-GENE_SITE_242
**GCATGATTCACGCTAACCAA**	ONE_NON-GENE_SITE_243	ONE_NON-GENE_SITE_243
**GCATGGAGAAAGCTCCACTG**	RAB3GAP2	25782
**GCATGGTTGACCGTTCTGGA**	FGFR1	2260
**GCATGTGCTGTCACGCACAA**	RASSF1	11186
**GCATTAAGGACCTTCCTGCG**	HCFC1	3054
**GCATTGGAGCCCAACACAAG**	EXOC2	55770
**GCATTTGACACCGCTACTGG**	ONE_NON-GENE_SITE_244	ONE_NON-GENE_SITE_244
**GCCAAAAGTAGCCATAACCC**	SLC50A1	55974
**GCCAAAGCATCCTTATAGGT**	RASGRP1	10125
**GCCAAAGTACCCGCACTGCG**	CXCL14	9547
**GCCAAATAGCGCCTTCACGA**	CDC25A	993
**GCCAACTTCTACAAGGACTC**	DUSP3	1845
**GCCAAGCTCCACCGAAAGCG**	RSPH3	83861
**GCCAAGTATCGATACTCATG**	EXOC5	10640
**GCCACACCAGTGGATCGCAA**	SEC23B	10483
**GCCACAGAATGTCTTCATGG**	AKT3	10000
**GCCACTCACCTAAACTATCA**	RHOA	387
**GCCACTGAAAAGTTGTTGAG**	AKT3	10000
**GCCAGAGGCTGCTCCCCCCG**	TP53	7157
**GCCAGGTACCTCTACTACAC**	PSMD3	5709
**GCCAGGTAGGAACCATCCTG**	GMDS	2762
**GCCAGTGGAATATAATTGGG**	CNKSR2	22866
**GCCAGTGGCTCCATTCACCG**	ABALON	103021294
**GCCAGTGGCTCCATTCACCG**	BCL2L1	598
**GCCATAGTTGACCAGAGTCT**	SPRY4	81848
**GCCATGCATGCTGCCAGCAG**	LOC152845	152845
**GCCATGCATGCTGCCAGCAG**	PLAGL2	5326

**GCCATTCCCTCTCACCCAAG**	INPP4B	8821
**GCCATTGCAGATGCCATCCG**	RANGAP1	5905
**GCCCAAAGTTCAGATCTCAG**	RASSF2	9770
**GCCCAAATCTTGATTCACAG**	BSCL2	26580
**GCCCAAATCTTGATTCACAG**	HNRNPUL2-BSCL2	100534595
**GCCCACGGTGCATTTCGCAG**	SCRIB	23513
**GCCCAGTGGACTGACGTCAT**	ONE_NON-GENE_SITE_245	ONE_NON-GENE_SITE_245
**GCCCATCTGCCAGGTCACAA**	RCE1	9986
**GCCCCATAGCCAATCATTCG**	SLCO2B1	11309
**GCCCCCCCCGACGCTCCAAG**	IRS1	3667
**GCCCCGACCAACCTTCCAGC**	PPP1CA	5499
**GCCCGACAAATGCAAATGCG**	MCM5	4174
**GCCCGAGCCCTGCCAGCCCA**	SPRR1B	6699
**GCCCGAGTCTACAGATTCGT**	ONE_NON-GENE_SITE_246	ONE_NON-GENE_SITE_246
**GCCCGCGACTTGAAGAACGG**	CDK6	1021
**GCCCGCGCCCCATTCTGCAA**	ONE_NON-GENE_SITE_247	ONE_NON-GENE_SITE_247
**GCCCGTGCTCACGCTCACGG**	PCDH7	5099
**GCCCGTGCTGGAAAAGTGAG**	FAU	2197
**GCCCGTGCTGGAAAAGTGAG**	FAUP1	140623
**GCCCTCCATGTACTGGCGAT**	CDC37	11140
**GCCCTGAAGGAGAAGGAGAA**	COX4I2	84701
**GCCCTTACCAGTTCTTTGGG**	CHMP3	51652
**GCCCTTACCTTGACTGGACG**	FUNDC2	65991
**GCCGAACAAGCAAAGAACAG**	RAF1	5894
**GCCGACAGCGAGCTGTACTG**	GATA6	2627
**GCCGAGCACCACCCGGCGAG**	EIF4EBP1	1978
**GCCGAGGAGGCAGAGCACCG**	SLBP	7884
**GCCGCCCGGTGTACCTGGGT**	PEBP1	5037
**GCCGGGAAAAGTGATTCGAG**	LCK	3932
**GCCGGGTGCGCTGTTACACA**	HDAC5	10014
**GCCTAACAACCGGCACATCA**	EXT2	2132
**GCCTACAGACCAAATATCAA**	MAPK1	5594
**GCCTACCATGTACCCACCCT**	PRKCQ	5588
**GCCTAGTAGTTCGTGGACCA**	VPS45	11311
**GCCTCAGTACATCAAAGCCA**	SMC1A	8243
**GCCTCCCCACTGAGGAACTG**	RPS6KA2	6196
**GCCTCGTGGGATGCGTTGTG**	ONE_NON-GENE_SITE_248	ONE_NON-GENE_SITE_248
**GCCTGAAAAAGCAACTGAAC**	SNX14	57231
**GCCTGACTTGGTGGATACCG**	FLVCR1	28982
**GCCTGCGTTCACCATTCATG**	CCNA2	890
**GCCTGGACATAATTTCGTTG**	RAPGEF1	2889
**GCCTGGACTACATTCCGCAA**	CASP8	841
**GCCTGGTGTGGGTGTCACAG**	RASSF7	8045
**GCCTGTGGGACACTATGAAG**	RPS6KB2	6199
**GCCTGTTGGGCCGATCAAGG**	ONE_NON-GENE_SITE_249	ONE_NON-GENE_SITE_249
**GCCTTACGTAGCAGACAGAC**	UGCG	7357
**GCCTTAGGACTGCACATGTG**	POLR2C	5432

**GCCTTCAAAGGTCAACTACC**	MTAP	4507
**GCCTTCGATCAAGTGCACTG**	BPTF	2186
**GCCTTCTAGTCTCACACGAA**	ONE_NON-GENE_SITE_250	ONE_NON-GENE_SITE_250
**GCCTTGGACGTTGAACTACG**	ONE_NON-GENE_SITE_251	ONE_NON-GENE_SITE_251
**GCCTTGTCATTTGACAACCG**	PRKCE	5581
**GCGAATTTCGTTGTCTCGAA**	TRAPPC2L	51693
**GCGACCCCGCGCCCCGACAG**	SHC2	25759
**GCGAGCGGTGCATCCCAACA**	DAB2IP	153090
**GCGAGGAGAGCTCCTCCGAG**	PIK3R5	23533
**GCGAGGTCATCACAGACACT**	RASA3	22821
**GCGAGTTCGACAGCTCACTG**	EXOC8	149371
**GCGAGTTGCAGATCTACACT**	SAP18	10284
**GCGATGATGAGCACCTCGAA**	LGALS7	3963
**GCGATGATGAGCACCTCGAA**	LGALS7B	653499
**GCGATTACAGCCGACCCCCG**	RASAL1	8437
**GCGATTGCAGAAGATGACCT**	VHL	7428
**GCGCCAATTCACTACCGCGA**	ONE_NON-GENE_SITE_252	ONE_NON-GENE_SITE_252
**GCGCCCAGCTGCCAACACCA**	RPS8	6202
**GCGCGCGCGTGCGCGGTTGG**	NRTN	4902
**GCGCGCTCACCAGATGAAGT**	PRKCB	5579
**GCGCGGGAAGTCGGCGCTGC**	TRAPPC5	126003
**GCGCGGGCCAGAGTCATAAG**	FAM20B	9917
**GCGCGTCAGAACAGGCGCTG**	LPCAT3	10162
**GCGCTCCGGCCTCTACTCGG**	DUSP4	1846
**GCGCTTCCGGCTGCCCCTCC**	UBE2Q2L	100505679
**GCGGAACCGAACCCTATCCG**	ARHGEF2	9181
**GCGGACACTGTGCTGCGACA**	MARCH2	51257
**GCGGACCCACGAGAGCGCGT**	RASSF10	644943
**GCGGACGAAGTGCCTCACCA**	NLRC3	197358
**GCGGATGAAGAAAGGTCCAA**	TGIF1	7050
**GCGGATGCCCTCGGGCAGTG**	LGALS7	3963
**GCGGATGCCCTCGGGCAGTG**	LGALS7B	653499
**GCGGATGTGCTGGTAACCTA**	MLST8	64223
**GCGGCAGGCAGCAACGTAAG**	EXOC8	149371
**GCGGCCCAGGCCGTGGAATG**	RTN4R	65078
**GCGGCCCGCAACGTGCCCGT**	UNG	7374
**GCGGCGAGGTCCTGGCGACC**	BCL2	596
**GCGGCGGAGCACTTCACCCA**	VKORC1L1	154807
**GCGGCGGCTGGACGCATCAG**	PCDH7	5099
**GCGGCTCCTAGACCGGCAGG**	MED11	400569
**GCGGGCCAGACAGGGATGTG**	PRPF38B	55119
**GCGGGCCTGCAACGGACTCA**	TMEM220	388335
**GCGGGCCTGCAACGGACTCA**	TMEM220-AS1	101101775
**GCGGGCGTAGAGGACCGCGT**	OSGIN1	29948
**GCGGGCGTAGTTGTTGTGGG**	RASGRP4	115727
**GCGGGGACTCACAGCCATGT**	MAP2K2	5605
**GCGTCTTCACGTACTCGGTG**	SLC25A1	6576

**GCGTGCTGACTGAATGTGCC**	RIC8A	60626
**GCGTGGCAATCAGGTAGAAG**	MAF1	84232
**GCGTGGCCGGCACCCCCGGG**	SOX9	6662
**GCGTGTGCGCATCAGCTGTG**	RASSF2	9770
**GCGTTGCGGCTACTACAACA**	MAEA	10296
**GCTAACATGTTGCCAATCAG**	NF1	4763
**GCTACTCACCCAACCCTCGA**	PRKCH	5583
**GCTACTGGAAGGCCACGCAC**	PIK3CG	5294
**GCTACTTCTAGCTCTGAACC**	RALGDS	5900
**GCTAGATGAGGCTCTAAAGG**	EXOC1	55763
**GCTATGCCTGTACCTAACCA**	TUBGCP2	10844
**GCTATTACTCAAGAAATAGT**	VTA1	51534
**GCTCAACTCCAACCACGACG**	SET	6418
**GCTCACCCAAGACCGAGACT**	DNAJC9	23234
**GCTCAGCACTACGCGCGCCT**	RASSF10	644943
**GCTCAGTGAAGCCATGAGCG**	CYTH2	9266
**GCTCATAAATATAATCCCAG**	POLR1A	25885
**GCTCATAAGAAAACCTGGTC**	SLC11A2	4891
**GCTCATTTACGGAGCATGCG**	SLC50A1	55974
**GCTCCACACTAAATCCGCAG**	PRKCA	5578
**GCTCCAGGCCATGAACTTTG**	UAP1	6675
**GCTCCAGTGTGTCAGTTAGC**	RIC8A	60626
**GCTCCCGCTTCATCTTGTTG**	NEDD9	4739
**GCTCCGAAACTCCAAAGGAA**	PRKCA	5578
**GCTCCGATATGCCAAGCTGA**	EXOC1	55763
**GCTCCTTTGACTTCGACAAG**	HDAC5	10014
**GCTCGAAGATTGAGATCTGG**	FAM217B	63939
**GCTCGCTGTTCAATTTCAGC**	MET	4233
**GCTCGGCGTTGTGCAAGTGC**	SOX9	6662
**GCTCGTCGTTGGCACTCCTT**	CBLC	23624
**GCTCGTGAGGCTCTACGCTG**	LYN	4067
**GCTCTACGCCATGCACCCGT**	PIK3CG	5294
**GCTCTAGATGGCATGCACGA**	ONE_NON-GENE_SITE_253	ONE_NON-GENE_SITE_253
**GCTCTCACCTGAGGTCTCGT**	SET	6418
**GCTCTCCAGAAAATTGATGC**	CCDC6	8030
**GCTCTCCAGGAAGCTAAACC**	PEA15	8682
**GCTCTCTCCTGCCAGAACCG**	KIR2DL3	3804
**GCTCTGCGCCAGCTTGACGA**	EXOC7	23265
**GCTCTGCTGGAGGGTCCGTG**	E2F3	1871
**GCTCTGTGGACCCCACACTG**	HDAC5	10014
**GCTCTTCAGCTACATGCAGT**	BSCL2	26580
**GCTCTTCAGCTACATGCAGT**	HNRNPUL2-BSCL2	100534595
**GCTCTTCCTACACTATGGCA**	RASA3	22821
**GCTCTTGGGAGGGTCAACTC**	ONE_NON-GENE_SITE_254	ONE_NON-GENE_SITE_254
**GCTGACAACACATTTGGTGA**	RGL1	23179
**GCTGACCACACCAACCTACG**	TUBB	203068
**GCTGACCCACGCCGAAAAGG**	COX4I2	84701

**GCTGACCTTGAGATCACAGG**	MAPK1	5594
**GCTGACTACATTTGAAGGGA**	FAXDC2	10826
**GCTGACTCGACGGTGATGGG**	MMP9	4318
**GCTGAGCCCATGCAGCACAG**	RASAL2	9462
**GCTGAGTCTTCGGTCCGACA**	RALGAPA2	57186
**GCTGATATTAGACTAAGCTG**	STAM	8027
**GCTGATTTCCAGTCTCCGAG**	TSPAN13	27075
**GCTGCAGAACGGGCCCAAAG**	FLVCR1	28982
**GCTGCATAGCTGTTCTACAT**	ONE_NON-GENE_SITE_255	ONE_NON-GENE_SITE_255
**GCTGCATGAACTCGAAGGCG**	C4orf48	401115
**GCTGCATGTCACCTACGCCG**	PEBP1	5037
**GCTGCCACAAGAGTGTCCAG**	RASSF7	8045
**GCTGCCGGAGCAGTCACACA**	MARCH2	51257
**GCTGCCTTACCCGAACGTTG**	RASAL3	64926
**GCTGCGCCTGCACCCCGGAG**	BIRC5	332
**GCTGCGCGAGCTGACGCCCT**	NRTN	4902
**GCTGCGTAGAGCAGTAGTAT**	ONE_NON-GENE_SITE_256	ONE_NON-GENE_SITE_256
**GCTGCTATGGCGTATGGCAT**	SPTLC2	9517
**GCTGCTGCTCAGCGTGATCC**	INIP	58493
**GCTGGAAAGTGACCTCAAAG**	TP73	7161
**GCTGGACAACTTAACATGCG**	SLC30A1	7779
**GCTGGACCTCCGCGACGACA**	RAC3	5881
**GCTGGACCTGCGGGACGACA**	RAC2	5880
**GCTGGACGGTACATTTCCCC**	SAP18	10284
**GCTGGACGTATGATCGTAGC**	ONE_NON-GENE_SITE_257	ONE_NON-GENE_SITE_257
**GCTGGATCCTCCGAGAATCG**	ATP2A2	488
**GCTGGCCAAGAGCCTAAAGG**	CDC37	11140
**GCTGGCCCAGAAACCGCATA**	ONE_NON-GENE_SITE_258	ONE_NON-GENE_SITE_258
**GCTGGCGGACACGTTGAGGA**	DUSP2	1844
**GCTGGGAGCGGTAAAACCGG**	NOX1	27035
**GCTGGGATCTAACCGCATCC**	PPP1R7	5510
**GCTGGGCACCTAGGACATCG**	SIRT1	23411
**GCTGGTCCGACTCTACGCAG**	BLK	640
**GCTGGTGAAACACACGCAGG**	VAV1	7409
**GCTGGTGACCCCCGGAATGG**	EIF4EBP1	1978
**GCTGGTTCGGCTCTACGCTG**	LCK	3932
**GCTGGTTCGTACCAAGACCC**	RPS8	6202
**GCTGGTTCGTACCAAGACCC**	RPS8P8	728553
**GCTGGTTCTGAAGTCTGAAG**	LONRF1	91694
**GCTGTAACACCATCGTGCGG**	DUSP4	1846
**GCTGTACGTGAATAGTGTGA**	TIAM1	7074
**GCTGTCACTGAACCAAGTCA**	FERMT1	55612
**GCTGTCCGCACCGTTCTCAG**	ETS2	2114
**GCTGTGAAAAGAAATCATAG**	BARD1	580
**GCTGTGATGTTCCCGTTCTG**	RASSF4	83937
**GCTGTGCTGAAGTCATAGTC**	MAF1	84232
**GCTGTGGATGTTGATCACGG**	IFITM1	8519

**GCTGTGGGCCAAATCCCCTG**	QPRT	23475
**GCTGTTCCGGGAAAACACAT**	RASAL3	64926
**GCTGTTCGGGACTATCACAT**	VPS16	64601
**GCTGTTGTCAGGTCGTGCTG**	EBP	10682
**GCTGTTTAACTACACTCCTG**	RIC1	57589
**GCTTATATGCCACTCAACTG**	SOS1	6654
**GCTTATGCCCAGATTTGAAG**	RALBP1	10928
**GCTTATGGATAGTTACAACT**	JAK2	3717
**GCTTCAAGAAGCGTGCACCT**	RPL31	6160
**GCTTCAGCACTAAGAAATTG**	RPS6KA6	27330
**GCTTCAGCTGTCCGCCACAA**	RASSF9	9182
**GCTTCAGGACTCAAAAACTG**	RPS6KA3	6197
**GCTTCCAACATTGACCCGCT**	ONE_NON-GENE_SITE_259	ONE_NON-GENE_SITE_259
**GCTTCCAACTGGCTGAAGGT**	PDGFRA	5156
**GCTTCCAAGTAGCACTCAGT**	MAGEA12	4111
**GCTTCCCTTGATCTGACTGG**	FOS	2353
**GCTTCGGCCAGACTATACTG**	ONE_NON-GENE_SITE_260	ONE_NON-GENE_SITE_260
**GCTTCTGGTTTATGCCACAA**	PRKCQ	5588
**GCTTCTTAGCAAATTCCGCA**	FUNDC2	65991
**GCTTCTTCACGTCGCTGTAG**	CXCL14	9547
**GCTTCTTGCTCCAATCAACA**	BLK	640
**GCTTGAACAGGGCCCGCAAG**	RPS6KA1	6195
**GCTTGAACCGGGATTAGCTA**	ONE_NON-GENE_SITE_261	ONE_NON-GENE_SITE_261
**GCTTGCACAGACAGTCGGTA**	ONE_NON-GENE_SITE_262	ONE_NON-GENE_SITE_262
**GCTTGGTGATGATGTACACT**	NPRL2	10641
**GCTTGTTATAGTAATAGCTG**	ERF	2077
**GCTTTAACCTCAGCATCCGA**	HDAC3	8841
**GCTTTCCTGTTAGTCCATTG**	SNRPF	6636
**GCTTTGGCCACAAAATTGAA**	LCK	3932
**GGAAAAACTAGAGTTCATCC**	FOS	2353
**GGAAACCAGACATTTATGAG**	MCM4	4173
**GGAAACCTAATTGCATATGG**	NUP37	79023
**GGAAACTATAGATGCGGGCA**	CDK6	1021
**GGAAACTGACCATCAGTACC**	E2F1	1869
**GGAAATTCCTGATGGAGTGT**	EIF4EBP1	1978
**GGAACACTGTCCATTGGCAT**	PIK3CA	5290
**GGAACAGACGAGTACAACAG**	PRKCSH	5589
**GGAACAGATTCTCCGTGTGA**	RALB	5899
**GGAACAGTTCCATGCCTTCG**	STAMBP	10617
**GGAACCAACGCTCTACCTGG**	CERS2	29956
**GGAACCCAATGCCAACCATG**	FGFR2	2263
**GGAACCCCTGCAAAGCAGCA**	KPNB1	3837
**GGAACCGGAGCAGTTCGGCG**	DTYMK	1841
**GGAACGTGATATCTTGGTAG**	RPS6KA3	6197
**GGAACTCAGACTCCAGGTCA**	MAGEA6	4105
**GGAACTCGCACTTGGATGCA**	DUSP5	1847
**GGAAGAAGACGAGTTCATTG**	RASSF2	9770

**GGAAGACCGAATATGCCCTG**	RIC1	57589
**GGAAGAGGTCGGCGTCTTCG**	PRKAB1	5564
**GGAAGCAGAGATATACCAGA**	ACVR1B	91
**GGAAGCGAATCAATGGACTC**	CASP3	836
**GGAAGCGGCACATGTCATGT**	HDAC3	8841
**GGAAGGCACCCAGATCCGGA**	ERF	2077
**GGAAGGCCCTGGTCGACCAG**	TRAPPC2L	51693
**GGAAGGTGGGTAGCTCACGG**	FAXDC2	10826
**GGAAGTAACACGCACCAGCT**	RPS8	6202
**GGAAGTATTAGGGTACAAAG**	SOS2	6655
**GGAAGTCCTTCTCGCTCAAG**	SAV1	60485
**GGAAGTCTGTATGCTAGACA**	TM9SF3	56889
**GGAAGTGCCCGACAGGACTG**	E2F2	1870
**GGAAGTTGCTGCAAATACGA**	LIPK	643414
**GGAAGTTTGGAGCATGCGGA**	CD1E	913
**GGAATGCCCCAGAGCGTGCG**	C9orf116	138162
**GGAATGGATTAGCCACATCG**	PLEKHF1	79156
**GGAATGGCTAAATTGGTGAG**	PLCXD3	345557
**GGAATTCATCGTGTACCCGG**	INSR	3643
**GGAATTTACCAAGTCCCCAC**	NEDD9	4739
**GGACAAGATCTACCTCATCG**	GINS2	51659
**GGACAAGCACAGTCTTCGGA**	NSD2	7468
**GGACAATAAATACGACGCTG**	USP7	7874
**GGACAGCAATTATCTTTGAG**	EXOC6	54536
**GGACAGTTCTGTTTCTCTTC**	LOC110117498-PIK3R3	110117499
**GGACAGTTCTGTTTCTCTTC**	PIK3R3	8503
**GGACATCATCCTGTACGCGT**	FGFR4	2264
**GGACATGGAAACCATCAACC**	PPP1R7	5510
**GGACATGGTGACACTGTAGT**	YES1	7525
**GGACATTCCCATCACTTACG**	HCFC1	3054
**GGACCAGATGAGCAGCCGCG**	TP73	7161
**GGACCAGGAGAATATCAACC**	CCNA2	890
**GGACCAGTAACCAGGCTTAC**	C9orf116	138162
**GGACCATGGACACTTAAGAC**	ONE_NON-GENE_SITE_263	ONE_NON-GENE_SITE_263
**GGACCCGATGCAGACCTTCG**	TTC7A	57217
**GGACCCTGACACTGTGATCC**	AUP1	550
**GGACCTCAAACTGTTAACCG**	E2F3	1871
**GGACGAGGGCAGTGCCTCGG**	DUSP2	1844
**GGACGAGTGGGAGGTTCCCA**	LCK	3932
**GGACGAGTTTGAGAACATGT**	PAK4	10298
**GGACGTGAATGGCAGGTACA**	TRAPPC4	51399
**GGACGTGAGATATGCGTCCG**	PLXNB1	5364
**GGACGTGTCCCGGAACGGTG**	SCRIB	23513
**GGACGTTCTCATAAGAGGCT**	RAC2	5880
**GGACTAAATGATTATCCCGC**	ARHGAP35	2909
**GGACTACCAGCACGAGAATG**	PAK4	10298
**GGACTACCCCGAGTACATGT**	AGPAT3	56894

**GGACTATTCCAACAGCCCTG**	ELOVL1	64834
**GGACTCGGATGACGTGCCCA**	HRAS	3265
**GGACTGACTCAAGTAATTCG**	MON2	23041
**GGAGAGGAGATTGTCCGAGT**	SPRED2	200734
**GGAGAGGATATGTAGCGAGG**	CHCHD3	54927
**GGAGAGTCAAAGCCTCCTCG**	TSC1	7248
**GGAGAGTGTGGGTTGGAACA**	VPS18	57617
**GGAGAGTTCTCAAGTAGATG**	BPTF	2186
**GGAGATCCGAGACTTGTTGG**	CNKSR1	10256
**GGAGATGAGTTCTGAGCGGT**	YPEL5	51646
**GGAGATGATCAGGTCAAGGA**	CHMP3	51652
**GGAGATGATCAGGTCAAGGA**	RNF103-CHMP3	100526767
**GGAGCAACGCAAGTTCATCT**	LMTK3	114783
**GGAGCAATACATGCGCACGG**	MRAS	22808
**GGAGCACTATTTCCACGCCG**	PIK3R6	146850
**GGAGCATGTGTATCAAACTG**	STAM	8027
**GGAGCCCATTGAGAGCGATG**	HDAC4	9759
**GGAGCCCATTTACATCGTCA**	SRC	6714
**GGAGCCGCTTCAGATATGCA**	CISD2	493856
**GGAGCTGCACCTTCACGATA**	CISD2	493856
**GGAGGAAGAGTACCTCGGAA**	PRKAB1	5564
**GGAGGAATGGACGAACTGCA**	CISD2	493856
**GGAGGAATGGACGAACTGCA**	SLC9B1	150159
**GGAGGACTGTGTCGTCGATG**	KLHDC3	116138
**GGAGGAGCTGATTAACTCAG**	TSC2	7249
**GGAGGCAGGCTACACTTCCA**	DHX8	1659
**GGAGGGAGGCGCTGTAATGG**	SCD	6319
**GGAGGGAGGGTATTACCGGA**	E2F3	1871
**GGAGGGGACACTCACAGTCA**	ANAPC11	51529
**GGAGGGGCAACATACCAATG**	VPS16	64601
**GGAGGGTATGACCTCCCTCA**	SRSF2	6427
**GGAGGTGTTACGGATGTGTG**	VPS39	23339
**GGAGGTTGGTTGGAACAATA**	TSPAN13	27075
**GGAGTACGTGACTCGTCACC**	PEX16	9409
**GGAGTACGTTGCCTGACACC**	PRKAG3	53632
**GGAGTCGCGCGTTACCCAGG**	SAP18	10284
**GGAGTGTGATGATATATCTG**	RASGRF2	5924
**GGAGTTAAACACCTCTTCGG**	ARHGAP35	2909
**GGAGTTCATTGGATGCGCGG**	KSR1	8844
**GGATACTATTACACTGCCAC**	LPCAT3	10162
**GGATACTCACGGTGTAAAGC**	TSPAN13	27075
**GGATATAAAAGACACCCCCT**	PRKAA2	5563
**GGATATGAAAAATACTCGTA**	EXOC4	60412
**GGATATGGATGCCTATACCC**	CDC23	8697
**GGATCAGTTGGAACTGACGG**	ESRP1	54845
**GGATCCCACTATGTCGCCAC**	ONE_NON-GENE_SITE_264	ONE_NON-GENE_SITE_264
**GGATCGTCGCAATTCTCCCA**	EIF4EBP2	1979

**GGATCTCTCCTGGCAAAGAC**	PSMD11	5717
**GGATGAAAACTACAAATCCC**	INPP5A	3632
**GGATGCATGCGGCTAGACAT**	IRF2	3660
**GGATGCCTACCGGGTACCGT**	KSR1	8844
**GGATGCGCCATAGTAATACG**	NLRC3	197358
**GGATGGACCAGAATACGAGG**	RASA1	5921
**GGATGGGTCATGCATCAATG**	KDM3B	51780
**GGATGGTAAAGAGTGGATGG**	RASGRF2	5924
**GGATGTATATCAGCATAAGG**	STK3	6788
**GGATGTGAAACTGAAACGCC**	STK4	6789
**GGATGTTCCAGATGGCACTC**	RUNX1	861
**GGATTAAAGCCTAGTAGTGG**	MON2	23041
**GGATTACAACGACAAGTACT**	TUBGCP2	10844
**GGATTCCCGAGTACACGGAA**	INPP5A	3632
**GGATTGAATTCATTATGACG**	SMAD5	4090
**GGATTTGCTCCGATGCAGCG**	KSR2	283455
**GGCAAAGATCCCCATCACAG**	PCDH7	5099
**GGCAAATGAGCCCAACCACG**	VPS18	57617
**GGCAACCTTTCAGTTCACTG**	USP7	7874
**GGCAATGTGCTCCCGCTCTG**	PRPF38B	55119
**GGCACCAGGTAGTCCACCAT**	NFKB1	4790
**GGCACCCTGGATCCCCGACA**	PLEKHF1	79156
**GGCACGAACATTCTCGAAGG**	RAC3	5881
**GGCACTCACCTCCTTGGCAC**	DTYMK	1841
**GGCACTCACGATGGTCACGC**	CCDC6	8030
**GGCACTGCACCCGTTCGCGG**	STK11	6794
**GGCAGCGCTTACGGACAGAA**	EXT2	2132
**GGCAGGAAGACCCTCTGCAA**	TBC1D3B	414059
**GGCAGGAAGACTCTAACGAT**	BRAF	673
**GGCAGGTCTCCTCCATACGT**	ONE_NON-GENE_SITE_265	ONE_NON-GENE_SITE_265
**GGCAGTAAATACGGGCCCGA**	FGFR2	2263
**GGCATCCAGCGTGTGGTCTG**	RASSF7	8045
**GGCATCCCCATTATTAAAGG**	SOS2	6655
**GGCATTATAGGCCTTGCAGT**	POLR1A	25885
**GGCATTGTAGGGCTCGACCA**	TUBB	203068
**GGCCAAAAACTTGTGATCTG**	MCM4	4173
**GGCCAAGAGCCTTTGATCAG**	SPRY3	10251
**GGCCACCATGAACTTGGCAC**	PREB	10113
**GGCCAGATGCTGTGTAACCT**	ETS2	2114
**GGCCAGCCACGAGTCCATTG**	RIMS4	140730
**GGCCATACCTTGTCTCACAA**	RASGRF1	5923
**GGCCATGTCACCCTTCAGCA**	RASGRF1	5923
**GGCCCAGCTGTACTCGCCTG**	CBLC	23624
**GGCCCATCCCCTACCAGCGA**	MAP2K2	5605
**GGCCCGCACTCATACCACTG**	RPS6KB2	6199
**GGCCGCCGACACCAATGAAG**	INPP5A	3632
**GGCCGCTGGATGTACACGGG**	MLST8	64223

**GGCCGGAACGGGTTAAGCGA**	ONE_NON-GENE_SITE_266	ONE_NON-GENE_SITE_266
**GGCCGGACCTCAGACCGGAG**	NEDD9	4739
**GGCCGTGCAGCAGTGCATTG**	VPS16	64601
**GGCCTTCGACGTACCCCTGG**	FOSL1	8061
**GGCCTTCTTTGAGTTCGGTG**	BCL2	596
**GGCGACCTTTACCGATGGAG**	PSMD11	5717
**GGCGAGCGTGATGTCCGCAT**	HDAC11	79885
**GGCGCTGCGACGACTTGCTG**	RASSF10	644943
**GGCGCTTAGAGAATTGAGAG**	CDC23	8697
**GGCGGCGCAGCCGGTCAACG**	JUN	3725
**GGCGGTGAAGAAGAAGTCCT**	RASSF3	283349
**GGCGTGGTGCTGTAGCAGTC**	EIF4EBP3	8637
**GGCGTTAGCCACCTTGCAGG**	SMU1	55234
**GGCGTTGTGAAGACCATGAC**	FOS	2353
**GGCTATCTGGTATCTGTAGA**	SNRPF	6636
**GGCTCAGGGTCATTGAGGAG**	FOS	2353
**GGCTCAGGGTTACCGAAGAG**	BRCA1	672
**GGCTCCTACCCTGTAGATGA**	TBCB	1155
**GGCTCTTACCTGTAGCACAG**	CCND3	896
**GGCTGGCGCAGGCGCTTGTG**	PREB	10113
**GGCTTACCACTGTCAGCACG**	VPS18	57617
**GGCTTCCGTTGCCAAACCTG**	ARAF	369
**GGCTTTACTGAGGCGACTGG**	CDK4	1019
**GGGAAACAAGTCTGACCTAG**	RALB	5899
**GGGAAATACTATCAGATTTG**	MDM2	4193
**GGGAACCAGCATCAATCAGA**	LUZP4	51213
**GGGAACGGCGGCGGGTACGG**	FOSL1	8061
**GGGACAGAAGTGCTTCACCT**	RHOB	388
**GGGACTAAGAGATACCTACA**	SPC25	57405
**GGGACTATTGAGTAGAGGCT**	AUP1	550
**GGGAGATCAAGCCGCACATG**	CCND3	896
**GGGAGCCGTGCTCCTCCAAG**	LMTK3	114783
**GGGAGCTAATGTCAGCCGTG**	DUSP5	1847
**GGGATAGGGAGCCAGACCGG**	FAM71E2	284418
**GGGATGAGCTTCGGAAACTG**	EXOC4	60412
**GGGATGCATGGTTCCTGAGG**	SPRR1B	6699
**GGGATGGCCCCGTAAGAACA**	CNKSR1	10256
**GGGATGGCGCGGCTCTGGCT**	EIF4EBP2	1979
**GGGATGTTCTCCAGCGAGTC**	RHOB	388
**GGGCAAAACGGCTCAGCCAT**	MTAP	4507
**GGGCAACCTGTCGAGCTGGG**	GATA6	2627
**GGGCAAGAGGCGGATGGACA**	UBE2H	7328
**GGGCAAGGGCACTTTCGGCA**	AKT1	207
**GGGCACAAGGTCCTACATGT**	MAP2K1	5604
**GGGCAGCACCCACCACATGA**	PIK3CD	5293
**GGGCAGCTTTGTCCAACGCA**	KLHDC3	116138
**GGGCAGGAAGAAGTTCAACA**	CYTH2	9266

**GGGCAGTCGTCGGGGCACAG**	MESP1	55897
**GGGCATGAACTACTGTGTGA**	SHC4	399694
**GGGCATTGAAGTGACGTTTG**	RPL26L1	51121
**GGGCCCCAGGAACTCCACAT**	PIK3R2	5296
**GGGCCTCCTGGGCCACGTCT**	TK1	7083
**GGGCGCACTGCCGTCCGGGG**	SHC3	53358
**GGGCGCAGCCTCGGTCAAAG**	SPRED3	399473
**GGGCGGGTGCTCTTAACGCG**	SLC30A1	7779
**GGGCGGTCGCATGTACCCTG**	CECR2	27443
**GGGCGTGCTGCTACTACTTG**	HDAC7	51564
**GGGCGTGGAGCAATCACTGG**	PAK1	5058
**GGGCGTTGGGCAGTATACAT**	NPRL2	10641
**GGGCTACGCGTCCGACGAGA**	NRTN	4902
**GGGCTCATTGTAGCATACGC**	RTN4R	65078
**GGGCTGAAATTGGACCCCGC**	SLC30A1	7779
**GGGCTGCCAGCGTATCGTAG**	MTOR	2475
**GGGCTGGAAACAAGGTGATG**	SMAD5	4090
**GGGCTGTTCTGTAGTCGGGG**	HCFC1	3054
**GGGCTTCGACGACTAAGTAG**	ONE_NON-GENE_SITE_267	ONE_NON-GENE_SITE_267
**GGGGAATACTCCAGCTCACA**	ALK	238
**GGGGAGAGGACACTTACCGA**	RASGRP4	115727
**GGGGATGGAACCGTAGAGCA**	SLC25A1	6576
**GGGGCAACGAGGTTACCTGT**	YAP1	10413
**GGGGCACGTTGGCCGTCTTG**	YAP1	10413
**GGGGCCGCGAACTTCCACTG**	SHC2	25759
**GGGGCGGTGGCTACGGACGC**	MFSD11	79157
**GGGGCGGTGGCTACGGACGC**	SRSF2	6427
**GGGGCTGACTTACCGCAGAG**	HDAC4	9759
**GGGGGAAACTCATCTATCAG**	VPS25	84313
**GGGGGAAGGCTCTTACGGCA**	STK11	6794
**GGGGGCGTGTCGCTTCCTGG**	UNG	7374
**GGGGGGTAGAGTCTGATCAG**	AKT2	208
**GGGGTCTCAACGCACACCCG**	FLT3	2322
**GGGGTCTTGGGATGAATGCC**	SLBP	7884
**GGGGTGGTTTACATACTACG**	ONE_NON-GENE_SITE_268	ONE_NON-GENE_SITE_268
**GGGGTTGACAGCGAAAGCCT**	RANGAP1	5905
**GGGGTTGCGCGGGAAAGGCC**	JUN	3725
**GGGGTTTAATCAGATAGCAG**	CHD2	1106
**GGGTACATCTTCAATGGAGT**	SREBF1	6720
**GGGTACCACCGATATGATCA**	YPEL5	51646
**GGGTACGCTCCATCTCTGTG**	GID8	54994
**GGGTAGAGAACCCGGCATTG**	C12orf77	196415
**GGGTCATTCGCATGTGCCTG**	SLC50A1	55974
**GGGTCCCAGGGTCATTGTCA**	SLC22A25	387601
**GGGTGAGATCTACTCGCCAG**	ZFPM1	161882
**GGGTGCGGAGCGCAGCTTGG**	VPS18	57617
**GGGTGGAAAGACGAGAACTC**	ASXL3	80816

**GGGTGGGCACTCCACCAACA**	PRKAG3	53632
**GGGTTCTCCATTAAACACCC**	NSD2	7468
**GGGTTGGATGATGTCGGGAA**	KSR1	8844
**GGGTTTCTCTTATCAACACG**	BRCA2	675
**GGTAACCTGGGAAGACATCG**	VCP	7415
**GGTAACTGTGCCTATTCGAG**	FGFR4	2264
**GGTAAGGATATAACCTCCCG**	RAC1	5879
**GGTACAGAGGGCACGCTATG**	PSMA6	5687
**GGTACAGAGGGCACGCTATG**	PSMA6P1	64596
**GGTACAGAGGGCACGCTATG**	PSMA6P2	729412
**GGTACGGCTGAGGCCTTTCG**	FAM71E2	284418
**GGTACTCGTATACGTAGCCA**	NEDD9	4739
**GGTAGATATATGGAGCAGTG**	PRKAA1	5562
**GGTAGCAAAACAGTGTACCC**	BUB1	699
**GGTAGCACCATCATTTGTAA**	CCT4	10575
**GGTAGTACTGGAATAATGGG**	ESRP1	54845
**GGTATCCTCAACTAAACTGA**	FGFR2	2263
**GGTATCTTCAGTGTTACAAA**	TMEM263	90488
**GGTCAAACAATGTATGAGGG**	LIPK	643414
**GGTCAACTTGATGCCTGAAG**	LCK	3932
**GGTCAGCATGAGGCTCCTGG**	CXCL14	9547
**GGTCATTAAATCTTGCAACC**	RUNX1	861
**GGTCCAGTCAGTGAACGTAA**	NF1	4763
**GGTCCCCATTACGTCCCGAG**	PLAGL2	5326
**GGTCCCCGATCTTAAAACGG**	CRKL	1399
**GGTCCGCACAAAAGTCTGAT**	ONE_NON-GENE_SITE_269	ONE_NON-GENE_SITE_269
**GGTCCTGCACAATGTAGCTG**	MAPK3	5595
**GGTCGAATTCCTCGCCGAGA**	GINS2	51659
**GGTCGCAGGGTGCTCCACTG**	ELK1	2002
**GGTCGTACAAGCTGTAGTGT**	PSMD3	5709
**GGTCTCCGACACGTTGCGCG**	PDGFRB	5159
**GGTCTCGGAGAATGACCGAG**	ETS1	2113
**GGTCTCTGTAAATGATACCC**	RPS6KA1	6195
**GGTCTTCTGTCAAAAACACA**	NSD2	7468
**GGTCTTGATCACCCGCACTA**	NPRL3	8131
**GGTCTTGGTGCGATAACTGG**	PAK3	5063
**GGTGAACCTTCATGGTTGCA**	GAREM1	64762
**GGTGAAGTTGAATCGACTCA**	EXOSC9	5393
**GGTGACCGTACCTCTCCCCC**	CYTH2	9266
**GGTGACTTCCACTAGAGTAG**	MAGEA12	4111
**GGTGAGATTAAGCAAATCGC**	ONE_NON-GENE_SITE_270	ONE_NON-GENE_SITE_270
**GGTGAGGCAAGACATGAGAG**	IRS1	3667
**GGTGCACACATCTCTGGACG**	TRAPPC2L	51693
**GGTGCAGAAATTCAAAAACC**	CDK2	1017
**GGTGCAGAACGTTAGCTGAA**	MAPK1	5594
**GGTGCAGAGAAACCGGCATG**	TFDP1	7027
**GGTGCCATGACAACAAGTCC**	MAEA	10296

**GGTGCCTTTCACAACCAACG**	HCFC1	3054
**GGTGCGCGCAAAGACGAAGG**	INSRR	3645
**GGTGCTCTATGCTTCACCAT**	ONE_NON-GENE_SITE_271	ONE_NON-GENE_SITE_271
**GGTGCTGAATGCCTCCCACG**	FGFR3	2261
**GGTGCTGCCCCATGAGTCTG**	MESP1	55897
**GGTGCTTTGCGATTCACAGG**	FLT3	2322
**GGTGGAAGATCTCGAAGTGT**	CBL	867
**GGTGGAGGACCCGATGAGAA**	CDK2	1017
**GGTGGAGGCCCTGACTACCC**	FAU	2197
**GGTGGATTCGCCAAGGCGAA**	ALK	238
**GGTGGCCAATTCGATTCGGA**	POLR2C	5432
**GGTGGGAAGCGATACTGCCA**	VPS25	84313
**GGTGGGAAGTTCAACAGCTG**	CDC6	990
**GGTGGGCTGCCGAAGATAGG**	PRKCE	5581
**GGTGGGTGAGGGCGACAAGG**	DNAJC9	23234
**GGTGTAGCTGATGAGAAGGC**	RAC2	5880
**GGTGTCAGCTCTCACCGGAG**	BIRC7	79444
**GGTGTCGACCAGAACGTCCT**	PCDH7	5099
**GGTGTTTCACATAGGCCAAT**	IFIT5	24138
**GGTTCACACCCCGAGTGTCA**	KLHDC3	116138
**GGTTCAGAAACAAATCGAGT**	APC	324
**GGTTCATCTCATGCAAACTG**	TIAM1	7074
**GGTTCTAATACAGGCTCTCG**	ONE_NON-GENE_SITE_272	ONE_NON-GENE_SITE_272
**GGTTCTTAAAGATCTCATAG**	EXOC3	11336
**GGTTCTTTGAGCAACATGGG**	RB1	5925
**GGTTGAACTCCATGGCAATG**	PTGS1	5742
**GGTTGATCTTACCAGGCAAG**	FAM71E2	284418
**GGTTGATGCTGGCACACTCG**	PPP1CA	5499
**GGTTGGCATCGGGGTACGCG**	CCND1	595
**GGTTTACAAAAGTCACTGTG**	GRB10	2887
**GTAAAGATCTCTTCACACAG**	CDC25A	993
**GTAAAGTGAATAGCGGCTAC**	ONE_NON-GENE_SITE_273	ONE_NON-GENE_SITE_273
**GTAAAGTTGGTGGTACATCA**	BARD1	580
**GTAAATCACCTGATGCGGTA**	ONE_NON-GENE_SITE_274	ONE_NON-GENE_SITE_274
**GTAAATGTGCCCATTCAGGA**	HDAC8	55869
**GTAACAACAGGGATTCACTG**	PTGS1	5742
**GTAACATAGGCTTAAAACGG**	EMC3	55831
**GTAACCAAAGCTGATTGACT**	FLT3	2322
**GTAAGAGTCTAAACGCATCC**	ONE_NON-GENE_SITE_275	ONE_NON-GENE_SITE_275
**GTAAGCCATTACCTGTGACC**	GID8	54994
**GTAAGGAGTGCACCTACCCA**	SPRY2	10253
**GTAAGGTATATAATGACCAG**	UAP1	6675
**GTAATCCTGAGAGATTCACA**	RPS13	6207
**GTAATCTGGGTAAATCGTCC**	ONE_NON-GENE_SITE_276	ONE_NON-GENE_SITE_276
**GTAATGAACAGTTTATATAG**	PRKAA2	5563
**GTACACGTATCGTATTTGCC**	ONE_NON-GENE_SITE_277	ONE_NON-GENE_SITE_277
**GTACACTCCAAGGTTGTCTG**	SPRED3	399473

**GTACATTGTATAAGCGCTGT**	ONE_NON-GENE_SITE_278	ONE_NON-GENE_SITE_278
**GTACCAGGATCATCTATGAC**	EIF4EBP1	1978
**GTACCCCCAAAATAAAAACA**	ASXL3	80816
**GTACCTGGACACGCTCTTGG**	CXCL14	9547
**GTACGCTTCCCAGAACCACC**	EXT2	2132
**GTACGTAGCGCTCCTCCAGG**	CCND3	896
**GTACGTATCGGAGTTATAGC**	ONE_NON-GENE_SITE_279	ONE_NON-GENE_SITE_279
**GTACGTGTAAATCTGTCCCG**	DPF1	8193
**GTACTCCCAAGTAATGCCAG**	SPRY1	10252
**GTACTGCATTGCATATTATG**	ST3GAL6	10402
**GTACTTGTGACAAATTAGCA**	FLT3	2322
**GTAGAAACCGTAGCGCTTGA**	ARSI	340075
**GTAGAATATGACCCGTTGAG**	ONE_NON-GENE_SITE_280	ONE_NON-GENE_SITE_280
**GTAGACGTTCATGAAATCTG**	EXOC7	23265
**GTAGAGTGGTGAGATCAAGT**	KDM3B	51780
**GTAGATCCGTTTGATCCTAG**	CBL	867
**GTAGATGGCGAGCGCCTCGA**	RIMS4	140730
**GTAGCAATTAACTGGTCTGG**	HDAC8	55869
**GTAGCCATAAGGTCCGCTCT**	JUN	3725
**GTAGGATAAGCCAGAGACAT**	MSMO1	6307
**GTAGGGGTCCGTACTCAGCA**	MED12	9968
**GTAGTACTACGTTTGCTGAA**	RABGAP1	23637
**GTAGTCAAACAGCATGAGGT**	PIK3CD	5293
**GTAGTCCCGAATGTATCCCA**	ONE_NON-GENE_SITE_281	ONE_NON-GENE_SITE_281
**GTAGTGACCCTCAAAATGGT**	DUSP1	1843
**GTAGTGATGGAACCCCCCGG**	ARAF	369
**GTAGTGCGGGAGCCGCCAAG**	SPNS1	83985
**GTAGTTAGGAATACGCCAAG**	ONE_NON-GENE_SITE_282	ONE_NON-GENE_SITE_282
**GTAGTTCATACATATAAAAC**	CSNK2A2	1459
**GTAGTTGTCCTGAAATTCAA**	GRB10	2887
**GTATAATTCCAACCTTGAAG**	RASSF8	11228
**GTATACAGCTTTGATCCAGT**	PSMB1	5689
**GTATACCGTATGTCTATACA**	ONE_NON-GENE_SITE_283	ONE_NON-GENE_SITE_283
**GTATAGCCGAGACCATGTGG**	FAM20B	9917
**GTATCAGTACCCGAACAGAA**	DHX8	1659
**GTATCTGCTGCGTATAACAT**	WDR26	80232
**GTATGAAAACCCAATAGAGT**	BRCA2	675
**GTATGGCTATGAGATTCCTG**	PSMA6	5687
**GTATTCCTGCAGTTACCTCG**	PAK1	5058
**GTATTGTTGGGAAATTCCCG**	APC	324
**GTCAAACAAGATACAACAGT**	VPS11	55823
**GTCAAAGAACTGGACATCGT**	RIC8A	60626
**GTCAACAGACATTAGGACAG**	ASXL3	80816
**GTCAATATGAGCAACTGGCT**	CCT4	10575
**GTCACAATCTAGTAGATCGG**	ONE_NON-GENE_SITE_284	ONE_NON-GENE_SITE_284
**GTCACACATACTGACATACT**	HDAC8	55869
**GTCACCAGACGGCTTTAACA**	FERMT1	55612

**GTCACCCCGCGGATCTACGT**	DUSP3	1845
**GTCACCGAGACACCACTGGA**	CDKN1A	1026
**GTCACGGTCACATTTCTCGT**	EXOC4	60412
**GTCACTATGGCATTCGTGTA**	ONE_NON-GENE_SITE_285	ONE_NON-GENE_SITE_285
**GTCAGGTGGAGCTGGCTCTG**	RHOC	389
**GTCATAGTGACCACAGCTTG**	MTAP	4507
**GTCATGATTAAAGCCGTCCG**	MCCC1	56922
**GTCATGTCATATTACCGTAT**	ONE_NON-GENE_SITE_286	ONE_NON-GENE_SITE_286
**GTCATGTTGGCAGAACTCAA**	PRKCE	5581
**GTCATTACCAGCAGCTCCTG**	CCND2	894
**GTCATTCCCGATGATCCTCG**	MRAS	22808
**GTCCAAACTTGCAGTCACCC**	SPRED3	399473
**GTCCAAAGACACTGTTTGGC**	VKORC1L1	154807
**GTCCAACTGTGACTGGATGG**	EXOC3	11336
**GTCCAAGGCACGGATGACGG**	MARCH2	51257
**GTCCAATTATAAGTAAACAT**	ATP6V0A1	535
**GTCCACAGTACTTTCCCCCG**	CBLC	23624
**GTCCACATATGCAACACCTG**	CDK4	1019
**GTCCAGTTCTGTACCACGGC**	CASP3	836
**GTCCATCTGGATGTGTCGAG**	NRTN	4902
**GTCCCACATATCCTTGACCA**	GINS2	51659
**GTCCCATAAAGCATCAACGG**	ONE_NON-GENE_SITE_287	ONE_NON-GENE_SITE_287
**GTCCCCTATGATCACCAACG**	PDGFRB	5159
**GTCCCTTGAGCACAAACCAG**	CNKSR1	10256
**GTCCTGACATTACTCGAAGG**	PCDH7	5099
**GTCCTGTATGAGTGGGAACA**	CTNNB1	1499
**GTCGAGATGGCAGTGACCGT**	FOS	2353
**GTCGCTGTGTCCCAGAACAT**	DHFR	1719
**GTCGCTGTGTCCCAGAACAT**	DHFRP1	573971
**GTCGTAGCCATCTAGCCCAT**	ARSI	340075
**GTCGTCGCAGGGTTTAGCGG**	DCAF12L2	340578
**GTCGTGCTGTAGTCCCCGGG**	EIF4EBP1	1978
**GTCTACAAGAATGCTGTCCA**	SNX13	23161
**GTCTACCTGACCAATCGATG**	BRCA2	675
**GTCTCCAAGCTTAGCACACC**	MCM6	4175
**GTCTGCGTACTTCCAGACCA**	EGFR	1956
**GTCTTACATCCACAGCACAT**	CHD2	1106
**GTCTTAGGCTCCATTTGGAA**	MSMO1	6307
**GTCTTCAAAAGGAAGTATCT**	AKT3	10000
**GTCTTCAACAGCAAGGAGCA**	LGALS7	3963
**GTCTTCAACAGCAAGGAGCA**	LGALS7B	653499
**GTCTTGCGTCCCCACCCGAA**	CCND3	896
**GTGAAATGGAACTATGACTG**	RASGRP3	25780
**GTGAATACATACCACTCACA**	WDR70	55100
**GTGACAGGGAGAGACGTCGC**	ONE_NON-GENE_SITE_288	ONE_NON-GENE_SITE_288
**GTGACATCGGCTGAACGATG**	TSC1	7248
**GTGACATTGGTCCATCACAT**	ECT2	1894

**GTGACCGAGTTCATGTGTCA**	FGR	2268
**GTGACGCGAACACCTTGTTG**	DCAF12L2	340578
**GTGAGACTATTCAAGCAAGA**	CDC6	990
**GTGAGCAACAGGTATGCCAG**	ETS2	2114
**GTGAGCAATGAACTGAATAT**	ESRP1	54845
**GTGATACTGGTTCGTCCAGG**	PIK3R5	23533
**GTGATGGAGTACTGCGTGTG**	STK11	6794
**GTGATGGAGTATGCCAACGG**	AKT2	208
**GTGATGGAGTTCCTCAACGG**	PRKCD	5580
**GTGATGGTTCAGCCAAACGC**	CTNNB1	1499
**GTGATTATACTCTTACACTA**	PIK3R1	5295
**GTGCACGATCTGATGCCCGG**	YAP1	10413
**GTGCACGTTGAAAGAAAGCG**	TCF7L2	6934
**GTGCACTAGGCCTTCCAATG**	CNOT2	4848
**GTGCACTGTGATGATAGGGC**	HCFC1	3054
**GTGCAGCGTGAGAAGGCCAA**	RPL26L1	51121
**GTGCAGTCATTGAAGATACT**	MSMO1	6307
**GTGCATGATATCTTTCCCCA**	FAXDC2	10826
**GTGCCCACGTTCATAATACT**	ONE_NON-GENE_SITE_289	ONE_NON-GENE_SITE_289
**GTGCCTCCCAGGAATCAGTG**	RAB3GAP2	25782
**GTGCCTGGATCTAAACCAAG**	TTK	7272
**GTGCCTTACAAGATGTGACA**	FAM71E2	284418
**GTGCGACCCCCACCCCCCTG**	PEX16	9409
**GTGCGCGCGCTCTTCACGCG**	DUSP6	1848
**GTGCTAAGGGGGCGACAAGG**	SHC1	6464
**GTGCTGAACAAGAGCAACGA**	PFDN5	5204
**GTGCTGGGTCAAGGCCAGGA**	UBE2Q2L	100505679
**GTGGAAAGAATCTGGAAACG**	SMAD5	4090
**GTGGAACGGCAGCACTAAGG**	FGR	2268
**GTGGACATTATCCAGGCTCG**	RIMS4	140730
**GTGGACTGGTTAGTTGAAGT**	CCNA2	890
**GTGGAGAATGCACTGCTCCC**	SPRY1	10252
**GTGGAGATCGCCACTGATGG**	EGFR	1956
**GTGGAGATGGTCTAAAGCCA**	RPS6KA2	6196
**GTGGATACTTGCATTTAACG**	UBL5	59286
**GTGGATGGAGATGCTCATGC**	SHC2	25759
**GTGGATGGTTACCACTTGGA**	SPRED1	161742
**GTGGCCAACGATGAGAACTG**	ANAPC11	51529
**GTGGCCACCCAAATACTAGA**	RSPH3	83861
**GTGGCCATAAGACGCCCCGG**	PRKAB1	5564
**GTGGCGGAACCCATCAATGT**	NPRL2	10641
**GTGGCGTAAGCTGTTCGGGC**	FUNDC2	65991
**GTGGCGTACTGCACGTGTCG**	MED12	9968
**GTGGCGTATAGCGTGCCCCC**	EIF4EBP3	8637
**GTGGCTCACCCCGTCAGTCG**	ONE_NON-GENE_SITE_290	ONE_NON-GENE_SITE_290
**GTGGCTCACTTCTAACCGAC**	ONE_NON-GENE_SITE_291	ONE_NON-GENE_SITE_291
**GTGGCTCTCAAATGTCGGAC**	ONE_NON-GENE_SITE_292	ONE_NON-GENE_SITE_292

**GTGGCTTCAGCATGGAAGTG**	NOX1	27035
**GTGGGAGAGATAAAACCTCA**	NEDD9	4739
**GTGGGAGTGCCTTCCATGAG**	VPS39	23339
**GTGGGCCAGGAGCTCATGGA**	RASGRF1	5923
**GTGGGGACGCTGCGTCGATA**	RPS13	6207
**GTGGGTTCATGATCCATCAG**	PRKAB2	5565
**GTGGTACATCAAGTCTCCCC**	PRKCG	5582
**GTGGTAGATGCTCTACGTCC**	ONE_NON-GENE_SITE_293	ONE_NON-GENE_SITE_293
**GTGGTAGTTGTAGACGCTTT**	ERF	2077
**GTGGTGATGACAGCCGGGTG**	HACD2	201562
**GTGGTGGAAGTGGTCCGCAT**	MAGEA12	4111
**GTGGTGGAGTGCCCGGAACA**	CRKL	1399
**GTGGTGGCGGCCGTTATTGG**	SAP18	10284
**GTGGTGGGCGACGGCGCGTG**	RHOB	388
**GTGGTTATGACGTGTGGTTG**	LIPK	643414
**GTGTAAATTTGATGACGACG**	USP7	7874
**GTGTAACTCAAACGCCCCAC**	ONE_NON-GENE_SITE_294	ONE_NON-GENE_SITE_294
**GTGTAATACGCTGTTTGTGG**	DCAF12L2	340578
**GTGTACAAATTCCTGCACCA**	JAK3	3718
**GTGTACATGATGCCAACCGA**	USP7	7874
**GTGTACCCAATCACGACAGG**	TCF7L2	6934
**GTGTACTGCCCCGTGACAGT**	ONE_NON-GENE_SITE_295	ONE_NON-GENE_SITE_295
**GTGTAGTAGGCCCCGCTCCC**	CDC23	8697
**GTGTAGTGGAGAATGAATAC**	FGFR2	2263
**GTGTATTTAACTGTGACAAC**	LIPK	643414
**GTGTCAACCTACGTACAATC**	ONE_NON-GENE_SITE_296	ONE_NON-GENE_SITE_296
**GTGTCCCCGAGGGATATCTG**	FANCC	2176
**GTGTCTCAGTGTGGCCTGTG**	B3GAT3	26229
**GTGTCTCAGTGTGGCCTGTG**	B3GAT3P1	402146
**GTGTCTTGTGTACGCTCCAG**	ONE_NON-GENE_SITE_297	ONE_NON-GENE_SITE_297
**GTGTGCTCAAAATCAGATGG**	PAK2	5062
**GTGTGGGTTCAGTTGACAGG**	PPP1R7	5510
**GTGTTACTATTATCACGCCC**	PSMD3	5709
**GTGTTCAATGAAATCGTGCG**	CCND1	595
**GTGTTCCTGGGGGAGCAAAG**	RAB6A	5870
**GTGTTGATGCACTACCCCCG**	PTGS1	5742
**GTGTTTACATGCGATTCATG**	PRKAG2	51422
**GTTAACACACAGCCGTCCAT**	ONE_NON-GENE_SITE_298	ONE_NON-GENE_SITE_298
**GTTAACCAGCACGTAACCAC**	ONE_NON-GENE_SITE_299	ONE_NON-GENE_SITE_299
**GTTAACTCGAAAGCAAGTGA**	PRPF38B	55119
**GTTAATCCAATGAGGAACGG**	ETS2	2114
**GTTACAATGGACTCACAAGA**	E2F7	144455
**GTTACCCGACTGCATGTCCA**	PSMD11	5717
**GTTACCTGTAGGGAATGCCG**	HDAC7	51564
**GTTACCTTGATAGCATAGGG**	INTS6	26512
**GTTAGCAGGACGTAAGGACA**	EXOC2	55770
**GTTATGACTCAGTCCAACTA**	ONE_NON-GENE_SITE_300	ONE_NON-GENE_SITE_300

**GTTCAACTCGAGCAAAACAG**	ATP2A2	488
**GTTCAATCCCTTCGTTACCT**	RPL26L1	51121
**GTTCACAATTTCCCGCCGTG**	TGIF1	7050
**GTTCACCTGCCCGTACTCGA**	TUBGCP2	10844
**GTTCACGCACTCGCGGCTCT**	GATA6	2627
**GTTCATCATTAAGGTATGAT**	TMEM263	90488
**GTTCATCTTCACAATCCGAT**	MCM7	4176
**GTTCATTAAAATCGATGGCA**	RPS4X	6191
**GTTCCAACGGGTACCAGTTT**	UBL5	59286
**GTTCGAACAGGTATCTACCA**	PIK3CA	5290
**GTTCGGGCACGCCATGTGAA**	ONE_NON-GENE_SITE_301	ONE_NON-GENE_SITE_301
**GTTCTACCTTCAGTCCGATG**	ONE_NON-GENE_SITE_302	ONE_NON-GENE_SITE_302
**GTTCTAGCTTCAGACGAATG**	ONE_NON-GENE_SITE_303	ONE_NON-GENE_SITE_303
**GTTCTCCACTCACTTGTCAC**	TFDP2	7029
**GTTCTTCGAGTCCGTCACGT**	PDPK1	5170
**GTTCTTCTCCCAGAATATGG**	HSPE1	3336
**GTTCTTCTCCCAGAATATGG**	HSPE1P3	100507046
**GTTCTTCTCCCAGAATATGG**	HSPE1P4	100287369
**GTTCTTTGAGGCTGTAAACG**	FANCC	2176
**GTTGAAGCCTCCGAACAGCT**	SRC	6714
**GTTGAAGTTGGGCTTCTCTG**	TRIM64B	642446
**GTTGAAGTTGGGCTTCTCTG**	TRIM64DP	727828
**GTTGACATCTGACGACGTGA**	RPS13	6207
**GTTGACATCTGACGACGTGA**	RPS13P2	729236
**GTTGACCCTCACATTGGTCA**	RASSF4	83937
**GTTGATAGGAACTTAACTGG**	SHC4	399694
**GTTGATGAGCCAATGGAAGA**	RPS6KA6	27330
**GTTGCACTCGATTGGGACAG**	CBLB	868
**GTTGCCCGCCAACAAAACAG**	FGFR1	2260
**GTTGCCTATCACAATATCAG**	CDC23	8697
**GTTGCGAAGGATCCCCAACG**	STK11	6794
**GTTGGTCAGGTCTTGCAGGA**	PEA15	8682
**GTTGGTTATGGTGTTAGTGA**	RAD52	5893
**GTTGTCCAAAGAAATGATGA**	CHMP3	51652
**GTTGTCCAAAGAAATGATGA**	RNF103-CHMP3	100526767
**GTTGTGGACGATCAACGGGG**	JAK1	3716
**GTTGTTGACAGGCAAATAAG**	CHMP3	51652
**GTTGTTGACAGGCAAATAAG**	RNF103-CHMP3	100526767
**GTTTAATGACCCGAGCCTCA**	RAPGEF2	9693
**GTTTACACACAGGAAAGGGC**	RGPD5	84220
**GTTTACACACAGGAAAGGGC**	RGPD6	729540
**GTTTACACACAGGAAAGGGC**	RGPD8	727851
**GTTTACCTGTGACATCCCAT**	KDM3B	51780
**GTTTATAGTAACACTCTAGC**	FBXO11	80204
**GTTTATGGAGACTATCAACG**	ONE_NON-GENE_SITE_304	ONE_NON-GENE_SITE_304
**GTTTCCAGTAAACATTGCGA**	ASXL3	80816
**GTTTCCGCATGACCTCGAGG**	DPF1	8193

**GTTTCTATCATCCAAAGTAT**	BRCA1	672
**GTTTCTCAATAAAGAGCCTA**	STAMBP	10617
**GTTTGAATTCCGTCGGACAG**	VPS45	11311
**GTTTGATGATGCCCAACAGG**	RIC8A	60626
**GTTTGCACAAGAAAACAGAC**	YPEL5	51646
**GTTTGCACAGACTCAGCTGG**	GID8	54994
**GTTTGCCGTACTTCACATCA**	VCP	7415
**GTTTGCTCACTCGCATCAGT**	MCM6	4175
**GTTTGGTCAGAAATCACATG**	SMU1	55234
**TAAAACACTTCATCGAACTG**	RASA3	22821
**TAAAAGAAGATCTCGGTCAA**	DDX46	9879
**TAAAAGCAACTGGTCTCGGA**	TSC1	7248
**TAAAAGGCCTGACATATCTG**	MAP2K1	5604
**TAAAAGGCCTGACATATCTG**	MAP2K1P1	29778
**TAAAAGGCTAAGCTTGCCCG**	ONE_NON-GENE_SITE_305	ONE_NON-GENE_SITE_305
**TAAAATTTGAACTACGACAG**	ONE_NON-GENE_SITE_306	ONE_NON-GENE_SITE_306
**TAAACTCCAACACGATCCCG**	IRF2	3660
**TAAACTTACTTGATTACTGG**	NUP37	79023
**TAAAGAGTATCAAGAACCAG**	TTK	7272
**TAAAGCAGGATTCCTCTAGG**	VCP	7415
**TAAAGCTGTCTTGAGTAACA**	BPIFB2	80341
**TAAAGTCATGATCTCACCAA**	RGL2	5863
**TAAATGCATTGAGAATCTGG**	PPP1R7	5510
**TAAATTATCTTCATGAGCGA**	PRKCI	5584
**TAACAAAGCTACGCCCATCA**	VPS18	57617
**TAACATTTCTACTTTACCAG**	SHOC2	8036
**TAAGAGATCATGTGTGCAGT**	ZFP90	146198
**TAAGAGTCTAAACGCATCCA**	ONE_NON-GENE_SITE_307	ONE_NON-GENE_SITE_307
**TAAGATACTTACAAACACAC**	RPS4X	6191
**TAAGATGAGACTATCGTAGC**	ONE_NON-GENE_SITE_308	ONE_NON-GENE_SITE_308
**TAAGCACACCCAGCGCCCCG**	IRS1	3667
**TAAGCGTATAATTTGTCAGA**	C12orf77	196415
**TAAGTCAGGGGAAACGATTG**	PDGFRA	5156
**TAATAACATCACCATGCACA**	LYN	4067
**TAATAATGCAAGCTGTGAAG**	ITFG1	81533
**TAATCAATTTGCCGTATGCA**	ONE_NON-GENE_SITE_309	ONE_NON-GENE_SITE_309
**TAATCTATAAAGAGTTAGAA**	LUZP4	51213
**TAATGACCCCAATGACTACG**	POLR2C	5432
**TAATGGAGTGGACGTGACTG**	SCRIB	23513
**TAATGGATGAAAGGATTCAG**	EXOC5	10640
**TAATGGCACAGGGTTAATGG**	RABGAP1	23637
**TAATGTAAGTTGCTTAGAAA**	CCDC6	8030
**TAATGTATGGAAAAGTGTAC**	POLR2H	5437
**TAATTAATCAGGCTTCACAA**	CREBBP	1387
**TAATTGGTTTCTGTACCCAC**	PHB	5245
**TACAACAGATCGTGTAATGA**	HDAC2	3066
**TACAACGCCCCTACAGACCA**	GRB10	2887

**TACAAGGAAGAATGGATACC**	POLR3G	10622
**TACACATTTAGGCTGACACT**	FGFR2	2263
**TACACCCCAGTCTACCGCAG**	ROS1	6098
**TACACCTTGTAGTCCTCCGT**	TRAPPC2L	51693
**TACACGAGGAGAACAGGTTA**	CDC6	990
**TACACGTATCGTATTTGCCA**	ONE_NON-GENE_SITE_310	ONE_NON-GENE_SITE_310
**TACAGAAATGTTAGCAACTG**	LOC246784	246784
**TACAGAAATGTTAGCAACTG**	SMU1	55234
**TACAGACAACGCCTCTGACA**	FGFR4	2264
**TACAGCCAGTCGCTGAAGCG**	KSR1	8844
**TACAGGCATCTCGTGCACGG**	C9orf116	138162
**TACAGGGAACCTCGAGTGGT**	VPS25	84313
**TACAGTCACTGACTGAGCCT**	ERF	2077
**TACATAGATGGAGCTTTGTC**	SNRPF	6636
**TACATTAGGATGATAAATTT**	LOC100507422	100507422
**TACATTAGGATGATAAATTT**	UBE2N	7334
**TACATTAGGATGATAAATTT**	UBE2NP1	100288613
**TACCACAATGACATTAACCT**	GPRC6A	222545
**TACCACAGTCATACCTCACG**	SMU1	55234
**TACCACGTCCAGCCGATGAG**	AGPAT3	56894
**TACCACTTTGAGCTGAGCAA**	PFDN5	5204
**TACCATAAGTGATCAACAGA**	FLVCR1	28982
**TACCATGATCTACAGGAACT**	MDM2	4193
**TACCCACTGGGAGCAAACAA**	C12orf77	196415
**TACCGATAGCCAATCATGAT**	ONE_NON-GENE_SITE_311	ONE_NON-GENE_SITE_311
**TACCTAACTGGAGTGTGTGG**	RB1	5925
**TACCTATGATGGAATCCCTC**	RASA3	22821
**TACCTGCATAGGAAGACACG**	IRF2	3660
**TACGAATGCGTGGCGGAGAT**	CDK6	1021
**TACGACAGAGATGAATTGAG**	CRYGB	1419
**TACGCCATCCCGGGCGACTG**	STK11	6794
**TACGTGCCCACCACCCGCGT**	GATA6	2627
**TACTAACCTGTGCCCTTGGG**	PEBP1	5037
**TACTCACATTAGATGCATCC**	EMC3	55831
**TACTCCCTGAAGCAACCTGA**	CHURC1-FNTB	100529261
**TACTCCCTGAAGCAACCTGA**	FNTB	2342
**TACTGTACCAGACAAACTCG**	PRKCQ	5588
**TACTTACCAGCCATCAACAC**	PRKCB	5579
**TACTTGTGATTCCACGAACA**	MON2	23041
**TACTTTGCAACTTCATATCC**	UGCG	7357
**TAGAAGTCTTACGCCAAGAC**	ONE_NON-GENE_SITE_312	ONE_NON-GENE_SITE_312
**TAGACATTGACCCACCCCTG**	INSR	3643
**TAGACCAAGAAGGGAACCGA**	RASGRP2	10235
**TAGACCAGAACCCTACTAGC**	ONE_NON-GENE_SITE_313	ONE_NON-GENE_SITE_313
**TAGACGGCGAAATCTTGTCG**	DHX8	1659
**TAGACGTTGTTCTCACACAG**	NPRL3	8131
**TAGACTAGACCGTACTGAAG**	ONE_NON-GENE_SITE_314	ONE_NON-GENE_SITE_314

**TAGACTGTGCTGACTGAGCA**	LONRF1	91694
**TAGAGATCTTCAGGAAGATC**	RPS13	6207
**TAGAGATCTTCAGGAAGATC**	RPS13P2	729236
**TAGAGTTATAGATGGCCAGT**	JAK2	3717
**TAGATACTATAAATCACCTA**	PRKAG2	51422
**TAGATATTCTGGACACCGCT**	RALB	5899
**TAGATGGCGTCTGATACCAC**	NFKB1	4790
**TAGATTGGTCTCGCAATGCA**	DUSP6	1848
**TAGCAAAATGCGTCTAGAGC**	LOC110117498-PIK3R3	110117499
**TAGCAAAATGCGTCTAGAGC**	PIK3R3	8503
**TAGCAAGCATGATCTTTCGA**	CSNK2A2	1459
**TAGCAGCAAAGGGCGCGTAG**	RAPGEF1	2889
**TAGCATCGAGAGTAAACATG**	UBE2H	7328
**TAGCGATCATCACACAGTGC**	RIC8A	60626
**TAGCGCGGCGAGTACACGAT**	HDAC11	79885
**TAGCTGCGACTGATAGATTG**	CSNK2A2	1459
**TAGGAACTCTGACCCCAAAG**	RASA1	5921
**TAGGACTCTGTGATGAAGCT**	ETS1	2113
**TAGGAGAAGTTCTTGCAGCT**	TFDP2	7029
**TAGGCAAAATGACGCTCACT**	ONE_NON-GENE_SITE_315	ONE_NON-GENE_SITE_315
**TAGGCGCTTACAGTCATCAT**	LOC653406	653406
**TAGGCGCTTACAGTCATCAT**	LOC728519	728519
**TAGGCGCTTACAGTCATCAT**	NAIP	4671
**TAGGTGGCGACAGTGCTATG**	PHAX	51808
**TAGGTTCAGAGCTTCATGCG**	RASGRF2	5924
**TAGTAGGTAAATCCGATGAA**	ROCK2	9475
**TAGTAGTCGCTGGTCCTCTC**	C9orf116	138162
**TAGTCGTGTTAATGTCCACG**	ONE_NON-GENE_SITE_316	ONE_NON-GENE_SITE_316
**TAGTGATTCATTGAGAACTG**	LPCAT3	10162
**TAGTGGACCTTACCCATACC**	NF1	4763
**TAGTGTCAATACCTAGCTCC**	INIP	58493
**TAGTGTTTACAGCCACGATC**	ONE_NON-GENE_SITE_317	ONE_NON-GENE_SITE_317
**TAGTTATACGATTAAAGCGA**	SHOC2	8036
**TAGTTTACGATATCACAAGT**	RAB6A	5870
**TATAAACAGGCTCATGACTC**	WDR70	55100
**TATAATTCATATACGGTACG**	ONE_NON-GENE_SITE_318	ONE_NON-GENE_SITE_318
**TATAATTGGTGATACGCACA**	ONE_NON-GENE_SITE_319	ONE_NON-GENE_SITE_319
**TATACAGACAGTATTATAAA**	LOC653406	653406
**TATACAGACAGTATTATAAA**	LOC728519	728519
**TATACAGACAGTATTATAAA**	NAIP	4671
**TATACTTACAGGAAATAGTG**	POLR3G	10622
**TATAGGATTCGGAAGGACGT**	VPS39	23339
**TATAGGGGAACCCATACGTA**	ONE_NON-GENE_SITE_320	ONE_NON-GENE_SITE_320
**TATATACTACAGTTATTACT**	VKORC1L1	154807
**TATATAGTGTCATCGAACCC**	ONE_NON-GENE_SITE_321	ONE_NON-GENE_SITE_321
**TATATATGACCCCACCTACA**	SCD	6319
**TATATGACGTCCTCACGCCG**	ONE_NON-GENE_SITE_322	ONE_NON-GENE_SITE_322

**TATATTGAAATGGATCCTCC**	PIK3R3	8503
**TATCAATAGCCGAGAAACCA**	PRKCQ	5588
**TATCACCAACAATGAGAGAG**	CSNK2A2	1459
**TATCAGACACCATATACCCG**	XIAP	331
**TATCCAAACATTATTGCTAT**	PTEN	5728
**TATCCGTGGAGAGCGACTCA**	SPRED1	161742
**TATCGTCGTTGCTTGCTACC**	ONE_NON-GENE_SITE_323	ONE_NON-GENE_SITE_323
**TATCTCGAACAACTACACTG**	KPNB1	3837
**TATCTTCCCAGAGTTACTGG**	CAMK2D	817
**TATCTTCTTCAATTCCAACA**	VTA1	51534
**TATGAATTAAACAATCGTGT**	SMAD5	4090
**TATGACAGAAGGGTAATTCG**	RSPH3	83861
**TATGACGATCCACATCGCAT**	ONE_NON-GENE_SITE_324	ONE_NON-GENE_SITE_324
**TATGCCACTCTCTTACCAGG**	PAK2	5062
**TATGCCCTGTACTTACAGTG**	HDAC5	10014
**TATGGTAGCTACCCTTAGCC**	HACD2	201562
**TATGGTGCGTTCTACAGCGA**	MAP2K1	5604
**TATGTGGTGAACAAAGCTGG**	TRAPPC4	51399
**TATGTTTACAACATCATCGG**	PSMB1	5689
**TATTAAAAGGATTTGAGCTG**	CHD2	1106
**TATTCGATGTCAAGCGAACG**	PRKCH	5583
**TATTGAGCGATTTCGCAGAG**	LOC110117498-PIK3R3	110117499
**TATTGAGCGATTTCGCAGAG**	PIK3R3	8503
**TATTGATGCACAGGTATACA**	SPTLC2	9517
**TATTGATGGCAAATACACAG**	NRAS	4893
**TATTGCGAACCGAATGTACG**	ONE_NON-GENE_SITE_325	ONE_NON-GENE_SITE_325
**TATTTAATGGAATCTCTAAG**	RASGRP3	25780
**TATTTACATGTAACGCTACA**	MYB	4602
**TATTTGAAACTACTAGGTAA**	AKT3	10000
**TATTTGACTTCAGTCAGCGA**	NFE2L2	4780
**TCAAAAAGATGATCATACGG**	TSC1	7248
**TCAAACTTGCAGTACAACGC**	ONE_NON-GENE_SITE_326	ONE_NON-GENE_SITE_326
**TCAAAGAGTCCATCAAACAC**	HDAC2	3066
**TCAAATACCAGAAGGCCACA**	RASGRP4	115727
**TCAACAATCAATGTCTACCG**	ONE_NON-GENE_SITE_327	ONE_NON-GENE_SITE_327
**TCAACACCAACATCGATGGG**	RPS18	6222
**TCAACACCAACATCGATGGG**	RPS18P12	388339
**TCAACACCAACATCGATGGG**	RPS18P13	100271364
**TCAACAGGCAATGAACACCA**	PRKCI	5584
**TCAACATCAATACAAAACAC**	SNX14	57231
**TCAACCGTGATTGAAAATCC**	FRK	2444
**TCAAGGCACAATATCAGCAG**	KPNB1	3837
**TCAAGGCCAGGAGGGGCAGC**	UBE2Q2L	100505679
**TCAAGTGCTCGCAGAAGCAT**	FLT3	2322
**TCAATAGGCGTGTAATGATG**	APC	324
**TCAATGTAGGAGAGGTTGTC**	PEA15	8682
**TCAATTAACAGGCCATAAAG**	PRKAA2	5563

**TCAATTTCACAGTAAGTGGC**	TM9SF3	56889
**TCACAAACCCGTCGTCCATG**	FNTA	2339
**TCACAATGATCTCGTTGACA**	MCM4	4173
**TCACACCCTTACGAGGCAAG**	ONE_NON-GENE_SITE_328	ONE_NON-GENE_SITE_328
**TCACACGTACAATCCTGGTA**	ONE_NON-GENE_SITE_329	ONE_NON-GENE_SITE_329
**TCACACTTGAAAATATCTTT**	ICMT	23463
**TCACATCTAGTGGTATCCTG**	RAC1	5879
**TCACATCTCGAACCATGTGG**	YPEL5	51646
**TCACCACTTAGAACCCAACA**	RASGRP3	25780
**TCACCAGACGGGAGTCAGAG**	SRC	6714
**TCACCAGCAGGTGCGAGCAG**	PIN1	5300
**TCACCAGGGATAGCCCCATA**	ONE_NON-GENE_SITE_330	ONE_NON-GENE_SITE_330
**TCACCCACCTACAATGACAC**	SMC1A	8243
**TCACCTCTCATTGTCCGCGG**	DAB2IP	153090
**TCACCTTGATGAGGGGATCG**	RPS4X	6191
**TCACGCTCCCTTATGGAACA**	FERMT1	55612
**TCACGTACCTATCGGCAAAG**	TUBGCP2	10844
**TCACGTCATAGTCTTCAGTG**	LPCAT3	10162
**TCACGTCCATAGAACAGGTG**	FNTB	2342
**TCACGTTGGTCCACATCCTG**	AKT1	207
**TCACTGAAATATTAGAAGAT**	NUDT4	11163
**TCACTGAAATATTAGAAGAT**	NUDT4B	440672
**TCACTGAAATATTAGAAGAT**	NUDT4P2	170688
**TCAGAAAACATGGATGATAC**	ATXN3L	92552
**TCAGAAAACATGGATGATAC**	GS1-600G8.3	100093698
**TCAGAAACAACAGCATATAG**	YES1	7525
**TCAGAAACAACAGCATATAG**	YES1P1	7526
**TCAGAAACTAGTGTGAACAG**	RPS6KB1	6198
**TCAGAACAGACATACATGAA**	CCNA1	8900
**TCAGAATAGGCTCCATGTAG**	MTOR	2475
**TCAGAATCCATCTACCGTAG**	PRKCI	5584
**TCAGACCACCAGTTAACTAC**	ONE_NON-GENE_SITE_331	ONE_NON-GENE_SITE_331
**TCAGACCAGCGGATAACAGT**	PRKAB2	5565
**TCAGACCCACTAAAGCAATC**	TSPAN13	27075
**TCAGACCCCATCGAACACTT**	ONE_NON-GENE_SITE_332	ONE_NON-GENE_SITE_332
**TCAGATCCGCTACCTCACCC**	MED11	400569
**TCAGCACACCATCACAACCA**	PREX2	80243
**TCAGCAGGAGGGTTTATCCC**	RASSF5	83593
**TCAGCTACTCAAGGCAACTT**	ONE_NON-GENE_SITE_333	ONE_NON-GENE_SITE_333
**TCAGCTTATTGGCTACAGCA**	PIK3R5	23533
**TCAGCTTTGAACTTGGACAG**	UBAP1	51271
**TCAGGAAATGATCCGCACAG**	MTOR	2475
**TCAGGCCCCAGCTTAACCGT**	ONE_NON-GENE_SITE_334	ONE_NON-GENE_SITE_334
**TCAGGGAAAGAGTATGCCAA**	EBP	10682
**TCAGGGCAGAGATTTGACGT**	SMC1A	8243
**TCAGGTAAAGCTATTGACTC**	ONE_NON-GENE_SITE_335	ONE_NON-GENE_SITE_335
**TCAGGTGTCTCCCACTCCAC**	RASSF1	11186

**TCAGTACAAGTGCATCCCAG**	DUSP4	1846
**TCAGTGACACCATACCACCT**	ONE_NON-GENE_SITE_336	ONE_NON-GENE_SITE_336
**TCAGTTGACAAGGGATAACT**	E2F7	144455
**TCATAATTAACACACATCAG**	BRAF	673
**TCATACAGAAATCTATTCTA**	CCT4	10575
**TCATACCACAAACCATAGAT**	TSC1	7248
**TCATACCTATAGTATCTGGG**	SHOC2	8036
**TCATACTGGTGACAATTCAC**	ATP2A2	488
**TCATAGCGAATCACCACATG**	SHC3	53358
**TCATAGGATTGGCTCCAGAG**	MAGEA3	4102
**TCATAGGATTGGCTCCAGAG**	MAGEA6	4105
**TCATATCCTTAAACAAATGG**	RASSF8	11228
**TCATCACTATGGCAGAACAT**	SNX14	57231
**TCATCAGACACTACATCAGG**	RALBP1	10928
**TCATCAGGTGGCATATAGGC**	SMAD5	4090
**TCATCATAGTCTGAATGATG**	INPP4B	8821
**TCATCATCTCATCCAGACAT**	RASA2	5922
**TCATCCAAAAAGTCTGAGCG**	NSD2	7468
**TCATCGCTCACAACCAAGTG**	ERBB2	2064
**TCATCTACACGTTAAAAGAG**	TUBGCP2	10844
**TCATCTGGATTATAGACCAG**	PTEN	5728
**TCATGCTAGCAATGACTACG**	SPRY3	10251
**TCATGGAATACCTTGCTGGG**	PAK2	5062
**TCATGGAGTTTGAGCGACGA**	ONE_NON-GENE_SITE_337	ONE_NON-GENE_SITE_337
**TCATGGCAGATCCGGCAGAA**	MARCH2	51257
**TCATGGCCCTAATTTCGACG**	ONE_NON-GENE_SITE_338	ONE_NON-GENE_SITE_338
**TCATGTCTGTCATTCGAGAG**	ATP2A2	488
**TCATGTGCACGATCTCCAAG**	LOC100420464	100420464
**TCATGTGCACGATCTCCAAG**	SAV1	60485
**TCATTGTAGATCGAGCGAAG**	CECR2	27443
**TCCAAAAACTCTTCAGCATG**	BUB1	699
**TCCAAAAGGCGTTACCCCTG**	YES1	7525
**TCCAAAAGTTATGTCTTCCG**	SEC23B	10483
**TCCAAACATTATCACTCTAA**	RPS6KA3	6197
**TCCAAAGTGAAGGATATGGC**	SPNS1	83985
**TCCAACGTTTCATCACTGGA**	SPRED2	200734
**TCCAACTGCTCGGCACTCAG**	UNG	7374
**TCCAAGACCGTTCAGACCCT**	RASA3	22821
**TCCAATCAGATCCACAACTG**	CNKSR2	22866
**TCCACAGAATGACCCGGACA**	EXOC7	23265
**TCCACATCATGCAGCTTCCC**	PFDN5	5204
**TCCACCTTCGGCATACACCA**	INSRR	3645
**TCCACGTAGTTCAACGTCCA**	ONE_NON-GENE_SITE_339	ONE_NON-GENE_SITE_339
**TCCACGTTATGATTTAGACG**	TBK1	29110
**TCCACTTCTTGCAGGCTCAA**	PEBP1	5037
**TCCAGACCCGGCCCTAGACG**	PIK3CG	5294
**TCCAGATACGCTTACTGACA**	UGCG	7357

**TCCAGCACACGACACCACCT**	CREBBP	1387
**TCCAGCACTTCATCCCAGTG**	RASAL1	8437
**TCCAGCCACAGCTCTTCGTA**	GAREM1	64762
**TCCAGGGCTACCCTCTGATG**	ELOVL1	64834
**TCCATACTCCTCAAGGACTT**	SPTLC2	9517
**TCCATCACCAGAATGGGACA**	CAMK2D	817
**TCCATCATCCGAACCCAACC**	SPRY3	10251
**TCCATGCTTTCCGTGTGGTG**	CRKL	1399
**TCCATTGTGTCTGCAGCCCG**	COX4I2	84701
**TCCCACAGGTCGCTTTGACA**	VCP	7415
**TCCCACATCCACCATCAAGC**	HCK	3055
**TCCCAGCTGTCTCCTGAACA**	TRAPPC4	51399
**TCCCAGGCTCCATCGCGCGG**	SLBP	7884
**TCCCATCAAAACTCATGGCG**	UAP1	6675
**TCCCCAGGAAGCATACGTGA**	ERBB2	2064
**TCCCCAGGTTCACGCAAACT**	ONE_NON-GENE_SITE_340	ONE_NON-GENE_SITE_340
**TCCCGAACATAGGCTATCTG**	ITCH	83737
**TCCCGACAGTACAGGATCTG**	ARID5B	84159
**TCCCTACTTGGTGTCCGGCG**	SHC3	53358
**TCCCTGCACTCCAGGCACTG**	PEX16	9409
**TCCCTGGACAGCAGCAACAG**	DDIT4	54541
**TCCCTTCTCAGGATTCCTAC**	KRAS	3845
**TCCGACGGCCAGCTCCACCA**	MAP2K2	5605
**TCCGACGGCGAGGTCCGACG**	DNAJC9	23234
**TCCGCTCGCGCCTTACTCGG**	FOSL1	8061
**TCCGCTGGTCGGCCACAAAG**	SPNS1	83985
**TCCGGAGCTACCTTCGTCCA**	SHC4	399694
**TCCGGTGATTCATACAAATG**	GAREM1	64762
**TCCTAAAGATAACTCGACAC**	SLC11A2	4891
**TCCTAGCAGTCTCTGTTCAG**	SPRY2	10253
**TCCTAGGAGAGTGCGTAGAA**	GAREM1	64762
**TCCTCAAAGACTGGGCTGTC**	SREBF1	6720
**TCCTCAGAAAGGAACCACCG**	RALGAPA2	57186
**TCCTCCCCCAAACAGGTCCC**	PRKAG3	53632
**TCCTCCTCAGAACACGCCGA**	SRC	6714
**TCCTGAGAAGTGGACCCCAG**	RHOC	389
**TCCTGCCAACATCAGCCGAG**	PLXNB1	5364
**TCCTGCTCGTCGACCTCGGG**	CCDC6	8030
**TCCTGGACTTCACGGGATGG**	ELK1	2002
**TCCTGGCAGAGACTTGGGGA**	PSMB1	5689
**TCCTGGGGAGAATTTGTGCA**	HDAC8	55869
**TCCTGGTACGACTCCCCTTG**	TIAM2	26230
**TCCTGTCATTCGAGTCAGGT**	SREBF2	6721
**TCCTTACCAATGCACACAGA**	CNKSR2	22866
**TCCTTCACCAAGATTTACCG**	JAK3	3718
**TCCTTCCCAAGAGACTGAGC**	RIC8A	60626
**TCCTTGCGATGTACTCGTCG**	TSC2	7249

**TCGAATTACATCTTTACAAG**	ROCK1	6093
**TCGAGCCACACTGTCTGAAG**	RASSF8	11228
**TCGAGCGGCTGTAGCGAGAT**	SRSF2	6427
**TCGATATCTTAGATACAGCT**	RALA	5898
**TCGCACCCGCCGCGGTACCG**	SLC25A1	6576
**TCGGCGTTGCATATCCTAAG**	UAP1	6675
**TCGGGCCCGAACACGTACTG**	ZFPM1	161882
**TCGGGCCTCAGACTGTACAG**	FNTB	2342
**TCGGGCCTCAGACTGTACAG**	LOC107984655	107984655
**TCGGGTCCCTTATCCGCACC**	PRKCA	5578
**TCGGGTTACCACTTCGTTGA**	RPL31	6160
**TCGGGTTACCACTTCGTTGA**	RPL31P4	729646
**TCGGGTTACCACTTCGTTGA**	RPL31P49	100129882
**TCGTACAAGTTGTCGGCCAG**	SIRT1	23411
**TCGTAGTACTCGAGCCGCGG**	IRS2	8660
**TCGTAGTGTATTACGATGCT**	ONE_NON-GENE_SITE_341	ONE_NON-GENE_SITE_341
**TCGTCCTGAGAGAAACACAT**	MCM6	4175
**TCGTTACTCGAAAAATTAGC**	GMDS	2762
**TCGTTTGCGATTGCTGTTAG**	ECT2	1894
**TCTAAAATGAAGAGGTCCAA**	RALBP1	10928
**TCTAAATATAGCACCCAGCA**	SLC30A1	7779
**TCTAAGGACAAAATAGCAGT**	SPRY1	10252
**TCTAAGTCAATCATGTCCCG**	ATP6V0A1	535
**TCTACACCGACAACTCCATC**	CCND1	595
**TCTACAGATTATTCAGGACA**	TYMS	7298
**TCTACAGTTCATGTACGATG**	RALA	5898
**TCTACGCCTACCACGTGGAG**	VKORC1L1	154807
**TCTACTATATGTGCACGTAG**	WDR70	55100
**TCTACTGTGCAGTCATCGTG**	CASP8	841
**TCTATACATGCAACGACTTG**	MCM4	4173
**TCTATAGATTTCTTCGAACT**	SUZ12	23512
**TCTATGATGCAGTAATCACT**	RASSF9	9182
**TCTCCCTTGGACACCCCAAT**	ONE_NON-GENE_SITE_342	ONE_NON-GENE_SITE_342
**TCTCGTCAGCCGACAGACAT**	ONE_NON-GENE_SITE_343	ONE_NON-GENE_SITE_343
**TCTGACAAGTCGCCATCCCT**	ONE_NON-GENE_SITE_344	ONE_NON-GENE_SITE_344
**TCTGACATTTAATTCCGTGG**	LOC110117498-PIK3R3	110117499
**TCTGACATTTAATTCCGTGG**	PIK3R3	8503
**TCTGACCAGGCACGATCACA**	XIAP	331
**TCTGACCTTAAGGTCGCAGG**	MAPK3	5595
**TCTGACTGGCCATCTACTTG**	MON2	23041
**TCTGACTTCGACAACGCCAA**	SRC	6714
**TCTGATACTCGATTGAGTGA**	PSMB1	5689
**TCTGATCTTGTAGACAGCTG**	PFDN5	5204
**TCTGATGATAAATGACTGCG**	PTK2	5747
**TCTGATGTCAAGCGAAAGGT**	CERS2	29956
**TCTGCATAATATGGGCGCAT**	ONE_NON-GENE_SITE_345	ONE_NON-GENE_SITE_345
**TCTGCATGGGTTGCTATAAG**	INPP4B	8821

**TCTGCATTACTGCCCTCGGA**	SLC50A1	55974
**TCTGCATTTCTCCTAATGCC**	PRKAG1	5571
**TCTGCCAAGCAAATCCACAT**	UBAP2L	9898
**TCTGCCAGAGGTGCAACTAA**	VPS39	23339
**TCTGCCCGGGAGCTAAAACG**	MTAP	4507
**TCTGCGTGCTCTTCCCGGCG**	DTYMK	1841
**TCTGCTGAGAAACATCGAAG**	PPP1R7	5510
**TCTGCTTATTAACACAGAGG**	CDK2	1017
**TCTGCTTTAGAGTACTTGCA**	PDPK1	5170
**TCTGGATAGCAATGCGCACC**	NPRL2	10641
**TCTGGATCAGGTTTCCCACA**	TIAM2	26230
**TCTGGATCAGTATCAGACGG**	RASGRP2	10235
**TCTGGCCCACAGAAGAACCT**	ARAF	369
**TCTGGGACTCAGGACCCACG**	BPIFB2	80341
**TCTGGGATCTTGTACTGGCG**	PCNP	57092
**TCTGGGATCTTGTACTGGCG**	RPS18	6222
**TCTGGGATCTTGTACTGGCG**	RPS18P5	100131863
**TCTGGTACCACGGGTCTACG**	PIK3R6	146850
**TCTGGTTAGGTTTGTAAGTG**	ESRP1	54845
**TCTGGTTCGGCTTTGATGCC**	UBE2N	7334
**TCTGTAGGTTCCTCCTTGGG**	NO_SITE_4	NO_SITE_4
**TCTGTCCAGTGGAACGATAG**	PEX16	9409
**TCTGTCTCACATGCACCCCG**	TSC2	7249
**TCTGTGGACTGGACAATGAG**	SAV1	60485
**TCTGTGTGATGGACAAAGAC**	RASGRP1	10125
**TCTGTTGTGTACGATGCAAG**	TMEM220	388335
**TCTTAAATATGGCATATCAA**	CD1E	913
**TCTTACATGAGGAAGAAGGC**	TMEM220	388335
**TCTTACCTGGAGTCAAACTG**	GPRC6A	222545
**TCTTACCTTAGTCACAATGG**	SLC22A25	387601
**TCTTAGATTGGAATAATTGG**	MTAP	4507
**TCTTAGCGTCCACACAAGCT**	ARHGAP35	2909
**TCTTCAAATTCGGCAGACGA**	FERMT1	55612
**TCTTCACCTGGTCAATGGGT**	SPRY4	81848
**TCTTCAGGAGAGAATACCAT**	JAK2	3717
**TCTTCAGGGTACACTTCCCG**	FAM20B	9917
**TCTTCCACCTCCCTAACGTG**	ONE_NON-GENE_SITE_346	ONE_NON-GENE_SITE_346
**TCTTCCATGTCAGTGCTGAG**	CCNA2	890
**TCTTCGTCAAGGGCGCCGTG**	TRAPPC5	126003
**TCTTCTAGTTGATCATACCA**	PTPN11	5781
**TCTTGCAACTTGAGAAAATT**	UBTFL1	642623
**TCTTGCCGGAATGTCAGCCG**	EGFR	1956
**TCTTGGTAGATGTTGAGCAT**	RASGRF1	5923
**TCTTGTCATCAATCTTCCAG**	SPRED1	161742
**TCTTGTCGATGCATTTCTTG**	PRKCD	5580
**TCTTGTTCTACTATTCACAC**	FAXDC2	10826
**TCTTTACAACAGAAATCACC**	PRKAA1	5562

**TCTTTAGGATCTTAAGTACC**	SMU1	55234
**TGAAAAGATCCTGCGATGGT**	ONE_NON-GENE_SITE_347	ONE_NON-GENE_SITE_347
**TGAAAAGCTTTCTTTCGGGA**	FAM217B	63939
**TGAAAGACAAGTCGTCCGGG**	LYN	4067
**TGAACACGAGACCTACACTC**	ONE_NON-GENE_SITE_348	ONE_NON-GENE_SITE_348
**TGAACACTTACTGGATGATG**	RASA2	5922
**TGAACCGTCGCTCCAATGAG**	COX4I2	84701
**TGAACCTAGTAACAGCCATA**	SUZ12	23512
**TGAACCTGAAAACTCACCGA**	AURKA	6790
**TGAACCTGGCCGACCCAGTG**	JUN	3725
**TGAACGAGAAGAACGTGTGA**	EXOSC9	5393
**TGAACGCGTGTACGTCATGG**	GAREM1	64762
**TGAACTACTTACGAACTGCT**	RB1	5925
**TGAACTGAATCATGCATCAC**	SPTLC2	9517
**TGAACTGTGTTCCTTGACCG**	HDAC6	10013
**TGAAGAAGAGAGCTCCGATG**	PDGFRA	5156
**TGAAGACATCCTAACCATGG**	SLC22A25	387601
**TGAAGACCCGTAGCAACAGT**	SAP18	10284
**TGAAGAGATTAGTCGTTCTG**	SMC1A	8243
**TGAAGCACAGCCAGTCACGG**	PIN1	5300
**TGAAGCATGGACTTTGGATG**	ATL2	64225
**TGAAGCGCAGGAGCCCCTCC**	AUP1	550
**TGAAGGGGCCACTACCGACA**	TIAM1	7074
**TGAAGGTCTGAGCATGCTTG**	CCND2	894
**TGAAGTACCAGACTACCATG**	CCNA2	890
**TGAAGTCACAACTTGCCAGG**	RASSF8	11228
**TGAAGTCTGTGTAGCACACA**	BPTF	2186
**TGAAGTTACCATGAGAATCA**	NOX1	27035
**TGAATAAGTCCATCAGACAG**	JAK1	3716
**TGAATATATCTCCAGATGCA**	PRKAG2	51422
**TGAATATGATCCCACCATAG**	NRAS	4893
**TGAATGACCGTTGCACTGAA**	SREBF2	6721
**TGAATTAGATCCAGGCGAGG**	CBLB	868
**TGAATTTGTATGACCAAACA**	APAF1	317
**TGACACAAATGGGCATGTGG**	EXOSC9	5393
**TGACACATTTCCTATATCAG**	ROS1	6098
**TGACACCAGTCAAAAACAGT**	INPP4B	8821
**TGACATAATGCAGTAAAATC**	RB1	5925
**TGACATAGATGAAGACGCAT**	METTL5	29081
**TGACATAGTTTACTAGCGGC**	ONE_NON-GENE_SITE_349	ONE_NON-GENE_SITE_349
**TGACATGACCCAGTAACGAG**	TSC1	7248
**TGACATGATCCGCCTATGCT**	ONE_NON-GENE_SITE_350	ONE_NON-GENE_SITE_350
**TGACCAAACGTATCCCCCTG**	FGFR2	2263
**TGACCATATAAAAGTTACAC**	CBLB	868
**TGACCATTCTAGTGCTCTCC**	VPS45	11311
**TGACCCCGGGAATCTGAGGG**	ANKHD1-EIF4EBP3	404734
**TGACCCCGGGAATCTGAGGG**	EIF4EBP3	8637

**TGACCTCGAGCCAATACTAA**	ONE_NON-GENE_SITE_351	ONE_NON-GENE_SITE_351
**TGACCTGCAGACTCATGGCT**	CD1E	913
**TGACCTGGCTGCACGCACTG**	HRAS	3265
**TGACCTTCATCATGACGCAT**	ONE_NON-GENE_SITE_352	ONE_NON-GENE_SITE_352
**TGACCTTCGGGACCAACACG**	RASAL1	8437
**TGACGCGCAGGATAAGCTGG**	MRAS	22808
**TGACGCGGAGGCGTATTCGG**	JAK3	3718
**TGACGTGGAGTGCACTATGG**	PRKCB	5579
**TGACGTTGCACTGGGACAGG**	PAK2	5062
**TGACTCCTGGGATATTGGGC**	EIF4EBP2	1979
**TGACTGCCAGAAGCAAGTCA**	EXOC1	55763
**TGAGAAAACACATAAAGGAG**	ZFP90	146198
**TGAGAAAATGTTTCGAATGG**	SUZ12	23512
**TGAGAAGCATTACCTTGATG**	CDK2	1017
**TGAGAAGGTGACTTACGGTG**	KDM3B	51780
**TGAGAGAACGAAGTACACGT**	ELOVL1	64834
**TGAGAGTGAATCTTTCAAGA**	POLR2H	5437
**TGAGAGTTCCACAGACAGCT**	RASSF4	83937
**TGAGATACTCCGTCTACGAG**	RASAL2	9462
**TGAGATCAAAGTATTTGGAA**	MET	4233
**TGAGATCATGACCTTAAATG**	TIAM2	26230
**TGAGCAAGAACAATCTGTAC**	ATL2	64225
**TGAGCACATACTCTTCACAC**	YAE1D1	57002
**TGAGCACCTCTTCATCTGTG**	SPRY3	10251
**TGAGCCAGCTGAGTTTCGAT**	JAK1	3716
**TGAGCCCAGTGAAGTGCGCG**	BSCL2	26580
**TGAGCCCAGTGAAGTGCGCG**	HNRNPUL2-BSCL2	100534595
**TGAGCCCTTCAAGATTCCTG**	CYTH2	9266
**TGAGCGCGGCGGCCGATCTG**	DDIT4	54541
**TGAGCTCGGCACAAGTCGGG**	ONE_NON-GENE_SITE_353	ONE_NON-GENE_SITE_353
**TGAGCTGGTAGATAAGTCGG**	CHD2	1106
**TGAGGAAAGCGGAGATGCGG**	UNG	7374
**TGAGGAAGGCCATGTAGCCT**	CBLC	23624
**TGAGGACGCCTAGTGACCAG**	RASSF2	9770
**TGAGGACGCTATGGATGCCA**	MFSD11	79157
**TGAGGACGCTATGGATGCCA**	SRSF2	6427
**TGAGGACTGGAGATAACAGA**	BARD1	580
**TGAGGAGAGCAGGCTTTACG**	TIAM1	7074
**TGAGGATGACACCGATGGTG**	FGFR2	2263
**TGAGGCACTGACTGAGACAT**	EXOC6	54536
**TGAGGGCCTGCACCAGATTG**	CYTH2	9266
**TGAGGTAGACGAATGTCACA**	ALK	238
**TGAGGTCAGACATGCGACGA**	ONE_NON-GENE_SITE_354	ONE_NON-GENE_SITE_354
**TGAGGTGCACTAATAGAGGG**	ROS1	6098
**TGAGTCACGAGAACACGTTT**	AURKA	6790
**TGAGTCATGCGGATTCGGTG**	HDAC1	3065
**TGAGTTAACAAATATTGACA**	LOC100131950	100131950

**TGAGTTAACAAATATTGACA**	METTL5	29081
**TGATACAGTGATCAATAATA**	FBXO11	80204
**TGATAGAGTCAAATTGACGG**	ONE_NON-GENE_SITE_355	ONE_NON-GENE_SITE_355
**TGATAGTGAAGTGTTGTTCG**	APAF1	317
**TGATATCTGTTCACTACGGT**	ONE_NON-GENE_SITE_356	ONE_NON-GENE_SITE_356
**TGATATGATCCTATTGACAC**	ONE_NON-GENE_SITE_357	ONE_NON-GENE_SITE_357
**TGATATTTGTTCCACCACGA**	CSNK2A2	1459
**TGATCACGGTGGACCTTGGA**	IFITM1	8519
**TGATCAGGCTAACGGCGACG**	DPM2	8818
**TGATCAGTGACAACCAATAC**	MCM3	4172
**TGATCGAGCTGATCCGCCAG**	GID8	54994
**TGATGAAATTCCGGGACGAA**	MCM5	4174
**TGATGAACTATGGGTAGTCA**	PAK3	5063
**TGATGAATCTTGGGTCATTG**	BUB1	699
**TGATGATCGCGTACTGAGTG**	DHX8	1659
**TGATGCAGGAAAGAAGAAGT**	IFIT5	24138
**TGATGCTTTAAATGTGCTAA**	TFDP2	7029
**TGATGGAGAAACCTGTCTCT**	KRAS	3845
**TGATGGAGAAACCTGTCTCT**	KRASP1	3844
**TGATGGCTGAGTTGGCTGAG**	VTA1	51534
**TGATGTAAATCACCCATTCG**	RPS6KA1	6195
**TGATTATGAGCAACCACTGG**	CDC25A	993
**TGATTATGGGTAAGAAGACC**	DHFR	1719
**TGATTATGGGTAAGAAGACC**	DHFRP1	573971
**TGATTCCAGCACATTAATGG**	EZH2	2146
**TGATTGCGATCGCGGACACG**	ONE_NON-GENE_SITE_358	ONE_NON-GENE_SITE_358
**TGATTTGTCGTATAAAACCG**	FRK	2444
**TGCAAACAGAGCACACCGAG**	TIAM2	26230
**TGCAAACAGATATATAGTAC**	MAPK1	5594
**TGCAACTCGCCGTACATCGT**	MAP2K2	5605
**TGCAAGATTTCACCGCCTGA**	ONE_NON-GENE_SITE_359	ONE_NON-GENE_SITE_359
**TGCAATCTTCAGTTCCCGAG**	SEC23B	10483
**TGCACAAGGACATCAAGCCG**	STK11	6794
**TGCACAAGTCTGGCGTTCTG**	SREBF2	6721
**TGCACAGAACTATCGTACCA**	CBLB	868
**TGCACAGAGGACATCAATAG**	CNKSR1	10256
**TGCACATCGTTTCCAAACCT**	GRB2	2885
**TGCACCGTTACAGAGCACCT**	MCM3	4172
**TGCACGACCTCGCTTTCATG**	ONE_NON-GENE_SITE_360	ONE_NON-GENE_SITE_360
**TGCACGTAGTCTGAGTGCTG**	XBP1	7494
**TGCAGACTCAGCAAAAGCCA**	MAF1	84232
**TGCAGCCAAGAATGGGCATG**	BARD1	580
**TGCAGGGCCGTGCTTCGATG**	ARHGAP35	2909
**TGCAGGGCTGATCTATGACT**	HDAC7	51564
**TGCAGTAGCGGCCATAGACG**	VAV1	7409
**TGCAGTCGGTCCACTCTGAG**	HDAC7	51564
**TGCAGTGGTACCCGGAGGTG**	RAC3	5881

**TGCATATGACAGGAACGCAG**	FANCA	2175
**TGCATCCTGTTCAAAATGGA**	ATP6V0A1	535
**TGCATCGGAGCAGCTATCGT**	ONE_NON-GENE_SITE_361	ONE_NON-GENE_SITE_361
**TGCATGGGGAGGACCAGTCG**	ETS1	2113
**TGCCAACCATGATCACCTTG**	RALB	5899
**TGCCACATCCCGAACTGACC**	CDK4	1019
**TGCCACTGACAAGGACCTGT**	FGFR3	2261
**TGCCACTTACATGTACTCAA**	RPS6KB2	6199
**TGCCAGCACACACTCATGCC**	NUP37	79023
**TGCCAGCATTGCTGCAATCG**	EXOSC9	5393
**TGCCATACCGAATCCGTGTG**	RPL31	6160
**TGCCATCAAACTCGTTGGCG**	BPIFB2	80341
**TGCCATCTTGACAACTATGC**	CAMK2D	817
**TGCCCACGTAGGAGCAGGCG**	RCE1	9986
**TGCCCCCCTTAGCTAAACTG**	RASSF8	11228
**TGCCCTCTGCCAGTTCTATG**	ENOSF1	55556
**TGCCCTCTGCCAGTTCTATG**	TYMS	7298
**TGCCCTTGGTGGGGTACAAG**	FBXO11	80204
**TGCCGATAGGAGTCCACCAG**	TP73	7161
**TGCCGATGACATTCTCATGG**	MAPK3	5595
**TGCCGCCAAATCATCCCAGT**	LUZP4	51213
**TGCCTCATATCCAAATGTGA**	SLC11A2	4891
**TGCCTGGTAGAGAACGCTGT**	FGFR4	2264
**TGCCTGTGAAGCGAAGTCAT**	PIK3R5	23533
**TGCCTGTGCCATTACGCTAA**	ONE_NON-GENE_SITE_362	ONE_NON-GENE_SITE_362
**TGCCTTGTTGAGTCGGACCA**	BPIFB2	80341
**TGCGAAACGCCAGAACAGCG**	PANX1	24145
**TGCGCACAACACGTTTATGC**	ONE_NON-GENE_SITE_363	ONE_NON-GENE_SITE_363
**TGCGCAGGCAGATAAATGCA**	GPRC6A	222545
**TGCGGCCCCCATCATCACGA**	SPRED3	399473
**TGCGGCCCTCAAAGGACCGG**	TP73	7161
**TGCGTACTCATACTTCTATG**	PSMD11	5717
**TGCGTATCACGCTTCCCGCA**	DUSP1	1843
**TGCGTATGTCAGCAAGCCTG**	MET	4233
**TGCTAAGAGTGTCGGTGCCG**	VKORC1L1	154807
**TGCTAAGTGCAACCGCTGAA**	ONE_NON-GENE_SITE_364	ONE_NON-GENE_SITE_364
**TGCTAATTGGAATTGCTGCG**	TSPAN13	27075
**TGCTACAGCACCACGCCGGG**	EIF4EBP3	8637
**TGCTATACATGCCGCCCCAG**	ELK1	2002
**TGCTCATGGGATACTCGCTG**	PAK4	10298
**TGCTCATTCCCGCAAACTGG**	SHC3	53358
**TGCTCCCCACAATAGGACAT**	RUNX1	861
**TGCTCTGGAAGACAGCGACG**	ARHGEF2	9181
**TGCTGCCAAGAAGGGCCAGA**	CHMP3	51652
**TGCTGCCAAGAAGGGCCAGA**	RNF103-CHMP3	100526767
**TGCTGCCCGCAATCACTCTG**	HDAC7	51564
**TGCTGCTTGGAGTTAATAGT**	ETS1	2113

**TGCTGGATGATCCTCGTCTG**	SREBF2	6721
**TGCTGGGTCAAGGCCAGGAG**	UBE2Q2L	100505679
**TGCTGTATCCCCAGACCCAA**	JAK3	3718
**TGCTTCTTACAGAGATCAGC**	SPC25	57405
**TGCTTGCAAAGGAATGCGCT**	AURKA	6790
**TGGAAAGGTGAAACCCATCA**	STAM	8027
**TGGAACATCAGACCAAGATG**	ATXN3L	92552
**TGGAACATCAGACCAAGATG**	GS1-600G8.3	100093698
**TGGAAGCCCAGATGGAACAA**	DPM1	8813
**TGGACACCGGGTAATTAGCA**	TRAPPC4	51399
**TGGACAGTGACCGTGCGCAG**	DAB2IP	153090
**TGGACATCTCGGCGAAGTCG**	BCL2	596
**TGGACCGGAGCCGTCGCGGG**	EXOC8	149371
**TGGACCTATCCTTGCACCAG**	ECT2	1894
**TGGACTTACACAAAGAACAG**	PIK3R5	23533
**TGGAGATCAAACCCGCAATC**	MAP2K1	5604
**TGGAGCAGGAATCAAATAAG**	ROCK1	6093
**TGGAGCGTCAGTATCAACTG**	PTGS1	5742
**TGGAGCTGTAAAACATCATC**	TSPAN13	27075
**TGGAGCTTAACTTACGGATG**	GRB2	2885
**TGGAGGAGTTTGCCTGGCGT**	RIMS4	140730
**TGGAGGGACAGCTTGCCTTG**	FANCA	2175
**TGGAGGGGGTGGCAGTTACT**	SUZ12	23512
**TGGAGTTTAGAGGACGATGA**	DDX46	9879
**TGGATACTACATTACCACCC**	FYN	2534
**TGGATCGGGTTCTAAAGGAA**	HSPE1	3336
**TGGATCGGGTTCTAAAGGAA**	HSPE1-MOB4	100529241
**TGGATCGGGTTCTAAAGGAA**	HSPE1P2	326300
**TGGATGAGCTCGATGCGGAA**	PIK3CD	5293
**TGGATGCAGATAAACCACAG**	MON2	23041
**TGGATTCTTACACTTAGGGT**	ONE_NON-GENE_SITE_365	ONE_NON-GENE_SITE_365
**TGGATTGTTATGGAGTACTG**	STK3	6788
**TGGATTTGATGCCCTGCAAG**	HDAC6	10013
**TGGCAACTGGACGTTCCCCA**	CREBBP	1387
**TGGCAAGCAGGGTATTATCG**	PREB	10113
**TGGCAGGAAGACCCTCTGCA**	TBC1D3B	414059
**TGGCATGGGGTAGTAGCGCT**	COX4I2	84701
**TGGCATGTTGAGCTGCACGT**	RALGDS	5900
**TGGCCAAACTGAAAAGGCTC**	PHAX	51808
**TGGCCAAAGCGATAGCTGAC**	UGCG	7357
**TGGCCATTACGCAACGTACT**	EXOC2	55770
**TGGCCGAGGTCACCCGCGAA**	PRKCSH	5589
**TGGCCGGCCCAAGATCGACG**	VAV1	7409
**TGGCCGGCTTGATCTCAAGG**	LOC407835	407835
**TGGCCGGCTTGATCTCAAGG**	MAP2K2	5605
**TGGCCGTCTTGATCATCCAG**	PLEKHF1	79156
**TGGCCTCACTTCATTCCCCT**	XBP1	7494

**TGGCGAAGAGCAGCAGGTCA**	E2F1	1869
**TGGCGCTGCTCGGACACCGT**	PRKAG2	51422
**TGGCGGCCCACAAACTGTGC**	RIMS4	140730
**TGGCGTAGACACGTCCATCT**	CERS2	29956
**TGGCTAGACTATACGTTGTG**	ONE_NON-GENE_SITE_366	ONE_NON-GENE_SITE_366
**TGGCTATAATGAAACCACAG**	PIK3R1	5295
**TGGCTATCTGACCACACTAG**	NPRL2	10641
**TGGCTCACCTGACCACGTTG**	CDK6	1021
**TGGCTGAGATGCTCTCTAAC**	MAPK3	5595
**TGGCTTCATTAAAGTACAGA**	RASSF3	283349
**TGGCTTCCTTTATGACTAGG**	C12orf77	196415
**TGGCTTTGGAACCGACACTG**	RALGAPA1	253959
**TGGGAAAATTTATTCGAATT**	C9orf116	138162
**TGGGAAGATAGAGCGAAGCC**	PAK3	5063
**TGGGACACAAACTCGTGACA**	FANCC	2176
**TGGGACCCCACTACACGACC**	DUSP4	1846
**TGGGACCTAGAGTTCTACGA**	ONE_NON-GENE_SITE_367	ONE_NON-GENE_SITE_367
**TGGGACTCCCATGCTAGCAG**	FGFR1	2260
**TGGGAGAAACCTTCCGGTCA**	VPS11	55823
**TGGGAGCAGCACACCGTCTG**	RAC2	5880
**TGGGCAGCCTTTAATTGAGC**	LUZP4	51213
**TGGGCAGGTAAAAGGAAGTG**	RASSF1	11186
**TGGGCATCCATGAGGTGGAG**	RASSF4	83937
**TGGGCCATCACAGTTGTCAT**	ARSI	340075
**TGGGCCGTTCTGCCGTGACG**	IRS1	3667
**TGGGCCTCTGTAAACGGCAC**	ONE_NON-GENE_SITE_368	ONE_NON-GENE_SITE_368
**TGGGCGAGAGCGATCCGTTG**	TCF7L2	6934
**TGGGCTGCAGCGAGAGATTG**	FOSL1	8061
**TGGGCTGCTAAATAAATGTG**	HDAC9	9734
**TGGGCTGGCATAGGTCAATG**	SMC1A	8243
**TGGGCTGGGATGACGAACTG**	PRKAG3	53632
**TGGGGAATAAGAAGGACCTG**	RHOC	389
**TGGGGCTTGGAAGACATGCA**	HCK	3055
**TGGGGGCGCGGCCACGCCCG**	ZFPM1	161882
**TGGGGGGTAAAGATTGCCCC**	ELOVL1	64834
**TGGGGTATTGGATTTGAACA**	E2F3	1871
**TGGGTCAAGACGATGGCTAG**	VPS18	57617
**TGGGTCAATGCCAGGCGATG**	HDAC3	8841
**TGGGTCATGATCTCTGAACA**	PREX2	80243
**TGGGTCATGCGGATTCTATG**	HDAC2	3066
**TGGGTGACCTCAAACTCCGC**	VPS16	64601
**TGGGTGTCCAACTCCACGCG**	VPS11	55823
**TGGGTTGGATGCCACTAACC**	ONE_NON-GENE_SITE_369	ONE_NON-GENE_SITE_369
**TGGGTTTGCACAATAAGGGA**	SPRY3	10251
**TGGTAACACTGTGGTCCACA**	EZH2	2146
**TGGTACAGCCTCATTTCCTG**	FOSL1	8061
**TGGTAGATGTGCTCGTTCTG**	RAPGEF1	2889

**TGGTCAAGATAATGCTTCCC**	MAPK1	5594
**TGGTCACGTGCATTGCGGTG**	PANX1	24145
**TGGTCCATTCCATTGCGACT**	ONE_NON-GENE_SITE_370	ONE_NON-GENE_SITE_370
**TGGTCCGCACTACGAATCTG**	AUP1	550
**TGGTCTACCGACTCATCAAG**	ARAF	369
**TGGTCTGGTAGTACCCGTCC**	EXOC3	11336
**TGGTCTTCGGAGAATGTCTG**	ICMT	23463
**TGGTGAGAGGTACATGCTGA**	SLC50A1	55974
**TGGTGATCACACGTTCCACC**	RPS18	6222
**TGGTGATGCCATACCATGTG**	ROS1	6098
**TGGTGCGGCCCATTAGTCGA**	LPCAT3	10162
**TGGTGCTGATATTACAAGCC**	FLVCR1	28982
**TGGTGGCAAAAACGGGCAGG**	PIN1	5300
**TGGTGGCATTCAATGTCGTC**	ITFG1	81533
**TGGTGGCCACTGGTACAAGG**	FGFR4	2264
**TGGTGTAAACAGCCAGACGT**	DTYMK	1841
**TGGTGTCAAAGACTCGAATG**	KLHDC3	116138
**TGGTGTGGACCCTCCCGATG**	RPTOR	57521
**TGGTTAAGCACATCGATGGA**	EXT2	2132
**TGGTTCGCTACTGACCCGGG**	RGL3	57139
**TGGTTCTGCCAGCAAACGCT**	UBE2N	7334
**TGGTTGCACAGCCAGCAGGA**	SPRY3	10251
**TGGTTGTGTAACTGATCAGT**	RAC1	5879
**TGGTTGTGTAACTGATCAGT**	RAC1P2	442775
**TGGTTGTGTAACTGATCAGT**	RAC1P4	286472
**TGGTTTAGAAAACTGAATAC**	SPRED1	161742
**TGGTTTCATTCGAATGACGG**	JAK1	3716
**TGTAACTGGGAATGAGGCTA**	RAB6A	5870
**TGTAATGTACGTGCTCACCA**	PIK3R6	146850
**TGTAATTAACACTCCGTACG**	ONE_NON-GENE_SITE_371	ONE_NON-GENE_SITE_371
**TGTACAGCGCCAGCAGCAGC**	CXCL14	9547
**TGTACATCGTATTACACCTG**	RPS4X	6191
**TGTACATCGTATTACACCTG**	RPS4XP2	92507
**TGTACCATCTATCCACGGAG**	NO_SITE_5	NO_SITE_5
**TGTACTGATATGTAGGCACT**	CASP7	840
**TGTACTTCATCACTATCTCC**	BCL2	596
**TGTAGACAATCATGAAGCCA**	ELOVL1	64834
**TGTAGGCACTCGGTCCCGGG**	CASP7	840
**TGTAGTAGGTGAAGATGATC**	DPM2	8818
**TGTAGTTGTGAACCTTGAAG**	PRKCD	5580
**TGTATATAGAGGTTCTGCGT**	ONE_NON-GENE_SITE_372	ONE_NON-GENE_SITE_372
**TGTATATAGGATAGAGACCT**	TM9SF3	56889
**TGTATTCCAGATATCTGGTG**	CDC23	8697
**TGTATTCCCCTGCACGCCAG**	KSR1	8844
**TGTATTTAAAGCAATACACA**	STK3	6788
**TGTATTTATAGGTTGTGTGT**	METTL5	29081
**TGTCAATGTAGAGCACCCGA**	HDAC3	8841

**TGTCACACAATGTAATTCAG**	APC	324
**TGTCACACAGTTTAACAAGG**	RGL2	5863
**TGTCACCACATAATTACCTG**	EGFR	1956
**TGTCAGGCAAAATCAGAAAG**	SNRPD3	6634
**TGTCAGTTGTCAACACCTTA**	TTK	7272
**TGTCATAGTAAGTGCCAATG**	EZH2	2146
**TGTCATCAGGAATTTCTTGA**	CDC6	990
**TGTCATGGAGTACGCCAACG**	AKT1	207
**TGTCATTTGGGTTAACACTT**	TSPAN13	27075
**TGTCCACTGGGCCGAAGAGG**	CDKN1A	1026
**TGTCCCAAAAGAGCTAGTTA**	BRCA2	675
**TGTCCGTCAACATTGAGAGA**	VHL	7428
**TGTCGAAGATGCTGAGCTGT**	YAP1	10413
**TGTCGATGGAGAAGCACATG**	RHOC	389
**TGTCGCAGAGGGGCTACGAG**	BCL2	596
**TGTCGCCTCTCCTCGCGTCG**	RSPH3	83861
**TGTCTCAATATAACGAGCAT**	ONE_NON-GENE_SITE_373	ONE_NON-GENE_SITE_373
**TGTCTCCCGACGGACCTCCG**	DUSP5	1847
**TGTCTCGAACCTCATTGCAA**	PRKCI	5584
**TGTCTGAACAATCTATTGCA**	ATXN3L	92552
**TGTCTGAACAATCTATTGCA**	GS1-600G8.3	100093698
**TGTCTGACAAATGGCCATAT**	SLC22A25	387601
**TGTCTGCATAGTTCTGGCCA**	CHMP3	51652
**TGTCTGCATAGTTCTGGCCA**	RNF103-CHMP3	100526767
**TGTCTGCTCTAGTAATAAGC**	CTNNB1	1499
**TGTCTGTTCCAACATAAACG**	PREX2	80243
**TGTCTTCCGCAGGTCCTGCA**	C4orf48	401115
**TGTGAAGGATATTGACCCGG**	POLR2H	5437
**TGTGACCAAAACACCCCCAA**	EIF4EBP1	1978
**TGTGAGAAAGTTCAACGAAA**	TFDP2	7029
**TGTGATAAGATAAATAGTCT**	RASSF6	166824
**TGTGATCCAAGCTGTCCCAA**	EGFR	1956
**TGTGATCCTAGAATTGTCCA**	MED11	400569
**TGTGATGGAGTACGTGAATG**	PRKCB	5579
**TGTGCATATTTATTACATCG**	PTEN	5728
**TGTGCGACACGACTTCTCCA**	EXOC7	23265
**TGTGCGCTGAGGGTACGGTA**	ONE_NON-GENE_SITE_374	ONE_NON-GENE_SITE_374
**TGTGCTCACTCTTCGACCCA**	CHSY1	22856
**TGTGCTGAGTTCCATTACCG**	HDAC6	10013
**TGTGCTGGCCCGAATTGATG**	RTN4R	65078
**TGTGGACCAACTTCTGAAAG**	FNTA	2339
**TGTGGAGAACCAACCAAGGT**	EXOC4	60412
**TGTGGATACTCCATTTGGCA**	MTAP	4507
**TGTGGATGCTGAACTTGTGT**	PRKCH	5583
**TGTGGCACAGAGGGCAACGA**	CCND1	595
**TGTGGCCGTTGTCCTTCACG**	RPTOR	57521
**TGTGGGATGTCGACTCCTAG**	CBLB	868

**TGTGGTAAGGCATATCCAAG**	PDGFRB	5159
**TGTGGTGTGGTTGATCGCAA**	ONE_NON-GENE_SITE_375	ONE_NON-GENE_SITE_375
**TGTGGTTGAAGTAGTACACT**	PIN1	5300
**TGTGTACGGCTTTGTGAACA**	MZT1	440145
**TGTGTATATCAATGCCAGGG**	PLCE1	51196
**TGTGTCACAGCTCAATCTCG**	RASGRP1	10125
**TGTGTCCAGACCGTCCTATG**	ROS1	6098
**TGTGTCTCCATATAAAACCA**	BRCA2	675
**TGTGTGACAAGGTCGTCCCT**	MCM5	4174
**TGTGTTTAGAGTGCATCGAA**	PSMD11	5717
**TGTTATAGGGCAAATAATAG**	PIK3CA	5290
**TGTTATTGCATGGACGATCA**	HACD2	201562
**TGTTCCCAGAGCAACACCTA**	HDAC7	51564
**TGTTCCCCGAGACAATGCAT**	TIAM2	26230
**TGTTCCTCAGGAAAATGATG**	RPS4X	6191
**TGTTCGTACTTACACCCATG**	CDK2	1017
**TGTTGATCCGGCCACGAAAA**	PREX2	80243
**TGTTGATGGCAAACACACAC**	HRAS	3265
**TGTTGCAGTAAAGATCCTAA**	RAF1	5894
**TGTTGCGCCAACCAACCCAA**	MCM6	4175
**TGTTGCTGATGGCATAATCC**	SPRY2	10253
**TGTTGTTGTTAGATGCATTG**	SOS2	6655
**TGTTTAGGCTGGGATCATAG**	CHMP3	51652
**TGTTTAGGCTGGGATCATAG**	RNF103-CHMP3	100526767
**TGTTTAGGGAGGTACGACTT**	ROCK2	9475
**TGTTTCAAAGCATGTGACAG**	VPS45	11311
**TTAAAATTCAGCAATACCTG**	CHCHD3	54927
**TTAAACTATGGACGACTCCG**	YAE1D1	57002
**TTAAACTGAACTCCAAACAA**	EED	8726
**TTAAAGAGCTCAACAAATGC**	SHOC2	8036
**TTAACTAACCACCAACATGA**	STAM	8027
**TTAACTCAGAGCTAGTCGAT**	ONE_NON-GENE_SITE_376	ONE_NON-GENE_SITE_376
**TTAAGCCACATGATTTAAGG**	ST3GAL6	10402
**TTAATCATGTCGCTGATACA**	LYN	4067
**TTAATGAGCATATTACCCGG**	ONE_NON-GENE_SITE_377	ONE_NON-GENE_SITE_377
**TTACAAAGAGATCTCCGTAT**	RASA2	5922
**TTACACAAACATCGGGTCAT**	ONE_NON-GENE_SITE_378	ONE_NON-GENE_SITE_378
**TTACACAAAGTCATCATGGT**	RALA	5898
**TTACACTTGTGACCAGTGTG**	MCM7	4176
**TTACAGATTAGAGATAACCT**	RALGAPA1	253959
**TTACATATTAAAGCCTCATG**	BRCA1	672
**TTACATATTATAGCAATCGT**	ROCK1	6093
**TTACATCAGTAGCTGTCAGA**	PSMA6	5687
**TTACCAAAAGTTTCTCCACG**	PDPK1	5170
**TTACCAAGGACACGTCATCC**	PHB	5245
**TTACCAATGTAGCTCCCAAG**	SHC1	6464
**TTACCCTCCGTTGAAAACGT**	PSMB1	5689

**TTACCGGCACGCACCCGTCA**	SPRED2	200734
**TTACCTTATGCAGCCACAGT**	PANX1	24145
**TTACGGTTCGATCAATCTCA**	ONE_NON-GENE_SITE_379	ONE_NON-GENE_SITE_379
**TTACGTCAATGATATCGTCT**	HDAC1	3065
**TTACTACAAAAACAACATGA**	BLK	640
**TTACTATAACAACAGTACCA**	LYN	4067
**TTAGAAAATTTGGAAAACAC**	ATXN3L	92552
**TTAGACACATCATGGATCAG**	DDX46	9879
**TTAGACCATCTACATAGCCT**	RPS6KA3	6197
**TTAGATCGCGATGCACTACT**	CDK6	1021
**TTAGATGGCTCCCGCAAAGA**	RPL31	6160
**TTAGCAATGTGTCACCAGAT**	STAM	8027
**TTAGCATCAAGGCATGCCAT**	RASA2	5922
**TTAGCATCTTGTTCTGTTTG**	PTEN	5728
**TTAGCATCTTGTTCTGTTTG**	PTENP1	11191
**TTAGCTTGGAAGCTGACAAG**	JAK3	3718
**TTAGGATAGGCCAATAACTG**	UBE2H	7328
**TTAGGATCTACCCCTTTCAG**	UGCG	7357
**TTAGTTTCTGATTCAAAGGC**	RASSF6	166824
**TTATAAGAAGAATCCTATGG**	PTPN11	5781
**TTATATAAACATCAACACAA**	EXOC5	10640
**TTATCACGGCAACATAATTG**	EXOC1	55763
**TTATCATCAGATATCTTTAG**	DNAJC9	23234
**TTATCGAGCTGTTGAAAGTG**	PHAX	51808
**TTATCGGCGATATGGTGATG**	INSR	3643
**TTATCTCATACATACTTGGT**	BIRC2	329
**TTATCTGCAGCATCTCCAGA**	HDAC8	55869
**TTATCTTGCACAAAATGTTG**	PRKCQ	5588
**TTATGACAGAAAGTTTCTGT**	EIF4EBP2	1979
**TTATGATACCTTTCAAGTGA**	INPP4B	8821
**TTATGCATCCAAGAGCCCAA**	PDPK1	5170
**TTATGGCAACCAAGAAAGCA**	CTNNB1	1499
**TTATGTACGCTTCTACACTG**	RAB3GAP2	25782
**TTATTAGTGTACTCACGATG**	ONE_NON-GENE_SITE_380	ONE_NON-GENE_SITE_380
**TTATTCCCAACCAACATAAT**	RHEB	6009
**TTATTCCCCCGGCTTGACTG**	TP73	7161
**TTATTCTCCGAAAGAAAGAC**	ELOVL1	64834
**TTATTGGTATATTAATGAGG**	CNKSR2	22866
**TTATTTACCACGCATACCCG**	ONE_NON-GENE_SITE_381	ONE_NON-GENE_SITE_381
**TTCACAGGGTCGATTTCACA**	RASA3	22821
**TTCACCGACTTCCTCCGCCG**	SOX9	6662
**TTCACTGTTGAACGAGCCAA**	RALGAPA1	253959
**TTCAGATCAATTCCTCAACA**	EXOC4	60412
**TTCAGCAGAAAAGTATAGCG**	FUNDC2	65991
**TTCAGCTTAACTGCCAAGGT**	PREX2	80243
**TTCAGGTGACAAGTTTCGGT**	POLR2H	5437
**TTCATACAGCCATTGCTACT**	RASSF3	283349

**TTCATCCACAAACCCGCGCA**	SHC2	25759
**TTCATCCTCACGGACGACCA**	ARSI	340075
**TTCATGCCCGTATATTTCTG**	MCM7	4176
**TTCATTAGCATTCGGCCTTG**	PRKCQ	5588
**TTCATTGCACCGCACATCGA**	POLR2C	5432
**TTCCAAGGGAGTGAAAATCT**	TYMS	7298
**TTCCAATACAAGCACTCTCA**	RALGAPA1	253959
**TTCCAATACAAGCACTCTCA**	RALGAPA1P1	26134
**TTCCACAAATCAAAGGCAGC**	MSMO1	6307
**TTCCAGAAGAGAAAAAGCCC**	PAK2	5062
**TTCCAGCCTTCAACGGCCAA**	ONE_NON-GENE_SITE_382	ONE_NON-GENE_SITE_382
**TTCCATCGGATCTCGTAACG**	PDGFRB	5159
**TTCCCAACGATGAACCGCCG**	PRKCD	5580
**TTCCCCACTGACTATACCTG**	PLCE1	51196
**TTCCCGGCGTACCATCACTC**	ONE_NON-GENE_SITE_383	ONE_NON-GENE_SITE_383
**TTCCCTAGGTACTTCCAATG**	ESRP1	54845
**TTCCGATCTTCTGTCCATCG**	RGPD5	84220
**TTCCGATCTTCTGTCCATCG**	RGPD6	729540
**TTCCGATCTTCTGTCCATCG**	RGPD8	727851
**TTCCGCAAAATAGAGCCCCA**	CBLB	868
**TTCCGCTGCTCGACACCACG**	B3GAT3	26229
**TTCCGGAGACACTTCAGCAT**	VPS16	64601
**TTCCGGCCCAGAACCACGTG**	OSGIN1	29948
**TTCCGGGTCAAAGCATCTGG**	PAK1	5058
**TTCCTAAGGAACTCTCCACA**	APAF1	317
**TTCCTCAAGTACCGAATGGT**	ONE_NON-GENE_SITE_384	ONE_NON-GENE_SITE_384
**TTCCTCAGGAAAATGATGAG**	RPS4X	6191
**TTCCTCCAACACGTCCAAGT**	DUSP6	1848
**TTCCTGTTGGATGACGTCAG**	HDAC10	83933
**TTCGAAAGGATCTCAGAGCT**	MAP2K2	5605
**TTCGAGTCTCCATTGCACAA**	KDM3B	51780
**TTCGATTGTGTTCCATGTTG**	SHOC2	8036
**TTCGCCTGGGAGCGTGTGCG**	CDKN1A	1026
**TTCGGAATGATGAGCACACA**	RHOA	387
**TTCGTTATTTCTAACACCAC**	FNTA	2339
**TTCTACATTAAAGGATCAGT**	ROCK1	6093
**TTCTAGGACAACGGTGCTCA**	ONE_NON-GENE_SITE_385	ONE_NON-GENE_SITE_385
**TTCTCACCATAGGGTGACCG**	GMDS	2762
**TTCTCATAGATGAACTACAC**	EXOC4	60412
**TTCTCATTAGAAGTGAGGCG**	TRAPPC4	51399
**TTCTCGGGATACAGACCAAT**	PIK3CA	5290
**TTCTGAATAAAGCTACAGCT**	BIRC2	329
**TTCTGGATAAAAAAGTCGGA**	ST3GAL6	10402
**TTCTGTTGGCCACCCTTGTG**	SLC11A2	4891
**TTCTTACACAGGCTTCATCA**	RASSF1	11186
**TTCTTACCAATAGTTTGAGT**	SUZ12	23512
**TTCTTATCCGACCAGTACAG**	ECT2	1894

**TTCTTCAGGAGAGCGCCCCA**	RASSF3	283349
**TTCTTCATGTCATAGAACCA**	CERS2	29956
**TTCTTCCAGATATCCTCGCT**	MYC	4609
**TTCTTCCAGTTCTCTAGTGA**	SPRED1	161742
**TTCTTCTCAGTCATTTGTAA**	LOC100420464	100420464
**TTCTTCTCAGTCATTTGTAA**	SAV1	60485
**TTCTTGTGTTCGATGCACAC**	NPRL2	10641
**TTCTTTAATATAACGATCGT**	SLBP	7884
**TTCTTTGTAAGGCGCCACAC**	ONE_NON-GENE_SITE_386	ONE_NON-GENE_SITE_386
**TTGAAGACATCTCTTCATCG**	BIRC2	329
**TTGAAGACCCCACCAGACCT**	E2F2	1870
**TTGAAGTTCTGCGCTCAATG**	SHC3	53358
**TTGACCTAGAGCGTCAACGC**	ONE_NON-GENE_SITE_387	ONE_NON-GENE_SITE_387
**TTGACTCATAGAGCCCCAAT**	ONE_NON-GENE_SITE_388	ONE_NON-GENE_SITE_388
**TTGACTGCATTCACCACCTA**	MAF1	84232
**TTGAGAACAGCTTTACAAGT**	SEC23B	10483
**TTGAGCACCTTCCTAACTGT**	FAM20B	9917
**TTGAGCATACGATTCATGTG**	PAK3	5063
**TTGAGCGATGTGTTACAAGC**	EXT2	2132
**TTGAGCGGGACCTTCCCACG**	PIK3R6	146850
**TTGATATTGGGCAACGCCCA**	ONE_NON-GENE_SITE_389	ONE_NON-GENE_SITE_389
**TTGATATTTAGGCTTGCCGA**	CASP7	840
**TTGATTTCAAGTCCAACCAT**	CHD2	1106
**TTGCACATAGGGGAAACCGT**	FGFR4	2264
**TTGCCATCATCATAGACCTG**	RPS6KA2	6196
**TTGCGCGGGACTAGGGAGGT**	EZH2	2146
**TTGCGGGCCCACAGCGAGAA**	OSGIN1	29948
**TTGCTGCACGGGGTTAACAA**	ETS2	2114
**TTGCTGCAGACGCCCCGTGA**	SPRY2	10253
**TTGCTTACCAATTCTGGAAG**	SPTLC1	10558
**TTGGAAAGGTTGTTCTGAAG**	SLC30A1	7779
**TTGGAAGTAACCCTAGAAGG**	FERMT1	55612
**TTGGAATAAATTCAAAAGTA**	SPC25	57405
**TTGGAATTGTTGGACCATGC**	SAV1	60485
**TTGGACAATAACTTGCCCAG**	UBAP1	51271
**TTGGACATACTGGATACAGC**	NRAS	4893
**TTGGAGTTCTAAGGAAACTC**	LOC100420464	100420464
**TTGGAGTTCTAAGGAAACTC**	SAV1	60485
**TTGGATTGGTGGAAAGAGTC**	TMEM263	90488
**TTGGCTCAGATGCTGAGGCA**	SPRY3	10251
**TTGGGATATGTGCTGCTAGC**	SMAD5	4090
**TTGGGGAACTCAGTGATCTG**	PRKAG1	5571
**TTGGGTATGTTACTGCTGAT**	CBL	867
**TTGGTATTTACCATGCTCTG**	CDC37	11140
**TTGGTGCAGAAAGTACAAAG**	INTS6	26512
**TTGGTGCATTGACCTAGAGG**	CDC37	11140
**TTGGTGCTGAGGAAAGGTGG**	COX4I2	84701

**TTGTAAAGAGCCCTGAGCAG**	STK4	6789
**TTGTACTACAGACACAGCCT**	ROS1	6098
**TTGTACTGATGCTCTCCTTC**	PRKAB1	5564
**TTGTAGATGACAATGAGGTG**	NFE2L2	4780
**TTGTAGGAGTATATATTCTG**	PEX3	8504
**TTGTATCACACGCATCCTAT**	ONE_NON-GENE_SITE_390	ONE_NON-GENE_SITE_390
**TTGTATCCGTAATCATGACA**	EXOC6	54536
**TTGTCCTTTGGAAACCCAAG**	FYN	2534
**TTGTCTATGAACATCTGTGG**	NFKB1	4790
**TTGTGAAGATTATGTGCATG**	NUP37	79023
**TTGTGAATGACATTCATACA**	EED	8726
**TTGTGCTCACCTGTGACGAG**	HDAC4	9759
**TTGTGTACGATGCAAGAGGT**	TMEM220	388335
**TTGTGTCCAAGAGCTCTGGG**	GID8	54994
**TTGTGTGCCCTCTAGGACGG**	MAP2K2	5605
**TTGTTGCTGGTGAAAAGGTG**	EXOSC9	5393
**TTGTTTGAAGCGCAACTCGA**	SHC1	6464
**TTTAATTCACCAGAAGAACT**	SOS2	6655
**TTTACAGTTCCGTCCCCGCA**	PSMD3	5709
**TTTACCTGGCCCATTTGATG**	HACD2	201562
**TTTACCTGTATAGGATCAGT**	LOC110117498-PIK3R3	110117499
**TTTACCTGTATAGGATCAGT**	PIK3R3	8503
**TTTAGGATACCATGGCCAAG**	STK4	6789
**TTTAGTAAATCTCATGACAC**	SHOC2	8036
**TTTATAAAGCTTGGGAAAGG**	ECT2	1894
**TTTATCTTTAAATCAGCTAA**	UNG	7374
**TTTATGGAGGAAGATATCCT**	WDR70	55100
**TTTATTTCATTCACTACTTT**	CISD2	493856
**TTTCAAGGATATAACCAGGA**	LYN	4067
**TTTCAATAACTTTCCCAAAG**	NFKB1	4790
**TTTCACAAGATTATCCAAAG**	RPS6KA6	27330
**TTTCACAAGCAGGACTTCCA**	SUZ12	23512
**TTTCACAAGCAGGACTTCCA**	SUZ12P1	440423
**TTTCACTAGACGGATCCTAC**	ONE_NON-GENE_SITE_391	ONE_NON-GENE_SITE_391
**TTTCACTTCCATTCTGTGTG**	TM9SF3	56889
**TTTCAGGCTACGTTTGCGAG**	C12orf49	79794
**TTTCCCAGAAAGGATACAGC**	ABALON	103021294
**TTTCCCAGAAAGGATACAGC**	BCL2L1	598
**TTTCCCGAATAGCAGGTGCA**	JAK1	3716
**TTTCCCGTCCACCATCACGT**	RAC3	5881
**TTTCCCTCTAAAGTAACATG**	HDAC9	9734
**TTTCCTAATATTGTACAACT**	APAF1	317
**TTTCCTACATTGAGTCTGCG**	ONE_NON-GENE_SITE_392	ONE_NON-GENE_SITE_392
**TTTCCTAGATACACCCTCCG**	PIK3R1	5295
**TTTCGCAGATTGTCCCAATG**	INPP4B	8821
**TTTCGGACCACGGTCGACCA**	ONE_NON-GENE_SITE_393	ONE_NON-GENE_SITE_393
**TTTCGTAATCCATGTTACCA**	ONE_NON-GENE_SITE_394	ONE_NON-GENE_SITE_394

**TTTCGTTATTCATTGCACAG**	BIRC3	330
**TTTCTATTGAAAATGACACG**	PCDH7	5099
**TTTCTGAAAGGACAGGACGA**	INTS6	26512
**TTTCTGGCTATCACTCCAAT**	ONE_NON-GENE_SITE_395	ONE_NON-GENE_SITE_395
**TTTCTTTGATTATCTCCTGG**	STK4	6789
**TTTGAACACACAATTCATGT**	PAK1	5058
**TTTGAATACTGGCTTAGATA**	MZT1	440145
**TTTGACAACAGGTACACCAG**	TUBGCP2	10844
**TTTGACAGCCCACTTTAGGG**	CASP7	840
**TTTGACAGCGACAAGAAGTG**	MMP9	4318
**TTTGAGGAAGAAGACTTGCG**	DUSP1	1843
**TTTGATACGTCCCTGCAGGT**	PRKAG1	5571
**TTTGATGCGTTTCAGAATGT**	EZH2	2146
**TTTGATGGGAGATTGAAAGG**	ATL2	64225
**TTTGATTGTCCAGAATTCTG**	FANCC	2176
**TTTGCAGATAAAAGTGCACA**	PAK3	5063
**TTTGCCCCTCTCACCAGCCG**	TUBB	203068
**TTTGCCTAGAGGCTATCACA**	RAPGEF2	9693
**TTTGCCTTGGGGGGTTTAGG**	PIK3R2	5296
**TTTGCGTTAGAGATCGGGAA**	ONE_NON-GENE_SITE_396	ONE_NON-GENE_SITE_396
**TTTGCTTTAAGATCCAGCAA**	TIAM2	26230
**TTTGGAGGCTCTGTAAAACT**	PDCD6IP	10015
**TTTGGCTCAAAAGAATGGTA**	DUSP3	1845
**TTTGGCTTGAAGCTCTGTGA**	PFDN1	5201
**TTTGGTAGCGCGCCTTGATG**	DAB2IP	153090
**TTTGGTAGGCTAGAAATACG**	BRCA2	675
**TTTGTAGTTCTCATGACGAG**	CDC25A	993
**TTTGTCAATAAGCCCACGCG**	SHC1	6464
**TTTGTCCATGGAGGCTGCAG**	E2F3	1871
**TTTGTCGTGAAGATGCACAA**	CREBBP	1387
**TTTGTGAAGATCTTGACCAA**	PTEN	5728
**TTTGTGAAGATCTTGACCAA**	PTENP1	11191
**TTTGTGCAGATCTTGCTGTG**	RPS6KA2	6196
**TTTGTTGTGACACTTCAACC**	KSR1	8844
**TTTTAATCCACGAGCCCTGT**	ONE_NON-GENE_SITE_397	ONE_NON-GENE_SITE_397
**TTTTAATCCACGAGCCCTGT**	INACTIVE_4T_5	INACTIVE_4T_5
**TTTTCAATATTGATCGCACT**	ONE_NON-GENE_SITE_398	ONE_NON-GENE_SITE_398
**TTTTCAATATTGATCGCACT**	INACTIVE_4T_6	INACTIVE_4T_6
**TTTTGTCAAACAATCCGAAG**	ONE_NON-GENE_SITE_399	ONE_NON-GENE_SITE_399
**TTTTGTCAAACAATCCGAAG**	INACTIVE_4T_7	INACTIVE_4T_7
**TTTTTACGGTATGCTTCCAC**	ONE_NON-GENE_SITE_400	ONE_NON-GENE_SITE_400
**TTTTTACGGTATGCTTCCAC**	INACTIVE_5T_8	INACTIVE_5T_8
