# Supplementary material for: Developing SHP2-based combination therapy for KRAS-amplified cancer
Source: JCI Insight. 2023 Feb 8;8(3):e152714. doi: 10.1172/jci.insight.152714 (PMC9977440; doi:10.1172/jci.insight.152714)
Supplement: Supplemental table 4 [file jciinsight-8-152714-s174.pdf]

NCBI gene name	KRAS-Amp average LFC	HUG1N_Average LFC	KE39_Average LFC
SHOC2	-0.849053638	-0.705793476	-1.070867237
RIC1	-0.664634809	-0.175073122	-0.07386207
GAREM1	-0.633357969	-0.111022561	-0.385776668
CRKL	-0.603319683	0.05147354	-0.691464542
RAPGEF1	-0.506082268	-0.168146203	-0.30599589
RUNX1	-0.481415975	0.046781265	-0.418776398
BRAF	-0.475473913	-0.40982565	-0.207155527
TCF7L2	-0.470729771	0.287593117	-0.395385254
CHD2	-0.459945366	0.220165426	-0.445184138
VPS16	-0.426570346	-0.393247976	0.050144453
ARID5B	-0.417067522	-0.236614235	-0.242428717
PFDN5	-0.416074033	-0.31470112	0.020717335
RAF1	-0.403037206	-0.148506848	-0.626574436
ELOVL1	-0.398082115	-0.348393073	0.039530661
SHC1	-0.382743707	0.186539286	-0.246892333
CREBBP	-0.37469734	-0.216351168	-0.38903409
SRC	-0.374487142	-0.117668189	0.02852831
ERBB2	-0.367671205	0.012245085	-0.047067747
PTK2	-0.36143423	-0.003393716	-0.710412846
PPP1CA	-0.330288982	-0.06638371	-0.000243993
PRKCE	-0.326442408	-0.260418456	-0.30893687
GATA6	-0.321955254	0.346033833	-0.491015324
RAC1	-0.31209957	-0.082078943	-0.569306988
PFDN1	-0.306308003	-0.46857023	-0.294679139
KRAS	-0.299735768	-0.087104769	-0.403869661
TRIM64C	-0.289420438	0.002068834	-0.117086657
RAB6C	-0.275942456	0.103403485	-0.276462272
RCE1	-0.275095425	-0.13621763	-0.03596661
YES1	-0.271562584	0.138114276	-0.092984149
PAK1	-0.261560016	0.053349611	-0.320305909
EGFR	-0.260974659	0.254557471	-0.211372077
SPNS1	-0.257515796	-0.712295448	0.056127257
UGCG	-0.256637556	-0.052946799	-0.616136209
SIRT1	-0.250138412	0.073929797	-0.438741096
NFE2L2	-0.248027367	-0.070899376	-0.120493286
SPTLC1	-0.234316482	-0.286857704	-0.098196821
TUBGCP2	-0.231081866	0.073584906	-0.113759938
VPS11	-0.230791999	-0.370664951	-0.091203055
MAP2K1	-0.22537657	0.133864957	-0.124322239
NEDD9	-0.224133048	0.111913292	-0.403852651
STAM	-0.222285062	-0.312131756	-0.235430416
CERS2	-0.221896976	-0.55578617	-0.140682248
INPP5A	-0.219176487	-0.047061779	-0.405040591
BPTF	-0.217788641	-0.30252938	0.021077077
SPTLC2	-0.211414348	-0.28087425	0.094481601
APC	-0.207614181	-0.051952662	-0.11703524

DPM2	-0.194347377	0.094445244	-0.517756535
PPP1R7	-0.193448199	-0.074949335	-0.196435645
HDAC4	-0.190542772	-0.101584916	0.177021273
METTL5	-0.1836834	-0.040419658	-0.413663992
SET	-0.182410935	0.107313945	-0.40768372
YAP1	-0.182294636	-0.155088647	-0.265200263
JUN	-0.181471525	-0.064740047	-0.290809296
VPS18	-0.181379764	-0.260202344	-0.286850251
PLCE1	-0.17898646	-0.007638288	-0.113491974
PLAGL2	-0.178332991	0.064473687	-0.642422574
JAK1	-0.173506168	0.132362922	0.175077355
BCL2L1	-0.172440822	0.161799474	-0.021936902
TMEM263	-0.172398251	-0.309871666	-0.068078744
FGFR4	-0.171906142	-0.121905297	-0.381775926
PREB	-0.169982437	0.031916592	-0.583229843
IRS1	-0.16907987	-0.039979347	0.154450614
VPS45	-0.164375815	-0.35765977	0.273316061
EED	-0.160525545	0.099925865	-0.350649539
FGFR3	-0.155976561	0.10987902	-0.178861904
NOX1	-0.149978883	0.088833534	0.098920434
CTNNB1	-0.145453343	0.102120817	-0.083719194
MAGEA12	-0.144124708	-0.144166926	-0.100602484
TUBB	-0.143492511	0.052203076	-0.100701541
HDAC6	-0.14211336	-0.05812194	0.110165149
LGALS7	-0.141765496	-0.091426257	-0.080765717
CNKSR1	-0.141174556	0.006949786	-0.053507987
ARSI	-0.138575976	0.166721635	-0.488739398
ST3GAL6	-0.134131781	-0.268083678	-0.005578212
BSCL2	-0.131695477	-0.281563757	-0.069498984
RALGAPA2	-0.130598344	0.228838817	0.09881878
STK3	-0.130165855	0.011555739	0.377958423
FLVCR1	-0.130015368	-0.320856415	0.456361387
RSPH3	-0.12965438	-0.292668052	-0.356639354
EZH2	-0.128926683	0.054066583	-0.190202306
CBL	-0.125992453	-0.250949222	-0.028011706
SLC22A25	-0.125646536	0.056432243	-0.421345781
FRK	-0.125456326	0.013449111	0.005802539
TP53	-0.12470297	-0.056525986	0.071436444
TIAM1	-0.124092187	0.076775003	0.085610325
LGALS7B	-0.123050205	-0.114016123	-0.140150242
CASP8	-0.12200876	0.101268717	-0.044279111
PANX1	-0.121844035	-0.260952835	-0.26569844
CASP7	-0.121054288	-0.198941249	-0.156351497
VPS39	-0.120734151	-0.305304404	-0.313429549
TRAPPC4	-0.11892367	-0.008309275	-0.328836772
TP73	-0.117944138	-0.098904141	0.110951265
KSR1	-0.117890114	0.111126409	-0.028536245

PIK3R2	-0.117884624	0.151511285	0.10508709
SPRR1A	-0.117345693	0.005616216	-0.158901559
SPRY1	-0.116797719	0.006438339	0.029882828
HDAC11	-0.116419858	-0.066938518	-0.006965199
RGL2	-0.114528296	-0.024779377	0.006730734
PIK3CG	-0.114427501	0.087143116	-0.068323514
KPNB1	-0.112694788	0.357485778	-0.465229454

KE39 Average LFCsecondary	CAT12_Average LFC	YCC1_Average LFC	GSU_Average LFC
-0.94761092	-0.3571548	-1.242395192	-0.710917346
-1.456155728	-0.650424115	0.112675416	-0.076872614
-0.811942482	-0.025729587	-1.062401837	-0.739719427
-0.742967739	-0.747375014	-0.319616295	-0.932790543
-0.352787124	-0.929604116	-0.235855565	-0.582358826
-1.811824239	0.067031341	0.300544972	-0.070427758
-0.80633452	-0.038446575	-0.581640645	-0.883558191
-0.67766503	-0.105701186	-0.628823097	-0.3465332
-0.170965332	-0.522602155	-0.686268613	-0.132948445
-0.523315773	0.0770044	-0.833399665	-0.208065072
-0.846632887	-0.296693261	-0.107876418	0.176347817
-0.50101117	-0.656894132	-0.090316796	-0.496902368
-0.619155089	-0.003991217	-0.585965311	-0.609396003
-0.372215527	-0.272530325	-0.549500492	-0.216634927
-0.174281678	-1.14833313	0.174383688	-0.44281358
-0.437219483	-0.408590155	-0.278282383	-1.455171239
-0.380393974	-0.502866115	-0.240201338	-0.241412421
-0.753637452	-0.414714534	0.065338372	-0.083026806
-0.472186475	-0.486247432	-0.125868782	-0.503163264
-0.319327301	-0.521217247	-0.150322399	-0.299753144
-0.212873449	0.040683342	-0.807137116	-0.695161777
-0.566126425	-0.083570175	-0.316169163	-0.198129149
-0.303353844	-0.607218391	-0.025726475	-0.589628066
-0.279019062	-0.695127951	0.055223004	-0.104067237
-0.608876544	-0.601575193	0.311244433	-0.289619922
-0.774391769	0.037202607	-0.131072152	-0.462930887
-0.134178927	-0.239479676	-0.454168766	-0.557564852
-0.011102967	-0.383804512	-0.430378796	-0.000239135
-0.218261277	-0.35589573	-0.240530745	-0.402277375
-0.363060494	-0.238431933	-0.183187623	-0.434812132
-0.591135872	-1.395886031	1.204097927	0.116815884
-0.02947583	0.02591253	-0.768984086	-0.280340023
-0.528062257	-0.10874899	-0.133101422	-0.083684845
-0.70377265	0.07979676	-0.126439347	-0.408321932
0.041506423	-0.431417564	-0.35417096	-0.508149392
-0.43650072	-0.098182973	-0.168265753	-0.322467988
-0.600993743	-0.256803569	0.164551714	-0.213616929
-0.324323816	0.171227061	-0.539279244	-0.383043499
0.055629784	-0.288504641	-0.443254853	-0.251823721
-0.153556687	-0.300588166	-0.218254292	-0.159948402
-0.210367089	-0.001046131	-0.455441965	-0.51767665
-0.21597452	-0.121221117	-0.328495291	0.12176719
-0.249119052	-0.385880287	-0.022530122	-0.061955571
-0.272314882	-0.371731386	-0.009319654	-0.269641138
-0.161594715	-0.096164361	-0.376483969	-0.132020805
-0.219960214	-0.130689604	-0.272192726	-1.160650657

	-0.300895246	-0.339225762	0.057078877	-0.47825326
	-0.160682895	-0.309141778	-0.110519924	-1.042666407
	-0.229846282	-0.183163245	-0.158618789	-0.42841275
	-0.232465099	-0.26189476	-0.05669034	-0.459024957
	-0.154390162	-0.029657467	-0.363185177	0.057327021
	-0.273212021	-0.320986696	0.047314808	-0.131014939
	-0.630699123	0.177671979	-0.09138743	0.299327394
	-0.273572351	-0.081280669	-0.189286272	-0.194290176
	-0.103216189	-0.187952781	-0.24579041	-0.318791061
	-0.291035375	-0.122055159	-0.12190844	-0.364948486
	-0.171116825	-0.388698618	0.039296937	-0.129216416
	-0.108446715	-0.353046734	-0.055829017	0.167085649
	-0.068663169	-0.31791284	-0.130618745	-0.259499596
	-0.486300522	-0.004784744	-0.024633159	-0.267083454
	-0.271140221	-0.316742823	0.077935734	-0.704025938
	-0.107106243	-0.293022137	-0.107111231	-0.240257637
	-0.131557156	0.165497186	-0.527067475	0.146367996
	-0.200150971	0.168307033	-0.449732698	-0.176845447
	-0.480375291	0.025366368	-0.012920761	-0.240072604
	-0.286327351	-0.070110826	-0.093498472	-0.239490666
	-0.487254647	0.222732843	-0.171838225	0.533072415
	-0.039226711	-0.218560979	-0.174586433	-0.111550145
	-0.207244169	-0.420558672	0.197325308	-0.379960492
	-0.342230747	-0.150866674	0.066757342	-0.158283092
	-0.161547642	-0.219442784	-0.044306062	-0.37586568
	-0.108391597	-0.238906505	-0.076225566	-0.323935051
	-0.129069118	-0.121055319	-0.165603491	-0.187272519
	-0.130240962	-0.070657277	-0.201497106	-0.061424622
	-0.238926613	-0.042814563	-0.113345257	-0.278896055
	-0.313738686	-0.07619278	-0.001863565	-0.384094099
	-0.255484545	-0.124728129	-0.010284892	-0.521920577
	-0.099055456	-0.198100172	-0.092890477	0.19052116
	-0.155095995	-0.103067112	-0.130800032	-0.159643795
	0.078242639	0.034881325	-0.499904012	-0.116613808
	0.067284271	-0.087162524	-0.358099105	-0.123368568
	0.012929795	-0.347660521	-0.042208882	0.076238455
	-0.229679581	0.006194707	-0.152884104	-0.064659316
	-0.205426754	-0.133376395	-0.035305762	-0.304343585
	-0.050570667	-0.225176114	-0.09652978	-0.157342503
	-0.061613451	-0.229888013	-0.077649153	-0.272792746
	-0.138841026	-0.074353422	-0.152831834	-0.202282926
	-0.119575554	-0.215350245	-0.030606307	-0.194021431
	-0.069737554	-0.127111743	-0.166313567	-0.115323696
	-0.325174266	0.041514305	-0.078542493	-0.573363094
	-0.29937237	-0.240966128	0.183567489	-0.047389417
	-0.041150906	-0.112199096	-0.200482412	-0.086719152
	-0.030656885	-0.234690023	-0.088323433	0.059869068

	-0.365674206	-0.006187774	0.018208107	0.021136926
	-0.156633521	-0.191081071	-0.004322487	-0.583423498
	-0.011223118	-0.111296834	-0.227873205	-0.176893175
	-0.107275598	-0.133845538	-0.108138439	-0.287134608
	-0.105905094	-0.138128701	-0.099551094	-0.557502914
	0.057237218	-0.180906371	-0.21961335	-0.086804005
	-0.016503581	-0.154693148	-0.166887635	-0.438135466
