# Supplementary material for: Developing SHP2-based combination therapy for KRAS-amplified cancer
Source: JCI Insight. 2023 Feb 8;8(3):e152714. doi: 10.1172/jci.insight.152714 (PMC9977440; doi:10.1172/jci.insight.152714)
Supplement: Supplemental table 5 [file jciinsight-8-152714-s175.pdf]

NCBI Gene name	HUG1N_Average LFC	KE39_primary_Average LFC
EGFR	0.2546	-0.2114
ERBB2	0.0122	-0.0471
PTK2	-0.0034	-0.7104
SRC	-0.1177	0.0285
SHOC2	-0.7058	-1.0709
BRAF	-0.4098	-0.2072
RAF1	-0.1485	-0.6266
PTEN	0.4738	2.1262
NF1	1.8322	2.9933

KE39_secondary_Average LFC	CAT12_Average LFC	YCC1_Average LFC	GSU_Average LFC
-0.5911	-1.3959	1.2041	0.1168
-0.7536	-0.4147	0.0653	-0.0830
-0.4722	-0.4862	-0.1259	-0.5032
-0.3804	-0.5029	-0.2402	-0.2414
-0.9476	-0.3572	-1.2424	-0.7109
-0.8063	-0.0384	-0.5816	-0.8836
-0.6192	-0.0040	-0.5860	-0.6094
0.7447	0.6304	0.1324	-0.0324
3.1713	0.9328	1.9797	3.1370
